# Discovery of Novel 4-Hydroxyquinazoline Derivatives: In Silico, In Vivo and In Vitro Studies Using Primary PARPi-Resistant Cell Lines

**DOI:** 10.3390/molecules29061407

**Published:** 2024-03-21

**Authors:** Lijie Zhu, Binzhuo Liu, Feng Jin, Weilong Cao, Guangzhao Xu, Xinwei Zhang, Peng Peng, Dingding Gao, Bin Wang, Kairui Feng

**Affiliations:** 1 School of Pharmacy, Shandong Second Medical University, Weifang 261053, China; 2 School of Pharmacy (Preparatory), East China Normal University, Shanghai 200241, China; 3 The Research Center of Chiral Drugs, Innovation Research Institute of Traditional Chinese Medicine, Shanghai University of Traditional Chinese Medicine, Shanghai 201203, China

**Keywords:** PARPi, 4-Hydroxyquinazoline, anti-tumor, primary drug-resistant

## Abstract

A series of novel 4-Hydroxyquinazoline derivatives were designed and synthesized to enhance sensitivity in primary PARPi-resistant cells. Among them, the compound **B1** has been found to have superior cytotoxicity in primary PARPi-resistant HCT-15 and HCC1937 cell lines, and dose-dependently suppressed the intracellular PAR formation and enhanced the γH2AX aggregation. Mechanistic study showed that **B1** stimulated the formation of intracellular ROS and the depolarization of the mitochondrial membrane, which could increase apoptosis and cytotoxicity. An in vivo study showed that **B1** significantly suppressed tumor growth at a dose of 25 mg/kg, and an acute toxicity study confirmed its safety. Molecular docking and dynamics simulations revealed that hydrogen bonding between **B1** and ASP766 may be helpful to enhance anti-drug resistance ability. This study suggests that **B1** is a potent PARP inhibitor that can overcome PARPi resistance and deserves further investigation.

## 1. Introduction

Poly(ADP-ribose) polymerase (PARP) is a key enzyme located in the nucleus, and its main functions include repairing single-stranded DNA breaks and maintaining chromosome integrity [1]. PARP can impact the PARylation of different nuclear proteins, such as histones, RNA polymerases, DNA polymerases, and DNA ligases. Among its 18 subtypes, PARP1 is responsible for 90% of the PARylation events linked to the repair of DNA damage [2]. In addition, it is the main substrate of Caspase-3 and plays a key role in cell apoptosis [3]. PARP can alter its conformation to respond to DNA damage; when DNA is damaged, the HD and ART domains are progressively detached, then NAD^+^ enters the catalytic pocket, resulting in the production of ADP-ribose and modification of the substrate to attract DNA repair proteins and complete DNA repair [4,5,6].

**Figure 1:**
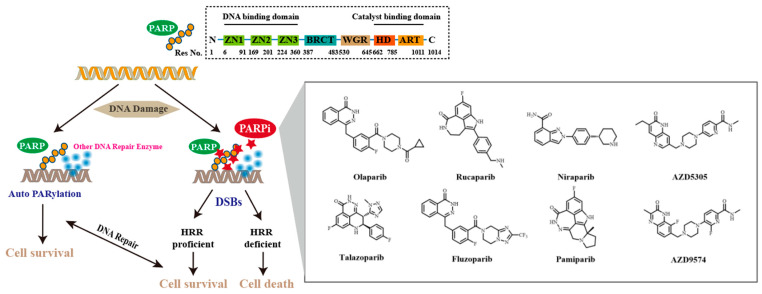
The domains of PARP, mechanism of action of PARPi, and chemical structures of representative PARP inhibitors.

Homologous recombination repair (HRR) is a critical mechanism for correcting DNA double-stranded breaks (DSBs) and is a type of DNA repair that maintains genome integrity and ensures that genetic information is inherited with high fidelity. BRCA1/2 play a significant role in the HRR pathway. Together with PARP, they provide double insurance to ensure accurate DNA replication in vivo. But the HRR pathway is defective in BRCA1/2-mutant cells, where the restriction of PARP function can cause severe genomic instability, rendering them inviable (Figure 1). The synthetic lethality effects of BRCA and PARP provide unique opportunities for targeted therapy [7,8,9]. To date, the FDA has approved six PARP inhibitors, including Olaparib, Rucaparib, Niraparib, Tarazoparib, Fluzoparib, and Pamiparib, for the treatment of BRCA1/2-mutated ovarian, breast, and pancreatic cancer, and the second-generation drugs entering clinical studies are AZD5305 and AZD9574 developed by AstraZeneca (Figure 1). Additionally, clinical trials of PARPis for prostate cancer, gastric cancer, and non-small-cell lung cancer have progressed, and PARPis are also being investigated for the treatment of esophageal, colorectal, endometrial, and other cancers [10,11,12,13].

Due to the DNA repair defect, BRCA1/2-deficient tumor cells are more sensitive to PARP inhibitors (PARPi) through the mechanism of synthetic lethality. However, PARPi resistance is ubiquitous in the clinic [14,15,16,17]. More than 40% BRCA1/2-mutant patients fail to respond to PARPi, especially Olaparib, causing minimal synthetic lethality in vitro in BRCA1-mutant human breast cancer HCC1937 and BRCA2-mutant human colorectal adenocarcinoma HCT-15 cells [18,19,20]. However, in mouse xenograft models, they were only moderately effective in reducing tumors. Therefore, HCC1937 and HCT-15 cell lines can be used as model cell lines for the screening of new compound structures for addressing drug resistance to existing PARPi drugs [21,22,23].

In this study, our in-house compound library was initially screened in primary PARPi-resistant HCT-15 and HCC1937 cell lines (see Supplementary Materials Tables S1 and S2). Among them, compared with Olaparib (IC_50_ = 45.53 ± 3.13 μM, against HCT-15; IC_50_ = 37.07 ± 1.89 μM against HCC1937), the compound **IN17** (IC_50_ = 33.45 ± 1.79 μM against HCT-15; IC_50_ = 34.29 ± 2.68 μM against HCC1937) with a 4-Hydroxyquinazoline scaffold displayed the most potent inhibitory activity against both cell lines. **IN17** is a fragment that has been used by previous groups in the design of antioxidant drugs but has not been the subject of further investigation. **IN17** showed potential in resisting PARP1 (IC_50_ = 0.47 ± 0.0032 μM). Although 4-Hydroxyquinazoline analogues such as PARPi have been reported [24,25,26,27], **IN17** is a completely new structure; we expect to obtain a lead compound that can resist primary resistance and enhance anti-tumor effects by using **IN17** as a template molecule for further modification.

## 2. Results and Discussion

### 2.1. Design Strategy Based on ***IN17***

**Figure 2:**
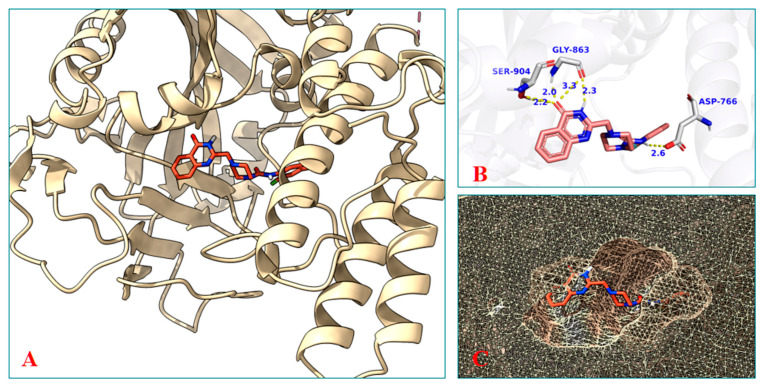
The docking of the compound **IN17** (PDB:7KK4). (**A**) **IN17** binding sites in proteins. (**B**) Hydrogen bonding of **IN17**. (**C**) **IN17** space conformation.

We firstly used MOE to perform a molecular docking analysis of **IN17**. **IN17** retained the Z-shape in the protein and maintained the necessary hydrogen bonding of the PARPi with the residues GLY863 and SER904 (Figure 2). Importantly, the urea group of **IN17** generated a new hydrogen bond with the residue ASP766. To determine the necessity of the hydrogen bonding between the urea group and the residue ASP766, we designed and synthesized five different compounds, **IN17(1-5)**, by replacing the urea group with methylene, acyl, sulfonyl, and thiourea groups as new linkers. However, their anti-proliferative activities against both cell lines were not as good as those of **IN17**, or were significantly reduced (see Supplementary Materials Table S3), suggesting that the urea group was an important pharmacophore. Therefore, we kept the urea in **IN17** and designed three additional series of compounds.

**Figure 3:**
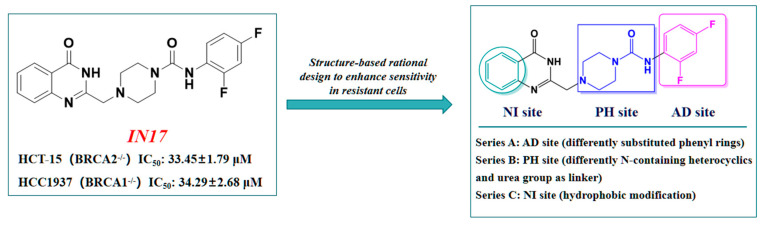
Design strategy of the target compound **IN17**.

Firstly, different substituents were introduced on the benzene ring of the AD site of Series A compounds in search of a compound that could enhance the hydrogen bonding interaction between urea and ASP766. After screening Series A compounds, our goal was to design new B- and C-series compounds at the PH and NI sites, which could help to maintain a better binding mode between the compound and the protein, increase the anti-proliferative activities on the two cell lines, and identify a new lead compound (Figure 3).

### 2.2. Chemistry

**Scheme 1:**
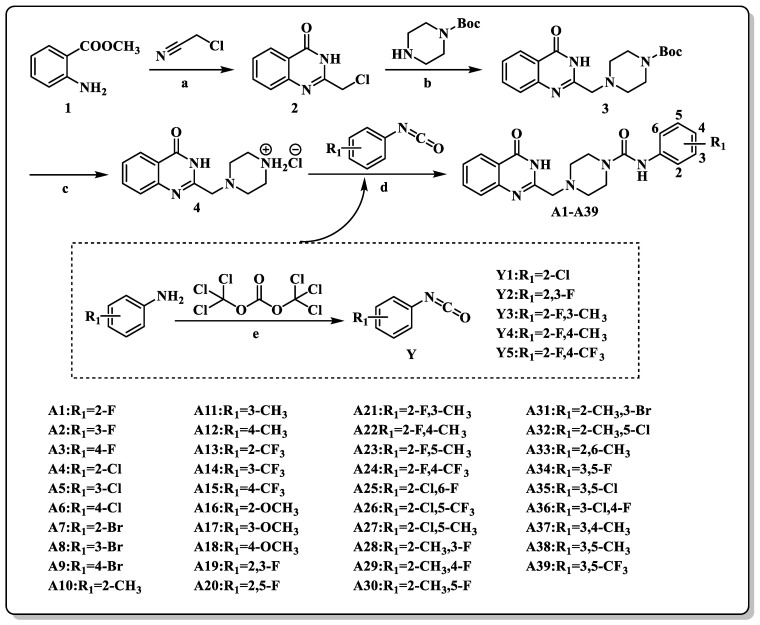
Synthesis of the compounds **A1**–**A39**. Reagents and conditions: (a) HCl/Dioxane, 80 °C, 12 h; (b) K_2_CO_3_, 95% Ethanol, rt, 12 h; (c) HCl/Dioxane, rt, 1 h; (d) Et_3_N, THF, rt, 2–4 h; (e) Toluene, Et_3_N, 0 °C to 100 °C, 2 h.

The synthetic route for Series A compounds is presented in Scheme 1. The commercially available methyl 2-aminobenzoate (**1**) was reacted with chloroacetonitrile under acidic conditions to obtain the intermediate **2**. Then, treatment of the compound **2** with 1-Boc-piperazine under basic conditions generated the compound **3**. The Boc protective group was removed from the compound **3** by using HCl/Dioxane, which resulted in the formation of the compound **4**. This compound was reacted directly with various isocyanate derivatives containing unique substituents and acquired the target compounds **A1**–**A39**. For the isocyanate derivatives **Y1**–**Y5**, they were synthesized by reacting aniline derivatives with triphosgene.

**Scheme 2:**
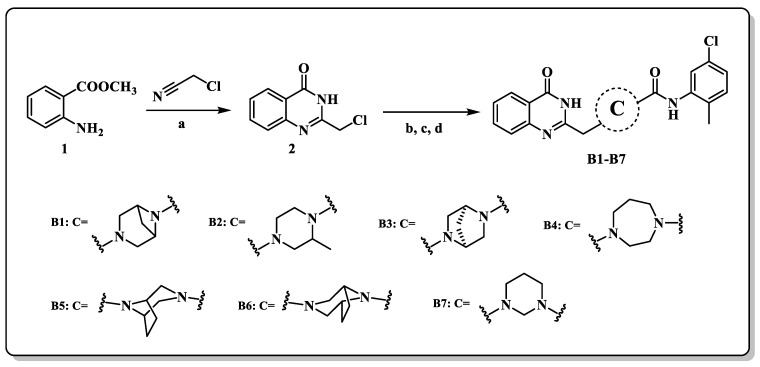
Synthesis of the compounds **B1**–**B7**. Reagents and conditions: (a) HCl/Dioxane, 80 °C, 12 h; (b) *N*-Boc-protected *N*-containing heterocyclics, K_2_CO_3_, 95% Ethanol, rt, 12 h; (c) HCl/Dioxane, rt, 1 h; (d) 5-Chloro-2-methylphenylisocyanate, Et_3_N, THF, rt, 2–4 h.

**Scheme 3:**
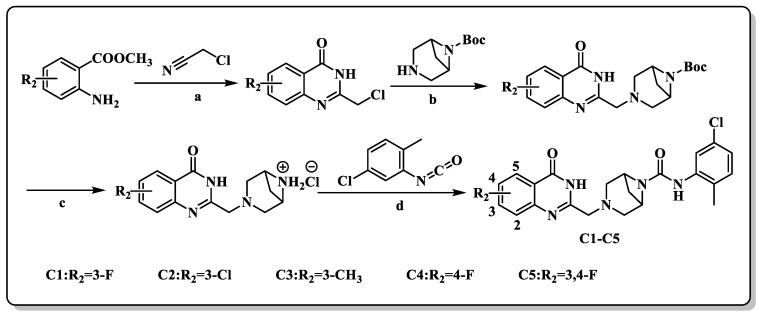
Synthesis of the compounds **C1**–**C5**. Reagents and conditions: (a) HCl/Dioxane, 80 °C, 12 h; (b) K_2_CO_3_, 95% Ethanol, rt, 12 h; (c) HCl/Dioxane, rt, 1 h; (d) 5-Chloro-2-methylphenylisocyanate, Et_3_N, THF, rt, 2–4 h.

The synthetic route of Series B compounds was similar with the synthesis of Series A compounds. As shown in Scheme 2, the compound **2** was reacted with differently *N*-Boc-protected *N*-containing heterocyclics to the corresponding intermediates. Finally, the target compounds **B1**–**B7** were obtained by reacting intermediates with 5-Chloro-2-methylphenylisocyanate. The synthetic routes of the compounds **C1**–**C5** were synthesized in the similar way as the compound **B1** except that the reactants were replaced by derivatives of methyl 2-aminobenzoate (Scheme 3).

### 2.3. Biological Evaluation

#### 2.3.1. In Vitro Anti-Proliferative Activities against HCT-15 and HCC1937 Cell Lines

The newly synthesized target compounds (Series A–C) were evaluated in vitro using primary PARPi-resistant cells HCT-15 and HCC1937, with Olaparib as the positive control. For the Series A compounds, different substitutes, such as halogen, methyl, trifluoromethyl, and methoxy, were installed on the right benzene ring, and the results are shown in Table 1. We found that compared with **IN17**, the anti-proliferative activities of most compounds were improved in HCT-15 cell lines, but it was found that the existence of substituents at the 4-position or the introduction of bromine into the benzene ring were not conducive to improve the anti-proliferation activity in HCC1937 cell lines. When the 2- and 3- positions were substituted by fluorine, chlorine, and methyl, the compounds **A1**, **A4**, and **A10** showed a better anti-proliferation activity compared with **IN17**.

According to the above results, we fixed fluorine, chlorine, and methyl at the 2- or 3- positions on the benzene ring, and introduced another substituent group at another position; thus, **A19**–**A39** were designed and synthesized. We found that the inhibitory activity of the compounds **A19**–**A39** against two cell strains could be maintained when the 5-position was substituted with methyl, fluorine, and chlorine groups after fixing the substitution at the 2-position, but the inhibitory activity of **A26** against HCC1937 was significantly reduced when the electronegative trifluoromethyl group was substituted with a large electronegative substitution at the 5-position. As with the monosubstituted compounds, the introduction of bromine on the benzene ring significantly reduced the inhibitory activity against both cell lines. Overall, the double substitution on the benzene ring still improved the inhibitory activity against both cell lines, especially the compound **A32** (IC_50_ = 10.93 ± 0.71 μM, against HCT-15; IC_50_ = 11.35 ± 0.73 μM against HCC1937) which exhibited better anti-proliferative activity compared with other compounds in both cell lines. 

Subsequently, the compound **A32** was used as a template for further structural optimization. Replacing the piperazine moiety with spirals, homopiperazine, or hexahydropyrimidine, Series B compounds (**B1**–**B7**) were designed and synthesized. As shown in Table 2, the compounds **B1**–**B6** exhibited potent anti-proliferative activities against two cancer cell lines. Specially, the compound **B1** (IC_50_ = 2.89 ± 0.78 μM, against HCT-15; IC_50_ = 3.26 ± 0.38 μM against HCC1937) exhibited the highest anti-proliferative activities, which were superior to Olaparib. Based on the compound **B1**, we then designed Series C compounds with some substituent group on the left benzene ring (**C1**–**C5**). However, no compounds showed better anti-proliferative activities than the compound **B1** (Table 3).

The IC_50_ values with drug treatment for 7 days against two cell lines were also tested (Table 4). Compared with Olaparib, **B1** showed the most potent time-dependent anti-proliferative activity. Meanwhile, the anti-proliferative activities were also tested in normal human breast cancer cells (MDA-MB-231), human normal hepatocytes (LO2), and human normal colonic epithelial cells (NCM460). The results indicated that **B1** exhibited more potent inhibitory activity than Olaparib in MDA-MB-231 cell lines, while showing no significant toxicity to normal cells, and had a good selectivity in normal and cancer cell lines (Table 4). We finally evaluated the ability of the compound **B1** to inhibit PARP1 activity in vitro, and the results are shown in Table 4. **B1** exhibited better inhibitory effects against PARP1 with IC_50_ values of 63.81 ± 2.12 nM. Although the IC_50_ values of **B1** were weaker than those of Olaparib, the inhibitory activity of less than 100 nM also proved that the compound **B1** can effectively inhibit PARP1 in vitro and bind efficiently to the enzymes.

Therefore, it is necessary to conduct comprehensive in vitro and in vivo anti-tumor research on the compound **B1**. This will provide a crucial foundation for the development of new drug-resistant PARP inhibitors.

#### 2.3.2. The Influence of B1 on the Expression of PAR in HCT-15 and HCC1937 Cell Lines

**Figure 4:**
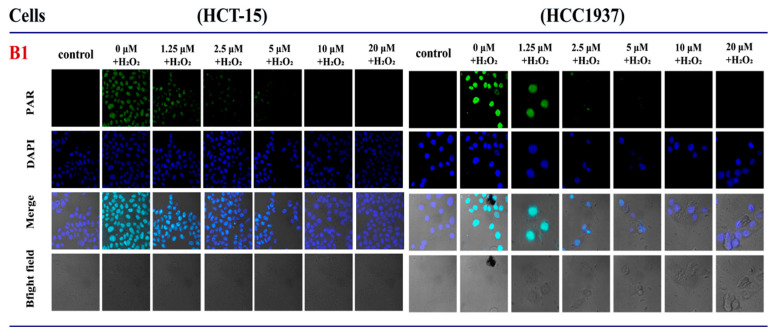
Immunofluorescence analysis of the changes in the formation of PAR in H_2_O_2_-treated HCT-15 and HCC1937 cell lines by different concentrations (1.25, 2.5, 5, 10, and 20 μM) of **B1**. Scale bar, 50 µm.

The effects of the compound **B1** on PAR aggregation in H_2_O_2_-treated HCT-15 and HCC1937 cell lines were investigated through immunofluorescence experiments [28,29]; PAR was denoted by green fluorescence. Figure 4 illustrates that in the absence of inhibitors, a large amount of green fluorescence was produced in two cell lines, and with an increase in **B1** concentration, the green fluorescence gradually decreased, Especially, when the concentration of **B1** was 1.25 μM, the green fluorescence obviously weakened, and when the concentration of **B1** reached 5 μM, the green fluorescence almost disappeared in two cell lines. This demonstrated that **B1** can effectively target and inhibit the function of PARP at low concentrations and interfere with DNA repair.

#### 2.3.3. The Influence of **B1** on the Expression of γH2AX in HCT-15 and HCC1937 Cell Lines

**Figure 5:**
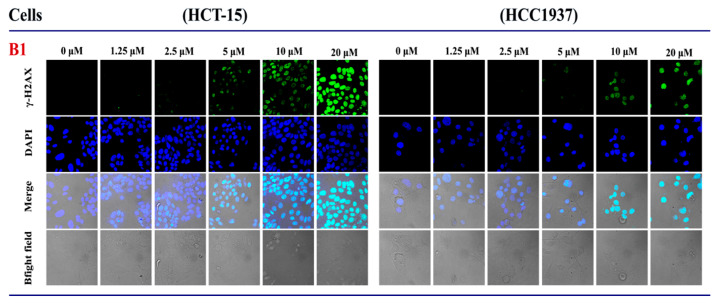
Immunofluorescence analysis of the changes in the formation of γH2AX in HCT-15 and HCC1937 cell lines by different concentrations (1.25, 2.5, 5, 10, and 20 μM) of **B1**. Scale bar, 50 µm.

γH2AX is closely related to DSBs, and quantitative detection of the γH2AX expression level can be used to evaluate the degree of DNA damage [30,31]. After treatment with five different concentrations of **B1** (1.25, 2.5, 5, 10, and 20 μM), the expression of γH2AX was studied in the HCT-15 and HCC1937 cell lines. As shown in Figure 5, **B1** can dose-dependently increase the expression of γH2AX, as indicated by the presence of green fluorescence. Particularly, the expression of γH2AX was significantly increased in both cell lines with the concentration of 10 μM. These data demonstrated that **B1** effectively induced the accumulation of cytotoxic DSBs and exerted synthetic lethality in BRCA1/2 mutant cells.

#### 2.3.4. Effects of **B1** on Apoptosis in HCT-15 and HCC1937 Cell Lines

**Figure 6:**
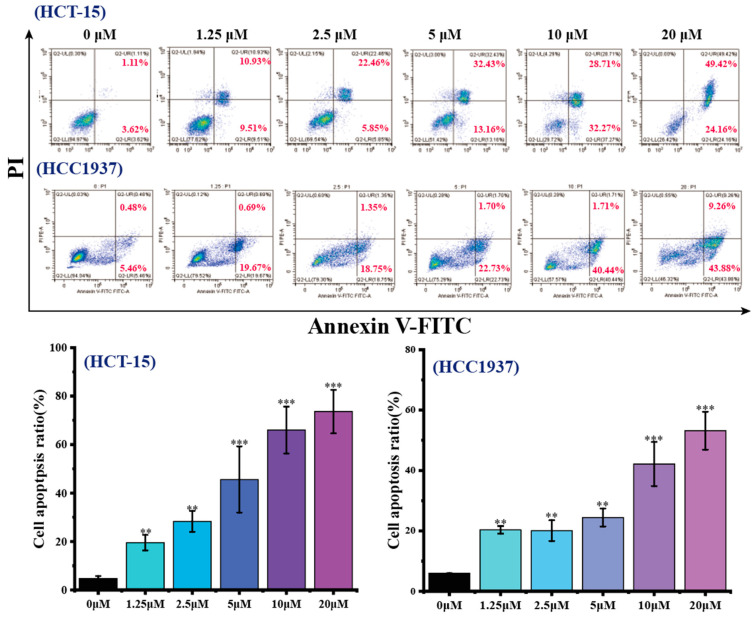
Effects of different concentrations (1.25, 2.5, 5, 10, and 20 μM) of **B1** on the apoptosis of HCT-15 and HCC1937 cell lines. Data are expressed as the mean ± SD of three independent experiments, ** *p* < 0.01, *** *p* < 0.001 as compared with control. Scale bar, 50 µm.

We explored the potential of **B1** to induce apoptosis in HCT-15 and HCC1937 cell lines using flow cytometry. As illustrated in Figure 6, **B1** can induce the cell apoptosis in a concentration dependent manner in both cell lines. This effect was more pronounced in HCT-15 cell lines compared with HCC1937 cell lines. In detail, **B1** can increase the apoptosis rate to 73.58% at a concentration of 20 μM in HCT-15 cell lines. In HCC1937 cells, the apoptosis rate of cells reached 53.14% after treatment with **B1** at a concentration of 20 μM.

#### 2.3.5. Effects of **B1** on the Expression of Apoptosis-Related Proteins in HCT-15 and HCC1937 Cell Lines

**Figure 7:**
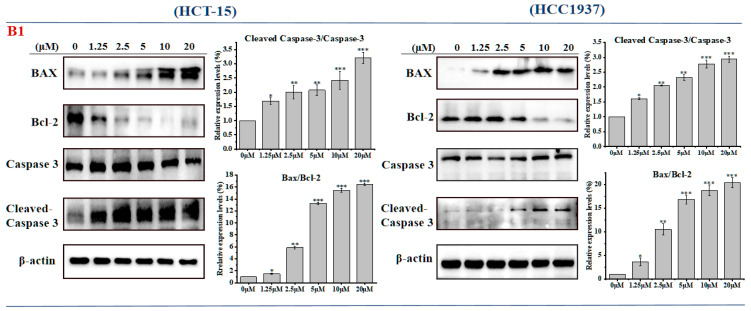
Western blot analysis of the different concentrations (1.25, 2.5, 5, 10, and 20 μM) of **B1** on the expression of apoptosis-related proteins of HCT-15 and HCC1937 cell lines. * *p* < 0.1,** *p* < 0.01, *** *p* < 0.001 as compared with control.

To further probe the mechanism of apoptosis induced by **B1**, Western blot analysis was performed to evaluate the expression of apoptosis-related proteins, including Caspase-3, cleaved Caspase-3, Bax, and Bcl-2, at the different concentrations. As shown in Figure 7, **B1** considerably increased Bax expression, decreased Bcl-2 expression, and activated Caspase-3 in both HCT-15 and HCC1937 cell lines. These results further substantiated the ability of **B1** to induce apoptosis in primary PARPi-resistant HCT-15 and HCC1937 cell lines, even at low concentrations.

#### 2.3.6. Effects of **B1** on Intracellular ROS Levels in HCT-15 and HCC1937 Cell Lines

**Figure 8:**
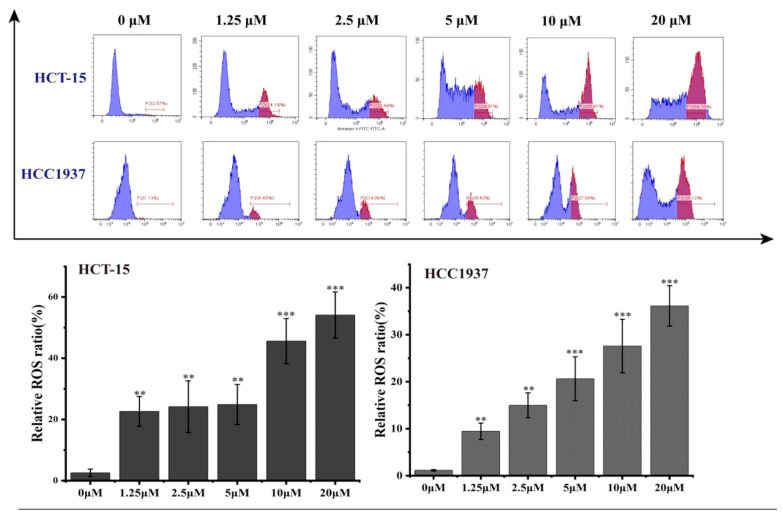
Effects of different concentrations (1.25, 2.5, 5, 10, and 20 μM) of **B1** on ROS generation in HCT-15 and HCC1937 cell lines by flow cytometry. Data are expressed as the mean ± SD of three independent experiments, ** *p* < 0.01, *** *p* < 0.001 as compared with the control.

**Figure 9:**
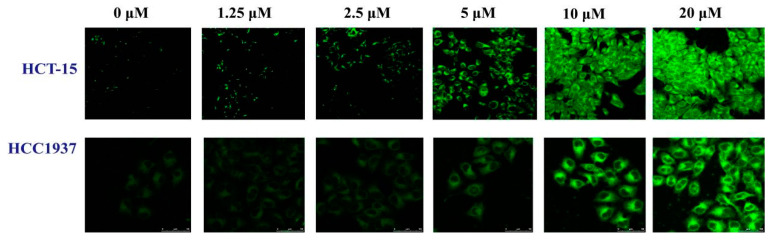
Immunofluorescence analysis of the ability of different concentrations (1.25, 2.5, 5, 10, and 20 μM) of **B1** to induce ROS in HCT-15 and HCC1937 cell lines. Scale bar, 50 µm.

ROS are often produced in the process of DNA damage and it is important to consider their potential role [32,33]. To further study the mechanisms of **B1** sensitizing drug-resistant cells, the level of ROS in HCT-15 and HCC1937 cell lines was analyzed by the fluorescence probe DCFH-DA and flow cytometry. As shown in Figure 8 and Figure 9, **B1** can significantly increase ROS levels in both cell lines at a concentration of 2.5 μM, and under the intervention of **B1** with the concentration of 20μM, green fluorescence increased significantly, and ROS ratios reached 54.10% and 36.12% in the two cell lines, respectively. These findings provided a possibility that **B1** enhances the cytotoxic efficacy against resistant cells by inducing cytotoxic ROS generation and accelerating cell apoptosis.

#### 2.3.7. Effects of **B1** on Mitochondrial Membrane Potential in HCT-15 and HCC1937 Cell Lines

**Figure 10:**
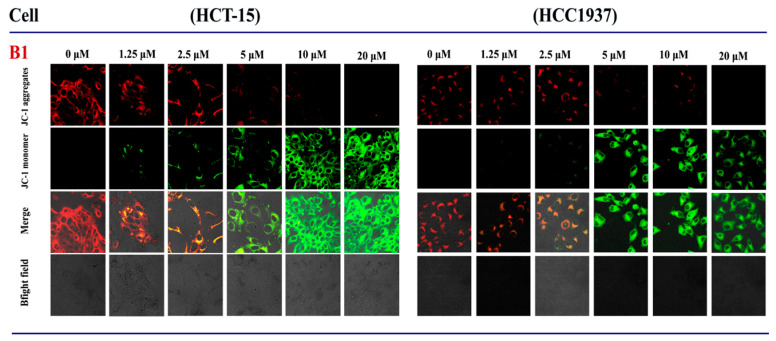
Immunofluorescence analysis of the detection of mitochondrial membrane potential in HCT-15 and HCC1937 cell lines by different concentrations (1.25, 2.5, 5, 10, and 20 μM) of **B1**; when the mitochondrial membrane potential is high, JC-1 forms aggregates inside the mitochondrial matrix, resulting in JC-1 aggregates that emit red fluorescence. When there is a decrease in the mitochondrial membrane potential, JC-1 remains in its monomeric form, resulting in the emission of green fluorescence. Scale bar, 50 µm.

The accumulation of ROS in cells induces oxidative stress, leading to changes in mitochondrial membrane permeability. We used the JC-1 probe to detect the changes in mitochondrial membrane potential of two cell lines under the intervention of different concentrations of **B1**. According to Figure 10, JC-1 mainly existed in the mitochondrial matrix in the form of aggregation and emitted strong red fluorescence without any chemical interference, and the intensity of the green fluorescence was extremely weakened.

With increasing concentrations of **B1**, the mitochondrial membrane potential decreased, resulting in a marked decrease in red fluorescence and a concomitant increase in green fluorescence in the cytoplasm. Importantly, at a concentration of 5 μM, green fluorescence began to change significantly in both cell lines, and at 20 μM the intensity of red fluorescence was hardly visible, indicating that **B1** not only accelerated the accumulation of ROS, but also effectively stimulated the depolarization of the mitochondrial membrane, expediting cellular apoptosis.

#### 2.3.8. In Vivo Study of **B1**

**Figure 11:**
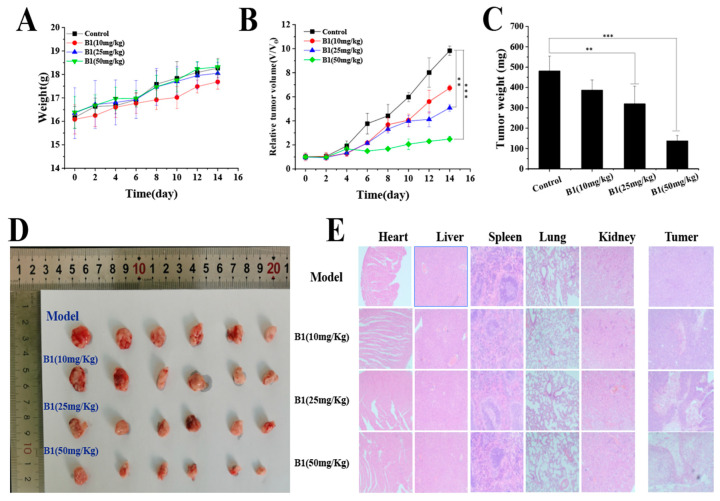
Anti-tumor activity of **B1** in vivo. (**A**) The average weight of the treated and control mice was recorded every 2 days. (**B**) Tumor growth curve. (**C**) Tumor average weight at the endpoint of the study. (**D**) Images of excised tumors at the endpoint of the study. (**E**) The H&E staining of the major organs of mice and the tumor in the in vivo anti-tumor study. ** *p* < 0.01, *** *p* < 0.001 as compared with the control. Scale bar, 50 µm.

To further evaluate the in vivo anti-tumor efficacy of the compound **B1**, an HCT-15 nude mouse xenograft model was used. After establishing the solid tumors, the compound **B1** was intraperitoneally administered at three doses (10, 25, and 50 mg/kg) once daily for 14 consecutive days. The tumor volume and mouse body weight were recorded, and the corresponding tissues were analyzed. As shown in Figure 11, no significant changes in mouse body weight were observed, suggesting that the compound **B1** was safe at these dosages (Figure 11A). Notably, the compound **B1** significantly inhibited xenograft tumor growth at doses of 25 mg/kg and 50 mg/kg, with evident reductions in both tumor volume and weight (Figure 11B–D). Moreover, no significant histopathological abnormalities were found in the heart, liver, spleen, lungs, and kidneys. Tumor cells in the treated groups exhibited irregular morphological alterations and severe vacuolization at doses of 10 mg/kg (Figure 11E), confirming the anti-tumor effect of the compound **B1** in vivo.

#### 2.3.9. Acute Toxicity Study of **B1**

**Figure 12:**
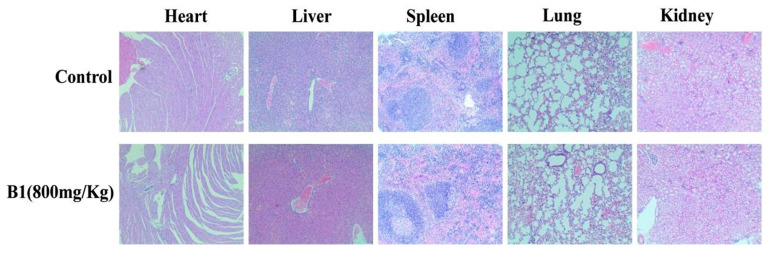
The H&E staining of the major organs of mice in the in vivo acute toxicity study. Scale bar, 50 µm.

After confirming **B1** had significant in vitro and in vivo anti-tumor effects, we performed an acute toxicity study in mice to assess its in vivo safety profile. The mice were administered a single intraperitoneal injection dose of 800 mg/kg and were constantly monitored for 14 days. The results showed that there was no significant reduction in body weight in the treated mice compared to the control group. Also, there were no significant changes in heart, liver, spleen, lung, or kidney for those mice (Figure 12), providing important evidence to support the safety profile of the compound **B1**.

#### 2.3.10. Molecular Docking Study of the Compound **B1**

The discovery of the compound **B1** indicated substantial potential for overcoming primary resistance and demonstrated the effectiveness of our design strategy. We conducted an in-depth docking study on the mechanism of effectiveness of the compound **B1** [34].

**Figure 13:**
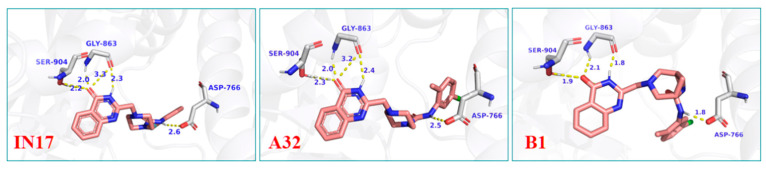
The docking of the compounds **IN17, A32**, and **B1** (PDB:7KK4).

**Figure 14:**
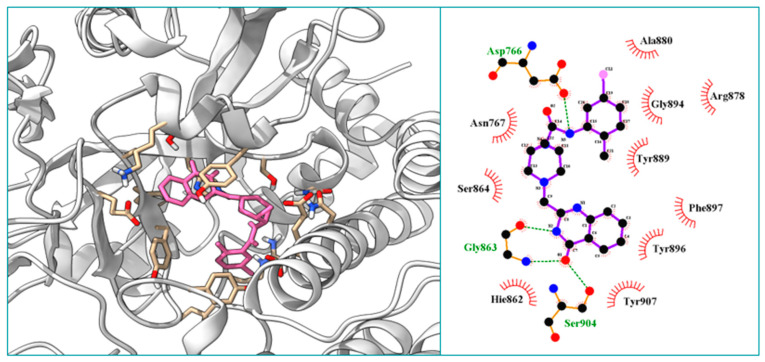
The binding mode and 2D interaction of the compound **B1** in the protein (PDB:7KK4).

As shown in Figure 13, compounds **IN17**, **A32**, and **B1** maintained vital hydrogen-bonding interactions with the residues GLY863, SER904, and ASP766 (Figure 13). However, the compounds have different spatial configurations in the protein. The compound **B1** is T-shaped due to the bridging ring, and its disubstituted benzene ring extends to the helical region at the AD site (Figure 14). Compared to the other two compounds, **B1** formed the shortest hydrogen bond between ASP766 and the urea group, with a distance of 1.8 Å. The hydrogen bonds between **B1** and GLY863 and SER904 were also shortened, resulting in a unique spatial conformation that enhances protein binding. This suggested that the existence of shorter hydrogen bonds between **the** compound and ASP766 may enhance its sensitivity to drug-resistant cells.

#### 2.3.11. Molecular Dynamics Study of the Compound **B1**

To further investigate whether this hydrogen bond between **B1** and ASP766 is stable, molecular dynamics simulations of **B1** and PARP complexes were carried out for 30 ns [35].

**Figure 15:**
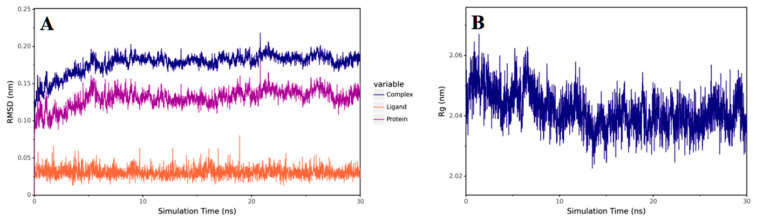
(**A**) The RMSD of the ligand (tangerine yellow), protein (violet), and complex (blue). (**B**) The Rg of the complex.

The root mean square deviation (RMSD) and the radius of gyration (Rg) are both important criteria for objectively evaluating system stability and overall structural changes. Figure 15 showed the results of the 30 ns simulation; there were minimal fluctuations in RMSD/Rg between **B1** and the protein, indicating a high level of overall stability for the complex.

**Figure 16:**
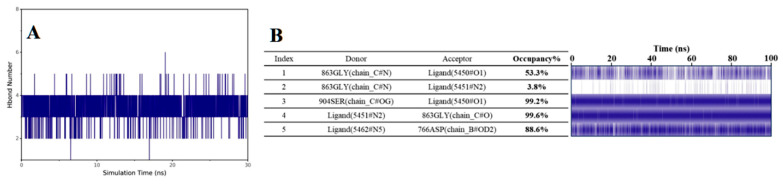
(**A**) The Hbond number of the complex. (**B**) The occupancy of the complex.

As shown in Figure 16, the data clearly indicated that an oxygen atom **B1** formed a strong hydrogen bond with SER904, GLY863, and ASP766, displaying an impressive occupancy rate of 99.2%, 99.6%, and 88.6%. These results highlight the strong stability of the hydrogen bonds, which further supports the strong binding interaction between **B1** and the PARP protein, and the importance of the direct hydrogen bond between **B1** and ASP766 was also proved by its stability.

**Figure 17:**
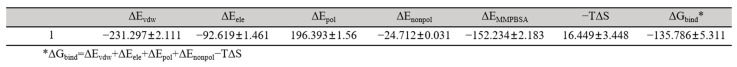
The binding energy of the complex.

We selected stable trajectories of the complex for energy analysis using the MM-PBSA method. As shown in Figure 17, the van der Waals interaction energy, ΔEvdw, surpasses the electrostatic interaction energy, ΔEele, by a factor of 2.5 and exceeds the hydrophobic interaction energy, ΔEnonpol, by a factor of 9 [36]. This discernment underscores the dominant role of van der Waals interactions, ΔEvdw, followed by electrostatic interactions, ΔEele, as secondary contributors, with hydrophobic interactions, ΔEnonpol, playing a supplementary role. This heightened binding energy observed between **B1** and the protein serves as a robust indicator of the substantial affinity between these two entities.

**Figure 18:**
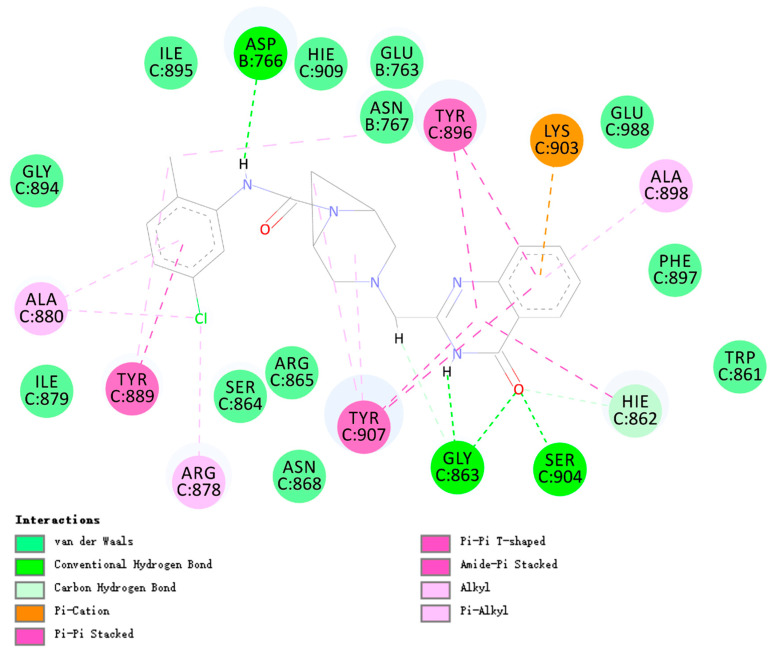
Interaction forces between **B1** and proteins in the steady state (2D).

After analyzing the final structure of the stable complex between **B1** and the protein, it is clear that crucial hydrogen-bonding interactions remain with the protein residues ASP766, GLY863, and SER904 (Figure 18). Moreover, **B1** has carbon–hydrogen bond interactions with HIE862 and different hydrophobic interactions, comprising Pi–Pi T-shaped interactions with TYR907, TYR889, TYR896, and HIE862, as well as amide–Pi stacked, Pi–Pi stacked, and Pi–cation interactions with LYS903. Alkyl and Pi–alkyl hydrophobic interactions were observed with residues ARG878, ALA880, ALA898, and ASN767. In addition, van der Waals interactions were observed between the residues ILE879, HIE909, and PHE897. These varied interactions underlie the secure binding of small molecules to proteins, which is essential to their anti-tumor activity.

## 3. Materials and Method

### 3.1. Chemistry

All the chemical reagents employed were obtained from commercial suppliers without further purification. Thin-layer chromatography (TLC) was carried out on silica gel GF254 and observed with UV light (254 nm). The silica gel used in the chromatography column was 200–300 mesh, and the melting points of all compounds were observed on the melting point instrument. Mass spectrometry was determined by Thermo Scientific™Q Exactive™. All the ^1^H NMR and ^13^C NMR spectra were determined by AVANCE NEO 400 MHz (Bruker) using CDCl_3_ and DMSO-d_6_ as a deuterium reagent. Chemical shifts were expressed in ppm relative to tetramethylsilane (TMS) as an internal standard, and the coupling constants were depicted in hertz (Hz) with multiplicities denoted as s (singlet), d (doublet), t (triplet), q (quartet), and m (multiplet).

#### 3.1.1. General Synthetic Procedures for the Synthesis of the Compound **2**

To a solution of Methyl 2-aminobenzoate (1.51 g, 10 mmol) in 4 N HCl-dioxane solution, chloroacetonitrile (2.26 g, 30 mmol) was added and the mixture was stirred at 80 °C for 12 h. After cooling to room temperature, the reaction solution was collected and dissolved in water and neutralized with sodium hydroxide to pH = 7. The solids were collected by filtration, and washed with water and dried to give the target product. White solid, yield 51.6%. ^1^H NMR (400 MHz, CDCl_3_) δ 9.45 (s, 1H), 8.30 (d, *J* = 7.1 Hz, 1H), 7.80 (s, 1H), 7.70 (d, *J* = 8.7 Hz, 1H), 7.54 (s, 1H), 4.59 (s, 2H).

#### 3.1.2. General Synthetic Procedures for the Synthesis of the Compound **3**

To a solution of 2-chloromethyl-4-(3H)-quinazolinone (0.39 g, 2 mmol) in 95% ethanol, *N*-Boc-piperazine (0.75 g, 4 mmol) and potassium carbonate (0.83 g, 6 mmol) were added. The mixture was stirred at room temperature for 12 h. After completion of the reaction, 95% ethanol was removed under vacuo, then 60 mL water was added, extracted with ethyl acetate (30 mL × 3), and washed with saturated sodium chloride (30 mL). The combined organic layer was dried on anhydrous sodium sulfate and concentrated in vacuo. The residue was purified by column chromatography on silica gel. Pure fractions were collected and concentrated to obtain the target product. White solid, yield 55.8%. ^1^H NMR (400 MHz, CDCl_3_) δ 9.94 (s, 1H), 8.28 (d, *J* = 7.9 Hz, 1H), 7.77 (t, *J* = 7.6 Hz, 1H), 7.66 (d, *J* = 8.1 Hz, 1H), 7.49 (t, *J* = 7.5 Hz, 1H), 3.57 (d, *J* = 17.6 Hz, 2H), 3.51 (s, 4H), 2.56 (s, 4H).

#### 3.1.3. General Synthetic Procedures for the Synthesis of the Compound **4**

To a solution of 4-((4-oxo-3,4-dihydroquinazolin-2-yl)methyl)piperazine-1-carboxylate (0.34 g, 1 mmol) in dichloromethane, 4N HCl-dioxane solution was added, and the mixture was stirred at room temperature for 1 h. The resulting mixture was concentrated in vacuo to obtain the crude target product, and this crude target product was used for the next step without any treatment.

#### 3.1.4. General Synthetic Procedures for the Synthesis of the Compounds **A1**–**A39**

To a solution of 2-(piperazin-1-ylmethyl)-4-(3H)-quinazolinone (0.24 g, 1 mmol) in tetrahydrofuran, phenyl isocyanate derivatives and triethylamine (0.30 g, 3 mmol) were added. The mixture was stirred at room temperature for 4 h. The organic phases were combined by extraction with dichloromethane and water, and the target compounds were obtained by column chromatography.

##### *N*-(2-fluorophenyl)-4-((4-oxo-3,4-dihydroquinazolin-2-yl)methyl)piperazine-1-carboxamide (**A1**)

White solid, yield 46.7%, m.p. 192.5–194.2 °C. ^1^H NMR (400 MHz, CDCl_3_) δ 10.21 (d, *J* = 33.5 Hz, 1H), 8.28 (d, *J* = 7.8 Hz, 1H), 8.04 (s, 1H), 7.77 (t, *J* = 7.5 Hz, 1H), 7.68 (d, *J* = 8.1 Hz, 1H), 7.49 (t, *J* = 7.3 Hz, 1H), 7.13–6.95 (m, 3H), 6.65 (s, 1H), 3.64 (s, 2H), 3.62 (s, 4H), 2.68 (s, 4H). ^13^C NMR (100 MHz, CDCl_3_) δ 161.89, 154.18, 153.90, 152.72, 151.51, 148.77, 134.89, 127.13, 126.55, 124.57, 123.25, 121.72, 114.68, 60.63, 52.91, 43.98. ESI-MS: calculated for C_20_H_20_FN_5_O_2_ [M+H]^+^ 382.16010, found 382.15900.

##### *N*-(3-fluorophenyl)-4-((4-oxo-3,4-dihydroquinazolin-2-yl)methyl)piperazine-1-carboxamide (**A2**)

White solid, yield 52.2%, m.p. 189.5–192.1 °C. ^1^H NMR (400 MHz, CDCl_3_) δ 9.95 (s, 1H), 8.28 (d, *J* = 7.8 Hz, 1H), 7.78 (t, *J* = 7.6 Hz, 1H), 7.67 (d, *J* = 8.1 Hz, 1H), 7.50 (t, *J* = 7.5 Hz, 1H), 7.30 (d, *J* = 11.0 Hz, 1H), 7.22 (dd, *J* = 15.1, 7.5 Hz, 1H), 7.02 (d, *J* = 8.1 Hz, 1H), 6.74 (t, *J* = 8.1 Hz, 1H), 6.56 (s, 1H), 3.66 (s, 2H), 3.61 (s, 4H), 2.68 (s, 4H). ^13^C NMR (100 MHz, DMSO-d_6_) δ 161.40, 155.00, 154.49, 148.85, 134.83, 130.18, 126.96, 126.24, 121.82, 120.55, 115.37, 106.45, 60.89, 52.91, 44.12. ESI-MS: calculated for C_20_H_20_FN_5_O_2_ [M+H]^+^ 382.16010, found 382.15906.

##### *N*-(4-fluorophenyl)-4-((4-oxo-3,4-dihydroquinazolin-2-yl)methyl)piperazine-1-carboxamide (**A3**)

White solid, yield 57.3%, m.p. 196.5–197.8 °C. ^1^H NMR (400 MHz, DMSO-d_6_) δ 11.98 (s, 1H), 8.55 (s, 1H), 8.12 (d, *J* = 7.9 Hz, 1H), 7.81 (t, *J* = 7.5 Hz, 1H), 7.66 (d, *J* = 8.0 Hz, 1H), 7.51 (t, *J* = 7.4 Hz, 1H), 7.47–7.42 (m, 2H), 7.06 (t, *J* = 8.2 Hz, 2H), 3.50 (s, 2H), 3.48 (s, 4H), 2.54 (s, 4H). ^13^C NMR (100 MHz, DMSO-d_6_) δ 162.07, 158.98, 156.62, 155.41, 154.51, 148.87, 137.27, 134.85, 127.51, 126.97, 126.26, 121.77, 115.34, 115.12, 60.94, 52.95, 44.09. ESI-MS: calculated for C_20_H_20_FN_5_O_2_ [M+H]^+^ 382.16010, found 382.15897.

##### *N*-(2-chlorophenyl)-4-((4-oxo-3,4-dihydroquinazolin-2-yl)methyl)piperazine-1-carboxamide (**A4**)

White solid, yield 45.2%, m.p. 197.9–199.6 °C. ^1^H NMR (400 MHz, CDCl_3_) δ 10.08 (s, 1H), 8.29 (d, *J* = 7.9 Hz, 1H), 8.17 (d, *J* = 8.3 Hz, 1H), 7.78 (t, *J* = 7.6 Hz, 1H), 7.68 (d, *J* = 8.1 Hz, 1H), 7.50 (t, *J* = 7.5 Hz, 1H), 7.34 (d, *J* = 8.0 Hz, 1H), 7.26 (d, *J* = 5.8 Hz, 1H), 6.98 (dd, *J* = 16.8, 9.1 Hz, 2H), 3.66 (s, 6H), 2.70 (s, 4H). ^13^C NMR (100 MHz, CDCl_3_) δ 161.81, 153.97, 152.65, 148.76, 135.51, 134.90, 128.81, 127.77, 127.13, 126.59, 123.40, 122.41, 121.70, 121.01, 60.63, 52.92, 43.98. ESI-MS: calculated for C_20_H_20_ClN_5_O_2_ [M+H]^+^ 398.13055, found 398.12985.

##### *N*-(3-chlorophenyl)-4-((4-oxo-3,4-dihydroquinazolin-2-yl)methyl)piperazine-1-carboxamide (**A5**)

White solid, yield 47.7%, m.p. 193.3–195.2 °C. ^1^H NMR (400 MHz, DMSO-d_6_) δ 12.05 (s, 1H), 8.77 (s, 1H), 8.19 (d, *J* = 7.6 Hz, 1H), 7.88 (t, *J* = 7.2 Hz, 1H), 7.72 (d, *J* = 10.2 Hz, 2H), 7.58 (t, *J* = 6.9 Hz, 1H), 7.45 (d, *J* = 8.1 Hz, 1H), 7.31 (t, *J* = 8.0 Hz, 1H), 7.04 (d, *J* = 7.6 Hz, 1H), 3.57 (s, 2H), 3.56 (s, 4H), 2.61 (s, 4H). ^13^C NMR (100 MHz, DMSO-d_6_) δ 162.08, 154.98, 154.50, 148.87, 142.64, 134.84, 133.18, 130.40, 127.51, 126.97, 126.26, 121.75, 119.16, 118.08, 60.90, 52.91, 44.13. ESI-MS: calculated for C_20_H_20_ClN_5_O_2_ [M+H]^+^ 398.13055, found 398.12985.

##### *N*-(4-chlorophenyl)-4-((4-oxo-3,4-dihydroquinazolin-2-yl)methyl)piperazine-1-carboxamide (**A6**)

White solid, yield 48.1%, m.p. 189.8–191.4 °C. ^1^H NMR (400 MHz, DMSO-d_6_) δ 11.98 (s, 1H), 8.65 (s, 1H), 8.12 (d, *J* = 7.8 Hz, 1H), 7.81 (t, *J* = 7.5 Hz, 1H), 7.66 (d, *J* = 8.1 Hz, 1H), 7.51 (t, *J* = 8.4 Hz, 3H), 7.27 (d, *J* = 8.3 Hz, 2H), 3.51 (s, 2H), 3.49 (s, 4H), 2.55 (s, 4H). ^13^C NMR (100 MHz, DMSO-d_6_) δ 162.10, 155.16, 154.51, 148.83, 140.00, 134.87, 128.62, 127.48, 126.99, 126.26, 125.72, 121.80, 121.45, 60.90, 52.92, 44.11. ESI-MS: calculated for C_20_H_20_ClN_5_O_2_ [M+H]^+^ 398.13055, found 398.13037.

##### *N*-(2-bromophenyl)-4-((4-oxo-3,4-dihydroquinazolin-2-yl)methyl)piperazine-1-carboxamide (**A7**)

White solid, yield 49.5%, m.p. 195.1–196.7 °C. ^1^H NMR (400 MHz, CDCl_3_) δ 10.07 (s, 1H), 8.29 (d, *J* = 7.9 Hz, 1H), 8.17 (d, *J* = 8.3 Hz, 1H), 7.78 (t, *J* = 7.6 Hz, 1H), 7.68 (d, *J* = 8.1 Hz, 1H), 7.50 (d, *J* = 7.4 Hz, 2H), 7.28 (d, *J* = 10.3 Hz, 1H), 7.03 (s, 1H), 6.91 (t, *J* = 7.6 Hz, 1H), 3.65 (s, 6H), 2.70 (s, 4H). ^13^C NMR (100 MHz, CDCl_3_) δ 161.90, 154.00, 152.73, 148.78, 136.60, 134.88, 132.00, 128.42, 127.13, 126.55, 123.94, 121.69, 121.27, 113.36, 60.65, 52.92, 43.99. ESI-MS: calculated for C_20_H_20_BrN_5_O_2_ [M+H]^+^ 442.08004, found 442.07788.

##### *N*-(3-bromophenyl)-4-((4-oxo-3,4-dihydroquinazolin-2-yl)methyl)piperazine-1-carboxamide (**A8**)

White solid, yield 51.2%, m.p. 194.2–196.3 °C. ^1^H NMR (400 MHz, DMSO-d_6_) δ 11.98 (s, 1H), 8.70 (s, 1H), 8.13 (d, *J* = 7.8 Hz, 1H), 7.83–7.77 (m, 2H), 7.66 (d, *J* = 8.1 Hz, 1H), 7.51 (t, *J* = 7.4 Hz, 1H), 7.45 (d, *J* = 8.1 Hz, 1H), 7.19 (t, *J* = 8.0 Hz, 1H), 7.10 (d, *J* = 7.8 Hz, 1H), 3.51 (s, 2H), 3.50 (s, 4H), 2.56 (s, 4H). ^13^C NMR (100 MHz, DMSO-d_6_) δ 162.09, 154.98, 154.50, 148.85, 142.76, 134.85, 130.72, 127.48, 126.97, 126.26, 124.58, 122.05, 121.77, 118.49, 60.89, 52.90, 44.12. ESI-MS: calculated for C_20_H_20_BrN_5_O_2_ [M+H]^+^ 442.08004, found 442.07776.

##### *N*-(4-bromophenyl)-4-((4-oxo-3,4-dihydroquinazolin-2-yl)methyl)piperazine-1-carboxamide (**A9**)

White solid, yield 53.3%, m.p. 187.8–188.9 °C. ^1^H NMR (400 MHz, DMSO-d_6_) δ 11.98 (s, 1H), 8.65 (s, 1H), 8.12 (d, *J* = 7.7 Hz, 1H), 7.81 (t, *J* = 7.6 Hz, 1H), 7.66 (d, *J* = 8.3 Hz, 1H), 7.51 (t, *J* = 7.7 Hz, 1H), 7.42 (dd, *J* = 18.6, 8.1 Hz, 4H), 3.50 (s, 2H), 3.48 (s, 4H), 2.54 (s, 4H). ^13^C NMR (100 MHz, DMSO-d_6_) δ 162.07, 155.09, 154.51, 148.87, 140.49, 134.83, 131.51, 127.50, 126.96, 126.26, 121.83, 113.61, 60.91, 52.93, 44.13. ESI-MS: calculated for C_20_H_20_BrN_5_O_2_ [M+H]^+^ 442.08004, found 442.07852.

##### 4-((4-oxo-3,4-dihydroquinazolin-2-yl)methyl)-*N*-(o-tolyl)piperazine-1-carboxamide (**A10**)

White solid, yield 52.1%, m.p. 199.9–201.6 °C. ^1^H NMR (400 MHz, CDCl_3_) δ 9.97 (s, 1H), 8.29 (d, *J* = 7.9 Hz, 1H), 7.78 (t, *J* = 7.6 Hz, 1H), 7.68 (d, *J* = 8.1 Hz, 1H), 7.56 (d, *J* = 7.9 Hz, 1H), 7.50 (t, *J* = 7.5 Hz, 1H), 7.18 (dd, *J* = 13.4, 7.1 Hz, 2H), 7.03 (t, *J* = 7.4 Hz, 1H), 6.17 (s, 1H), 3.63 (s, 2H), 3.58 (s, 4H), 2.66 (s, 4H), 2.25 (s, 3H). ^13^C NMR (100 MHz, CDCl_3_) δ 161.78, 155.37, 152.75, 148.78, 136.73, 134.89, 130.48, 129.55, 127.11, 126.68, 124.54, 123.32, 121.70, 60.62, 52.96, 44.13, 17.88. ESI-MS: calculated for C_21_H_23_N_5_O_2_ [M+H]^+^ 378.18518, found 378.18420.

##### 4-((4-oxo-3,4-dihydroquinazolin-2-yl)methyl)-*N*-(m-tolyl)piperazine-1-carboxamide (**A11**)

White solid, yield 48.8%, m.p. 197.8–200.2 °C. ^1^H NMR (400 MHz, CDCl_3_) δ 9.97 (s, 1H), 8.29 (d, *J* = 7.9 Hz, 1H), 7.78 (t, *J* = 7.6 Hz, 1H), 7.67 (d, *J* = 8.1 Hz, 1H), 7.50 (t, *J* = 7.5 Hz, 1H), 7.22 (s, 1H), 7.17 (t, *J* = 7.6 Hz, 1H), 7.11 (d, *J* = 8.0 Hz, 1H), 6.87 (d, *J* = 7.3 Hz, 1H), 6.41 (s, 1H), 3.63 (s, 2H), 3.58 (s, 4H), 2.65 (s, 4H), 2.32 (s, 3H). ^13^C NMR (100 MHz, CDCl_3_) δ 161.70, 154.95, 152.66, 148.79, 138.85, 138.64, 134.89, 131.83, 128.76, 127.04, 126.61, 124.22, 121.73, 120.85, 117.14, 60.60, 52.99, 44.02, 21.50. ESI-MS: calculated for C_21_H_23_N_5_O_2_ [M+H]^+^ 378.18518, found 378.18457.

##### 4-((4-oxo-3,4-dihydroquinazolin-2-yl)methyl)-*N*-(p-tolyl)piperazine-1-carboxamide (**A12**)

White solid, yield 49.6%, m.p. 197.7–199.5 °C. ^1^H NMR (400 MHz, CDCl_3_) δ 9.88 (s, 1H), 8.29 (d, *J* = 7.8 Hz, 1H), 7.78 (t, *J* = 7.7 Hz, 1H), 7.67 (d, *J* = 8.0 Hz, 1H), 7.50 (t, *J* = 7.4 Hz, 1H), 7.23 (d, *J* = 7.6 Hz, 2H), 7.10 (d, *J* = 7.8 Hz, 2H), 6.28 (s, 1H), 3.64 (s, 2H), 3.58 (s, 4H), 2.66 (s, 4H), 2.30 (s, 3H). ^13^C NMR (100 MHz, DMSO-d_6_) δ 162.07, 155.47, 154.52, 148.87, 138.36, 134.84, 130.93, 129.17, 127.50, 126.96, 126.26, 121.83, 120.23, 60.96, 52.99, 44.10, 20.81. ESI-MS: calculated for C_21_H_23_N_5_O_2_ [M+H]^+^ 378.18518, found 378.18423.

##### 4-((4-oxo-3,4-dihydroquinazolin-2-yl)methyl)-*N*-(2-(trifluoromethyl)phenyl) piperazine-1-carboxamide (**A13**)

White solid, yield 47.9%, m.p. 200.1–201.2 °C. ^1^H NMR (400 MHz, DMSO-d_6_) δ 11.99 (s, 1H), 8.23 (s, 1H), 8.12 (d, *J* = 8.0 Hz, 1H), 7.81 (t, *J* = 7.4 Hz, 1H), 7.70–7.61 (m, 3H), 7.51 (t, *J* = 7.4 Hz, 1H), 7.40 (t, *J* = 8.6 Hz, 2H), 3.50 (s, 2H), 3.46 (s, 4H), 2.53 (s, 4H). ^13^C NMR (100 MHz, DMSO-d_6_) δ 162.08, 156.27, 154.49, 148.88, 137.87, 134.82, 133.13, 131.03, 127.51, 126.95, 126.66, 126.32, 125.95, 122.96, 121.84, 60.97, 52.85, 44.29. ESI-MS: calculated for C_21_H_20_F_3_N_5_O_2_ [M+H]^+^ 432.15691, found 432.15488.

##### 4-((4-oxo-3,4-dihydroquinazolin-2-yl)methyl)-*N*-(3-(trifluoromethyl)phenyl)piperazine-1-carboxamide (**A14**)

White solid, yield 46.8%, m.p. 197.8–200.3 °C. ^1^H NMR (400 MHz, DMSO-d_6_) δ 11.99 (s, 1H), 8.86 (s, 1H), 8.12 (d, *J* = 7.9 Hz, 1H), 7.92 (s, 1H), 7.81 (t, *J* = 7.4 Hz, 1H), 7.73 (d, *J* = 8.1 Hz, 1H), 7.66 (d, *J* = 7.9 Hz, 1H), 7.49 (dt, *J* = 15.5, 7.4 Hz, 2H), 7.26 (d, *J* = 7.5 Hz, 1H), 3.51 (s, 6H), 2.56 (s, 4H). ^13^C NMR (100 MHz, DMSO-d_6_) δ 162.07, 155.03, 154.50, 148.89, 141.93, 134.82, 129.82, 129.42, 127.50, 126.95, 126.26, 123.21, 121.85, 118.24, 115.77, 60.89, 52.90, 44.12. ESI-MS: calculated for C_21_H_20_F_3_N_5_O_2_ [M+H]^+^ 432.15691, found 432.15555.

##### 4-((4-oxo-3,4-dihydroquinazolin-2-yl)methyl)-*N*-(4-(trifluoromethyl)phenyl)piperazine-1-carboxamide (**A15**)

White solid, yield 55.4%, m.p. 198.8–200.6 °C. ^1^H NMR (400 MHz, DMSO-d_6_) δ 12.06 (s, 1H), 8.98 (s, 1H), 8.19 (d, *J* = 7.9 Hz, 1H), 7.88 (t, *J* = 7.5 Hz, 1H), 7.74 (t, *J* = 7.4 Hz, 3H), 7.65 (d, *J* = 8.3 Hz, 2H), 7.58 (t, *J* = 7.5 Hz, 1H), 3.58 (s, 6H), 2.62 (s, 4H). ^13^C NMR (100 MHz, DMSO-d_6_) δ 162.07, 154.89, 154.49, 148.87, 144.90, 134.84, 127.24, 126.78, 126.21, 123.76, 122.12, 121.84, 119.32, 60.89, 52.91, 44.18. ESI-MS: calculated for C_21_H_20_F_3_N_5_O_2_ [M+H]^+^ 432.15691, found 432.15540.

##### *N*-(2-methoxyphenyl)-4-((4-oxo-3,4-dihydroquinazolin-2-yl)methyl)piperazine-1-carboxamide (**A16**)

White solid, yield 56.8%, m.p. 188.5–190.7 °C. ^1^H NMR (400 MHz, CDCl_3_) δ 10.06 (s, 1H), 8.29 (d, *J* = 7.9 Hz, 1H), 8.17–8.08 (m, 1H), 7.77 (t, *J* = 7.5 Hz, 1H), 7.68 (d, *J* = 8.1 Hz, 1H), 7.50 (t, *J* = 7.5 Hz, 1H), 7.11 (s, 1H), 6.96 (dt, *J* = 9.8, 5.0 Hz, 2H), 6.86 (d, *J* = 6.9 Hz, 1H), 3.87 (s, 3H), 3.64 (s, 2H), 3.61 (s, 4H), 2.67 (s, 4H). ^13^C NMR (100 MHz, CDCl_3_) δ 161.81, 154.50, 152.82, 148.80, 147.64, 134.86, 128.50, 127.10, 126.58, 122.31, 121.70, 121.22, 119.11, 109.76, 60.64, 55.76, 52.99, 43.88. ESI-MS: calculated for C_21_H_23_N_5_O_3_ [M+H]^+^ 394.18009, found 394.17911.

##### *N*-(3-methoxyphenyl)-4-((4-oxo-3,4-dihydroquinazolin-2-yl)methyl)piperazine-1-carboxamide (**A17**)

White solid, yield 53.7%, m.p. 186.3–188.5 °C. ^1^H NMR (400 MHz, CDCl_3_) δ 10.04 (s, 1H), 8.28 (d, *J* = 7.9 Hz, 1H), 7.78 (t, *J* = 7.6 Hz, 1H), 7.67 (d, *J* = 8.2 Hz, 1H), 7.50 (t, *J* = 7.5 Hz, 1H), 7.15 (dd, *J* = 18.2, 10.1 Hz, 2H), 6.84 (d, *J* = 8.0 Hz, 1H), 6.61 (t, *J* = 10.5 Hz, 2H), 3.78 (s, 3H), 3.62 (s, 2H), 3.58 (s, 4H), 2.63 (s, 4H). ^13^C NMR (100 MHz, CDCl_3_) δ 161.95, 160.11, 153.68, 152.87, 148.79, 140.33, 134.89, 130.47, 126.50, 124.65, 123.63, 121.64, 112.23, 108.96, 105.82, 60.61, 55.25, 52.94, 43.94. ESI-MS: calculated for C_21_H_23_N_5_O_3_ [M+H]^+^ 394.18009, found 394.17899.

##### *N*-(4-methoxyphenyl)-4-((4-oxo-3,4-dihydroquinazolin-2-yl)methyl)piperazine-1-carboxamide (**A18**)

White solid, yield 56.7%, m.p. 184.9–186.9 °C. ^1^H NMR (400 MHz, CDCl_3_) δ 10.05 (s, 1H), 8.28 (d, *J* = 7.9 Hz, 1H), 7.77 (t, *J* = 7.6 Hz, 1H), 7.67 (d, *J* = 8.1 Hz, 1H), 7.49 (t, *J* = 7.4 Hz, 1H), 7.24 (d, *J* = 7.9 Hz, 2H), 6.83 (d, *J* = 8.0 Hz, 2H), 6.46 (s, 1H), 3.77 (s, 3H), 3.62 (s, 2H), 3.56 (s, 4H), 2.62 (s, 4H). ^13^C NMR (100 MHz, CDCl_3_) δ 161.72, 156.09, 155.44, 152.72, 148.80, 134.88, 131.71, 127.11, 126.60, 122.61, 121.72, 114.17, 60.61, 55.52, 52.99, 43.97. ESI-MS: calculated for C_21_H_23_N_5_O_3_ [M+H]^+^ 394.18009, found 394.17880.

##### *N*-(2,3-difluorophenyl)-4-((4-oxo-3,4-dihydroquinazolin-2-yl)methyl)piperazine-1-carboxamide (**A19**)

White solid, yield 57.8%, m.p. 188.2–189.9 °C. ^1^H NMR (400 MHz, CDCl_3_) δ 10.07 (s, 1H), 8.28 (d, *J* = 7.9 Hz, 1H), 7.89–7.75 (m, 2H), 7.68 (d, *J* = 8.1 Hz, 1H), 7.50 (t, *J* = 7.4 Hz, 1H), 6.92 (ddd, *J* = 30.8, 15.7, 7.6 Hz, 2H), 6.58 (d, *J* = 54.3 Hz, 1H), 3.65 (s, 2H), 3.62 (s, 4H), 2.68 (d, *J* = 4.3 Hz, 4H). ^13^C NMR (100 MHz, CDCl_3_) δ 161.73, 154.34, 153.79, 152.59, 148.78, 134.90, 127.14, 126.60, 125.00, 121.73, 116.55, 115.34, 110.64, 60.61, 52.90, 44.05. ESI-MS: calculated for C_20_H_19_F_2_N_5_O_2_ [M+H]^+^ 400.15068, found 400.14935.

##### *N*-(2,5-difluorophenyl)-4-((4-oxo-3,4-dihydroquinazolin-2-yl)methyl)piperazine-1-carboxamide (**A20**)

White solid, yield 49.7%, m.p. 202.1–204.2 °C. ^1^H NMR (400 MHz, DMSO-d_6_) δ 11.99 (s, 1H), 8.43 (s, 1H), 8.12 (d, *J* = 7.7 Hz, 1H), 7.81 (t, *J* = 7.7 Hz, 1H), 7.66 (d, *J* = 8.2 Hz, 1H), 7.51 (t, *J* = 7.1 Hz, 1H), 7.41 (s, 1H), 7.23 (d, *J* = 4.7 Hz, 1H), 6.91 (s, 1H), 3.51 (s, 2H), 3.48 (s, 4H), 2.55 (s, 4H). ^13^C NMR (100 MHz, DMSO-d_6_) δ 162.08, 159.26, 156.90, 154.95, 152.39, 148.87, 134.82, 129.57, 127.50, 126.95, 126.25, 121.84, 116.77, 111.88, 110.66, 60.91, 52.86, 44.27. ESI-MS: calculated for C_20_H_19_F_2_N_5_O_2_ [M+H]^+^ 400.15068, found 400.14932.

##### *N*-(2-fluoro-3-methylphenyl)-4-((4-oxo-3,4-dihydroquinazolin-2-yl)methyl)piperazine-1-carboxamide (**A21**)

White solid, yield 52.9%, m.p. 200.5–202.1 °C. ^1^H NMR (400 MHz, DMSO-d_6_) δ 11.99 (s, 1H), 8.22 (s, 1H), 8.12 (d, *J* = 7.9 Hz, 1H), 7.81 (t, *J* = 7.5 Hz, 1H), 7.66 (d, *J* = 8.0 Hz, 1H), 7.51 (t, *J* = 7.3 Hz, 1H), 7.23 (s, 1H), 6.98 (d, *J* = 4.6 Hz, 2H), 3.51 (s, 2H), 3.47 (s, 4H), 2.54 (s, 4H), 2.22 (s, 3H). ^13^C NMR (100 MHz, DMSO-d_6_) δ 162.07, 155.49, 153.14, 148.88, 134.85, 127.85, 127.63, 126.90, 126.26, 124.73, 124.07, 123.71, 121.84, 60.96, 52.91, 44.21, 14.71. ESI-MS: calculated for C_21_H_22_FN_5_O_2_ [M+H]^+^ 396.17575, found 396.17487.

##### *N*-(2-fluoro-4-methylphenyl)-4-((4-oxo-3,4-dihydroquinazolin-2-yl)methyl)piperazine-1-carboxamide (**A22**)

White solid, yield 50.8%, m.p. 198.4–200.7 °C. ^1^H NMR (400 MHz, DMSO-d_6_) δ 11.99 (s, 1H), 8.19 (s, 1H), 8.12 (d, *J* = 7.8 Hz, 1H), 7.81 (t, *J* = 7.4 Hz, 1H), 7.66 (d, *J* = 8.1 Hz, 1H), 7.51 (t, *J* = 7.4 Hz, 1H), 7.25 (t, *J* = 8.2 Hz, 1H), 7.00 (d, *J* = 11.6 Hz, 1H), 6.91 (d, *J* = 7.8 Hz, 1H), 3.50 (s, 2H), 3.46 (s, 4H), 2.53 (s, 4H), 2.27 (s, 3H). ^13^C NMR (100 MHz, DMSO-d_6_) δ 162.07, 155.59, 154.49, 148.86, 135.55, 134.85, 127.51, 126.93, 126.26, 124.97, 121.83, 116.27, 60.96, 52.92, 44.15, 20.80. ESI-MS: calculated for C_21_H_22_FN_5_O_2_ [M+H]^+^ 396.17575, found 396.17462.

##### *N*-(2-fluoro-5-methylphenyl)-4-((4-oxo-3,4-dihydroquinazolin-2-yl)methyl)piperazine-1-carboxamide (**A23**)

White solid, yield 47.8%, m.p. 199.5–201.1 °C. ^1^H NMR (400 MHz, CDCl_3_) δ 9.98 (s, 1H), 8.29 (d, *J* = 7.9 Hz, 1H), 7.88 (d, *J* = 7.7 Hz, 1H), 7.78 (t, *J* = 7.6 Hz, 1H), 7.68 (d, *J* = 8.1 Hz, 1H), 7.50 (t, *J* = 7.5 Hz, 1H), 6.93 (t, *J* = 9.6 Hz, 1H), 6.80–6.72 (m, 1H), 6.55 (s, 1H), 3.65 (s, 2H), 3.62 (s, 4H), 2.68 (s, 4H), 2.31 (s, 3H). ^13^C NMR (100 MHz, CDCl_3_) δ 161.92, 154.26, 152.75, 148.77, 134.88, 134.19, 127.12, 126.61, 123.62, 122.11, 121.67, 114.21, 60.64, 52.91, 43.98, 21.13. ESI-MS: calculated for C_21_H_22_FN_5_O_2_ [M+H]^+^ 396.17575, found 396.17477.

##### *N*-(2-fluoro-4-(trifluoromethyl)phenyl)-4-((4-oxo-3,4-dihydroquinazolin-2-yl)methyl)piperazine-1-carboxamide (**A24**)

White solid, yield 49.5%, m.p. 202.3–203.9 °C. ^1^H NMR (400 MHz, CDCl_3_) δ 10.01 (s, 1H), 8.29 (t, *J* = 7.4 Hz, 2H), 7.78 (t, *J* = 7.6 Hz, 1H), 7.68 (d, *J* = 8.1 Hz, 1H), 7.51 (t, *J* = 7.5 Hz, 1H), 7.40 (d, *J* = 8.7 Hz, 1H), 7.33 (d, *J* = 11.2 Hz, 1H), 6.77 (s, 1H), 3.66 (s, 2H), 3.64 (s, 4H), 2.70 (s, 4H). ^13^C NMR (100 MHz, CDCl_3_) δ 161.77, 153.40, 152.54, 150.20, 148.74, 134.94, 130.71, 127.17, 126.60, 121.95, 121.70, 120.83, 112.22, 60.60, 52.87, 44.02. ESI-MS: calculated for C_21_H_19_F_4_N_5_O_2_ [M+H]^+^ 450.14749, found 450.14508.

##### *N*-(2-chloro-6-fluorophenyl)-4-((4-oxo-3,4-dihydroquinazolin-2-yl)methyl)piperazine-1-carboxamide (**A25**)

White solid, yield 53.2%, m.p. 198.4–199.8 °C. ^1^H NMR (400 MHz, CDCl_3_) δ 10.07 (s, 1H), 8.28 (d, *J* = 7.8 Hz, 1H), 7.78 (t, *J* = 7.5 Hz, 1H), 7.68 (d, *J* = 8.1 Hz, 1H), 7.50 (t, *J* = 7.4 Hz, 1H), 7.20 (d, *J* = 7.9 Hz, 1H), 7.14–7.01 (m, 2H), 6.18 (s, 1H), 3.64 (s, 6H), 2.67 (s, 4H). ^13^C NMR (100 MHz, CDCl_3_) δ 161.87, 159.10, 156.61, 154.54, 152.81, 148.78, 134.89, 131.07, 127.11, 126.55, 124.96, 121.66, 114.95, 114.75, 60.63, 52.91, 44.29. ESI-MS: calculated for C_20_H_19_ClFN_5_O_2_ [M+H]^+^ 416.12113, found 416.11945.

##### *N*-(2-chloro-5-(trifluoromethyl)phenyl)-4-((4-oxo-3,4-dihydroquinazolin-2-yl)methyl)piperazine-1-carboxamide (**A26**)

White solid, yield 54.1%, m.p. 191.2–193.9 °C. ^1^H NMR (400 MHz, CDCl_3_) δ 10.15 (s, 1H), 8.58 (s, 1H), 8.28 (d, *J* = 7.9 Hz, 1H), 7.78 (t, *J* = 7.6 Hz, 1H), 7.68 (d, *J* = 8.1 Hz, 1H), 7.53–7.43 (m, 2H), 7.22 (d, *J* = 8.3 Hz, 1H), 7.12 (s, 1H), 3.67 (s, 6H), 2.72 (s, 4H). ^13^C NMR (100 MHz, CDCl_3_) δ 161.90, 153.49, 152.58, 148.75, 136.18, 134.92, 129.21, 127.16, 126.54, 125.32, 124.96, 122.25, 121.67, 119.70, 117.68, 60.63, 52.85, 43.99. ESI-MS: calculated for C_21_H_19_ClF_3_N_5_O_2_ [M+H]^+^ 466.11794, found 466.11606.

##### *N*-(2-chloro-5-methylphenyl)-4-((4-oxo-3,4-dihydroquinazolin-2-yl)methyl)piperazine-1-carboxamide (**A27**)

White solid, yield 52.1%, m.p. 196.7–198.7 °C. ^1^H NMR (400 MHz, CDCl_3_) δ 10.11 (s, 1H), 8.28 (d, *J* = 7.9 Hz, 1H), 8.00 (s, 1H), 7.78 (t, *J* = 7.6 Hz, 1H), 7.68 (d, *J* = 8.1 Hz, 1H), 7.50 (t, *J* = 7.4 Hz, 1H), 7.20 (d, *J* = 8.1 Hz, 1H), 6.96 (s, 1H), 6.78 (d, *J* = 8.1 Hz, 1H), 3.65 (d, *J* = 4.9 Hz, 6H), 2.70 (s, 4H), 2.32 (s, 3H). ^13^C NMR (100 MHz, CDCl_3_) δ 161.81, 154.04, 152.66, 148.76, 137.91, 134.96, 128.36, 127.13, 126.58, 124.24, 121.61, 119.42, 60.63, 52.92, 43.98, 21.39. ESI-MS: calculated for C_21_H_22_ClN_5_O_2_ [M+H]^+^ 412.14620, found 412.14481.

##### *N*-(3-fluoro-2-methylphenyl)-4-((4-oxo-3,4-dihydroquinazolin-2-yl)methyl)piperazine-1-carboxamide (**A28**)

White solid, yield 51.8%, m.p. 198.9–200.6 °C. ^1^H NMR (400 MHz, DMSO-d_6_) δ 11.99 (s, 1H), 8.26 (s, 1H), 8.12 (d, *J* = 7.9 Hz, 1H), 7.81 (t, *J* = 7.4 Hz, 1H), 7.66 (d, *J* = 8.1 Hz, 1H), 7.52 (t, *J* = 7.5 Hz, 1H), 7.14 (dd, *J* = 14.5, 7.4 Hz, 1H), 7.02 (d, *J* = 7.8 Hz, 1H), 6.94 (t, *J* = 8.8 Hz, 1H), 3.49 (d, *J* = 11.6 Hz, 6H), 2.55 (s, 4H), 2.03 (s, 3H). ^13^C NMR (100 MHz, DMSO-d_6_) δ 162.07, 160.01, 155.77, 154.51, 148.87, 140.56, 134.85, 127.51, 126.97, 126.64, 126.26, 122.07, 121.83, 120.84, 111.25, 60.97, 52.95, 44.31, 10.39. ESI-MS: calculated for C_21_H_22_FN_5_O_2_ [M+H]^+^ 396.17575, found 396.17432.

##### *N*-(4-fluoro-2-methylphenyl)-4-((4-oxo-3,4-dihydroquinazolin-2-yl)methyl)piperazine-1-carboxamide (**A29**)

White solid, yield 52.7%, m.p. 197.6–200.1 °C. ^1^H NMR (400 MHz, DMSO-d_6_) δ 11.99 (s, 1H), 8.12 (d, *J* = 7.8 Hz, 1H), 8.06 (s, 1H), 7.81 (t, *J* = 7.5 Hz, 1H), 7.66 (d, *J* = 8.1 Hz, 1H), 7.52 (t, *J* = 7.5 Hz, 1H), 7.18–7.11 (m, 1H), 7.04 (d, *J* = 9.8 Hz, 1H), 6.94 (t, *J* = 8.5 Hz, 1H), 3.51 (s, 2H), 3.46 (s, 4H), 2.54 (s, 4H), 2.14 (s, 3H). ^13^C NMR (100 MHz, DMSO-d_6_) δ 162.08, 158.52, 156.07, 154.51, 148.87, 136.67, 134.84, 128.51, 127.51, 126.97, 126.26, 121.83, 116.82, 112.77, 60.98, 52.95, 44.26, 18.42. ESI-MS: calculated for C_21_H_22_FN_5_O_2_ [M+H]^+^ 396.17575, found 396.17441.

##### *N*-(5-fluoro-2-methylphenyl)-4-((4-oxo-3,4-dihydroquinazolin-2-yl)methyl)piperazine-1-carboxamide (**A30**)

White solid, yield 47.8%, m.p. 201.1–202.8 °C. ^1^H NMR (400 MHz, CDCl_3_) δ 9.99 (s, 1H), 8.29 (d, *J* = 7.9 Hz, 1H), 7.78 (t, *J* = 7.6 Hz, 1H), 7.68 (d, *J* = 8.1 Hz, 1H), 7.57–7.47 (m, 2H), 7.08 (t, *J* = 7.2 Hz, 1H), 6.71 (t, *J* = 8.2 Hz, 1H), 6.24 (s, 1H), 3.64 (s, 2H), 3.60 (s, 4H), 2.67 (s, 4H), 2.20 (s, 3H). ^13^C NMR (100 MHz, CDCl_3_) δ 162.71, 161.74, 160.30, 154.57, 152.61, 148.77, 137.96, 134.92, 131.03, 127.21, 126.60, 122.92, 121.70, 110.46, 109.24, 60.61, 52.92, 44.09, 17.15. ESI-MS: calculated for C_21_H_22_FN_5_O_2_ [M+H]^+^ 396.17575, found 396.17477.

##### *N*-(3-bromo-2-methylphenyl)-4-((4-oxo-3,4-dihydroquinazolin-2-yl)methyl)piperazine-1-carboxamide (**A31**)

White solid, yield 53.6%, m.p. 200.6–202.2 °C. ^1^H NMR (400 MHz, DMSO-d_6_) δ 11.99 (s, 1H), 8.34 (s, 1H), 8.12 (d, *J* = 7.8 Hz, 1H), 7.81 (t, *J* = 7.1 Hz, 1H), 7.66 (d, *J* = 8.2 Hz, 1H), 7.52 (t, *J* = 7.2 Hz, 1H), 7.40 (d, *J* = 8.0 Hz, 1H), 7.16 (d, *J* = 7.8 Hz, 1H), 7.08 (t, *J* = 8.0 Hz, 1H), 3.51 (s, 2H), 3.48 (s, 4H), 2.55 (s, 4H), 2.19 (s, 3H). ^13^C NMR (100 MHz, DMSO-d_6_) δ 162.08, 155.87, 154.50, 140.13, 134.85, 133.87, 129.16, 127.50, 126.97, 126.28, 125.00, 121.83, 60.96, 52.96, 44.27, 18.90. ESI-MS: calculated for C_21_H_22_BrN_5_O_2_ [M+H]^+^ 456.09569, found 456.09409.

##### *N*-(5-chloro-2-methylphenyl)-4-((4-oxo-3,4-dihydroquinazolin-2-yl)methyl)piperazine-1-carboxamide (**A32**)

White solid, yield 58.3%, m.p. 203.1–204.7 °C. ^1^H NMR (400 MHz, DMSO-d_6_) δ 11.99 (s, 1H), 8.12 (d, *J* = 8.7 Hz, 2H), 7.81 (t, *J* = 7.3 Hz, 1H), 7.66 (d, *J* = 7.9 Hz, 1H), 7.52 (t, *J* = 7.5 Hz, 1H), 7.32 (s, 1H), 7.19 (d, *J* = 8.7 Hz, 1H), 7.08 (d, *J* = 8.0 Hz, 1H), 3.51 (s, 2H), 3.48 (s, 4H), 2.55 (s, 4H), 2.14 (s, 3H). ^13^C NMR (100 MHz, DMSO-d_6_) δ 162.08, 155.55, 154.49, 148.88, 139.94, 134.84, 131.85, 130.05, 126.97, 126.26, 125.22, 124.34, 121.83, 120.54, 60.93, 52.90, 44.30, 17.89. ESI-MS: calculated for C_21_H_22_ClN_5_O_2_ [M+H]^+^ 412.14620, found 412.14517.

##### *N*-(2,6-dimethylphenyl)-4-((4-oxo-3,4-dihydroquinazolin-2-yl)methyl)piperazine-1-carboxamide (**A33**)

White solid, yield 55.7%, m.p. 200.3–201.9 °C. ^1^H NMR (400 MHz, DMSO-d_6_) δ 11.99 (s, 1H), 8.12 (d, *J* = 7.8 Hz, 1H), 7.86 (s, 1H), 7.81 (t, *J* = 7.7 Hz, 1H), 7.66 (d, *J* = 8.1 Hz, 1H), 7.52 (t, *J* = 7.3 Hz, 1H), 7.03 (s, 3H), 3.51 (s, 2H), 3.48 (s, 4H), 2.54 (s, 4H), 2.12 (s, 6H). ^13^C NMR (100 MHz, DMSO-d_6_) δ 162.07, 156.03, 154.50, 148.87, 137.02, 136.35, 134.84, 127.98, 127.51, 126.96, 126.25, 121.82, 61.03, 53.01, 44.45, 18.62. ESI-MS: calculated for C_22_H_25_N_5_O_2_ [M+H]^+^ 392.20083, found 392.19940.

##### *N*-(3,5-difluorophenyl)-4-((4-oxo-3,4-dihydroquinazolin-2-yl)methyl)piperazine-1-carboxamide (**A34**)

White solid, yield 56.1%, m.p.199.8–200.2 °C. ^1^H NMR (400 MHz, DMSO-d_6_) δ 11.98 (s, 1H), 8.90 (s, 1H), 8.13 (d, *J* = 7.9 Hz, 1H), 7.81 (t, *J* = 7.6 Hz, 1H), 7.66 (d, *J* = 8.1 Hz, 1H), 7.51 (t, *J* = 7.5 Hz, 1H), 7.25 (d, *J* = 9.8 Hz, 2H), 6.73 (t, *J* = 9.2 Hz, 1H), 3.51 (d, *J* = 5.3 Hz, 6H), 2.56 (s, 4H). ^13^C NMR (100 MHz, DMSO-d_6_) δ 164.07, 162.09, 161.67, 154.57, 148.85, 143.88, 134.85, 127.48, 126.97, 126.25, 121.81, 102.26, 101.97, 96.88, 60.84, 52.84, 44.10. ESI-MS: calculated for C_20_H_19_F_2_N_5_O_2_ [M+H]^+^ 400.15068, found 400.14957.

##### *N*-(3,5-dichlorophenyl)-4-((4-oxo-3,4-dihydroquinazolin-2-yl)methyl)piperazine-1-carboxamide (**A35**)

White solid, yield 55.8%, m.p. 207.5–209.5 °C. ^1^H NMR (400 MHz, DMSO-d_6_) δ 11.98 (s, 1H), 8.86 (s, 1H), 8.12 (d, *J* = 8.0 Hz, 1H), 7.81 (t, *J* = 7.4 Hz, 1H), 7.66 (d, *J* = 8.0 Hz, 1H), 7.59 (s, 2H), 7.51 (t, *J* = 7.6 Hz, 1H), 7.12 (s, 1H), 3.51 (s, 2H), 3.49 (s, 4H), 2.55 (s, 4H). ^13^C NMR (100 MHz, DMSO-d_6_) δ 162.07, 154.58, 148.85, 143.65, 134.84, 134.12, 127.51, 126.97, 126.26, 121.84, 121.02, 117.56, 60.82, 52.83, 44.10. ESI-MS: calculated for C_20_H_19_Cl_2_N_5_O_2_ [M+H]^+^ 432.09158, found 432.09000.

##### *N*-(3-chloro-4-fluorophenyl)-4-((4-oxo-3,4-dihydroquinazolin-2-yl)methyl)piperazine-1-carboxamide (**A36**)

White solid, yield 57.6%, m.p. 200.1–202.7 °C. ^1^H NMR (400 MHz, DMSO-d_6_) δ 11.98 (s, 1H), 8.70 (s, 1H), 8.12 (d, *J* = 7.7 Hz, 1H), 7.81 (t, *J* = 7.7 Hz, 1H), 7.74 (d, *J* = 6.8 Hz, 1H), 7.66 (d, *J* = 7.9 Hz, 1H), 7.51 (t, *J* = 7.4 Hz, 1H), 7.39 (s, 1H), 7.29 (t, *J* = 9.1 Hz, 1H), 3.51 (s, 2H), 3.48 (s, 4H), 2.54 (s, 4H). ^13^C NMR (100 MHz, DMSO-d_6_) δ 162.10, 155.06, 154.51, 151.53, 148.83, 138.32, 134.87, 127.48, 127.00, 126.26, 121.80, 121.14, 120.01, 119.01, 116.97, 60.87, 52.87, 44.07. ESI-MS: calculated for C_20_H_19_ClFN_5_O_2_ [M+H]^+^ 416.12113, found 416.11984.

##### *N*-(3,4-dimethylphenyl)-4-((4-oxo-3,4-dihydroquinazolin-2-yl)methyl)piperazine-1-carboxamide (**A37**)

White solid, yield 58.9%, m.p. 194.4–196.1 °C. ^1^H NMR (400 MHz, DMSO-d_6_) δ 11.98 (s, 1H), 8.34 (s, 1H), 8.12 (d, *J* = 7.8 Hz, 1H), 7.81 (t, *J* = 7.4 Hz, 1H), 7.66 (d, *J* = 8.0 Hz, 1H), 7.51 (t, *J* = 7.3 Hz, 1H), 7.21 (s, 1H), 7.15 (d, *J* = 8.1 Hz, 1H), 6.97 (d, *J* = 8.1 Hz, 1H), 3.50 (s, 2H), 3.46 (s, 4H), 2.54 (s, 4H), 2.15 (d, *J* = 7.9 Hz, 6H). ^13^C NMR (100 MHz, DMSO-d_6_) δ 162.07, 155.48, 154.52, 148.87, 138.57, 136.15, 134.84, 129.71, 127.50, 126.96, 126.26, 121.83, 121.61, 117.73, 60.95, 52.99, 44.11, 20.10, 19.14. ESI-MS: calculated for C_22_H_25_N_5_O_2_ [M+H]^+^ 392.20083, found 392.19974.

##### *N*-(3,5-dimethylphenyl)-4-((4-oxo-3,4-dihydroquinazolin-2-yl)methyl)piperazine-1-carboxamide (**A38**)

White solid, yield 56.3%, m.p. 201.5–203.3 °C. ^1^H NMR (400 MHz, DMSO-d_6_) δ 11.97 (s, 1H), 8.35 (s, 1H), 8.11 (d, *J* = 7.7 Hz, 1H), 7.80 (t, *J* = 7.4 Hz, 1H), 7.65 (d, *J* = 8.1 Hz, 1H), 7.51 (t, *J* = 7.4 Hz, 1H), 7.06 (s, 3H), 3.49 (s, 2H), 3.46 (s, 4H), 2.53 (s, 4H), 2.19 (s, 6H). ^13^C NMR (100 MHz, DMSO-d_6_) δ 162.07, 155.38, 154.52, 148.87, 140.76, 138.17, 137.55, 134.84, 127.50, 126.97, 126.26, 123.75, 121.83, 117.86, 116.29, 60.93, 52.97, 44.15, 21.59. ESI-MS: calculated for C_22_H_25_N_5_O_2_ [M+H]^+^ 392.20083, found 392.19919.

##### *N*-(3,5-bis(trifluoromethyl)phenyl)-4-((4-oxo-3,4-dihydroquinazolin-2-yl)methyl)piperazine-1-carboxamide (**A39**)

White solid, yield 54.5%, m.p. 204.2–206.1 °C. ^1^H NMR (400 MHz, DMSO-d_6_) δ 11.99 (s, 1H), 9.19 (s, 1H), 8.20 (s, 2H), 8.12 (d, *J* = 8.0 Hz, 1H), 7.81 (t, *J* = 7.4 Hz, 1H), 7.65 (d, *J* = 7.6 Hz, 1H), 7.59 (s, 1H), 7.51 (t, *J* = 7.6 Hz, 1H), 3.51 (s, 6H), 2.56 (s, 4H). ^13^C NMR (100 MHz, DMSO-d_6_) δ 162.07, 154.53, 148.88, 143.19, 134.83, 130.96, 130.64, 130.32, 127.50, 126.96, 126.25, 125.24, 122.53, 121.84, 119.11, 114.52, 60.84, 52.80, 44.09. ESI-MS: calculated for C_22_H_19_F_6_N_5_O_2_ [M+H]^+^ 500.14429, found 500.14133.

#### 3.1.5. General Synthetic Procedures for the Synthesis of Compounds **B1**–**B7**

We replaced the *N*-Boc-piperazine of Series A with other *N*-Boc-heterocyclic compounds, replaced the phenyl isocyanate derivatives with 5-chloro-2-methylphenyl isocyanate of Series A, and the other steps were the same as those in Series A.

##### *N*-(5-chloro-2-methylphenyl)-3-((4-oxo-3,4-dihydroquinazolin-2-yl)methyl)-3,6-diazabicyclo[3.1.1]heptane-6-carboxamide (**B1**)

White solid, yield 43.5%, m.p. 181.2–182.3 °C. ^1^H NMR (400 MHz, DMSO-d_6_) δ 11.98 (s, 1H), 8.08 (d, *J* = 11.4 Hz, 2H), 7.73 (t, *J* = 7.5 Hz, 1H), 7.55 (d, *J* = 7.9 Hz, 1H), 7.47 (t, *J* = 7.4 Hz, 1H), 7.37 (s, 1H), 7.17 (d, *J* = 8.1 Hz, 1H), 7.07 (d, *J* = 8.3 Hz, 1H), 4.23 (s, 2H), 3.67 (s, 2H), 3.29 (s, 2H), 2.98 (d, *J* = 10.5 Hz, 2H), 2.25 (s, 1H), 2.11 (s, 3H), 1.87 (d, *J* = 7.5 Hz, 1H). ^13^C NMR (100 MHz, DMSO-d_6_) δ 156.66, 154.96, 139.03, 134.68, 131.99, 131.19, 130.76, 130.14, 126.79, 126.19, 124.93, 124.35, 122.57, 121.72, 120.53, 59.83, 58.03, 50.78, 27.91, 18.02. ESI-MS: calculated for C_22_H_22_ClN_5_O_2_ [M+H]^+^ 424.14620, found 424.14423.

##### *N*-(5-chloro-2-methylphenyl)-2-methyl-4-((4-oxo-3,4-dihydroquinazolin-2-yl)methyl)piperazine-1-carboxamide (**B2**)

White solid, yield 45.2%, m.p. 189.5–190.8 °C. ^1^H NMR (400 MHz, DMSO-d_6_) δ 11.84 (s, 1H), 8.15–8.06 (m, 2H), 7.80 (t, *J* = 7.6 Hz, 1H), 7.64 (d, *J* = 7.9 Hz, 1H), 7.50 (t, *J* = 7.3 Hz, 1H), 7.29 (s, 1H), 7.19 (d, *J* = 8.5 Hz, 1H), 7.07 (d, *J* = 8.2 Hz, 1H), 3.83–3.69 (m, 3H), 3.47 (d, *J* = 14.7 Hz, 1H), 3.21 (t, *J* = 11.1 Hz, 1H), 2.99–2.91 (m, 1H), 2.82 (d, *J* = 11.2 Hz, 1H), 2.62 (s, 1H), 2.41 (t, *J* = 10.4 Hz, 1H), 2.13 (s, 3H), 1.08 (d, *J* = 5.8 Hz, 3H). ^13^C NMR (100 MHz, DMSO-d_6_) δ 162.04, 155.41, 148.94, 139.96, 134.87, 131.90, 130.04, 127.42, 126.87, 126.29, 125.30, 124.36, 122.57, 121.76, 57.04, 55.19, 51.16, 50.53, 44.38, 17.89, 15.31. ESI-MS: calculated for C_22_H_24_ClN_5_O_2_ [M+H]^+^ 426.16185, found 426.16016.

##### (1S,4S)-*N*-(5-chloro-2-methylphenyl)-5-((4-oxo-3,4-dihydroquinazolin-2-yl)methyl)-2,5-diazabicyclo[2.2.1]heptane-2-carboxamide (**B3**)

White solid, yield 38.3%, m.p. 191.1–192.4 °C. ^1^H NMR (400 MHz, DMSO-d_6_) δ 11.91 (s, 1H), 8.10 (d, *J* = 7.9 Hz, 1H), 7.85–7.73 (m, 2H), 7.63 (d, *J* = 8.1 Hz, 1H), 7.52–7.45 (m, 2H), 7.20 (d, *J* = 7.9 Hz, 1H), 7.06 (d, *J* = 8.1 Hz, 1H), 4.46 (s, 1H), 3.67 (dt, *J* = 23.0, 11.8 Hz, 4H), 3.29 (s, 1H), 2.95 (d, *J* = 9.3 Hz, 1H), 2.73 (d, *J* = 9.3 Hz, 1H), 2.19 (s, 3H), 1.89 (d, *J* = 9.2 Hz, 1H), 1.74 (d, *J* = 9.5 Hz, 1H). ^13^C NMR (100 MHz, DMSO-d_6_) δ 162.08, 155.84, 154.36, 149.00, 139.67, 134.81, 131.91, 131.01, 130.12, 127.41, 126.85, 126.27, 124.65, 124.05, 121.82, 61.91, 60.24, 57.36, 50.86, 35.88, 17.89. ESI-MS: calculated for C_22_H_22_ClN_5_O_2_ [M+H]^+^ 424.14620, found 424.14468.

##### *N*-(5-chloro-2-methylphenyl)-4-((4-oxo-3,4-dihydroquinazolin-2-yl)methyl)-1,4-diazepane-1-carboxamide (**B4**)

White solid, yield 46.9%, m.p. 186.6–187.9 °C. ^1^H NMR (400 MHz, DMSO-d_6_) δ 11.89 (s, 1H), 8.11 (d, *J* = 7.9 Hz, 1H), 7.80 (d, *J* = 13.2 Hz, 2H), 7.64 (d, *J* = 7.8 Hz, 1H), 7.50 (t, *J* = 7.4 Hz, 1H), 7.35 (s, 1H), 7.19 (d, *J* = 8.6 Hz, 1H), 7.07 (d, *J* = 8.2 Hz, 1H), 3.63 (s, 2H), 3.56 (s, 4H), 2.78 (d, *J* = 28.8 Hz, 4H), 2.15 (s, 3H), 1.85 (s, 2H). ^13^C NMR (100 MHz, DMSO-d_6_) δ 162.08, 155.57, 148.92, 140.11, 134.82, 132.05, 131.85, 130.03, 127.46, 126.89, 126.26, 125.35, 124.24, 121.81, 120.53, 59.86, 55.80, 54.63, 46.28, 45.54, 27.92, 17.90. ESI-MS: calculated for C_22_H_24_ClN_5_O_2_ [M+H]^+^ 426.16185, found 426.16043.

##### (1R,5S)-*N*-(5-chloro-2-methylphenyl)-8-((4-oxo-3,4-dihydroquinazolin-2-yl)methyl)-3,8-diazabicyclo[3.2.1]octane-3-carboxamide (**B5**)

White solid, yield 37.4%, m.p. 189.5–191.7 °C. ^1^H NMR (400 MHz, DMSO-d_6_) δ 11.88 (s, 1H), 8.13 (dd, *J* = 7.9, 1.1 Hz, 1H), 7.93 (s, 1H), 7.84–7.78 (m, 1H), 7.65 (d, *J* = 7.8 Hz, 1H), 7.54–7.47 (m, 1H), 7.32 (d, *J* = 2.2 Hz, 1H), 7.19 (d, *J* = 8.4 Hz, 1H), 7.07 (dd, *J* = 8.1, 2.2 Hz, 1H), 3.72 (d, *J* = 10.6 Hz, 2H), 3.48 (s, 2H), 3.31 (s, 2H), 3.13 (d, *J* = 11.7 Hz, 2H), 2.15 (s, 3H), 2.00–1.88 (m, 2H), 1.64 (d, *J* = 7.5 Hz, 2H). ^13^C NMR (100 MHz, DMSO-d_6_) δ 162.03 (s), 156.48 (s), 155.69 (s), 149.01 (s), 140.10 (s), 134.85 (s), 131.85 (d, *J* = 13.4 Hz), 130.05 (s), 127.41 (s), 126.88 (s), 126.30 (s), 125.09 (s), 124.27 (s), 121.87 (s), 59.43 (s), 56.27 (s), 50.17 (s), 25.48 (s), 17.79 (s). ESI-MS: calculated for C_23_H_24_ClN_5_O_2_ [M+H]^+^ 438.16185, found 438.15988.

##### *N*-(5-chloro-2-methylphenyl)-3-((4-oxo-3,4-dihydroquinazolin-2-yl)methyl)-3,8-diazabicyclo[3.2.1]octane-8-carboxamide (**B6**)

White solid, yield 39.6%, m.p. 192.3–193.6 °C. ^1^H NMR (400 MHz, CDCl_3_) δ 9.67 (s, 1H), 8.29 (d, *J* = 7.9 Hz, 1H), 7.84 (s, 1H), 7.77 (t, *J* = 7.6 Hz, 1H), 7.67 (d, *J* = 7.9 Hz, 1H), 7.50 (t, *J* = 7.6 Hz, 1H), 7.08 (d, *J* = 7.9 Hz, 1H), 6.99 (d, *J* = 8.1 Hz, 1H), 6.14 (s, 1H), 4.30 (s, 2H), 3.59 (s, 2H), 2.73 (q, *J* = 10.7 Hz, 4H), 2.21 (s, 3H), 2.10 (s, 4H). ^13^C NMR (100 MHz, CDCl_3_) δ 162.08, 154.61, 154.22, 148.88, 139.54, 134.81, 131.88, 130.08, 126.96, 126.24, 125.31, 124.43, 122.58, 120.57, 60.27, 57.26, 54.35, 28.01, 17.99. ESI-MS: calculated for C_23_H_24_ClN_5_O_2_ [M+H]^+^ 438.16185, found 438.16016.

##### *N*-(5-chloro-2-methylphenyl)-3-((4-oxo-3,4-dihydroquinazolin-2-yl)methyl)tetrahydropyrimidine-1(2H)-carboxamide (**B7**)

White solid, yield 44.3%, m.p. 197.3–198.7 °C. ^1^H NMR (400 MHz, DMSO-d_6_) δ 8.16 (dd, *J* = 8.0, 1.2 Hz, 1H), 8.06 (d, *J* = 2.2 Hz, 1H), 7.83 (ddd, *J* = 8.5, 7.2, 1.6 Hz, 1H), 7.75 (s, 1H), 7.67 (d, *J* = 7.7 Hz, 1H), 7.56–7.51 (m, 1H), 7.12 (d, *J* = 8.1 Hz, 1H), 6.88 (dd, *J* = 8.1, 2.3 Hz, 1H), 6.77 (t, *J* = 5.6 Hz, 1H), 4.84 (s, 2H), 4.03 (s, 2H), 3.19 (d, *J* = 6.0 Hz, 2H), 2.72 (t, *J* = 7.1 Hz, 2H), 2.15 (s, 3H), 1.70 (p, *J* = 6.9 Hz, 2H). ^13^C NMR (100 MHz, DMSO-d_6_) δ 159.06 (s), 157.17 (s), 155.56 (s), 149.39 (s), 140.17 (s), 134.89 (s), 131.81 (s), 130.75 (s), 127.39 (s), 126.89 (s), 126.30 (s), 121.28 (s), 120.89 (s), 119.11 (s), 68.50 (s), 56.94 (s), 51.62 (s), 37.53 (s), 28.64 (s), 17.86 (s). ESI-MS: calculated for C_21_H_22_ClN_5_O_2_ [M+H]^+^ 412.14620, found 412.14468.

#### 3.1.6. General Synthetic Procedures for the Synthesis of Compounds **C1**–**C5**

We replaced the methyl 2-aminobenzoate of Series A with other methyl 2-aminobenzoate derivatives, replaced the *N*-Boc-piperazine of Series A with 6-*N*-Boc-3,6-diazabicyclo[3.1.1]heptane, and replaced the phenyl isocyanate derivatives of Series A with 5-chloro-2-methylphenyl isocyanate, and the other steps were the same as the steps in Series A.

##### *N*-(5-chloro-2-methylphenyl)-3-((7-fluoro-4-oxo-3,4-dihydroquinazolin-2-yl)methyl)-3,6-diazabicyclo[3.1.1]heptane-6-carboxamide (**C1**)

White solid, yield 36.4%, m.p. 193.2–195.1 °C. ^1^H NMR (400 MHz, DMSO-d_6_) δ 12.09 (s, 1H), 8.13 (t, *J* = 7.3 Hz, 1H), 8.04 (s, 1H), 7.36 (s, 1H), 7.34–7.24 (m, 2H), 7.15 (d, *J* = 8.3 Hz, 1H), 7.06 (d, *J* = 8.1 Hz, 1H), 4.23 (s, 2H), 3.68 (s, 2H), 2.96 (d, *J* = 10.5 Hz, 2H), 2.37 (d, *J* = 6.3 Hz, 1H), 2.25 (s, 2H), 2.10 (s, 3H), 1.86 (d, *J* = 7.6 Hz, 1H). ^13^C NMR (100 MHz, DMSO-d_6_) δ 164.80, 156.63, 138.92, 132.00, 131.01, 130.16, 129.19, 126.37, 124.83, 124.31, 122.58, 120.56, 118.69, 115.43, 59.84, 57.68, 50.63, 27.86, 18.01. ESI-MS: calculated for C_22_H_21_ClFN_5_O_2_ [M+H]^+^ 442.13678, found 442.13474.

##### *N*-(5-chloro-2-methylphenyl)-3-((7-chloro-4-oxo-3,4-dihydroquinazolin-2-yl)methyl)-3,6-diazabicyclo[3.1.1]heptane-6-carboxamide (**C2**)

White solid, yield 35.5%, m.p. 192.3–194.1 °C. ^1^H NMR (400 MHz, DMSO-d_6_) δ 12.14 (s, 1H), 8.09–8.00 (m, 2H), 7.56 (s, 1H), 7.49 (d, *J* = 8.4 Hz, 1H), 7.36 (s, 1H), 7.15 (d, *J* = 8.1 Hz, 1H), 7.06 (d, *J* = 8.2 Hz, 1H), 4.24 (s, 2H), 3.69 (s, 2H), 3.38 (s, 2H), 2.96 (d, *J* = 10.4 Hz, 2H), 2.37 (d, *J* = 6.3 Hz, 1H), 2.10 (s, 3H), 1.86 (d, *J* = 7.5 Hz, 1H). ^13^C NMR (100 MHz, DMSO-d_6_) δ 156.69, 139.32, 138.90, 131.95, 130.94, 130.19, 128.23, 127.06, 124.81, 124.33, 120.49, 59.81, 57.54, 50.57, 27.91, 17.96. ESI-MS: calculated for C_22_H_21_Cl_2_N_5_O_2_ [M+H]^+^ 458.10723, found 458.10510.

##### *N*-(5-chloro-2-methylphenyl)-3-((7-methyl-4-oxo-3,4-dihydroquinazolin-2-yl)methyl)-3,6-diazabicyclo[3.1.1]heptane-6-carboxamide (**C3**)

White solid, yield 37.2%, m.p. 197.7–199.3 °C. ^1^H NMR (400 MHz, DMSO-d_6_) δ 11.88 (s, 1H), 8.02 (s, 1H), 7.96 (d, *J* = 7.9 Hz, 1H), 7.38 (s, 1H), 7.33 (s, 1H), 7.28 (d, *J* = 8.1 Hz, 1H), 7.16 (d, *J* = 8.0 Hz, 1H), 7.06 (d, *J* = 8.1 Hz, 1H), 4.24 (s, 2H), 3.67 (s, 2H), 3.36 (s, 2H), 2.96 (d, *J* = 10.3 Hz, 2H), 2.37 (s, 4H), 2.11 (s, 3H), 1.88 (t, *J* = 12.0 Hz, 1H). ^13^C NMR (100 MHz, DMSO-d_6_) δ 161.98, 156.58, 154.91, 149.09, 145.09, 138.98, 131.96, 131.07, 130.15, 128.15, 127.26, 126.01, 124.87, 124.29, 119.30, 59.83, 57.70, 50.66, 27.92, 21.75, 17.98. ESI-MS: calculated for C_23_H_24_ClN_5_O_2_ [M+H]^+^ 438.16185, found 438.16043.

##### *N*-(5-chloro-2-methylphenyl)-3-((6-fluoro-4-oxo-3,4-dihydroquinazolin-2-yl)methyl)-3,6-diazabicyclo[3.1.1]heptane-6-carboxamide (**C4**)

White solid, yield 38.9%, m.p. 192.1–193.5 °C. ^1^H NMR (400 MHz, DMSO-d_6_) δ 12.14 (s, 1H), 8.04 (s, 1H), 7.75 (d, *J* = 8.6 Hz, 1H), 7.62 (s, 2H), 7.34 (s, 1H), 7.16 (d, *J* = 8.3 Hz, 1H), 7.06 (d, *J* = 8.1 Hz, 1H), 4.23 (s, 2H), 3.67 (s, 2H), 3.30 (s, 2H), 2.97 (d, *J* = 10.3 Hz, 2H), 2.36 (d, *J* = 6.2 Hz, 1H), 2.10 (s, 3H), 1.86 (d, *J* = 7.4 Hz, 1H). ^13^C NMR (100 MHz, DMSO-d_6_) δ 161.99, 156.58, 154.90, 149.09, 145.08, 138.98, 131.95, 131.07, 130.16, 128.14, 127.26, 126.01, 124.87, 124.29, 119.31, 59.84, 57.71, 50.67, 21.75, 17.98. ESI-MS: calculated for C_22_H_21_ClFN_5_O_2_ [M+H]^+^ 442.13678, found 442.13501.

##### *N*-(5-chloro-2-methylphenyl)-3-((6,7-difluoro-4-oxo-3,4-dihydroquinazolin-2-yl)methyl)-3,6-diazabicyclo[3.1.1]heptane-6-carboxamide (**C5**)

White solid, yield 35.8%, m.p. 194.4–195.8 °C. ^1^H NMR (400 MHz, DMSO-d_6_) δ 12.25 (s, 1H), 8.10 (s, 1H), 8.03 (t, *J* = 9.1 Hz, 1H), 7.79–7.71 (m, 1H), 7.19 (d, *J* = 8.2 Hz, 1H), 7.07 (d, *J* = 8.1 Hz, 1H), 5.76 (s, 1H), 3.50 (s, 2H), 3.47 (s, 4H), 2.54 (s, 4H), 2.14 (s, 3H). ^13^C NMR (100 MHz, DMSO-d_6_) δ 156.53, 156.10, 139.11, 138.88, 131.98, 130.80, 130.15, 126.36, 124.63, 124.19, 122.58, 120.55, 115.49, 113.53, 59.84, 57.37, 50.42, 27.68, 17.99. ESI-MS: calculated for C_22_H_20_ClF_2_N_5_O_2_ [M+H]^+^ 460.12736, found 460.12552.

### 3.2. Biological Evaluation

#### 3.2.1. Cell Lines and Cell Culture

HCT-15, HCC1937, MDA-MB-231, LO2, and NCM460 cell lines were purchased from Procell (www.procell.com.cn). HCT-15, HCC1937, MDA-MB-231, LO2, and NCM460 cell lines were cultured in DMEM medium or RPMI 1640 medium (www.thermofisher.cn) with 10% fetal bovine serum (FBS). All cells were incubated at 37 °C in a humidified incubator (Thermo Scientific) with 5% CO_2_.

#### 3.2.2. Cytotoxicity Assay

Cells were seeded into 96-well plates with 5 × 10^3^ cells per well. After treatment with target compounds at the different concentrations, MTT was added and the cells were incubated for another 4 h. The IC_50_ values of the selected compounds were evaluated by the same method. The cells were treated with compounds at different concentrations. The OD values were detected using a microplate reader at 570 nm.

#### 3.2.3. PARP1 Enzyme Inhibition Assay

The PARP1 enzyme inhibition activity was determined for compounds using a commercially available PARP1 enzyme activity kit (Sigma-Aldrich, catalog No. 17-10149), according to the manufacturer’s protocol.

#### 3.2.4. Immunofluorescence Analyses of PAR and γH2AX

The cells were seeded in 6-well plates with 1 × 10^5^ cells per well and incubated for 24 h. Then, the cells were co-incubated with different concentrations of **B1** (1.25, 2.5, 5, 10, and 20 μM) for 48 h. Cells were fixed with addition of 4% paraformaldehyde, and immunostaining blocking solution was added at room temperature for 20 min. The immunostaining blocking solution was aspirated, the primary antibody γH2AX (Beyotime, C2035S) or PAR (Enzo Life Sciences, ELS-BML-SA216-0100) was added and incubated at room temperature for 1 h, and anti-rabbit 488 was added and incubated for 1 h at room temperature. Then, a cytosolic staining solution (DAPI) was added for staining, the cytosolic staining solution was aspirated, and the well washed 3 times with washing solution. Photographs were taken through a Leica SP8 Laser confocal microscope.

#### 3.2.5. Cell Apoptosis Assay

The cells were seeded in 6-well plates with 1 × 10^5^ cells per well and incubated for 24 h. Then, the cells were co-incubated with different concentrations of **B1** (1.25, 2.5, 5, 10, and 20 μM) for 48 h. The cells were digested, collected, and resuspended in the binding buffer. After adding 5 μL of Annexin V-FITC and 10 μL of propidium iodide (PI), the cells were incubated for 30 min away from light; this was followed by detection using flow cytometry (Beckman Coulter).

#### 3.2.6. Western Blot Analysis

Different concentrations of **B1** (1.25, 2.5, 5, 10, and 20 μM) were co-incubated with the cells for 48 h. Subsequently, the cells were lysed with RIPA lysis buffer and protein samples were prepared. Denatured proteins were separated by 10% SDS-PAGE electrophoresis and transferred to a PVDF membrane. The membrane was blocked with 5% blocking solution for 2 h. The membrane was incubated overnight at 4 °C with specific primary antibody. Then, the membrane was rinsed three times with TBST, secondary antibody was added, and the membrane was incubated at room temperature for 2 h. Finally, the target protein was detected by the ECL detection system. The relative expression was quantified using Image J (National Institutes of Health).

#### 3.2.7. ROS Assay

Cell culture methods were the same as for the cell apoptosis assay. Then, fresh medium containing the DCFH-DA probe was added, and the incubation was continued at 37 °C for 2 h; this was followed by detection using flow cytometry (Beckman Coulter), and photographs were taken through a Leica SP8 Laser confocal microscope.

#### 3.2.8. Mitochondrial Membrane Potential Assay

The experimental operation was the same as ROS, only replacing the DCFH-DA probe with the JC-1 probe.

#### 3.2.9. In Vivo Anti-Tumor Study

All experimental procedures were performed according to the National Institutes of Health guidelines for the use of laboratory animals, and an application was made to the Institutional Animal Care and Use Committee, Shandong Second Medical University, which was approved. BRCA2-mutant HCT-15 cells (5 × 10^6^ cells) were injected into the subcutaneous tissue of 6-week-old female BALB/c nude mice (Jinan Pengyue Experimental Animal Breeding Co.). When the tumor volume reached about 80–150 mm^3^, the mice were randomly divided into three groups (the control group, and different concentration administration group) and administered for 14 consecutive days, while the tumor volume was measured once every two days and the body weight of the mice was recorded. At the end stage, mice were killed by neck-breaking and tumor tissues were removed. All the photographs were taken through a Leica SP8 Laser confocal microscope.

#### 3.2.10. Molecular Docking

In order to accurately predict the docking posture, we used two different molecular docking programs, AMDock [37] and molecular operating environment software (MOE, Chemical Computing Group, 2019.0101 edition), to detect the binding ability of different compounds to PARP. The crystal structural files of PARP were downloaded from the protein database (PDB: 7KK4). Protein and ligand processing was carried out using the tools that come with the software, which performs repair treatments such as hydrogenation, the removal of metal ions, side-chain repair, addition of missing atom types, repair of side-chain amino acids, field optimization, and other repair treatments for proteins. The active pocket creation method is to extract the original ligand from the protein to obtain an active docking pocket for docking; all other parameters remain the default software standard.

#### 3.2.11. Molecular Dynamics

The docked proteins were separated from the small-molecule ligands, and the small-molecule force field files were generated by the antechamber tool in Ambertools software. The small-molecule force field files were generated by the antechamber tool in Ambertools software, and then converted to gromacs force field files by the Acpype software tool. The GAFF force field was used for small molecules, and the AMBER14SB force field and TIP3P water model were used for proteins. The protein and small-molecule ligand files were merged to construct the simulation system for the complexes. The molecular dynamics (MD) simulations were performed using the Gromacs2022 program under constant temperature and pressure and periodic boundary conditions. In the MD simulations, all hydrogen bonds were bound using the LINCS algorithm with an integration step of 2 fs. Electrostatic interactions were calculated using the (particle-mesh Ewald) PME method with a cutoff value of 1.2 nm, and the cutoff value of non-bonded interactions was set at 10 Å and updated every 10 steps. The simulation temperature was controlled by the V-rescale temperature coupling method at 298 K, and the pressure was controlled by the Berendsen method at 1 bar. In total, 100 ps of NVT and NPT equilibrium simulations were performed at 298 K. A 30 ns MD simulation was performed for the complex system, and the conformation was saved every 10 ps. After completion of the simulations, the trajectories were analyzed using VMD and Pymol, and the free energy of binding of MMPBSA between the protein and the small-molecule ligand was analyzed using the g_mmpbsa program. All 2D and 3D drawings were created through Pymol, ChimeraX, LigPlot+ v.2.2.8, and Discovery Studio 2019 Client.

#### 3.2.12. Statistical Analysis

The data were visualized using GraphPad Prism software, and statistical analysis was performed using the two-sided Student’s t-test or one-way ANOVA followed by an appropriate post hoc test. Statistical significance was denoted by ** *p* < 0.01, *** *p* < 0.001. The IC_50_ values of tested compounds were calculated by GraphPad Prism software.

## 4. Conclusions

In summary, a novel lead compound **B1** was discovered based on **IN17** with a 4-hydroxyquinazoline fragment. **B1** has the potential to effectively target intracellular PARP and enhance sensitivity in primary PARPi-resistant cells. The mechanism of synthetic lethality showed that **B1** can be effective in inhibiting intracellular PAR formation and promoting γH2AX accumulation. Furthermore, **B1** can induce apoptosis in HCT-15 and HCC1937 cell lines at a concentration of 5 μM, which was accompanied by an upregulation of Bax expression, downregulation of Bcl-2 expression, and activation of Caspase-3 in a process that exhibited concentration dependence. We discovered that **B1** can induce ROS production and cause mitochondrial membrane depolarization, accelerating cell apoptosis, which may serve as an additional mechanism to overcome PARPi resistance. In vivo studies demonstrated that the compound **B1** significantly suppressed the growth of HCT-15 xenografts in nude mice. Moreover, it exhibited favorable safety profiles, supporting its potential as a novel anticancer drug. Finally, we conducted a preliminary study of the binding mechanism using molecular docking and molecular dynamics and found a stable binding mode between **B1** and the protein, with a hydrogen-bonding interaction with ASP766 facilitating the antiproliferative activity. The findings provide useful structures for the discovery of novel PARPi to overcome PARPi resistance.

## Figures and Tables

**Table 1:** IC_50_ values of the compounds **A1**–**A39** against HCT-15 and HCC1937 cell lines.

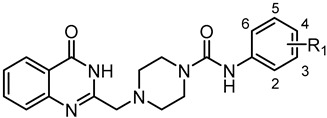			
No.	R_1_	IC_50_ (μM) ^a^	
		HCT-15	HCC1937
**A1**	2-F	18.39 ± 0.91	16.63 ± 1.12
**A2**	3-F	34.89 ± 2.23	>50
**A3**	4-F	>50	>50
**A4**	2-Cl	19.12 ± 1.12	14.65 ± 1.67
**A5**	3-Cl	19.05 ± 0.79	25.89 ± 0.96
**A6**	4-Cl	21.35 ± 0.86	>50
**A7**	2-Br	29.61 ± 1.37	40.28 ± 3.32
**A8**	3-Br	27.65 ± 2.56	47.41 ± 2.96
**A9**	4-Br	>50	>50
**A10**	2-CH_3_	15.89 ± 0.85	14.73 ± 1.03
**A11**	3-CH_3_	25.57 ± 1.17	>50
**A12**	4-CH_3_	>50	>50
**A13**	2-CF_3_	20.23 ± 0.78	>50
**A14**	3-CF_3_	20.02 ± 1.15	17.67 ± 0.77
**A15**	4-CF_3_	15.56 ± 0.56	>50
**A16**	2-OCH_3_	30.65 ± 2.13	>50
**A17**	3-OCH_3_	>50	>50
**A18**	4-OCH_3_	>50	>50
**A19**	2,3-F	19.07 ± 0.59	>50
**A20**	2,5-F	33.13 ± 2.16	41.67 ± 1.89
**A21**	2-F,3-CH_3_	28.29 ± 1.55	>50
**A22**	2-F,4-CH_3_	21.73 ± 1.16	>50
**A23**	2-F,5-CH_3_	26.02 ± 1.67	44.20 ± 3.47
**A24**	2-F,4-CF_3_	22.77 ± 1.82	44.09 ± 2.19
**A25**	2-Cl,6-F	41.24 ± 1.44	>50
**A26**	2-Cl,5-CF_3_	37.29 ± 2.76	>50
**A27**	2-Cl,5-CH_3_	25.37 ± 1.48	17.52 ± 0.66
**A28**	2-CH_3_,3-F	30.31 ± 3.30	>50
**A29**	2-CH_3_,4-F	29.83 ± 1.57	>50
**A30**	2-CH_3_,5-F	18.31 ± 0.44	39.46 ± 1.84
**A31**	2-CH_3_,3-Br	>50	>50
**A32**	2-CH_3_,5-Cl	10.93 ± 0.71	11.35 ± 0.73
**A33**	2,6-CH_3_	35.01 ± 2.28	39.58 ± 1.35
**A34**	3,5-F	47.25 ± 3.35	>50
**A35**	3,5-Cl	21.15 ± 0.89	>50
**A36**	3-Cl,4-F	25.20 ± 1.66	>50
**A37**	3,4-CH_3_	22.49 ± 1.49	46.41 ± 2.96
**A38**	3,5-CH_3_	18.02 ± 0.88	17.85 ± 1.32
**A39**	3,5-CF_3_	20.99 ± 2.33	42.16 ± 2.77
**Olaparib ^b^**		45.53 ± 3.13	37.07 ± 1.89

^a^ IC_50_: concentration of the compound producing 50% cell growth inhibition after 72 h of drug exposure, as determined by the MTT assay. Each experiment was performed at least three times. ^b^ Olaparib served as the positive control.

**Table 2:** IC_50_ values of the compounds **B1**–**B7** against HCT-15 and HCC1937 cell lines.

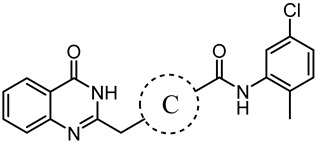			
No.	C	IC_50_ (μM) ^a^	
		HCT-15	HCC1937
**B1**	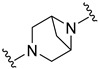	2.89 ± 0.78	3.26 ± 0.38
**B2**	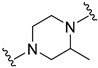	6.37 ± 2.83	5.89 ± 0.50
**B3**	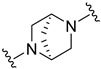	12.50 ± 1.49	16.46 ± 0.94
**B4**	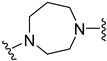	5.65 ± 1.29	5.77 ± 0.99
**B5**	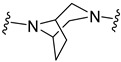	10.95 ± 2.40	9.61 ± 1.35
**B6**	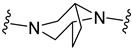	7.27 ± 1.28	7.52 ± 1.37
**B7**	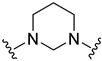	13.72 ± 1.21	57.91 ± 3.33
**Olaparib ^b^**		45.53 ± 3.13	37.07 ± 1.89

^a^ IC_50_: concentration of the compound producing 50% cell growth inhibition after 72 h of drug exposure, as determined by the MTT assay. Each experiment was performed at least three times. ^b^ Olaparib served as the positive control.

**Table 3:** IC_50_ values of the compounds **C1**–**C5** against HCT-15 and HCC1937 cell lines.

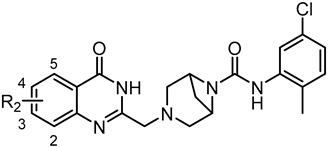			
No.	R_2_	IC_50_ (μM) ^a^	
		HCT-15	HCC1937
**C1**	3-F	24.12 ± 2.25	9.22 ± 1.19
**C2**	3-Cl	23.01 ± 1.93	32.38 ± 2.66
**C3**	3-CH_3_	28.36 ± 2.87	30.14 ± 3.12
**C4**	4-F	9.84 ± 0.63	8.49 ± 0.71
**C5**	3,4-F	29.57 ± 2.16	54.98 ± 4.28
**Olaparib ^b^**		45.53 ± 3.13	37.07 ± 1.89

^a^ IC_50_: concentration of the compound producing 50% cell growth inhibition after 72 h of drug exposure, as determined by the MTT assay. Each experiment was performed at least three times. ^b^ Olaparib served as the positive control.

**Table 4:** IC_50_ values of the compound **B1** against cell lines and PARP1.

No.	IC_50_ (μM) ^a^								PARP1 IC_50_ (nM) ^a^
	HCT-15		HCC1937		MDA-MB-231		LO2	NCM460	
	3-Days	7-Days	3-Days	7-Days	3-Days	7-Days	3-Days	3-Days	
**B1**	2.89 ± 0.78	0.13 ± 0.03	3.26 ± 0.38	0.17 ± 0.04	11.29 ± 1.93	1.33 ± 0.51	>100	>100	63.81 ± 2.12
**Olaparib ^b^**	45.53 ± 3.13	19.25 ± 0.75	37.07 ± 1.89	15.25 ± 0.87	33.93 ± 2.57	9.22 ± 1.12	90.83 ± 2.61	>100	7.30 ± 1.43

^a^ IC_50_: Each experiment was performed at least three times. ^b^ Olaparib served as the positive control.

## Data Availability

Data are contained within the article and Supplementary Materials.

## Supplementary Materials

The following supporting information can be downloaded at: https://www.mdpi.com/article/10.3390/molecules29061407/s1. S1. Table S1, Table S2, Table S3; S2. The Synthesis method of Compounds Y1-Y5; S3. The Synthesis method of Compounds IN17, IN17-(1-5); S4. 1H-NMR and 13C-NMR Spectral of synthetic compounds; S5. Mass Spectral of synthetic compounds; S6. IR Spectral of compound B1.
