# Supplementary material for: Discovery of Novel 4-Hydroxyquinazoline Derivatives: In Silico, In Vivo and In Vitro Studies Using Primary PARPi-Resistant Cell Lines
Source: Molecules. 2024 Mar 21;29(6):1407. doi: 10.3390/molecules29061407 (PMC10974284; doi:10.3390/molecules29061407)

Supporting Information

S1: TableS1, TableS2, TableS3 ……….. 1-7

S2: The Synthesis method of Compounds Y1-Y5 ……….. 8-9

S3: The Synthesis method of Compounds IN17, IN17-(1-5) …….. 10-14

S4: ^{1}H-NMR and ^{13}C-NMR Spectral of synthetic compounds ………..15-67

S5: Mass Spectral of synthetic compounds ……….. 68-95

S6: IR Spectral of compound B1 ……….. 96

S1: Table S1, TableS2, TableS3

Table S1. The inhibition rate of in-hourse compounds *in vitro*.

No.	MF	MW	SMILES	Purity	Inhibition rate (%) (50μM)[a]		Inhibition rate (%) (0.5μM)
					HCT-15	HCC1937	PARP1
**1**	C7H6N2OS	166.2	NC1=NC2=CC=C(O)C=C2S1	>95%	13.7	12.7	
**2**	C16H18FN3O3	319.33	OC(C1=CN(CC)C2=CC(N3CCNCC3)=C(F)C=C2C1=O)=O	>95%	31.9	10.3	
**3**	C19H22FN3O4	375.39	OC(C1=CN(C2CC2)C3=C(OC)C(N4CC(C)NCC4)=C(F)C=C3C1=O)=O	>95%	52.3	19.5	
**4**	C13H10F4N2O2	302.22	CC(N1CC2=C(F)C=CC=C2C(F)(F)F)=CC(NC1=O)=O	>95%	48.9	16.5	
**5**	C17H13N3O5S2	403.43	OC(C1=CC=CC=C1C(NC2=CC=C(S(=O)(NC3=NC=CS3)=O)C=C2)=O)=O	>95%	40.5	18.1	
**6**	C18H14ClFN4O4	404.77	FC(C=C1)=C(Cl)C=C1NC2=NC=NC3=C2C=C([N+]([O-])=O)C(O[C@H]4COCC4)=C3	>95%	69.7	44.1	
**7**	C26H19Cl3F3N3O4S	632.86	O=C(NC1=CC=C(Cl)C(C(F)(F)F)=C1)C2=CC3=C(N4CCN(S(=O)(C5=C(Cl)C=CC=C5Cl)=O)CC4)C=CC=C3O2	>95%	10	6.5	
**8**	C13H5F2NO2	245.18	O=C(C1=C(F)C=CC(F)=C12)C3=C(C=NC=C3)C2=O	>95%	51.4	54.9	9.1
**9**	C16H15F6N5O*H2O*H3O4P	523.32	FC1=CC(C[C@H](CC(N2CCN3C(C2)=NN=C3C(F)(F)F)=O)N)=C(F)C=C1F	>95%	29.2	22.4	
**10**	C10H10N2O	174.19	CC(C1)=NN(C2=CC=CC=C2)C1=O	>95%	26.9	15.7	
**11**	C26H20Cl2N4O4S	555.43	O=C(NC1=CC(C#N)=CC=C1)C2=CC3=C(N4CCN(S(=O)(C5=C(Cl)C=CC=C5Cl)=O)CC4)C=CC=C3O2	>95%	11.9	8.4	
**12**	C26H19Cl3F3N3O4S	632.86	O=C(NC1=CC=C(Cl)C(C(F)(F)F)=C1)C2=CC3=C(N4CCN(S(=O)(C5=C(Cl)C=CC=C5Cl)=O)CC4)C=CC=C3O2	>95%	8.2	11.1	
**13**	C28H36ClN5O3S	558.13	ClC(C=N1)=C(NC2=C(S(=O)(C(C)C)=O)C=CC=C2)N=C1NC3=C(OC(C)C)C=C(C4CCNCC4)C(C)=C3	>95%	52.8	42.8	11.1
**14**	C11H15ClN4O	254.72	ClC1=NC2=C(NC([C@@H](CC)N2C(C)C)=O)C=N1	>95%	39	14.1	

**15**	C23H16F2N2O4	422.39	O=C(NC1=CC(C(OC)=O)=CC=C1)C2=CC3=C(NC4=CC=C(F)C(F)=C4)C=CC=C3O2	>95%	21	1.2	
**16**	C24H20ClN5O2	445.9	N#CC1=C(NC2=CC=C(OCC3=NC=CC=C3)C(Cl)=C2)C4=CC(N)=C(OCC)C=C4N=C1	>95%	52.1	66.6	5.7
**17**	C20H19F2N5O2	399.4	O=C1NC(CN2CCN(C(NC3=CC=C(F)C=C3F)=O)CC2)=NC4=CC=CC=C41	>95%	63.3	60.6	53.1
**18**	C20H24O5S	376.46	CC(C1=CC=C(CCOS(=O)(C2=CC=C(C)C=C2)=O)C=C1)(C)C(OC)=O	>95%	41.1	32	
**19**	C15H14ClN3O4S	367.8	O=C(N[C@H]1[C@](SCC(Cl)=C2C(O)=O)([H])N2C1=O)[C@H](N)C3=CC=CC=C3	>95%	46.9	8.9	
**20**	C16H18N3NaO4S	371.38	O=C1NC(CN2CCN(C(NC3=CC=C(F)C=C3F)=O)CC2)=NC4=CC=CC=C41	>95%	50.4	6.5	
**21**	C14H8FNO2Se	320.19	O=C1N(C(C2=CC=CC=C2F)=O)[Se]C3=CC=CC=C31	>95%	15.6	2.3	
**22**	C26H28Cl2N4O4	531.43	CC(N1CCN(C2=CC=C(OC[C@@H]3O[C@@](CN4C=CN=C4)(C5=CC=C(Cl)C=C5Cl)OC3)C=C2)CC1)=O	>95%	16.8	51.5	9.7
**23**	C10H14N2O	178.23	OC1=CC=C(N2CCNCC2)C=C1	>95%	41.9	9.2	
**24**	C5H5N3O	123.11	O=C(C1=NC=CN=C1)N	>95%	23.1	15.7	
**25**	C16H13Cl3N2OS	387.71	ClC1=CC=C(C(OCC2=C(Cl)SC=C2)CN3C=CN=C3)C(Cl)=C1	>95%	43.2	58.6	
**26**	C12H12N2O2S	248.3	O=S(C1=CC=C(N)C=C1)(C2=CC=C(N)C=C2)=O	>95%	14.6	22.2	
**27**	C22H19N4NaO8S2	554.52	O=C1N(C(C([O-])=O)=C2C[N+]3=CC=C(C(N)=O)C=C3)[C@H](SC2)[C@@H]1NC([C@H](S(=O)([O-])=O)C4=CC=CC=C4)=O.[Na+]	>95%	27	20.3	
**28**	C13H12F2N6O	306.27	OC(CN1C=NC=N1)(CN2C=NC=N2)C3=C(F)C=C(F)C=C3	>95%	40.5	4.6	
**29**	C24H35Cl2N3O4	500.45	O=C(NC1=C(C)C=CC=C1C)CN2CCN(CC(O)COC3=CC=CC=C3OC)CC2.[H]Cl.[H]Cl	>95%	33.4	25.4	
**30**	C14H9NO2Se	302.2	O=C(C1=CC=CC=C1)OC2=N[Se]C3=CC=CC=C32	>95%	12.4	5.4	

**31**	C35H38Cl2N8O4	705.63	O=C1N(C(C)CC)N=CN1C2=CC=C(N3CCN(C4=CC=C(OC[C@@H]5O[C@@](CN6N=CN=C6)(C7=CC=C(Cl)C=C7Cl)OC5)C=C4)CC3)C=C2	>95%	50.4	51.1	7.8
**32**	C22H19ClO3	366.83	O=C1C([C@H]2CC[C@H](C3=CC=C(Cl)C=C3)CC2)=C(O)C(C4=C1C=CC=C4)=O	>95%	47.3	50.8	39.2
**33**	C11H13BrN2O5	333.13,	OC[C@@H]1[C@H](C[C@H](N2C(NC(C(/C=C/Br)=C2)=O)=O)O1)O	>95%	28.5	17.3	
**34**	C11H16N4O5	284.26	O=C(C1=CC=C(C)C(NC(N)=N)=C1)OCC.O[N+]([O-])=O	>95%	35.3	31.9	
**35**	C24H34O5	402.52	O=C(C[C@@](CC1=O)([H])[C@](C)(CC1)[C@@]2([H])C3=O)[C@@]2([H])[C@@](CC[C@]4([H])[C@H](C)CCC(O)=O)([H])[C@]34C	>95%	12.3	21.4	
**36**	C16H17N7O2S	371.41	N#CCC1(N2N=CC(C3=C4C(NC=C4)=NC=N3)=C2)CN(S(=O)(CC)=O)C1	>95%	46.6	64.6	12.7
**37**	C24H29N7O2	447.53	O=C1C(C(C)=O)=C(C)C2=CN=C(NC3=NC=C(N4CCNCC4)C=C3)N=C2N1C5CCCC5	>95%	49.7	52.4	45.6
**38**	C17H14N4O2	306.31	O=C(O)C1=CC=C(C)C(NC2=NC=CC(C3=CC=CN=C3)=N2)=C1	>95%	22.4	22.4	
**39**	C21H20FN3O6S	461.46	O=C(C1=C(SC2C)N2C3=C(C=C(F)C(N4CCN(CC5=C(C)OC(O5)=O)CC4)=C3)C1=O)O	>95%	15.7	15.7	
**40**	C7H8N2O3S	200.21	O=C(C(N12)=CCS[C@]2([H])[C@H](N)C1=O)O	>95%	13.8	13.8	
**41**	C16H12O6	300.26	O=C1C=C(C2=CC=C(OC)C(O)=C2)OC3=CC(O)=CC(O)=C13	>95%	30.5	30.5	
**42**	C16H12O6	300.26	O=C1C=C(C2=CC=C(O)C(O)=C2)OC3=CC(OC)=CC(O)=C13	>95%	1.6	1.6	
**43**	C16H12O5	284.26	O=C1C=C(C2=CC=C(O)C=C	>95%	33.5	31.5	

			2)OC3=CC(OC)=CC(O)=C13				
**44**	C19H20N4	304.38	CCCC1=NC2=CC(C3=NC4=CC=CC=C4N3C)=CC(C)=C2N1	>95%	47.5	43.2	
**45**	C10H12N2O2	192.21	O=C1N(C2=CC=C(N)C=C2)CCOC1	>95%	33.6	24.4	
**46**	C11H9NO3	203.19	O=C1N(C[C@@H]2OC2)C(C3=C1C=CC=C3)=O	>95%	22.1	38.5	
**47**	C21H21N3O5	395.4	O=C1N(C[C@H](O)CNC2=CC=C(N3C(COCC3)=O)C=C2)C(C4=C1C=CC=C4)=O	>95%	31.2	46.9	
**48**	C10H10BrNO	240.1	O=C1NC2=CC=CC=C2CCC1Br	>95%	35.8	22.3	
**49**	C8H15ClN4O	218.68	CCCC1=NN(C)C(C(N)=O)=C1N.Cl	>95%	34.8	29	
**50**	C15H17N5O2	299.33	CC(C)(C)C(OCN1C=CC2=C(C3=CNN=C3)N=CN=C21)=O	>95%	33.6	44.7	
**51**	C18H22O2	270.36	C[C@]1([C@](CC2)([H])[C@]3([H])CCC4=C(C=CC(O)=C4)[C@@]3([H])CC1)C2=O	>95%	65.8	59.3	1.5
**52**	C16H8FNO2	265.24	N#CC1=CC(C=C2OC(C3=C2C=CC=C3)=O)=CC=C1F	>95%	46.2	41.5	
**53**	C18H22N4O	310.39	O=C(C1=C(C2=CC=CC=C2C)C=C(N3CCN(C)CC3)N=C1)N	>95%	16.5	49.6	
**54**	C17H14F3N3O2S	381.37	O=S(C1=CC=C(N2N=C(C(F)(F)F)C=C2C3=CC=C(C)C=C3)C=C1)(N)=O	>95%	33.5	33.1	
**55**	C16H20BrNO3	354.24	O=C(N1C[C@H](C(CBr)=O)[C@H](CC)C1)OCC2=CC=CC=C2	>95%	52.7	43.2	13.5
**56**	C18H20N4O4S	388.44	O=C(OC(C)(C)C)NC1=CN=C(N(S(=O)(C2=CC=C(C)C=C2)=O)C=C3)C3=N1	>95%	37.5	53.2	
**57**	C14H18ClF3N2O	322.75	O=C1N(CC(F)(F)F)[C@H](C)[C@H](C2=CC=CC=C2)C[C@@H]1N.[H]Cl	>95%	44.4	35.2	
**58**	C15H11N3O3	281.26	O=C(C1=CN=C2C(C[C@@]3(C4=CC=CN=C4NC3=O)C2)=C1)O	>95%	42.8	36	
**59**	C25H33F2NO2Si	445.61	O=C1[C@H](C2=CC=CC(F)=C2F)CC[C@@H](O[Si](C(C)C)(C(C)C)C(C)C)C3=NC=CC=C31	>95%	48.2	46.5	

**60**	C11H16Cl2N4O	291.17	O=C1N(C2CCNCC2)C3=CC=CN=C3N1.Cl.Cl	>95%	29.4	32.5	
	**Olaparib**[b]			>95%	53.3	55.8	99.9

[a] Inhibition rate (%): cells were exposed to compounds (50 μM) for 72 h, and the inhibition rate was determined by the MTT assay. Each experiment was performed at least three times.[b] Olaparib served as the positive control.

All the compounds come from the present laboratory, including the active intermediates purchased before and the compounds with unidentified activity synthesized by ourselves.

We tested the PARP1 enzyme inhibitory activity of those compounds whose cell inhibition rates exceeded 50% on HCT-15 and HCC1937 cell lines. Finally, **IN17** was found to have the same anti-proliferative activities as Olaparib against two cells, and it can target PARP1 well. Compound **IN17** had great potential for modification.

Table S2. IC_{50} values of compounds **IN17** against PARP1, HCT-15 and HCC1937 cell lines.

No.	IC_{50} (μM)[a]		PARP1 IC_{50} (nM)[b]
	HCT-15	HCC1937	
**IN17**	33.45 ± 1.79	34.29 ± 2.68	471.25 ± 3.18
**Olaparib**[c]	45.53 ± 3.13	37.07 ± 1.89	7.30 ± 1.43

[a] IC_{50}: concentration of the compound producing 50% cell growth inhibition after 72 h of drug exposure, as determined by the MTT assay. Each experiment was performed at least three times.

[b] The IC_{50} values were presented as mean±S.D. of three independent determinations. [c] Olaparib served as the positive control.

Table S3. IC_{50} values of compounds **IN17-(1-5)** against HCT-15 and HCC1937 cell lines.

No.	IC_{50} (μM) [a]	
	HCT-15	HCC1937
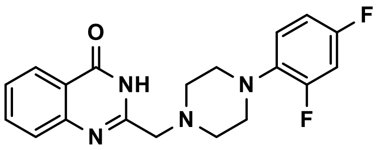	>100	>100
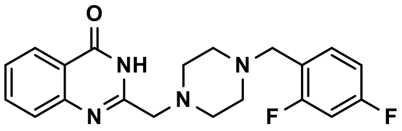	>100	>100
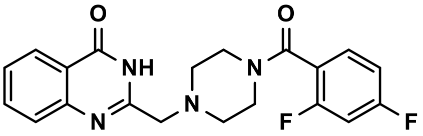	>100	>100
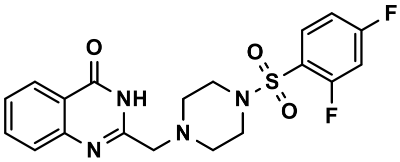	>100	>100
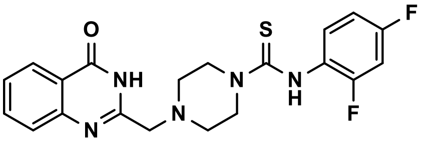	55.15 ± 4.08	46.41 ± 2.76
**Olaparib[b]**	45.53 ± 3.13	37.07 ± 1.89

[a] IC_{50}: concentration of the compound producing 50% cell growth inhibition after 72 h of drug exposure, as determined by the MTT assay. Each experiment was performed at least three times.

[b] Olaparib served as the positive control.

S2: The Synthesis method of Compounds Y1-Y5

S2: The Synthesis method of Compounds Y1-Y5

Procedure:

To a solution of triphosgene (2.96 g, 10 mmol) in toluene was added triethylamine (10.12 g, 0.1 mol) and the mixture was stirred at room temperature for 5 min. The aniline derivative was dissolved in toluene and added dropwise to the reaction mixture at 0 ℃. The mixture was stirred at 0 ℃ for 1 h, and then stirred for at 100 ℃ 1 h. After completion of the reaction, toluene was removed under vacuo, then 60 mL water was added, extracted with ethyl acetate (30 mL × 3) and washed with saturated sodium chloride (30 mL). The combined organic layer was dried on anhydrous sodium sulfate and concentrated in vacuo. The resulting mixture was concentrated in vacuo to obtain the crude target product, and this crude target product was used for the next step without any treatment.

S3: The Synthesis method of Compounds IN17，IN17-(1-5)

S3: The Synthesis method of Compounds IN17，IN17-(1-5)

1.1 The Synthesis method of Compounds IN17

Procedure:

To a solution of the compound **4** (0.28 g, 1 mmol) in tetrahydrofuran was added 2,4-difluorophenyl isocyanate (0.31 g , 2 mmol) and triethylamine (0.5 mL, 5 mmol). The reaction mixture was stirred at room temperature for 4 h. Then the mixture was filtered through a pad of Celite and washed with ethyl acetate. The organic solvent was removed under vacuum and the residue was purified by silica-gel column chromatography to give the compound **IN17**. White solid, yield 55.7 %.

N-(2,4-difluorophenyl)-4-((4-oxo-3,4-dihydroquinazolin-2-yl)methyl)piperazine-1-carboxamide:

^{1}H NMR (400 MHz, CDCl3) δ 9.95 (s, 1H), 8.29 (d, J = 7.9 Hz, 1H), 7.97 (dd, J = 15.6, 8.8 Hz, 1H), 7.78 (t, J = 7.5 Hz, 1H), 7.68 (d, J = 8.1 Hz, 1H), 7.50 (t, J = 7.5 Hz, 1H), 6.85 (dd, J = 12.7, 7.5 Hz, 2H), 6.45 (s, 1H), 3.65 (s, 2H), 3.61 (s, 4H), 2.68 (s, 4H). ^{13}C NMR (100 MHz, DMSO-d_6) δ 162.07, 155.50, 154.88, 148.85, 134.85, 128.22, 127.51, 126.98, 126.26, 121.84, 111.32, 111.07, 104.69, 104.44, 104.18, 60.91, 52.88, 44.13. ESI-MS: calculated for $C_{20}H_{19}F_2N_5O_2$ $[M+H]^+$ 400.15068, found 400.14975.

1.2 The Synthesis method of Compounds IN17-1

Procedure:

To a solution of the compound **2** (0.20 g, 1 mmol) in N,N-dimethylformamide was added 1-(2,4-difluorophenyl)piperazine (0.24 g ,1.2 mmol) and K_2CO_3 (0.41 g, 3 mmol). The reaction mixture was refluxed at 80 °C for 1 h. After cooling to room temperature, the mixture was filtered through a pad of Celite and washed with ethyl acetate. The organic solvent was removed under vacuum and the residue was purified by silica-gel column chro matography to give the compound **IN17-1**. White solid, yield 56.3 %.

2-((4-(2,4-difluorophenyl)piperazin-1-yl)methyl)quinazolin-4(3H)-one:

^{1}H NMR (400 MHz, DMSO-d_6) δ 12.01 (s, 1H), 8.13 (d, J = 7.7 Hz, 1H), 7.81 (t, J = 7.4 Hz, 1H), 7.68 (d, J = 8.1 Hz, 1H), 7.52 (t, J = 7.2 Hz, 1H), 7.23 – 7.15 (m, 1H),

7.07 (dd, J = 15.6, 8.0 Hz, 1H), 6.99 (t, J = 8.3 Hz, 1H), 3.53 (s, 2H), 3.00 (s, 4H), 2.69 (s, 4H). ^{13}C NMR (100 MHz, DMSO-d_6) δ 162.07, 154.53, 148.87, 134.82, 127.51, 126.95, 126.24, 121.80, 120.51, 111.44, 105.30, 105.05, 104.79, 60.98, 53.09, 50.83. ESI-MS: calculated for $C_{19}H_{18}F_2N_4O$ [M+H]$^+$ 356.14487, found 356.14568.

1.3 The Synthesis method of Compounds IN17-2

Procedure:

To a solution of the compound **2** (0.20 g, 1 mmol) in N,N-dimethylformamide was added 1-(2,4-difluorobenzyl)piperazine (0.25 g ,1.2 mmol) and K_2CO_3 (0.41 g, 3 mmol). The reaction mixture was refluxed at 80 ℃ for 1 h. After cooling to room temperature, the mixture was filtered through a pad of Celite and washed with ethyl acetate. The organic solvent was removed under vacuum and the residue was purified by silica-gel column chromatography to give the compound **IN17-2**. White solid, yield 58.9 %.

2-((4-(2,4-difluorobenzyl)piperazin-1-yl)methyl)quinazolin-4(3H)-one:

^{1}H NMR (400 MHz, DMSO-d_6) δ 11.90 (s, 1H), 8.11 (d, J = 7.8 Hz, 1H), 7.80 (t, J = 7.5 Hz, 1H), 7.65 (d, J = 8.1 Hz, 1H), 7.55 – 7.40 (m, 2H), 7.21 (t, J = 9.6 Hz, 1H), 7.07 (t, J = 8.1 Hz, 1H), 3.54 (s, 2H), 3.46 (s, 2H), 3.38 (s, 2H), 2.55 (s, 4H), 2.48 (s, 2H). ^{13}C NMR (100 MHz, DMSO-d_6) δ 162.01, 154.54, 148.84, 134.81, 133.23, 127.47, 126.93, 126.23, 121.78, 111.84, 111.62, 104.25, 103.86, 60.82, 54.28, 52.45. ESI-MS: calculated for $C_{20}H_{20}F_2N_4O$ [M+H]$^+$ 370.16052, found 370.15977.

1.4 The Synthesis method of Compounds IN17-3

Procedure:

To a solution of the compound **4** (0.28 g, 1 mmol) in tetrahydrofuran was added 2,4-difluorobenzoyl chloride (0.25 g ,1.2 mmol) and triethylamine (0.5 mL, 5 mmol). The reaction mixture was stirred at room temperature for 4 h. Then the mixture was filtered through a pad of Celite and washed with ethyl acetate. The organic solvent was removed under vacuum and the residue was purified by silica-gel column chromatography to give the compound **IN17-3**. White solid, yield 61.2 %.

2-((4-(2,4-difluorobenzoyl)piperazin-1-yl)methyl)quinazolin-4(3H)-one:

^{1}H NMR (400 MHz, DMSO-d_6) δ 12.01 (s, 1H), 8.11 (d, J = 7.9 Hz, 1H), 7.80 (t, J =

7.5 Hz, 1H), 7.64 (d, J = 8.1 Hz, 1H), 7.50 (dd, J = 12.8, 6.7 Hz, 2H), 7.37 (t, J = 9.8 Hz, 1H), 7.18 (t, J = 8.5 Hz, 1H), 3.68 (s, 2H), 3.50 (s, 2H), 3.35 (s, 2H), 3.26 (s, 2H), 2.59 (s, 2H). ^{13}C NMR (100 MHz, DMSO-d$_6$) δ 163.61, 154.41, 134.85, 130.95, 127.54, 127.00, 126.24, 121.82, 112.84, 104.90, 60.71, 53.16, 52.59, 46.99, 41.85. ESI-MS: calculated for $C_{20}H_{18}F_2N_4O_2$ $[M+H]^+$ 384.13978, found 384.14013.

1.5 The Synthesis method of Compounds IN17-4

Procedure:

To a solution of the compound **4** (0.28 g, 1 mmol) in tetrahydrofuran was added 2,4-difluorobenzenesulfonyl chloride (0.21 g ,1.2 mmol) and triethylamine (0.5 mL, 5 mmol). The reaction mixture was stirred at room temperature for 4 h. Then the mixture was filtered through a pad of Celite and washed with ethyl acetate. The organic solvent was removed under vacuum and the residue was purified by silica-gel column chromatography to give the compound **IN17-4**. White solid, yield 53.4 %.

2-((4-((2,4-difluorophenyl)sulfonyl)piperazin-1-yl)methyl)quinazolin-4(3H)-one:

^{1}H NMR (400 MHz, DMSO-d$_6$) δ 11.87 (s, 1H), 8.09 (d, J = 7.8 Hz, 1H), 7.89 – 7.76 (m, 2H), 7.63 (t, J = 7.0 Hz, 2H), 7.49 (t, J = 7.4 Hz, 1H), 7.35 (t, J = 8.3 Hz, 1H), 3.49 (s, 2H), 3.09 (s, 4H), 2.60 (s, 4H). ^{13}C NMR (100 MHz, DMSO-d$_6$) δ 162.01, 158.51, 154.42, 148.83, 134.83, 133.65, 127.49, 126.96, 126.23, 121.82, 113.09, 107.40, 106.99, 106.59, 60.31, 52.00, 45.92. ESI-MS: calculated for $C_{19}H_{18}F_2N_4O_3S$ $[M+H]^+$ 420.10677, found 420.10745.

1.6 The Synthesis method of Compounds IN17-5

Procedure:

To a solution of the compound **4** (0.28 g, 1 mmol) in tetrahydrofuran was added 2,4-difluoro-1-(sulfinylamino)benzene (0.35 g , 2 mmol) and triethylamine (0.5 mL, 5 mmol). The reaction mixture was stirred at room temperature for 4 h. Then the mixture was filtered through a pad of Celite and washed with ethyl acetate. The organic solvent was removed under vacuum and the residue was purified by silica-gel column chromatography to give the compound **IN17-5**. White solid, yield 55.9 %.

N-(2,4-difluorophenyl)-4-((4-oxo-3,4-dihydroquinazolin-2-yl)methyl)piperazine-1-carbothioamide:

^{1}H NMR (400 MHz, DMSO-d_6) δ 12.02 (s, 1H), 9.14 (s, 1H), 8.13 (d, J = 7.8 Hz, 1H), 7.81 (t, J = 7.5 Hz, 1H), 7.67 (d, J = 8.1 Hz, 1H), 7.52 (t, J = 7.3 Hz, 1H), 7.28 (d, J = 8.8 Hz, 2H), 7.06 (d, J = 9.0 Hz, 1H), 3.97 (s, 4H), 3.55 (s, 2H), 2.62 (s, 4H). ^{13}C NMR (100 MHz, DMSO-d_6) δ 182.45, 162.09, 159.27, 156.80, 154.43, 148.90, 134.84, 131.79, 127.52, 126.97, 126.27, 121.85, 111.47, 104.58, 60.55, 52.63, 48.37, 47.06. ESI-MS: calculated for $C_{20}H_{19}F_2N_5OS$ $[M+H]^+$ 415.12784, found 415.12662.

S4: ^{1}H-NMR and ^{13}C-NMR Spectral of synthetic compounds

However, in the hydrogen spectrum, when the deuterated solvent is chloroform, the displacement of the hydrogen atoms on the NH in the skeleton of the compounds are at 9.9 and 10.2, and the NH on the urea are at 6.7 and 7.1, but due to the different substituent groups, it can easily overlap with the other hydrogen atoms of the benzene ring and a cleavage occurs. Meanwhile, the hydrogen displacement of the piperazine ring in the A-series compounds are at 2.70 and 3.61, and the methylene group attached to the skeleton acts at 3.65, which is also influenced by the substituent group, and the top of the methylene group overlaps with the hydrogen on the piperazine. When the deuterated solvent is DMSO, the displacement of the hydrogen atoms on the NH in the skeleton of the compounds is are at 11.0 and 12.0, and the NH on the urea are at 8.5 and 8.6, but due to the different substituent groups, it can easily overlap with the other hydrogen atoms of the benzene ring and a cleavage occurs. Meanwhile, the hydrogen displacement of the piperazine ring in the A-series compounds are at 2.54 and 3.48, and the methylene group attached to the skeleton acts at 3.50, which is also influenced by the substituent group, and the top of the methylene group overlaps with the hydrogen on the piperazine.

S4: ^{1}H-NMR and ^{13}C-NMR Spectral of synthetic compounds

1.1 ^{1}H-NMR and ^{13}C-NMR ($CDCl_3$) spectrum of A1

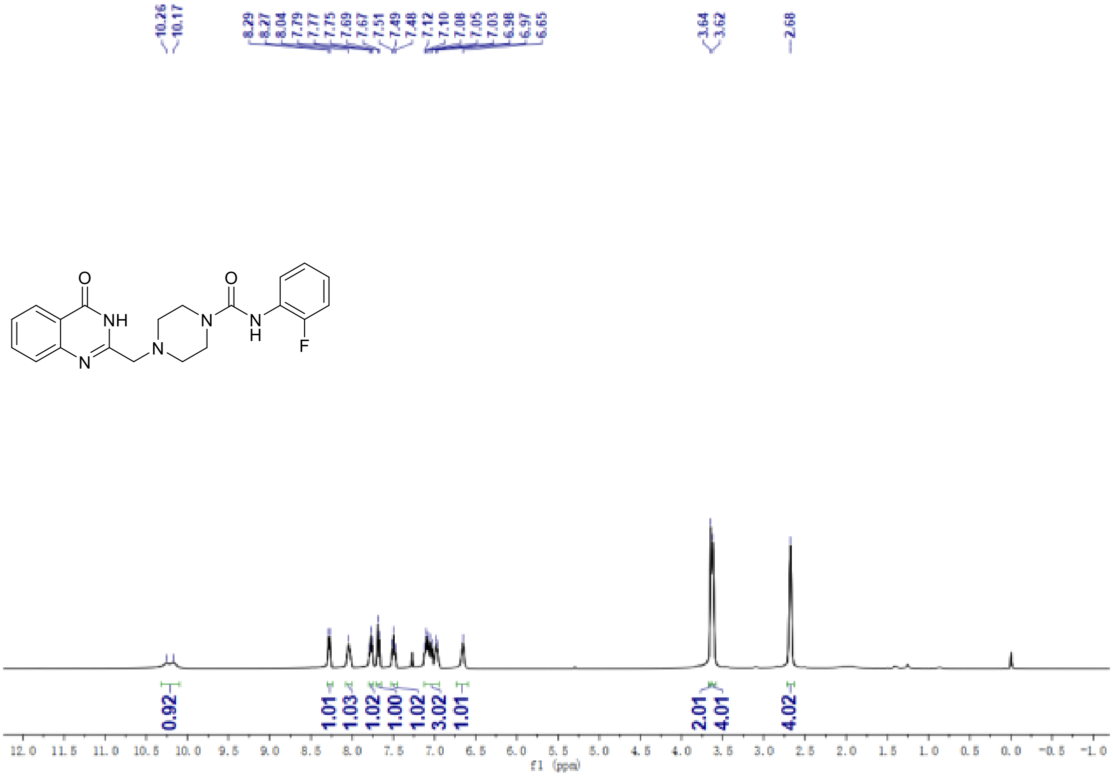

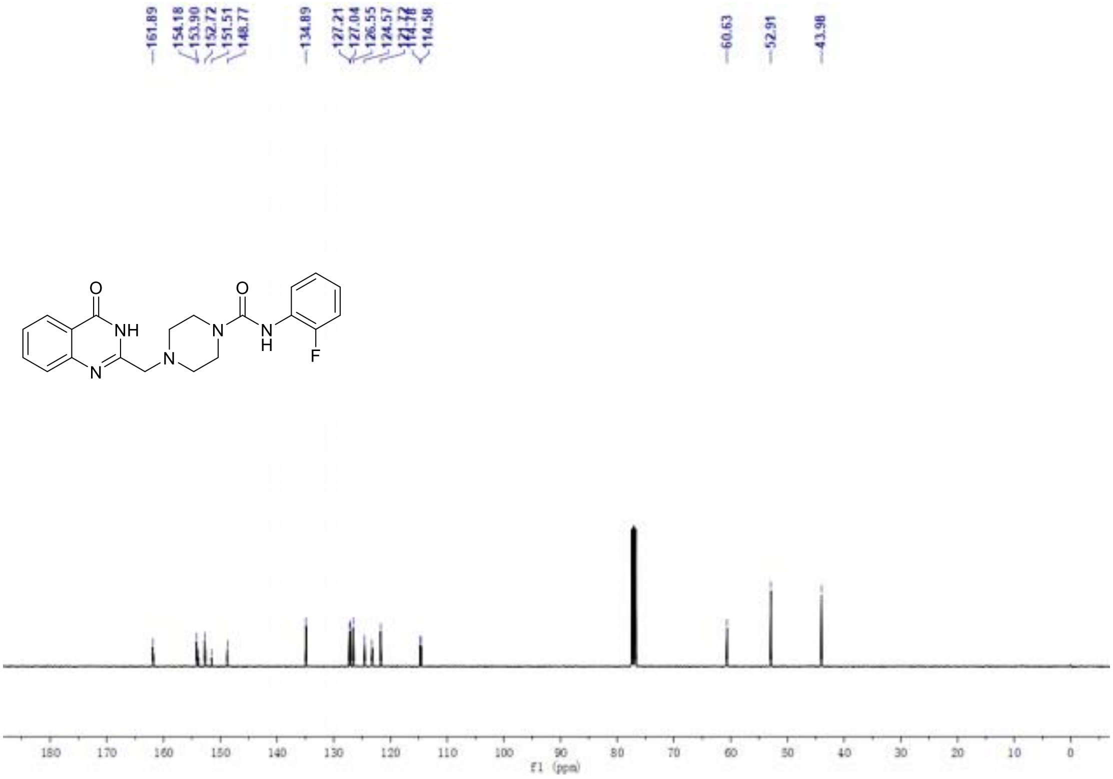

1.2 ^{1}H-NMR and ^{13}C-NMR (DMSO-d$_6$) spectrum of A2

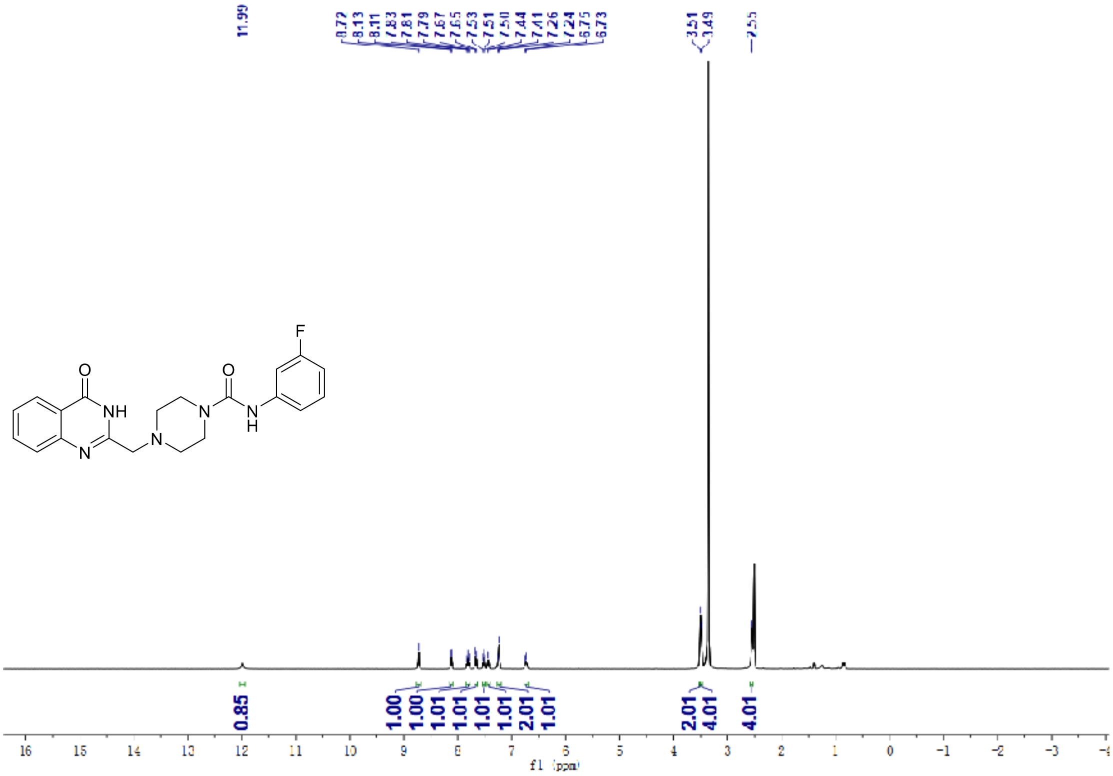

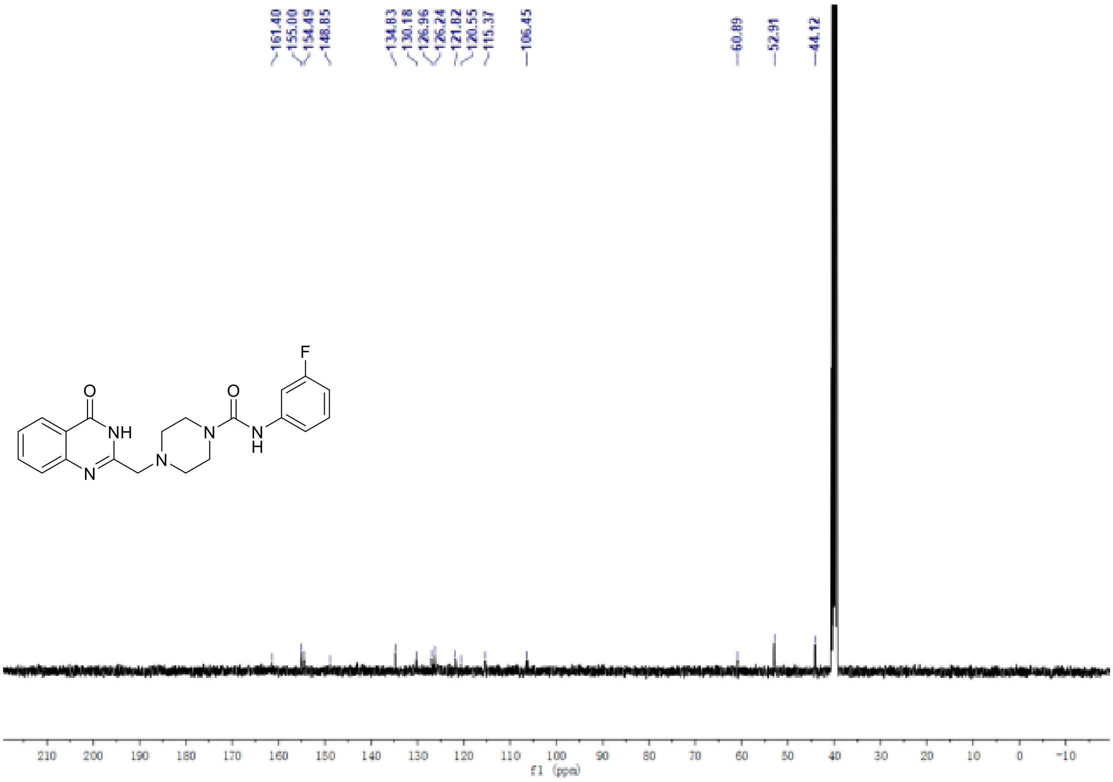

1.3 ^{1}H-NMR and ^{13}C-NMR (DMSO-d$_6$) spectrum of A3

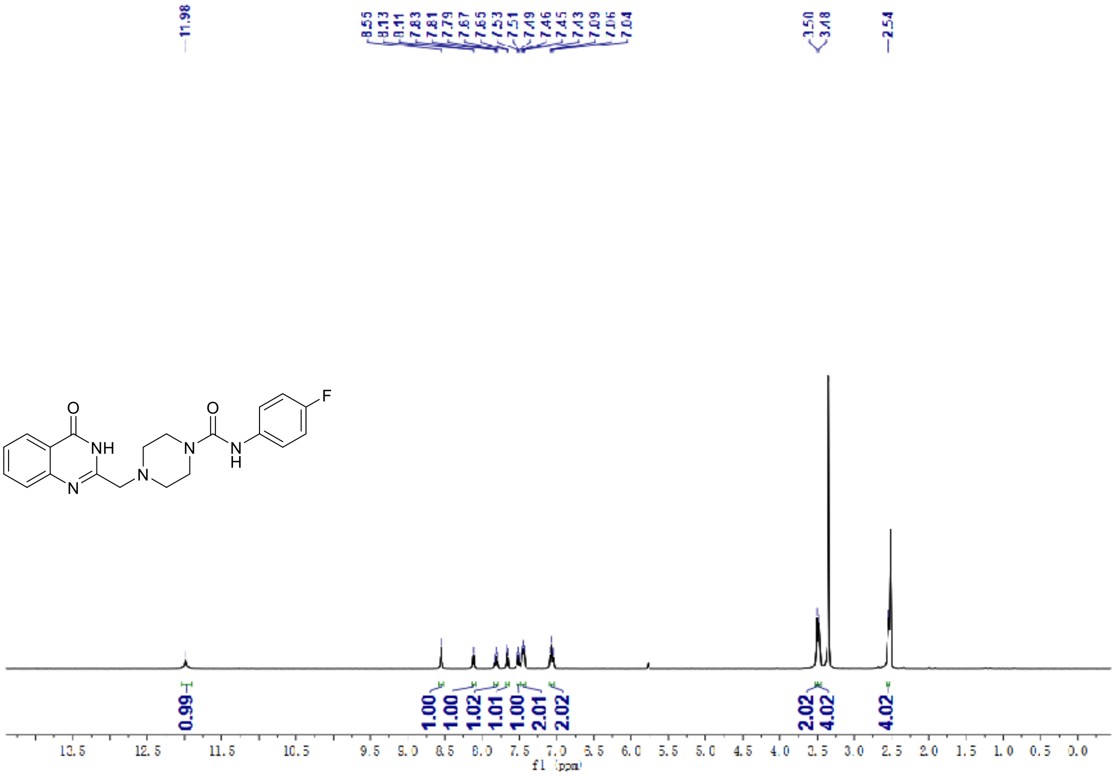

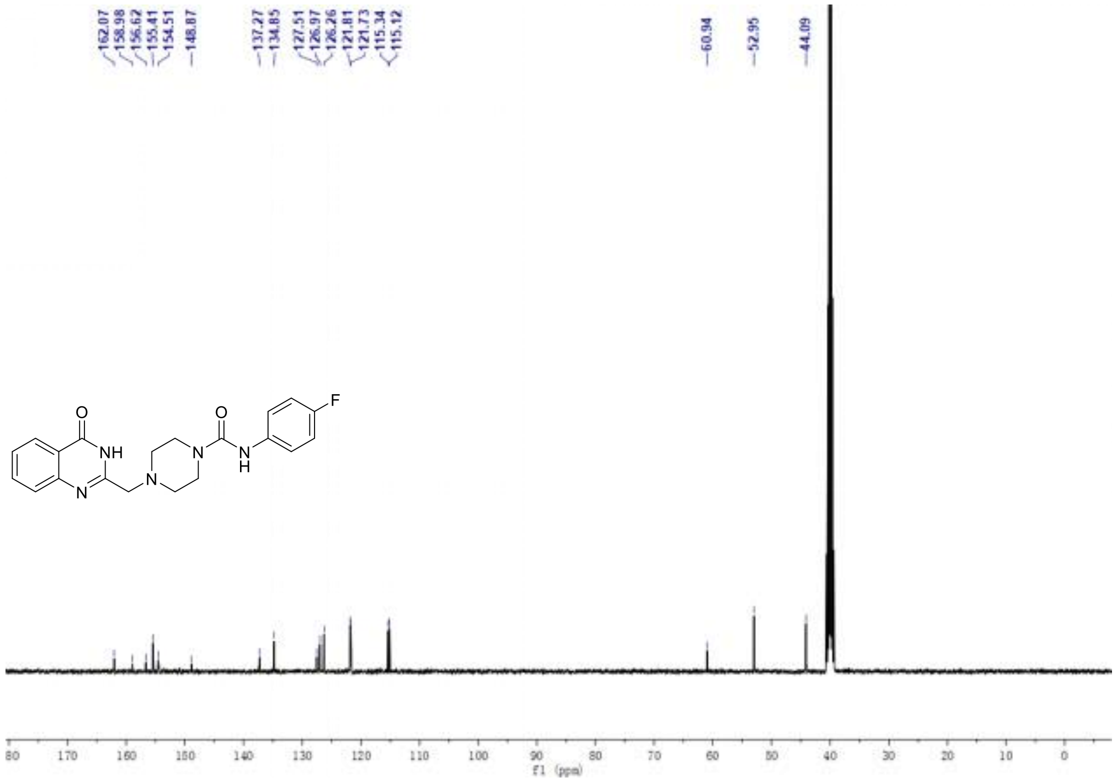

1.4 ^{1}H-NMR and ^{13}C-NMR ($CDCl_3$) spectrum of A4

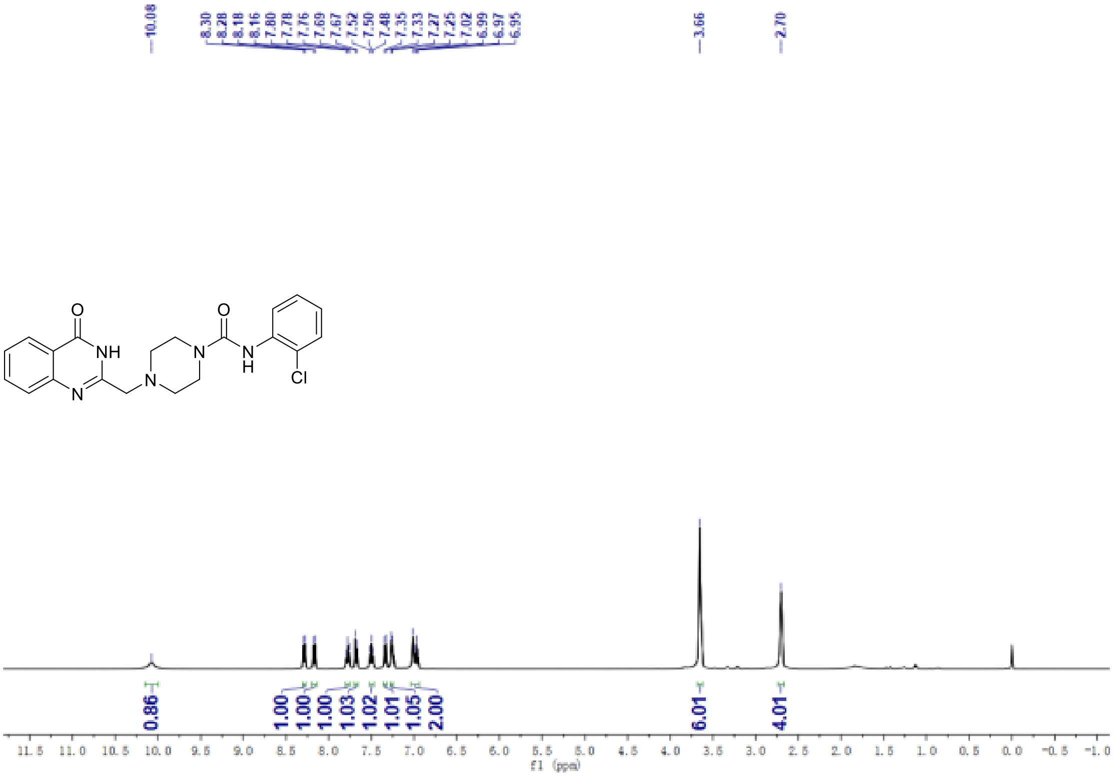

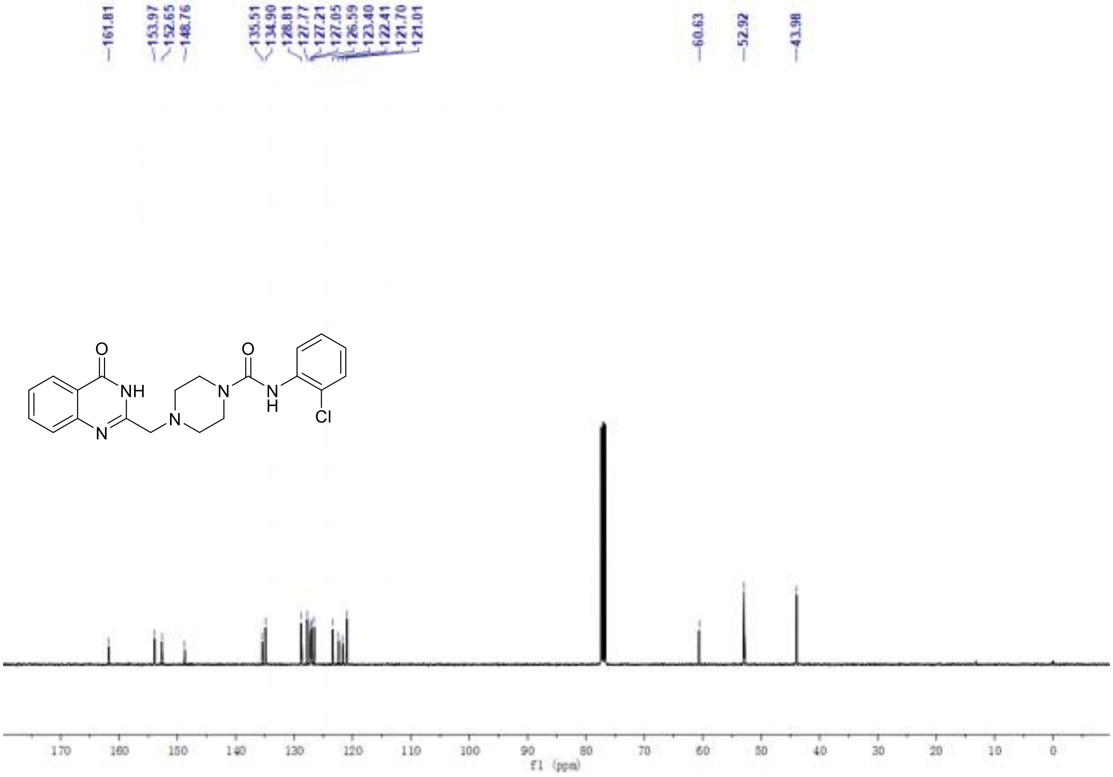

1.5 ^{1}H-NMR and ^{13}C-NMR (DMSO-d_6) spectrum of A5

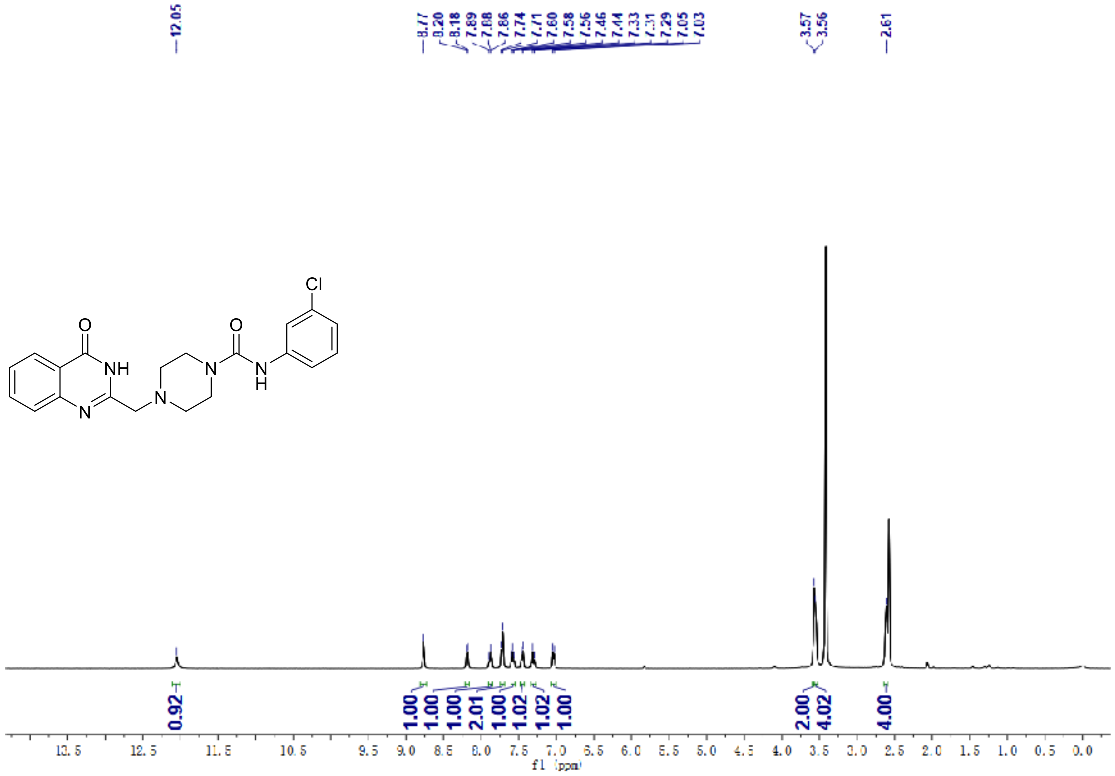

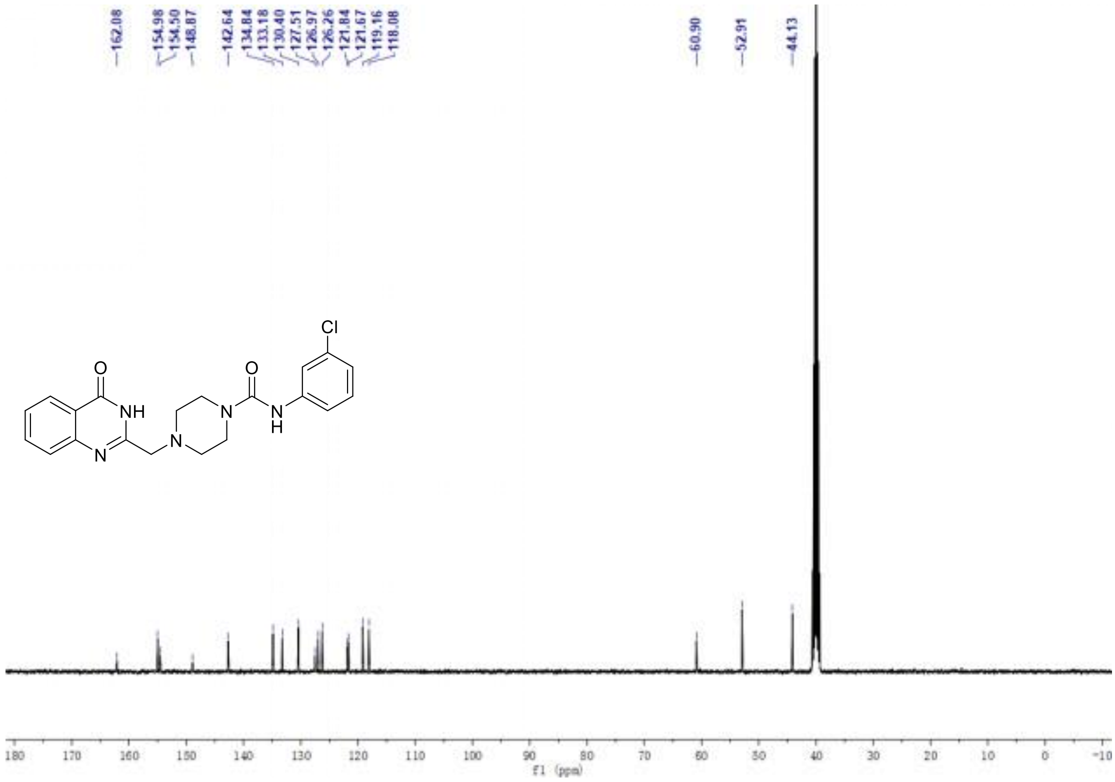

1.6 ^{1}H-NMR and ^{13}C-NMR (DMSO-d_6) spectrum of A6

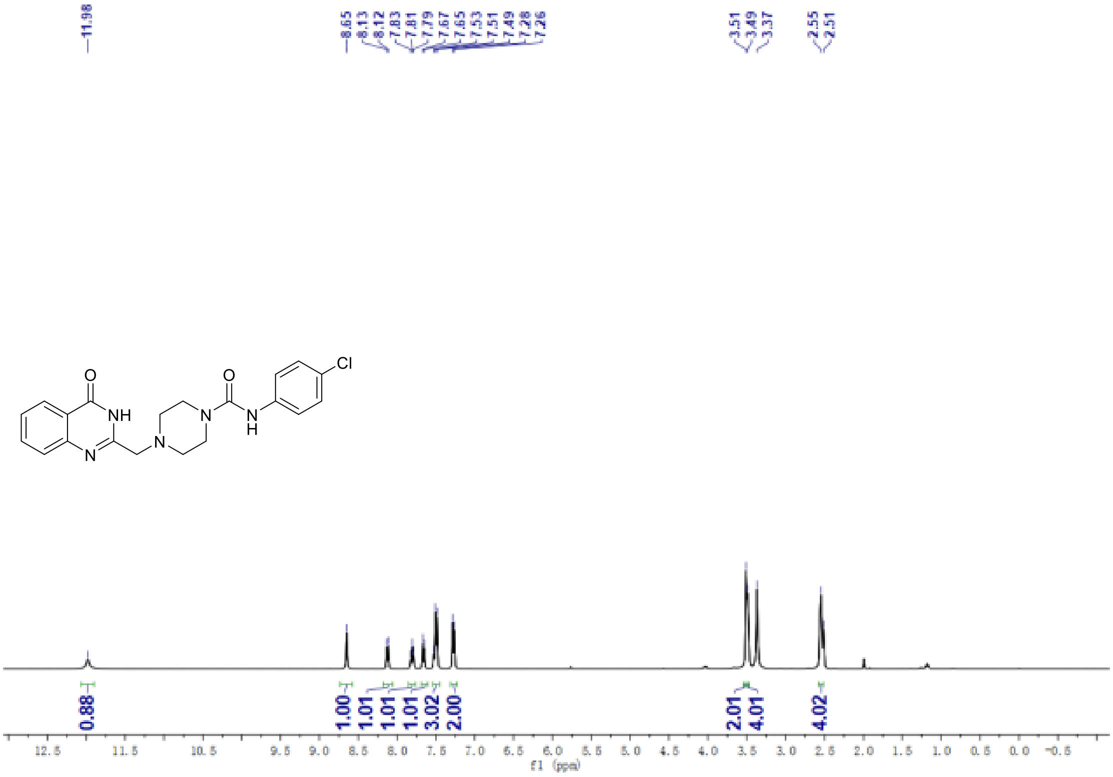

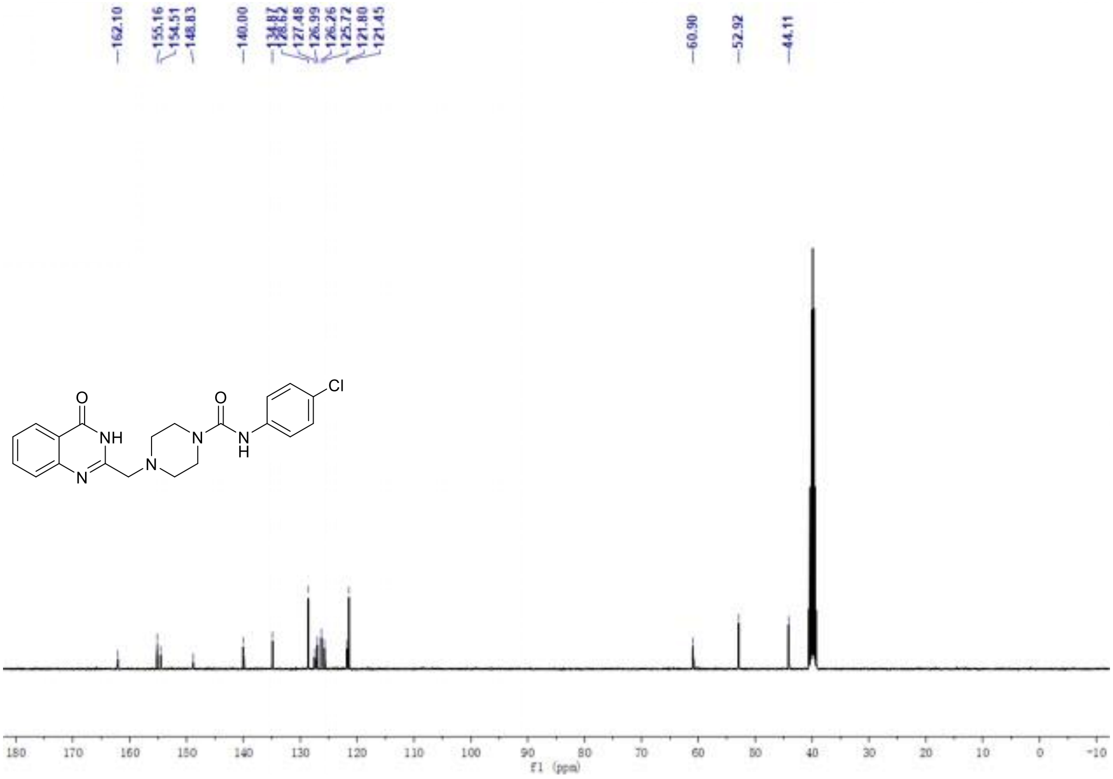

1.7 ^{1}H-NMR and ^{13}C-NMR ($CDCl_3$) spectrum of A7

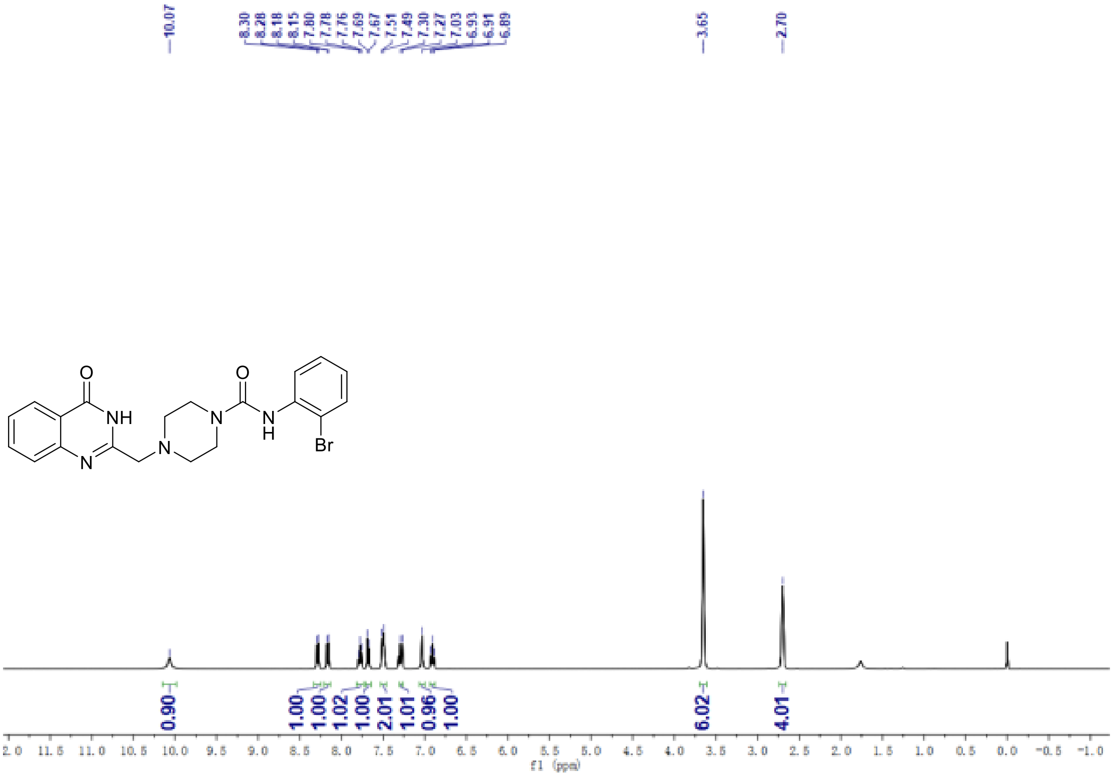

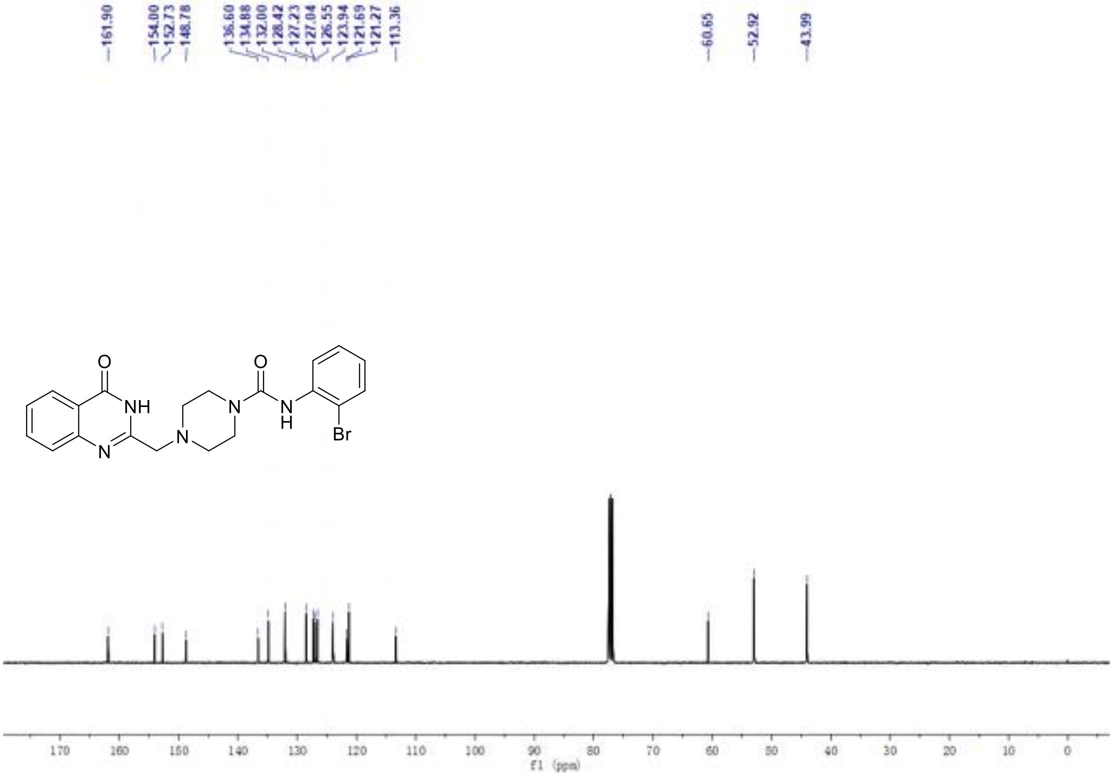

1.8 ^{1}H-NMR and ^{13}C-NMR (DMSO-d_6) spectrum of A8

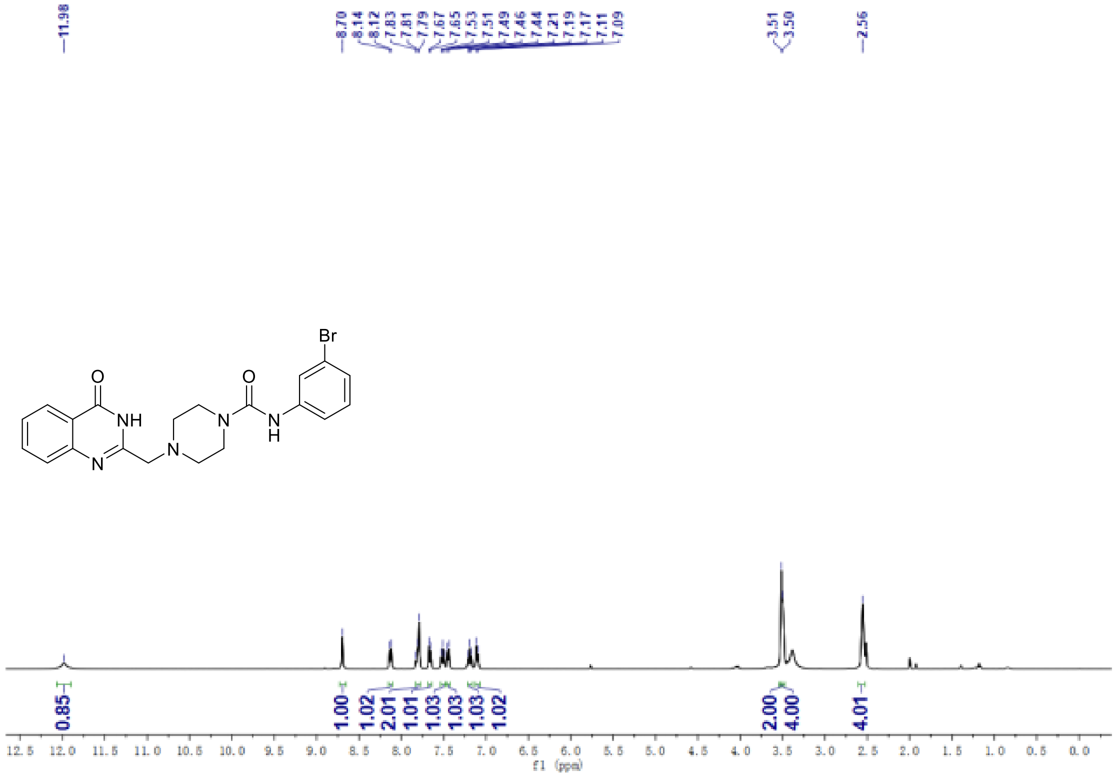

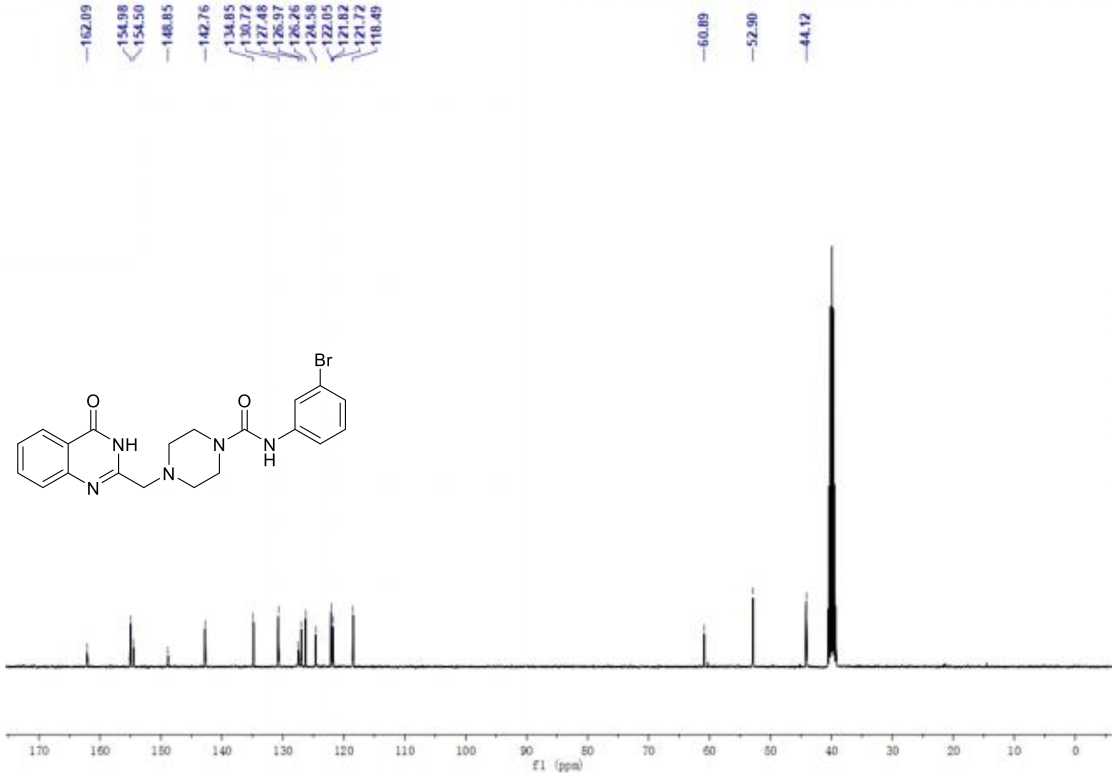

1.9 ^{1}H-NMR and ^{13}C-NMR (DMSO-d_6) spectrum of A9

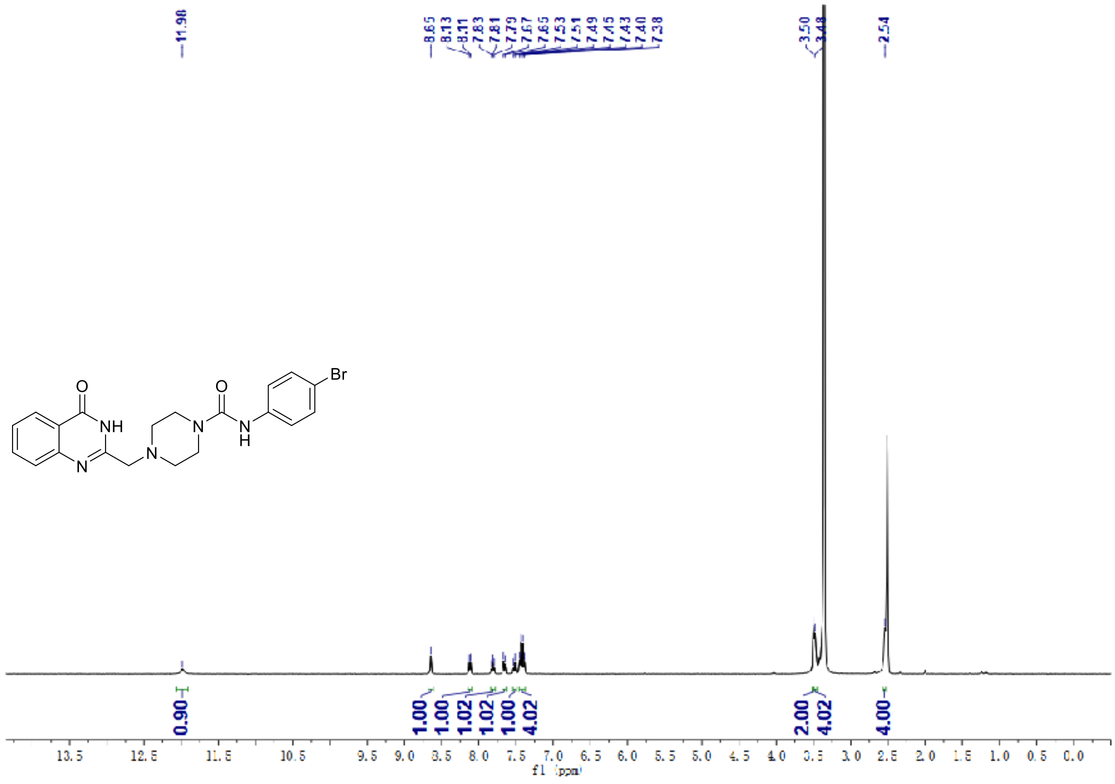

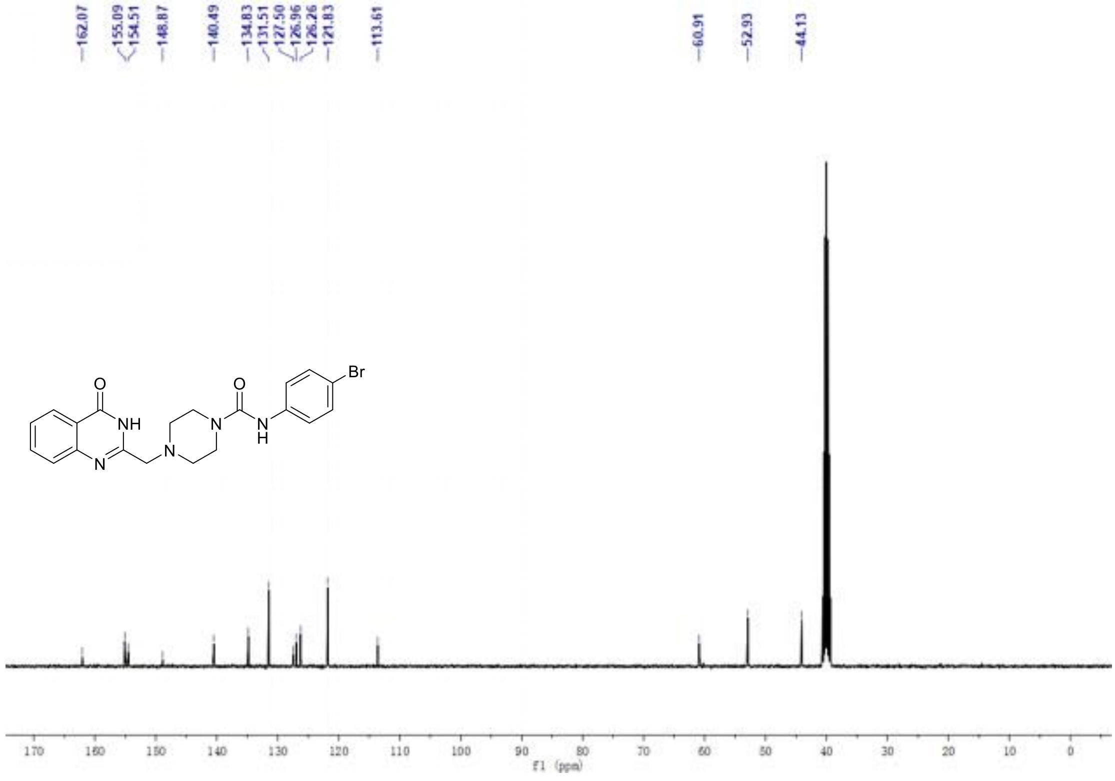

1.10 ^{1}H-NMR and ^{13}C-NMR ($CDCl_3$) spectrum of A10

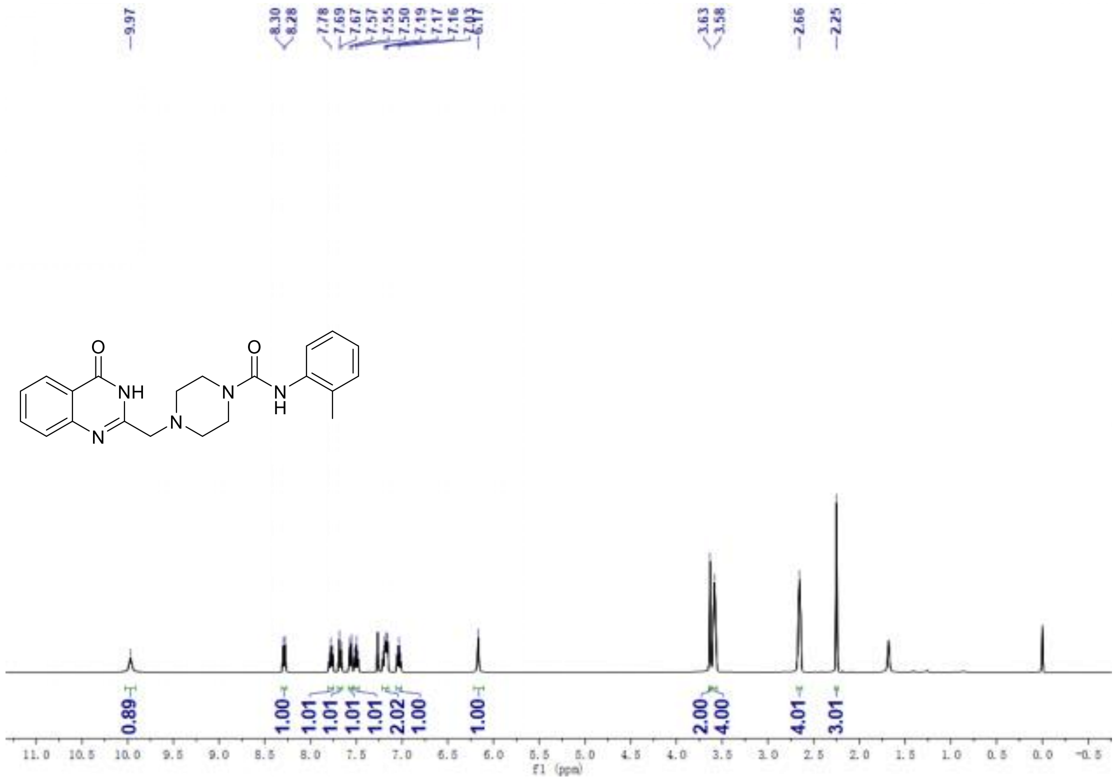

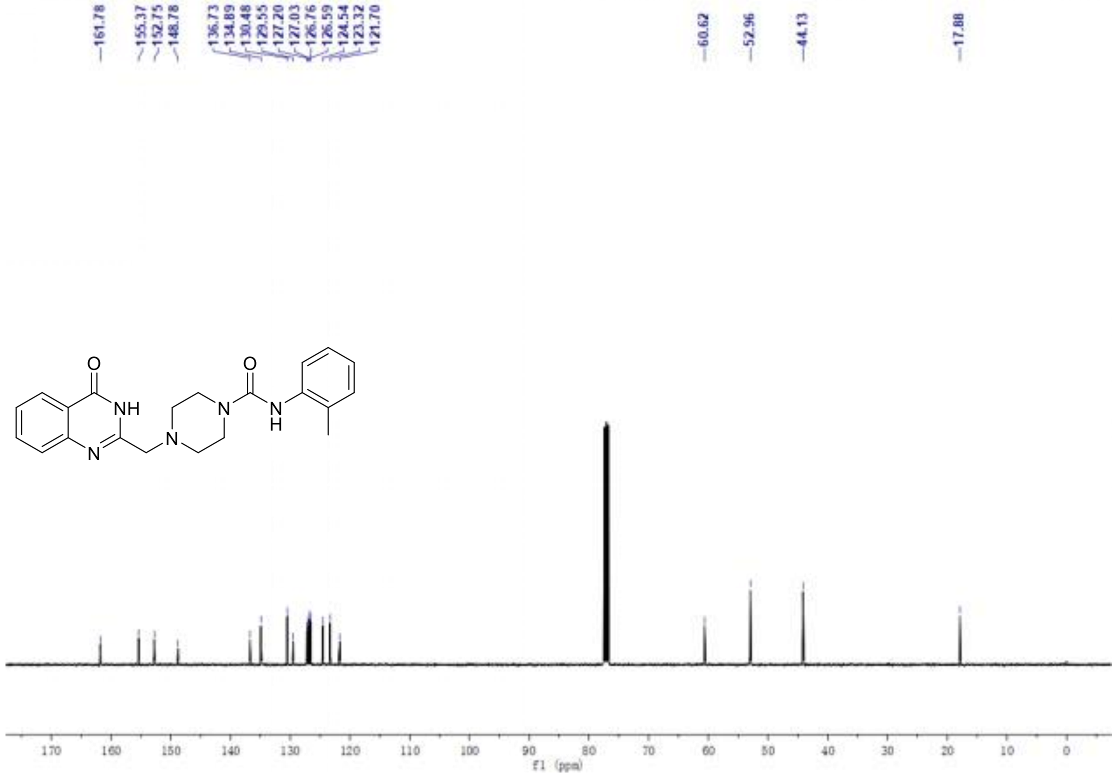

1.11 ^{1}H-NMR and ^{13}C-NMR (CDCl$_3$) spectrum of A11

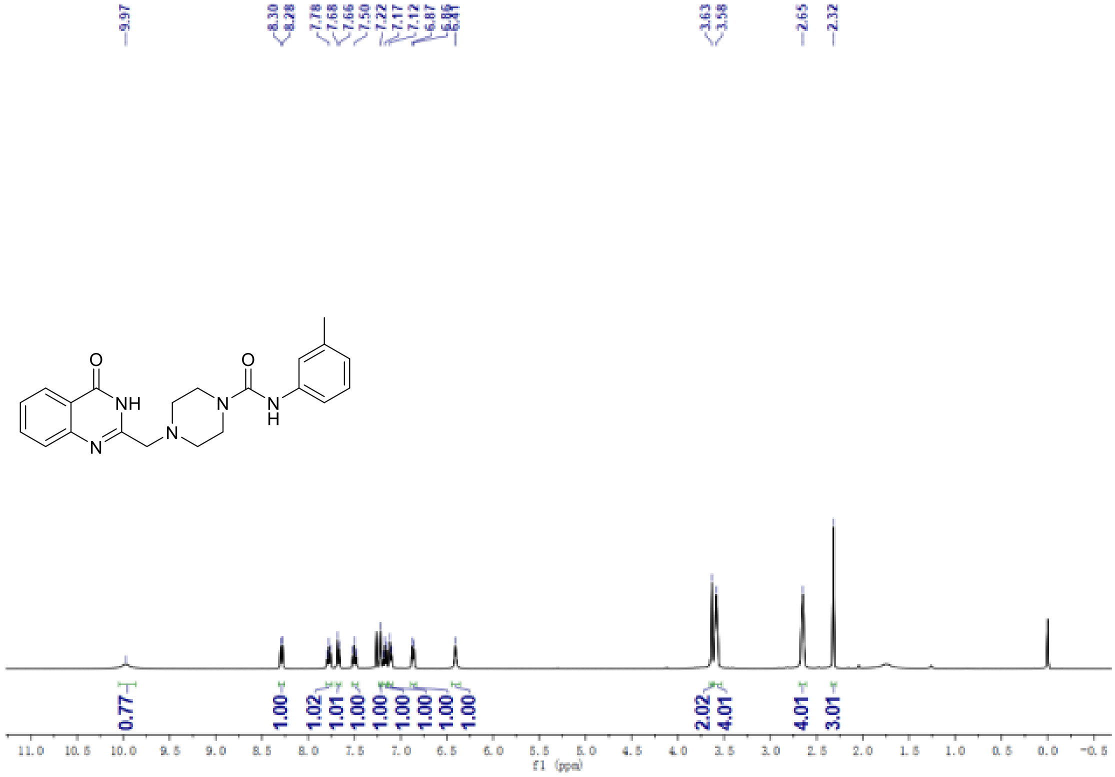

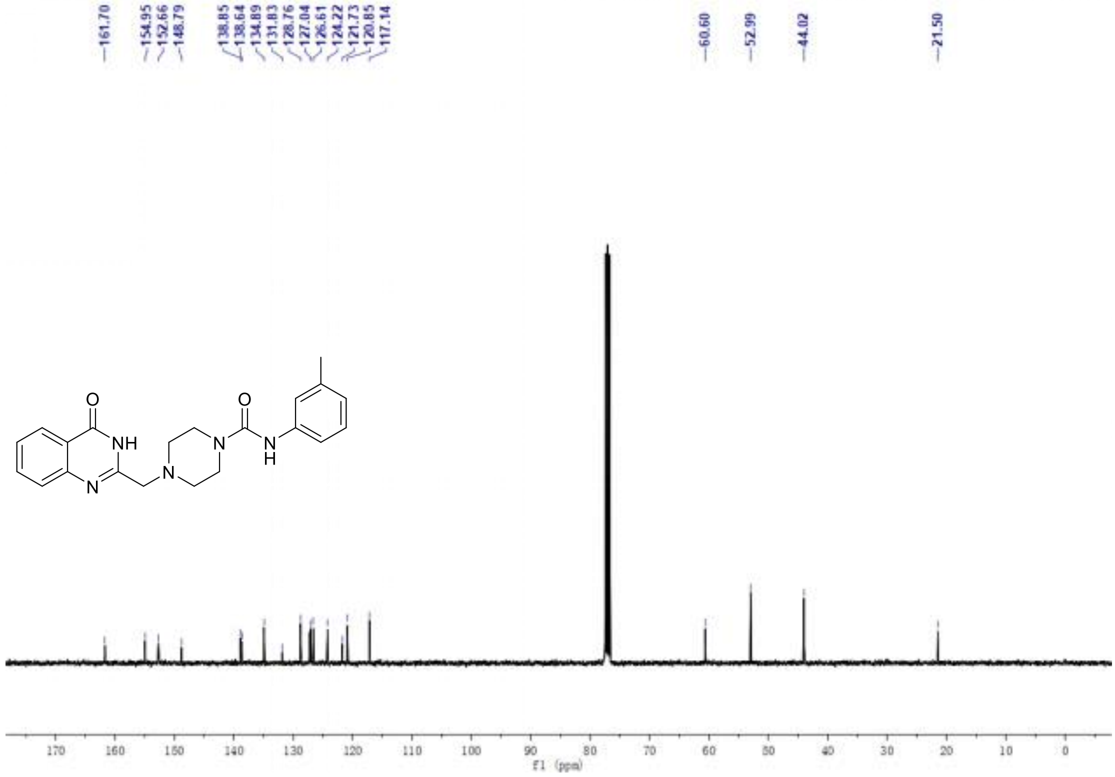

1.12 ^{1}H-NMR and ^{13}C-NMR (DMSO-d_6) spectrum of A12

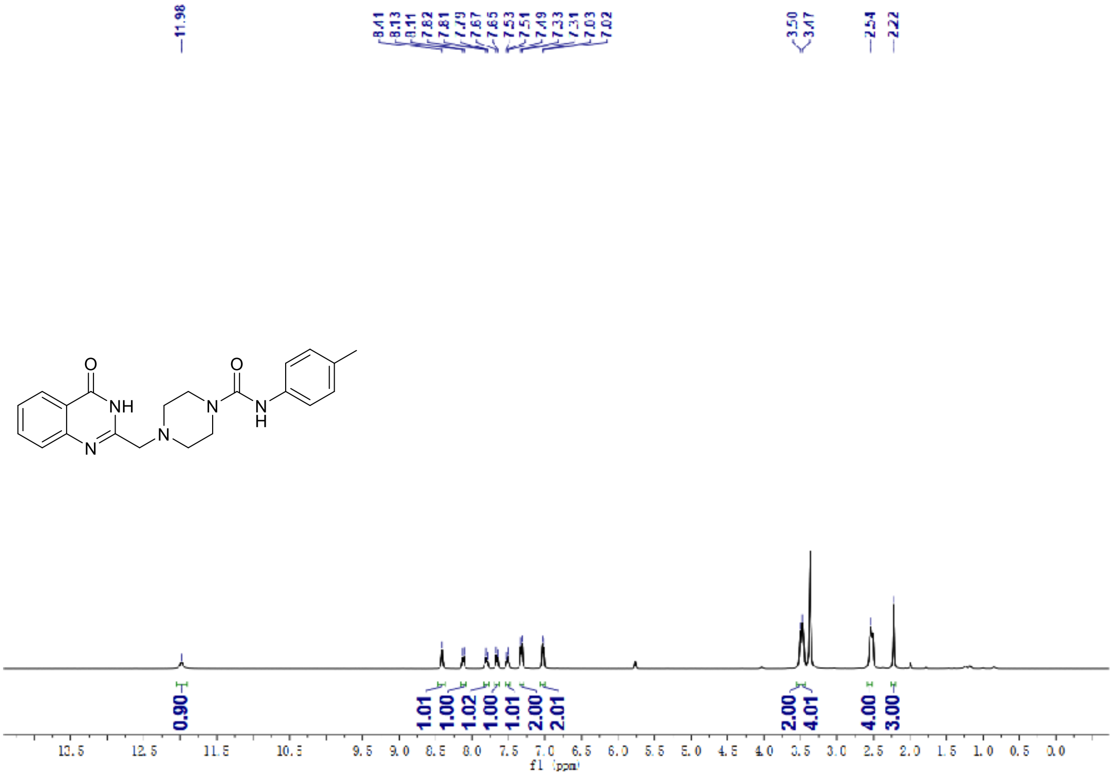

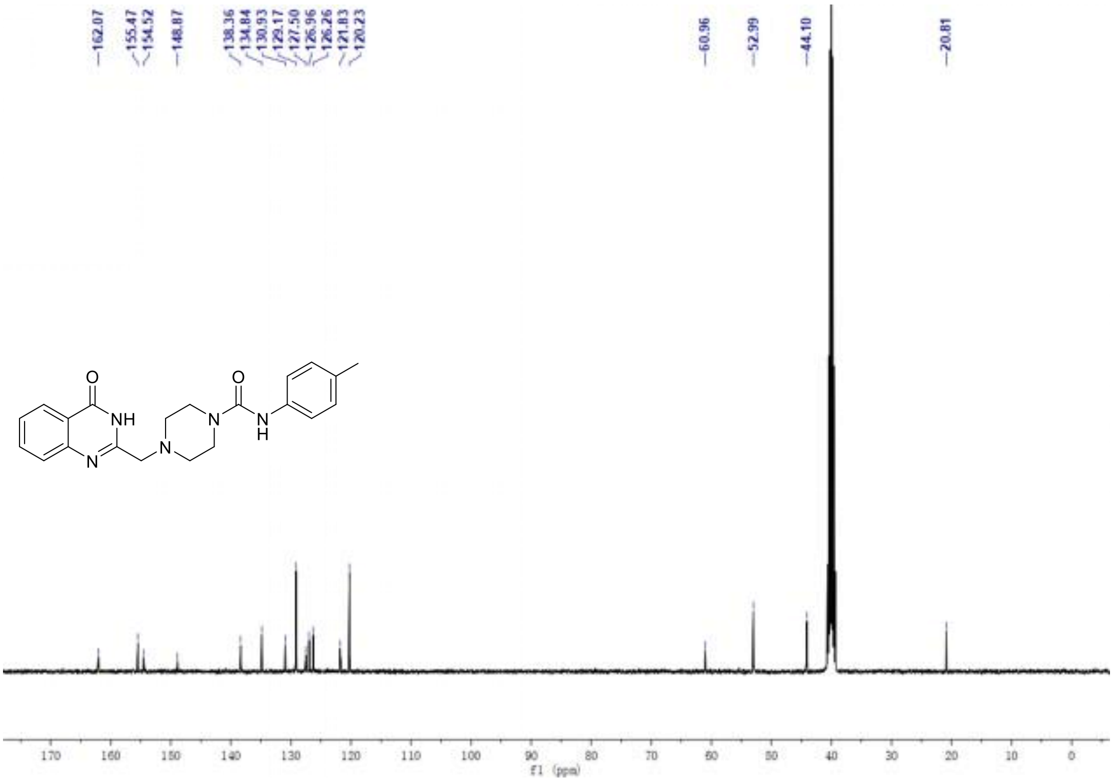

1.13 ^{1}H-NMR and ^{13}C-NMR (DMSO-d$_6$) spectrum of A13

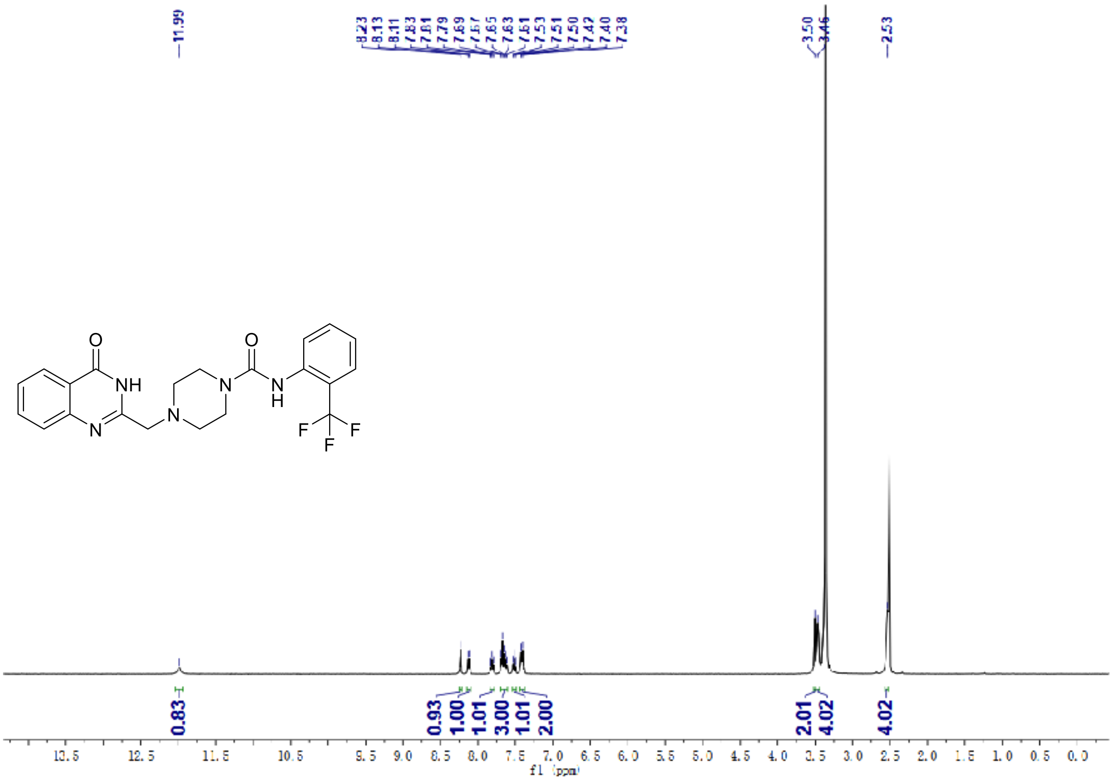

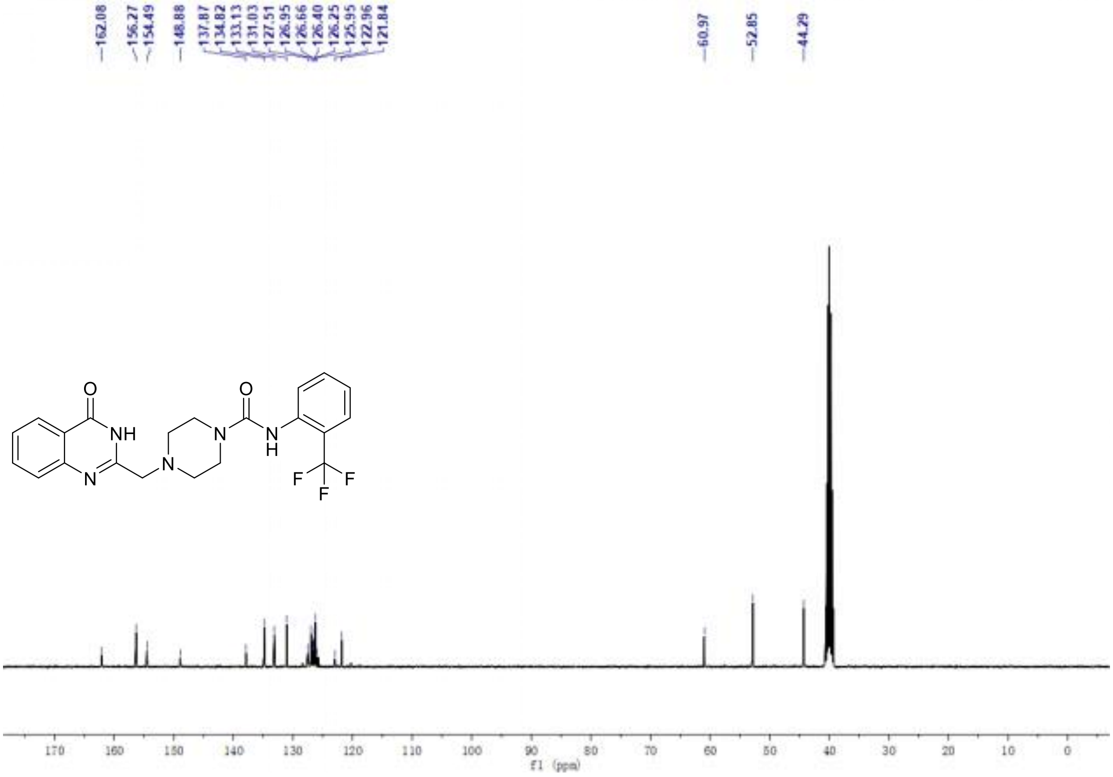

1.14 ^{1}H-NMR and ^{13}C-NMR (DMSO-d$_6$) spectrum of A14

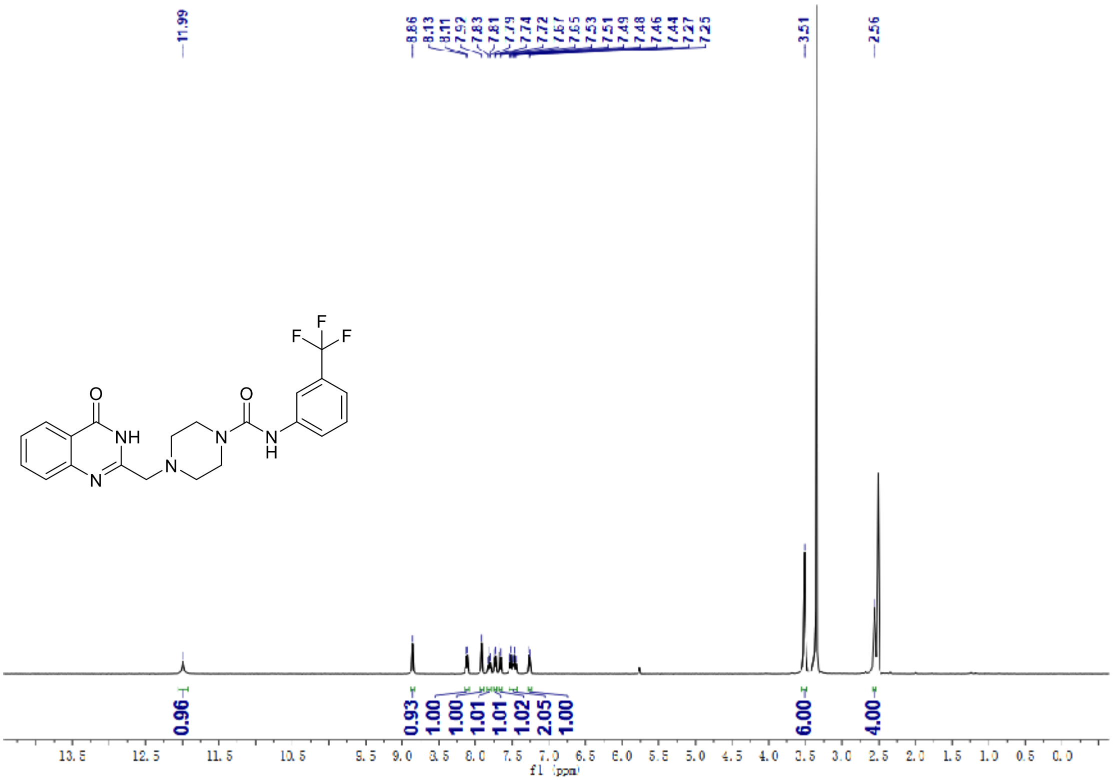

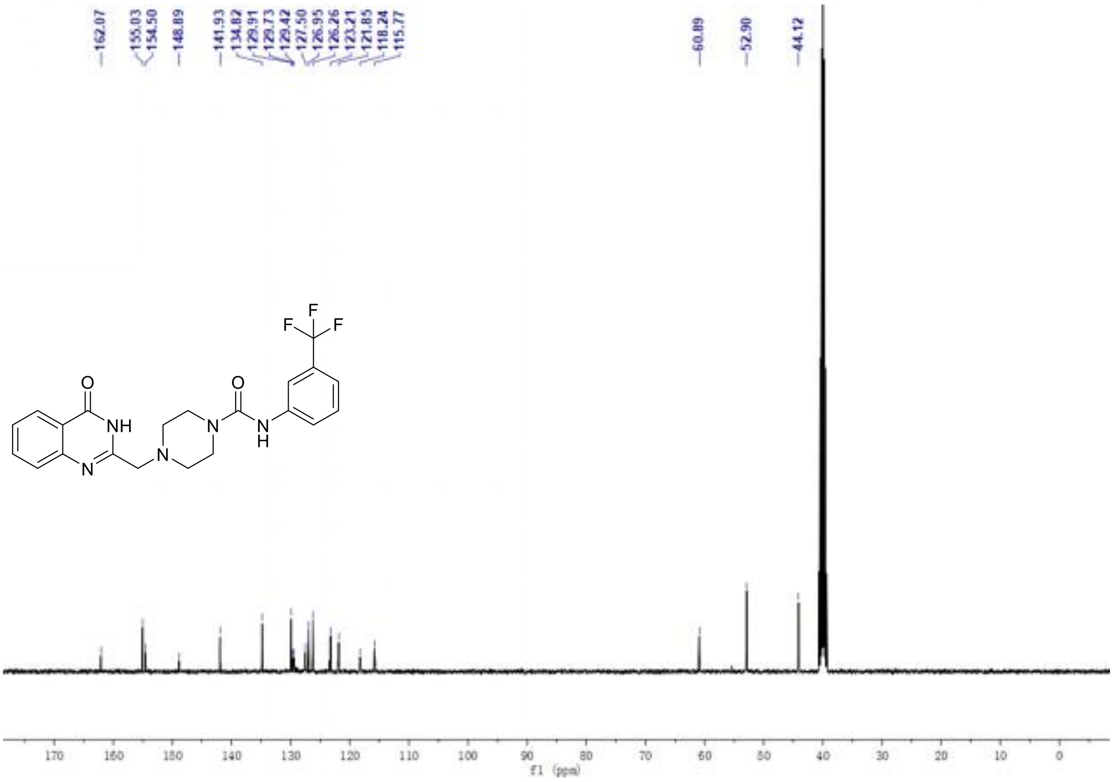

1.15 ^{1}H-NMR and ^{13}C-NMR (DMSO-d_6) spectrum of A15

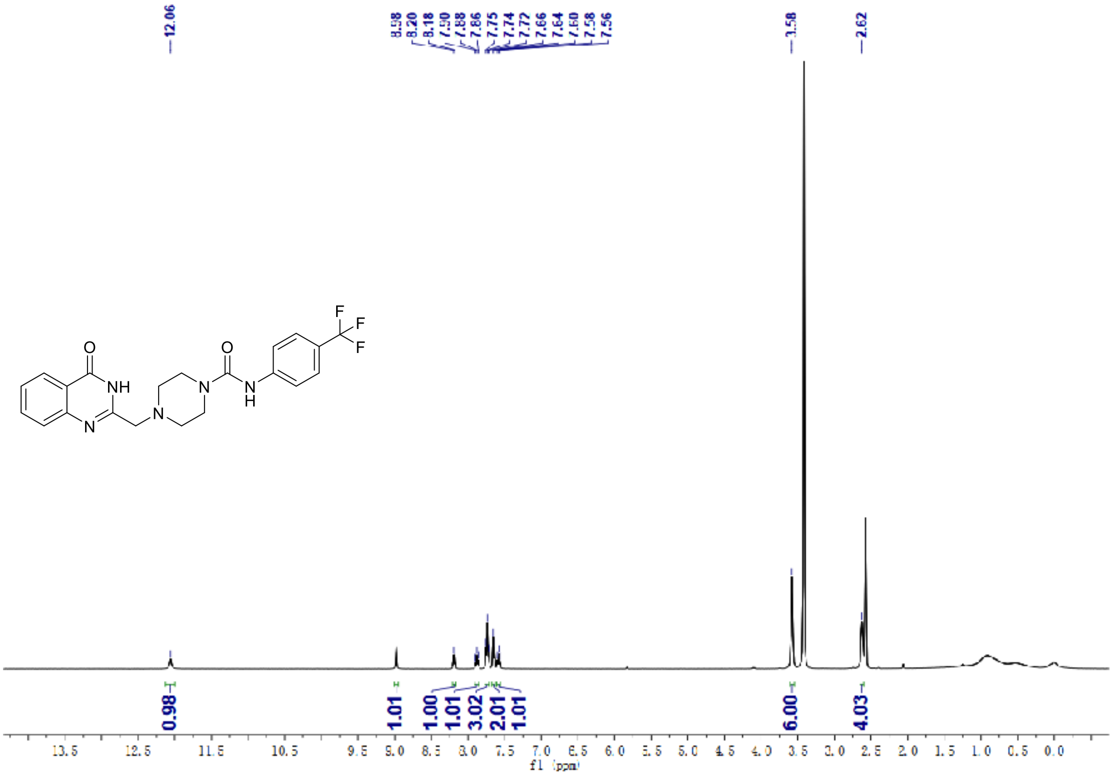

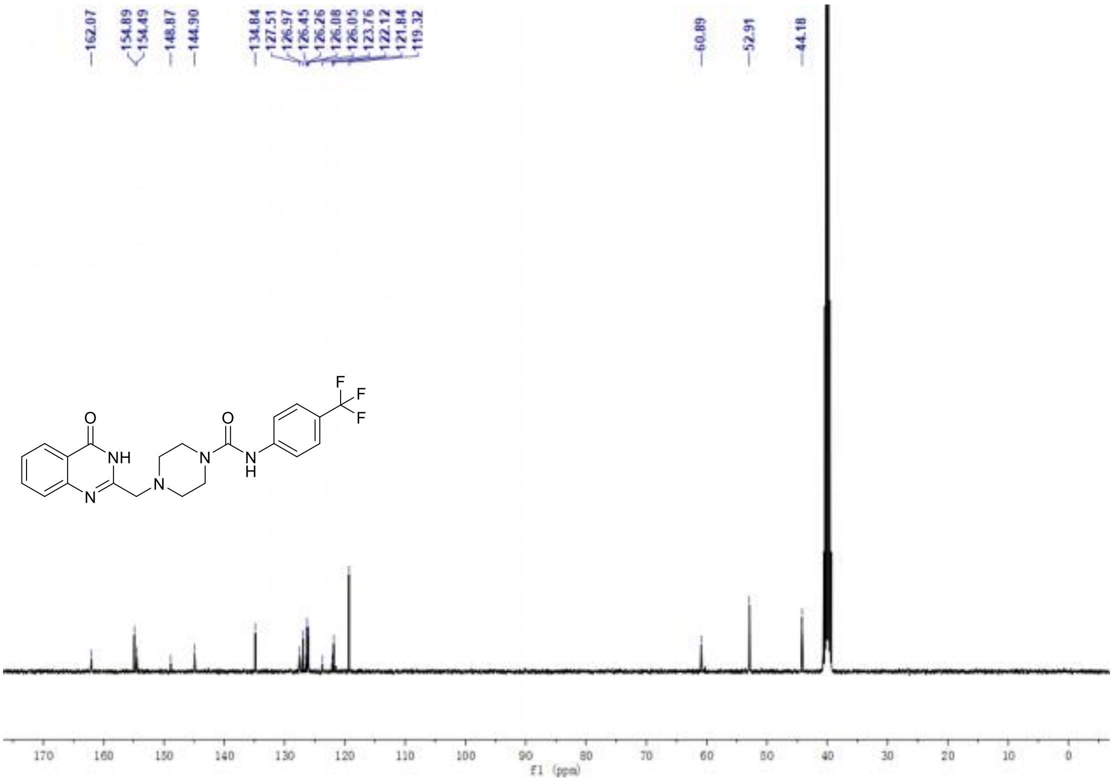

1.16 ^{1}H-NMR and ^{13}C-NMR (CDCl$_3$) spectrum of A16

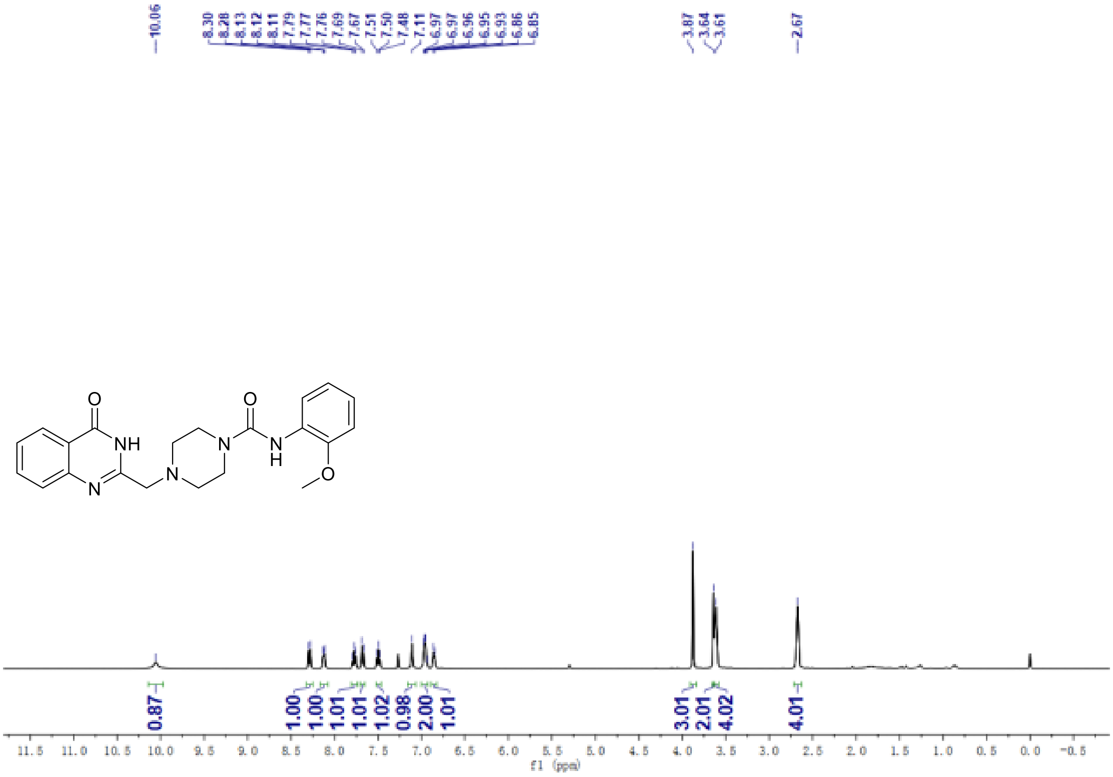

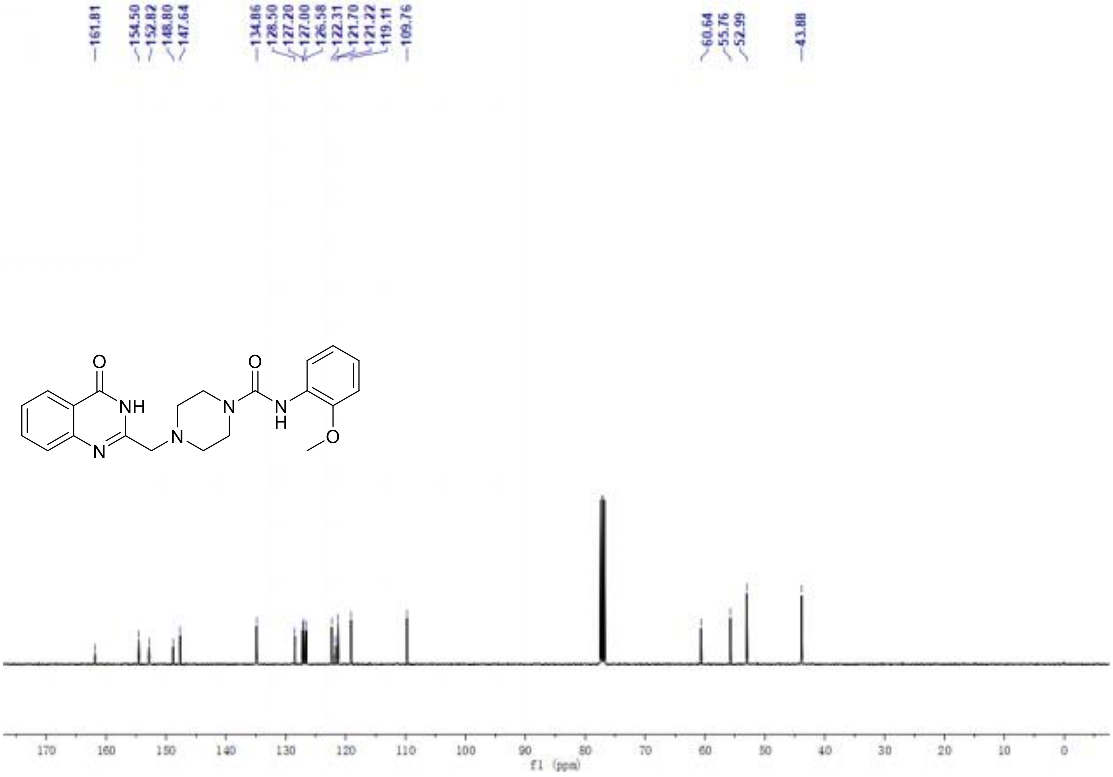

1.17 ^{1}H-NMR and ^{13}C-NMR ($CDCl_3$) spectrum of A17

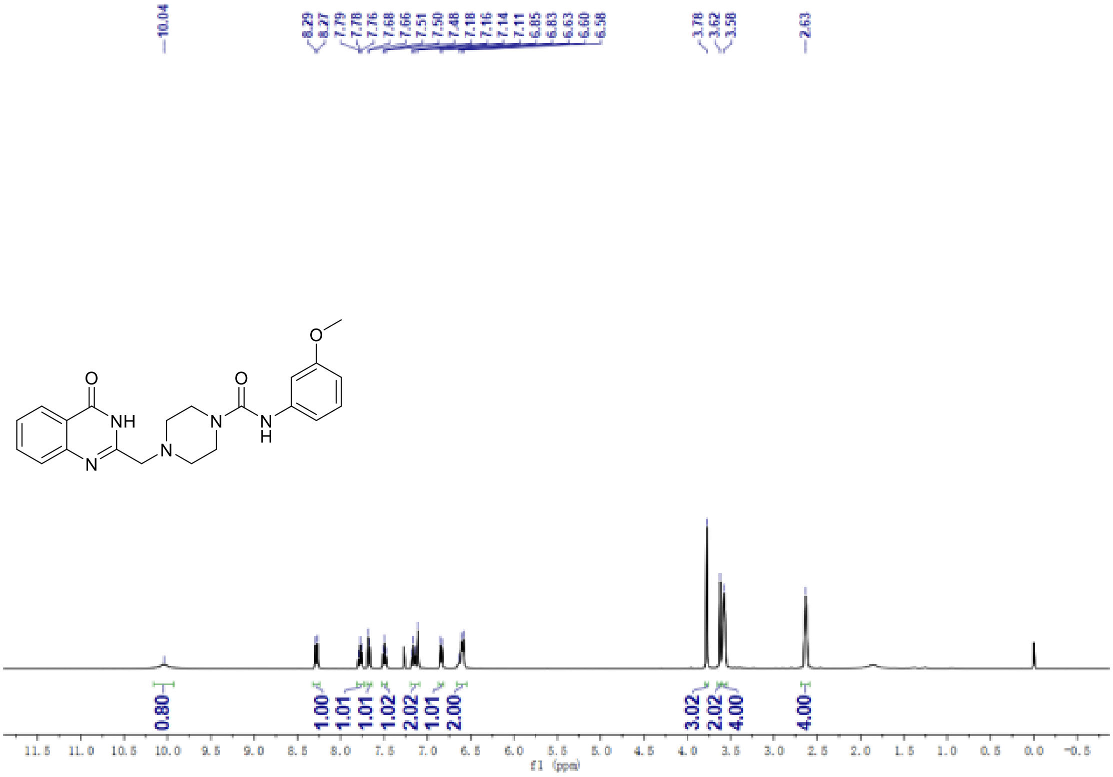

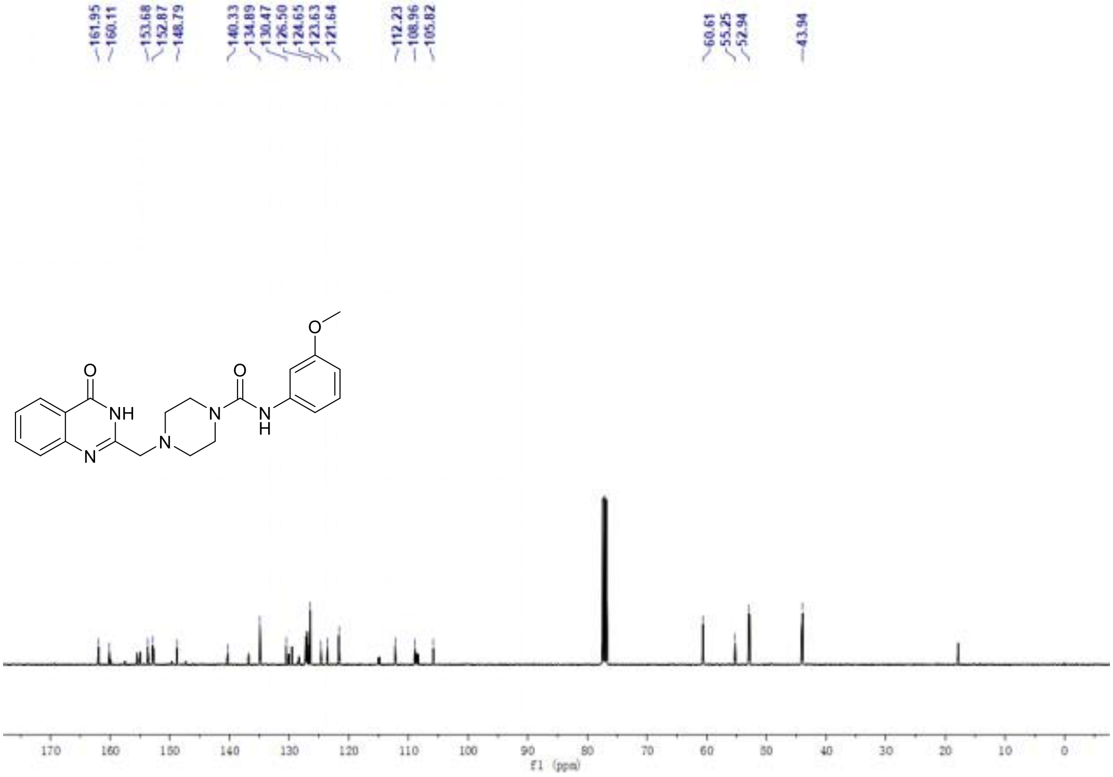

1.18 ^{1}H-NMR and ^{13}C-NMR (CDCl$_3$) spectrum of A18

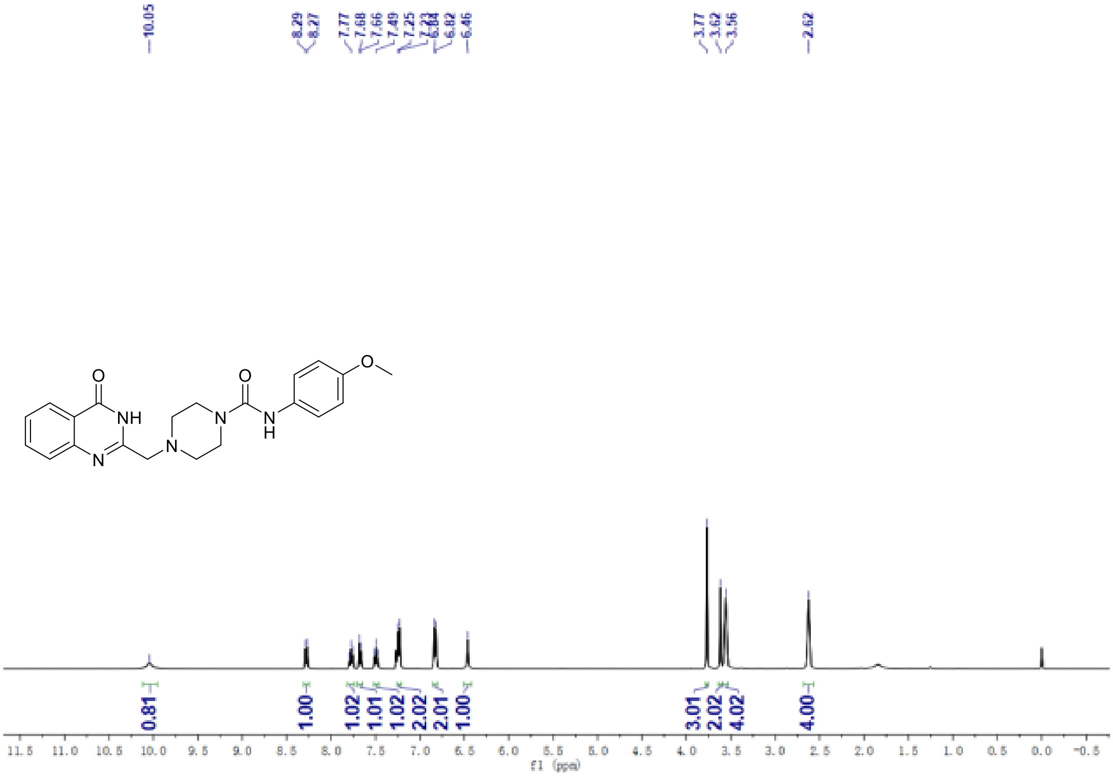

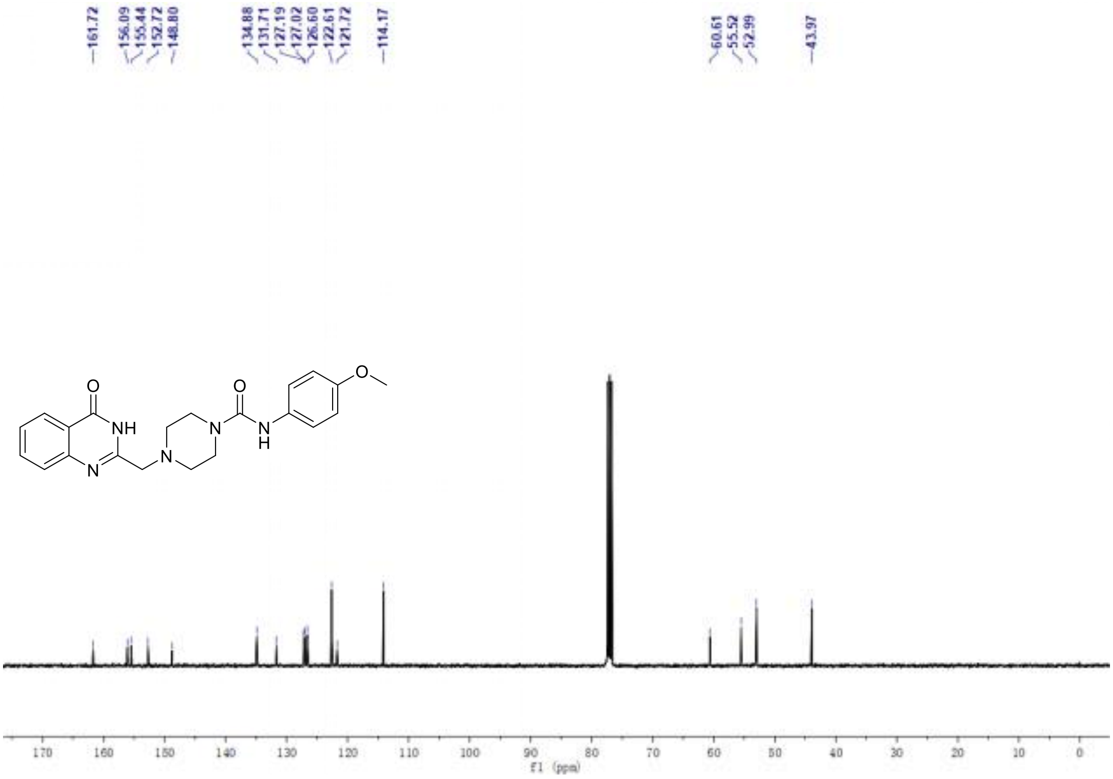

1.19 ^{1}H-NMR and ^{13}C-NMR ($CDCl_3$) spectrum of A19

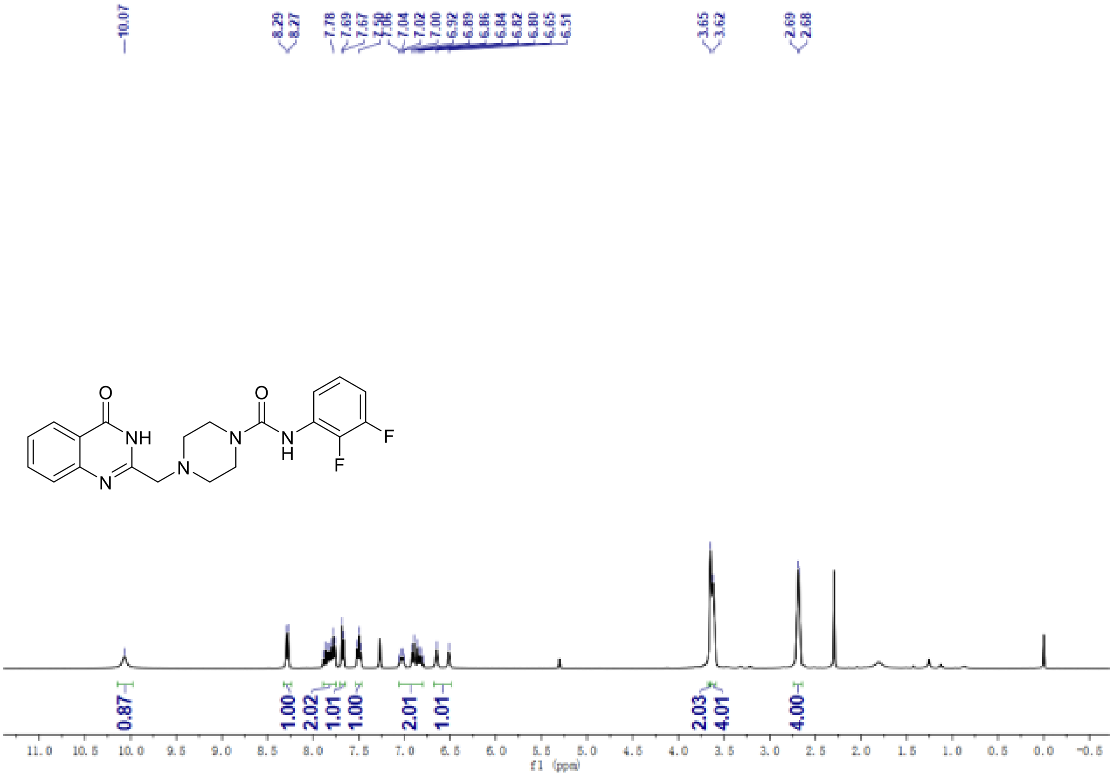

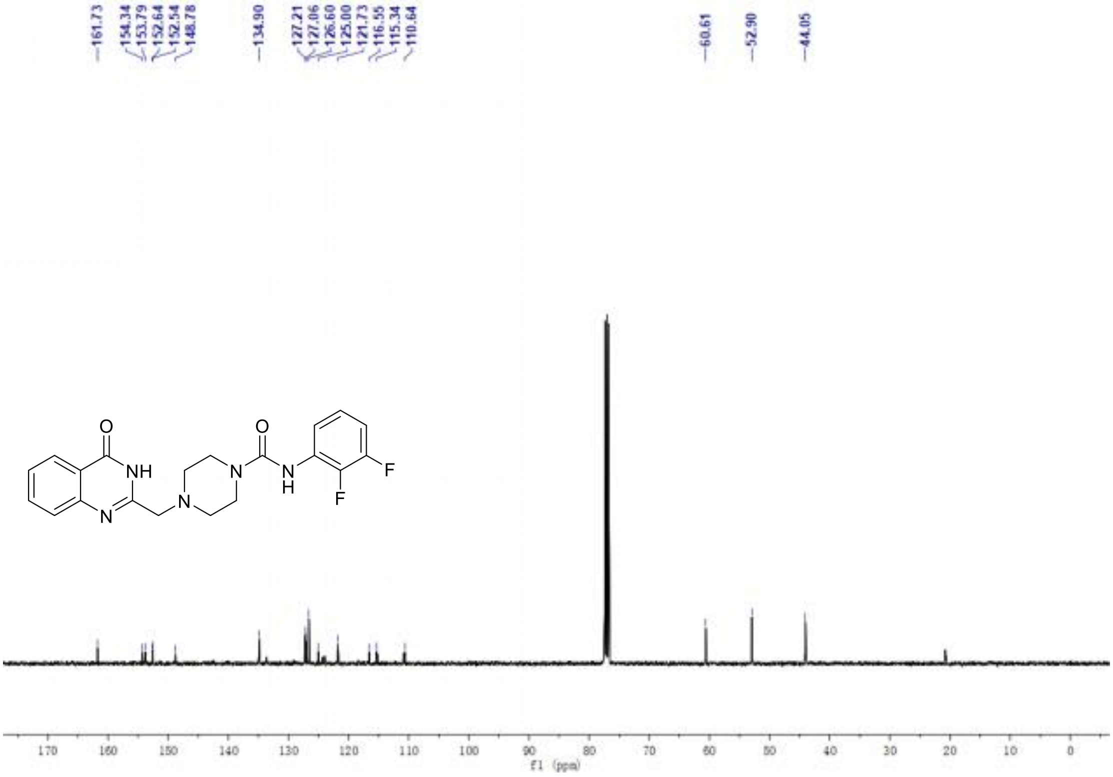

1.20 ^{1}H-NMR and ^{13}C-NMR (DMSO-d$_6$) spectrum of A20

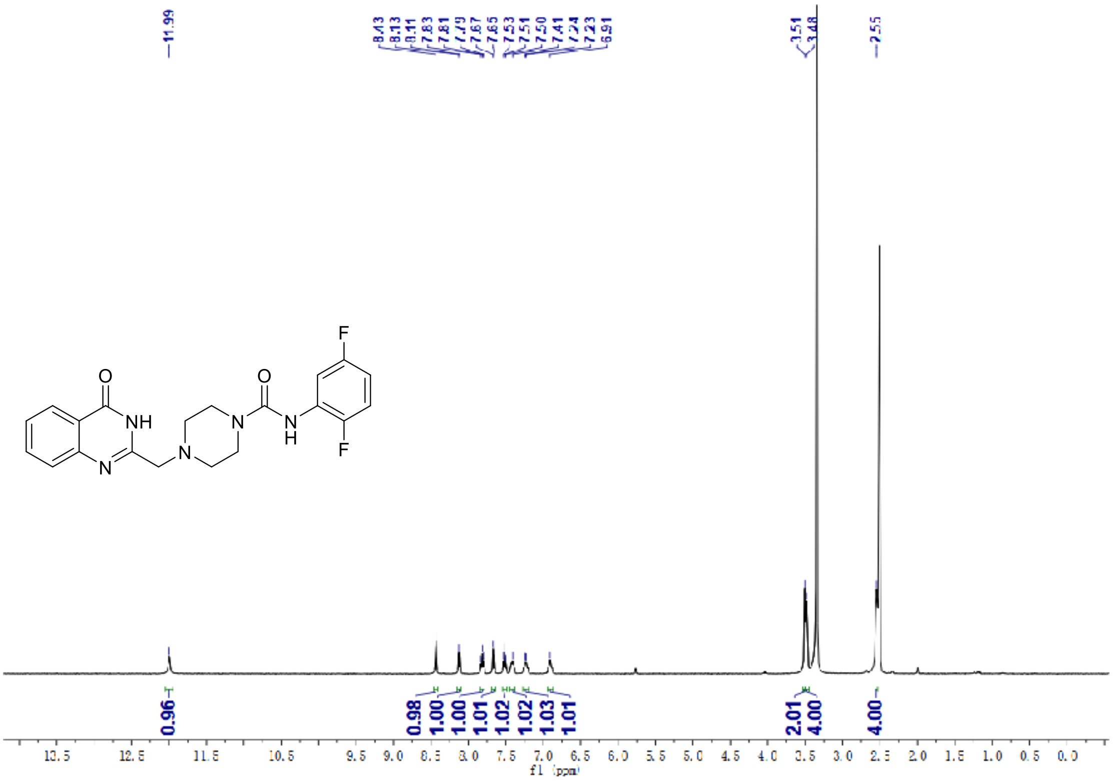

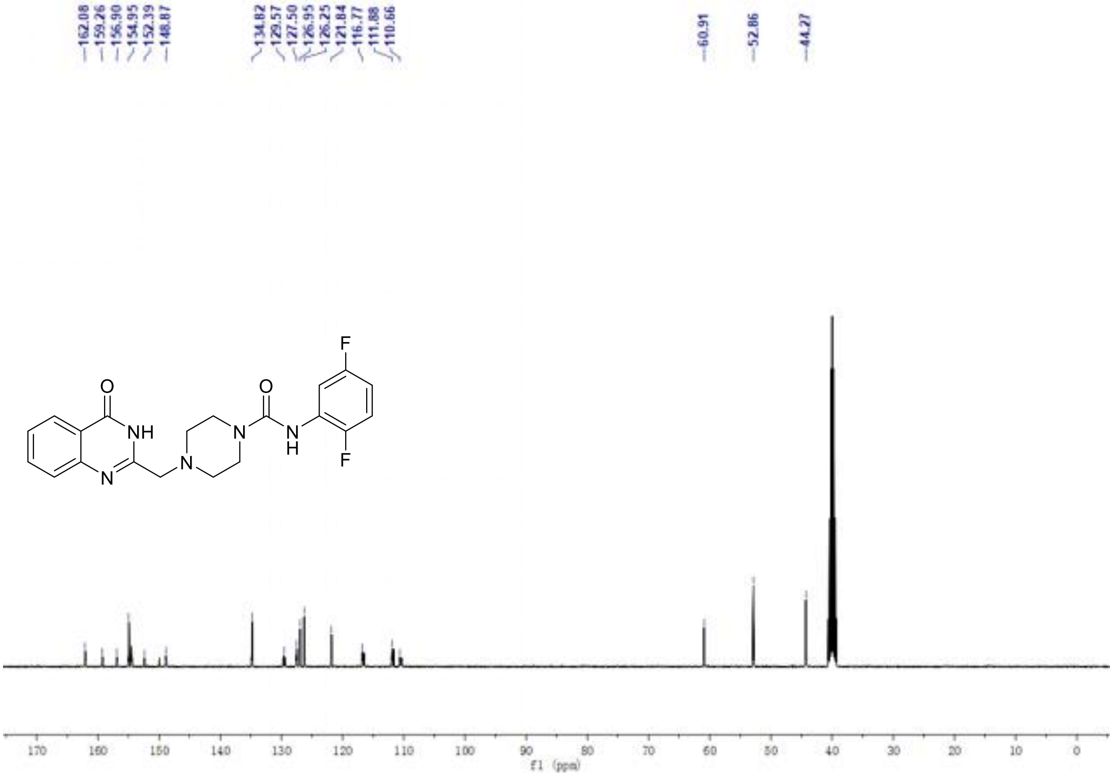

1.21 ^{1}H-NMR and ^{13}C-NMR (DMSO-d$_6$) spectrum of A21

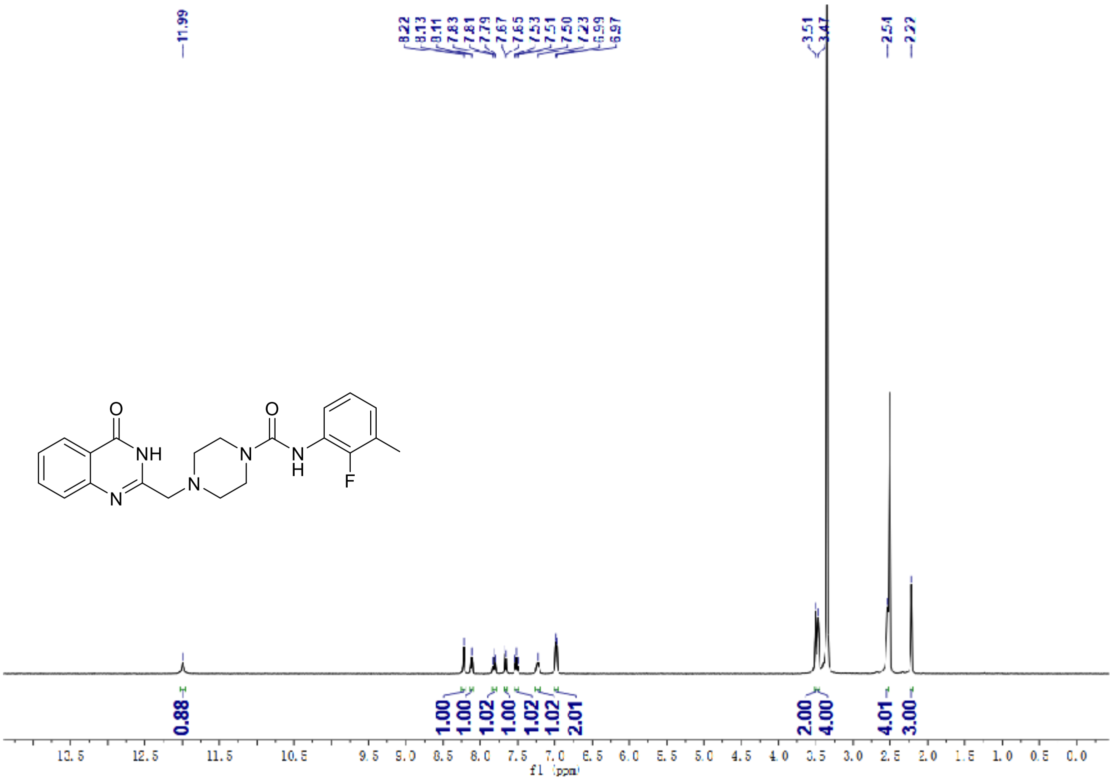

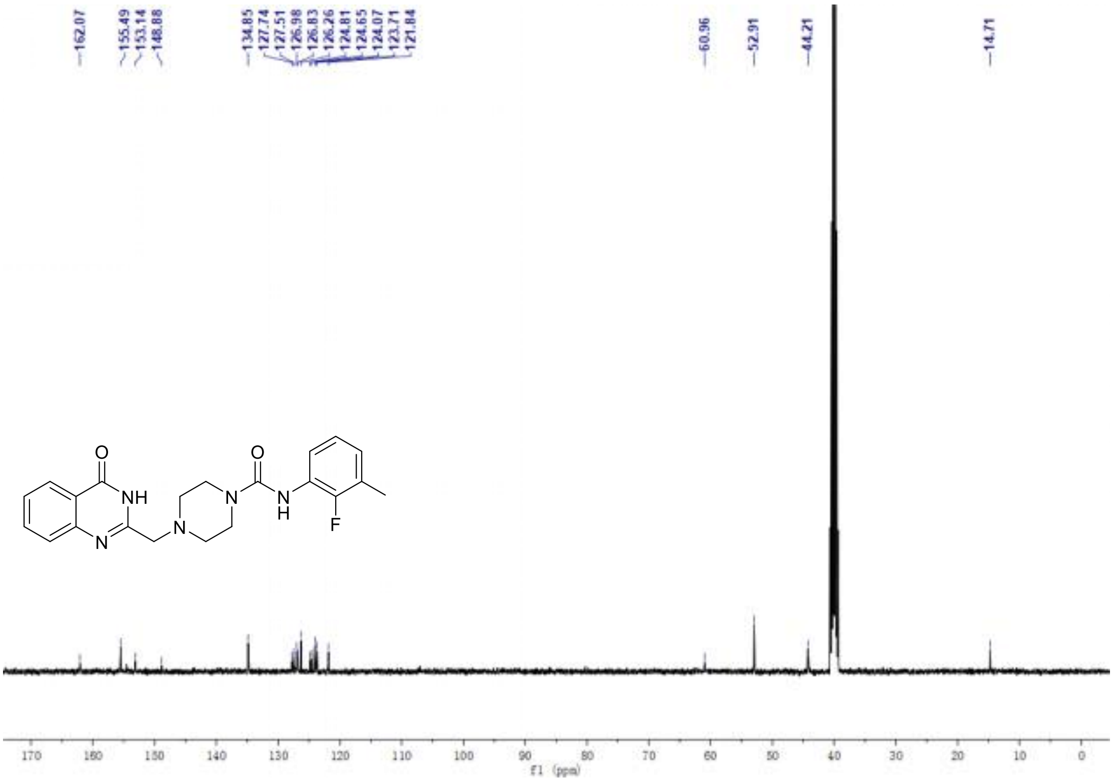

1.22 ^{1}H-NMR and ^{13}C-NMR (DMSO-d$_6$) spectrum of A22

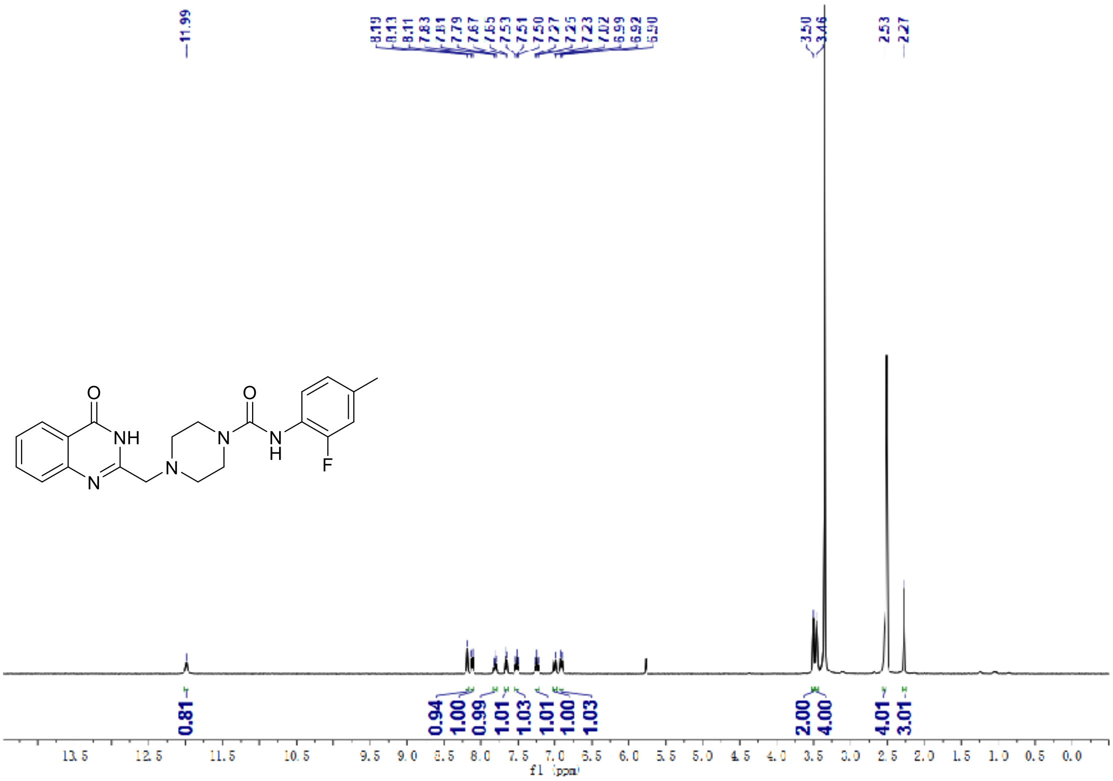

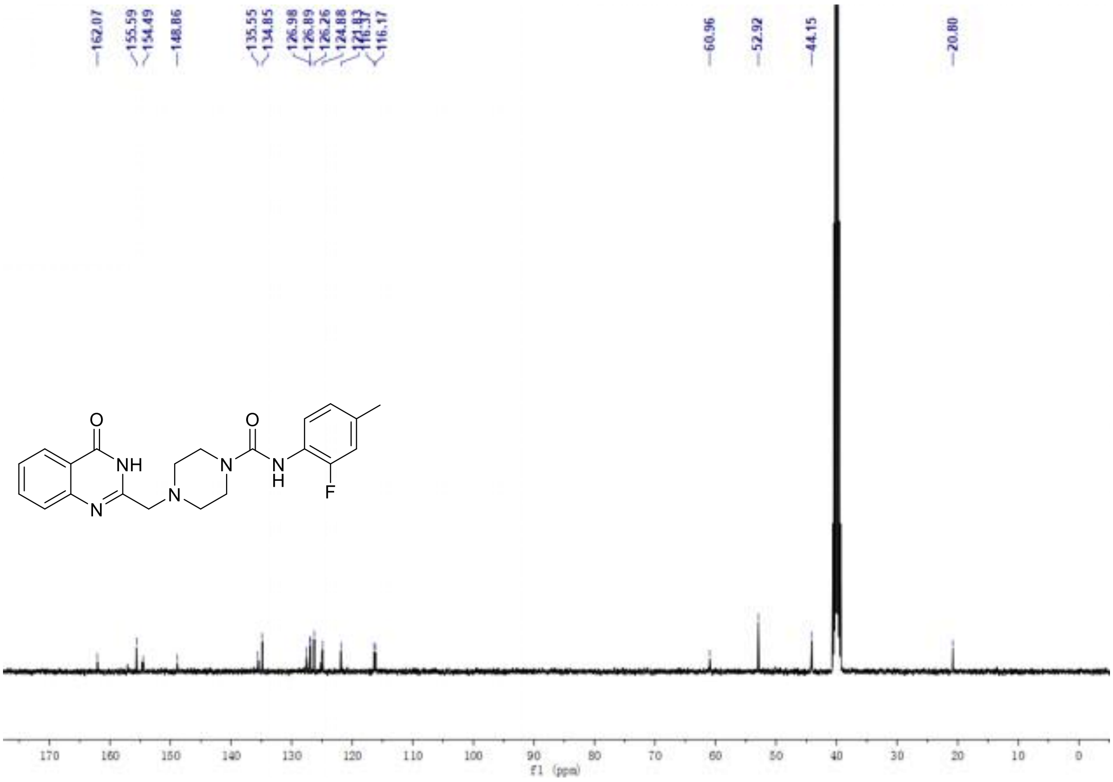

1.23 ^{1}H-NMR and ^{13}C-NMR (CDCl$_3$) spectrum of A23

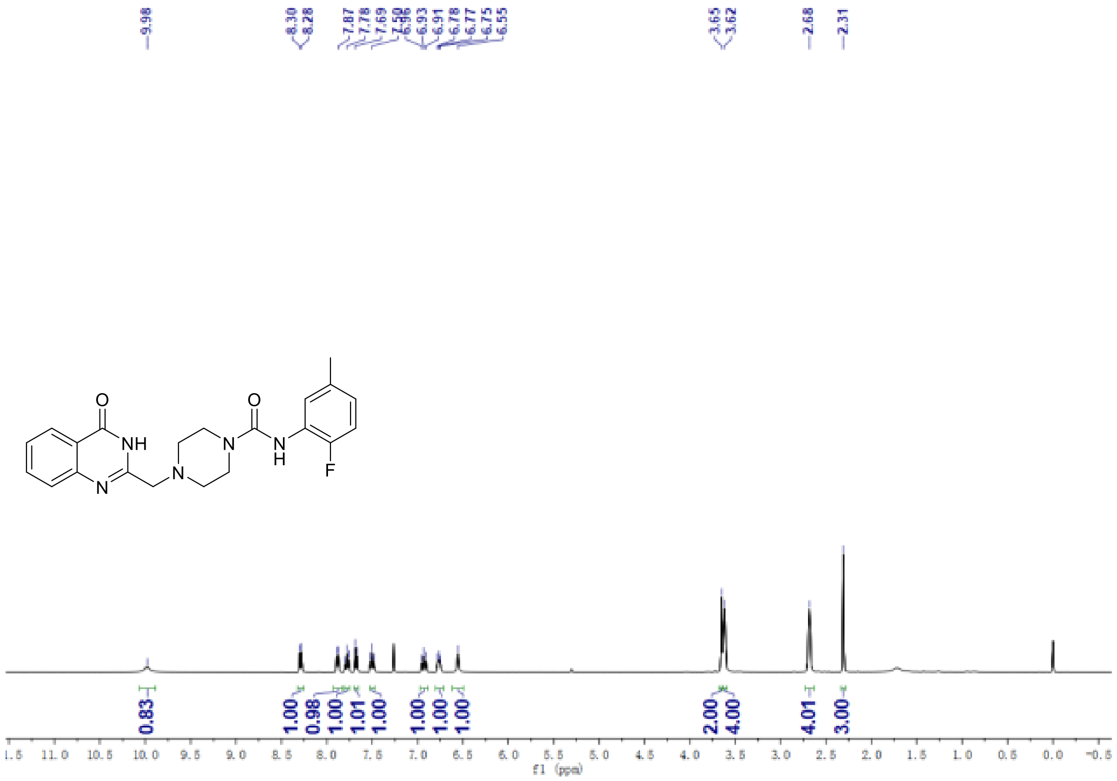

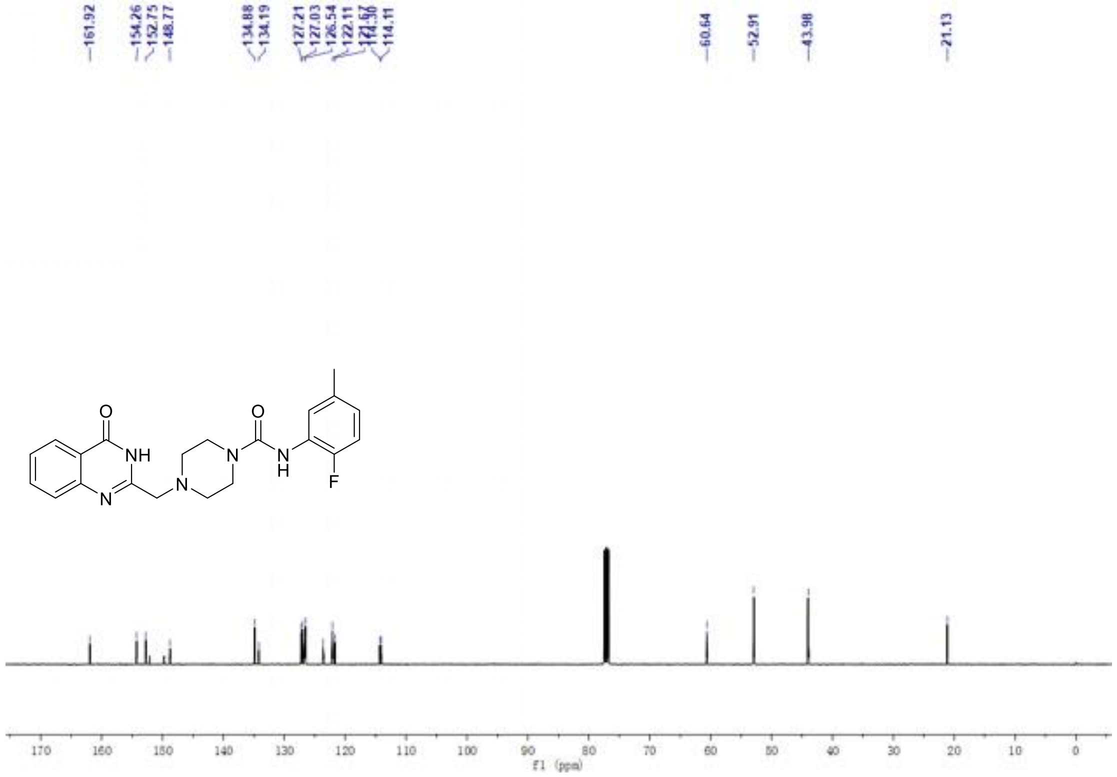

1.24 ^{1}H-NMR and ^{13}C-NMR ($CDCl_3$) spectrum of A24

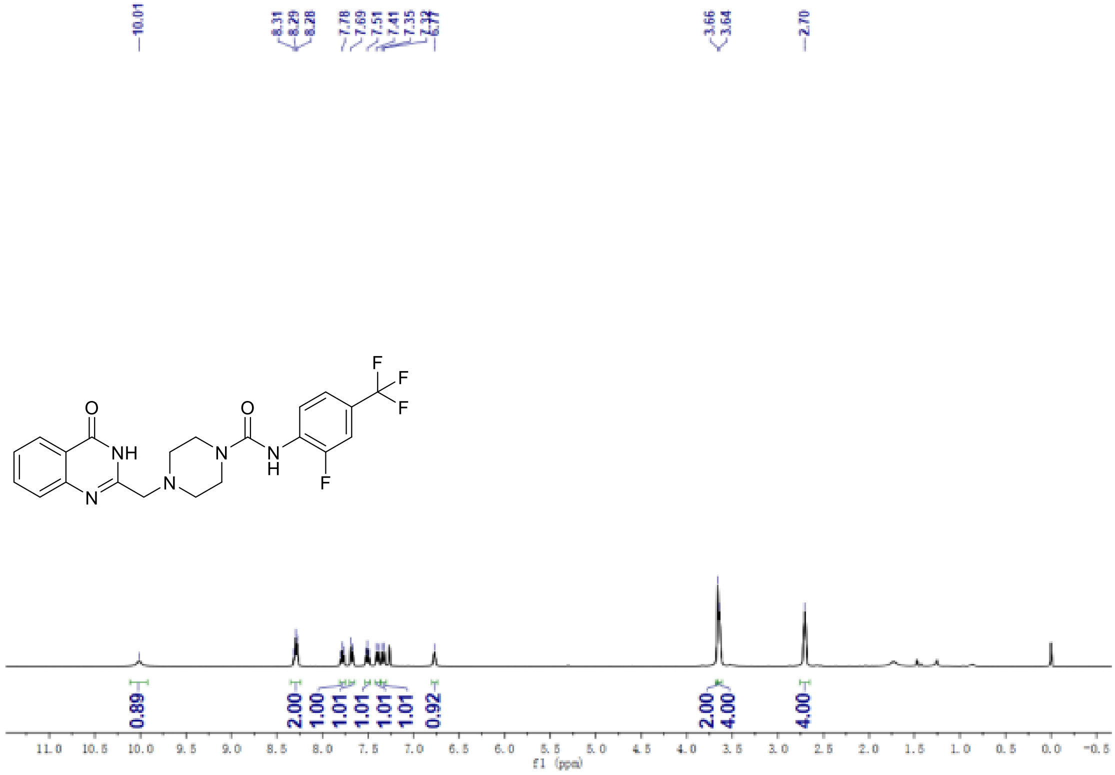

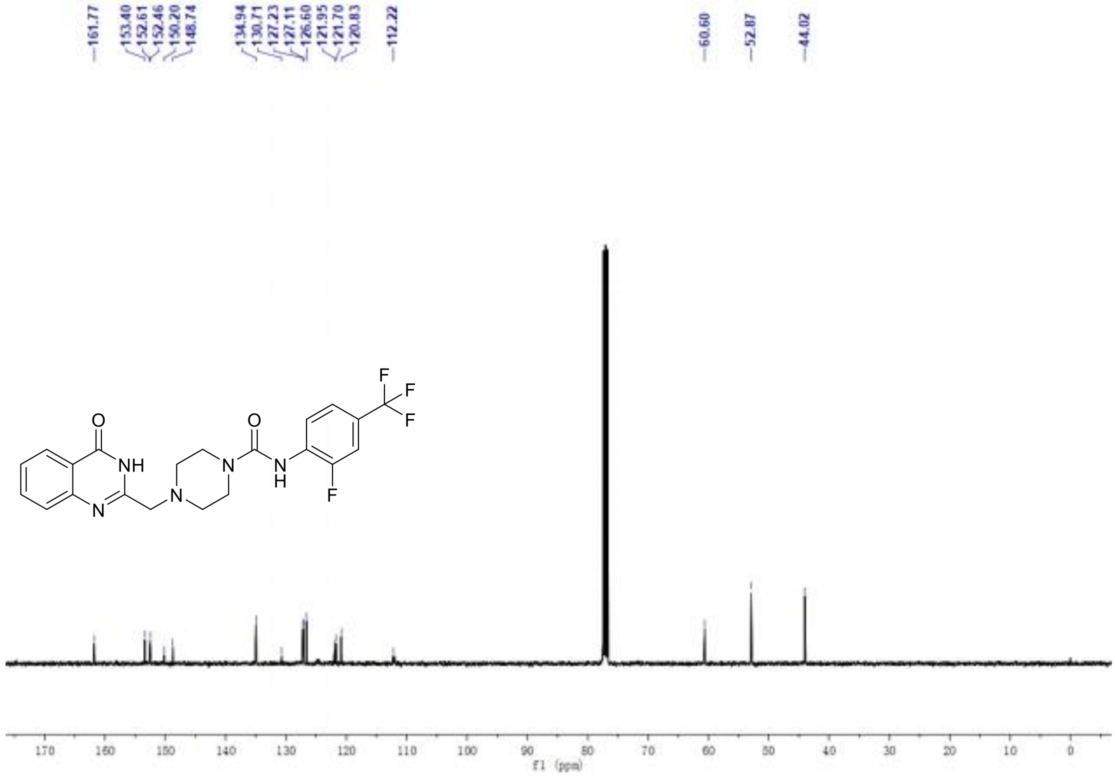

1.25 ^{1}H-NMR and ^{13}C-NMR ($CDCl_3$) spectrum of A25

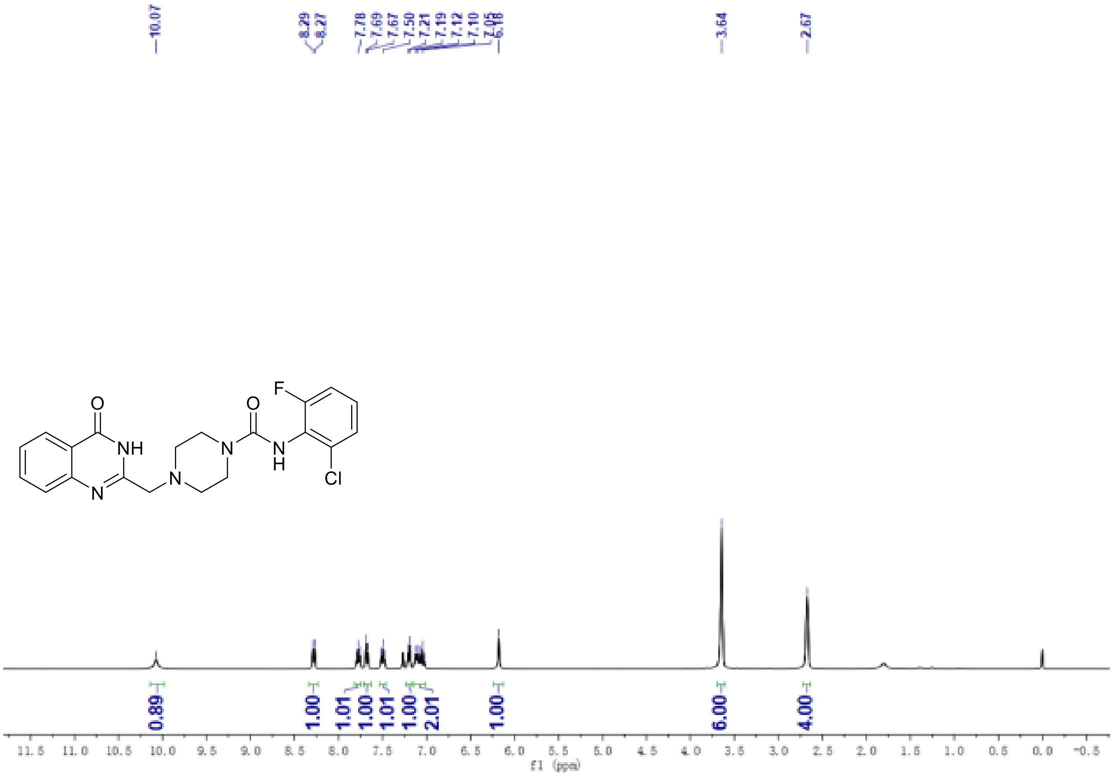

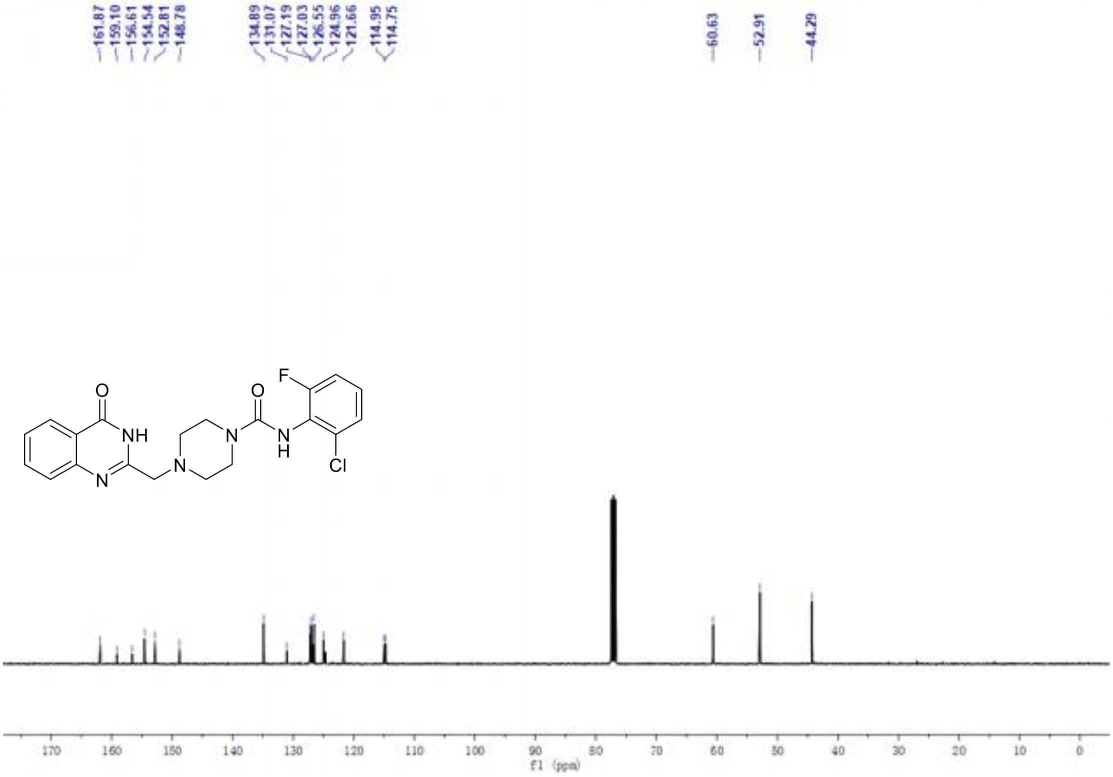

1.26 ^{1}H-NMR and ^{13}C-NMR ($CDCl_3$) spectrum of A26

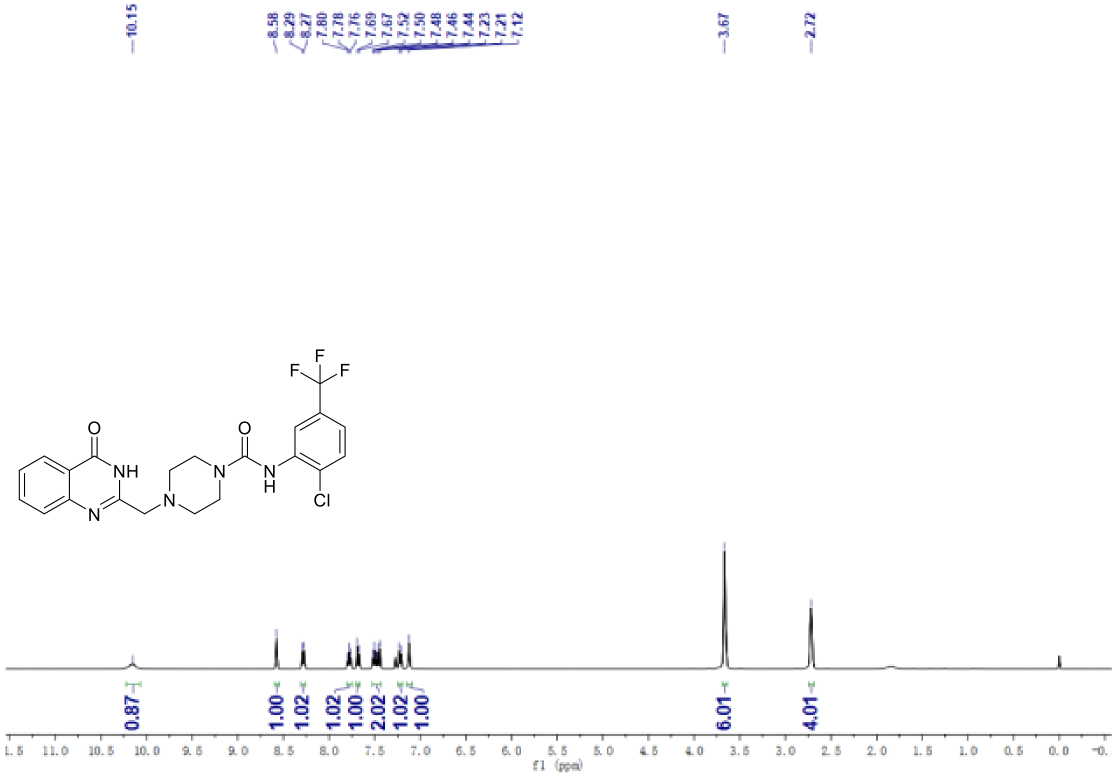

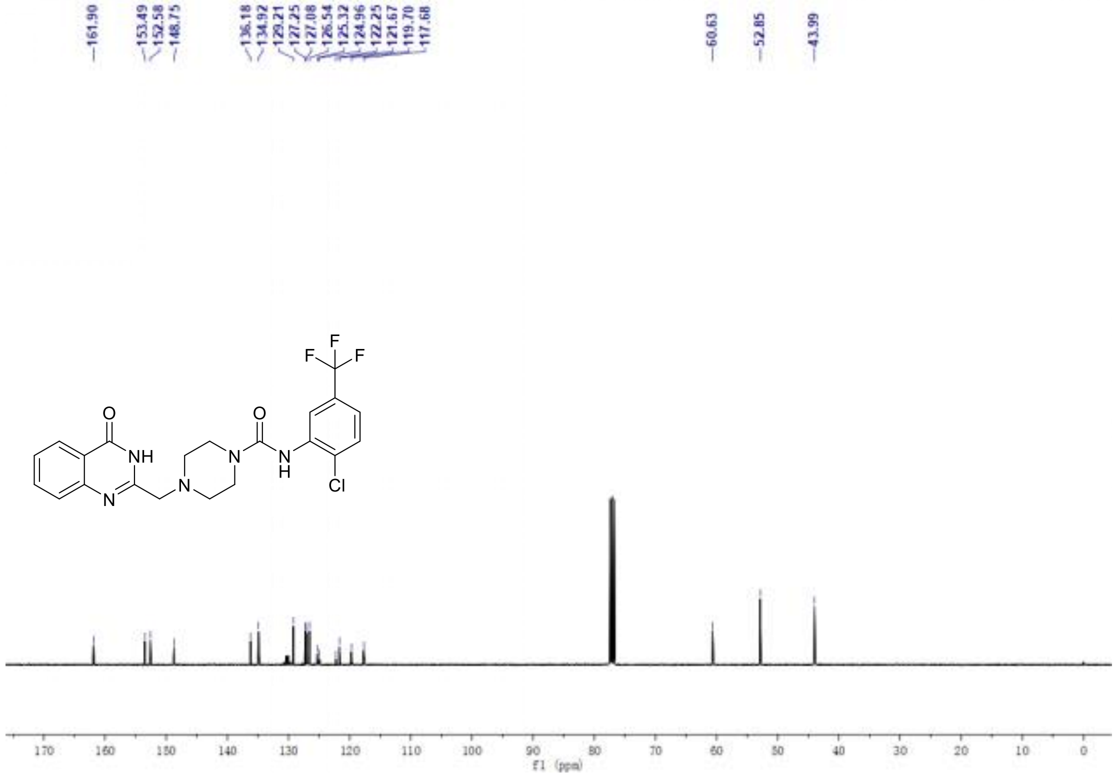

1.27 ^{1}H-NMR and ^{13}C-NMR (CDCl$_3$) spectrum of A27

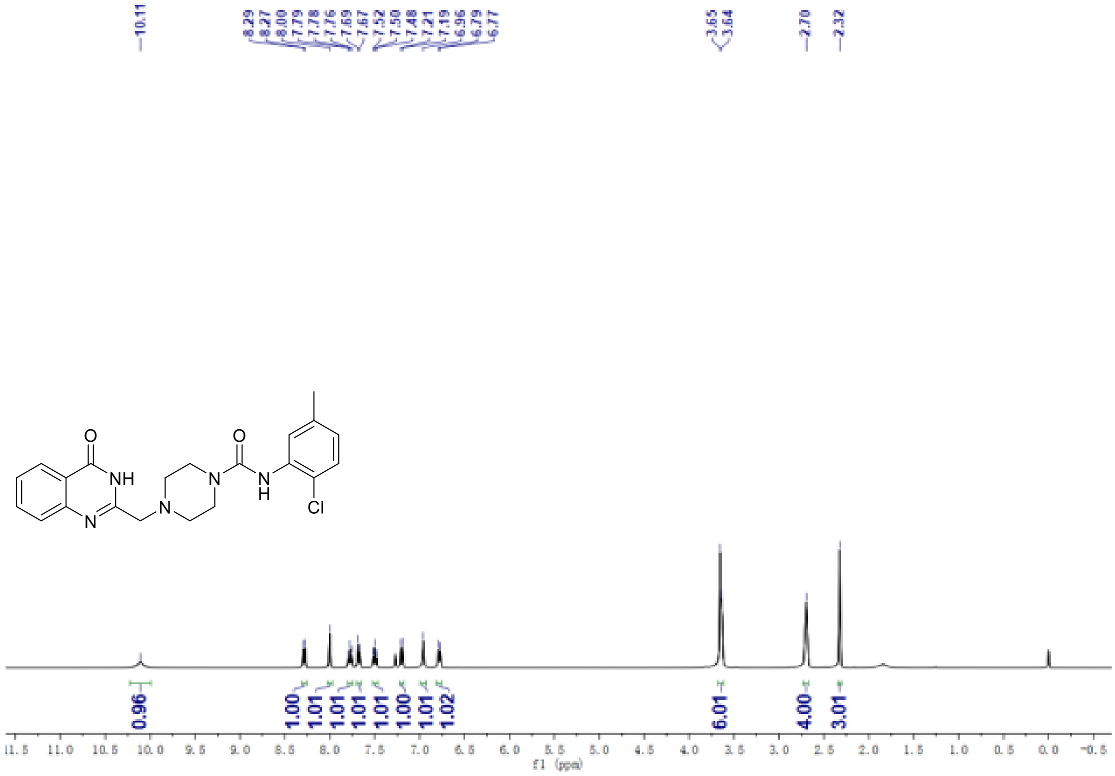

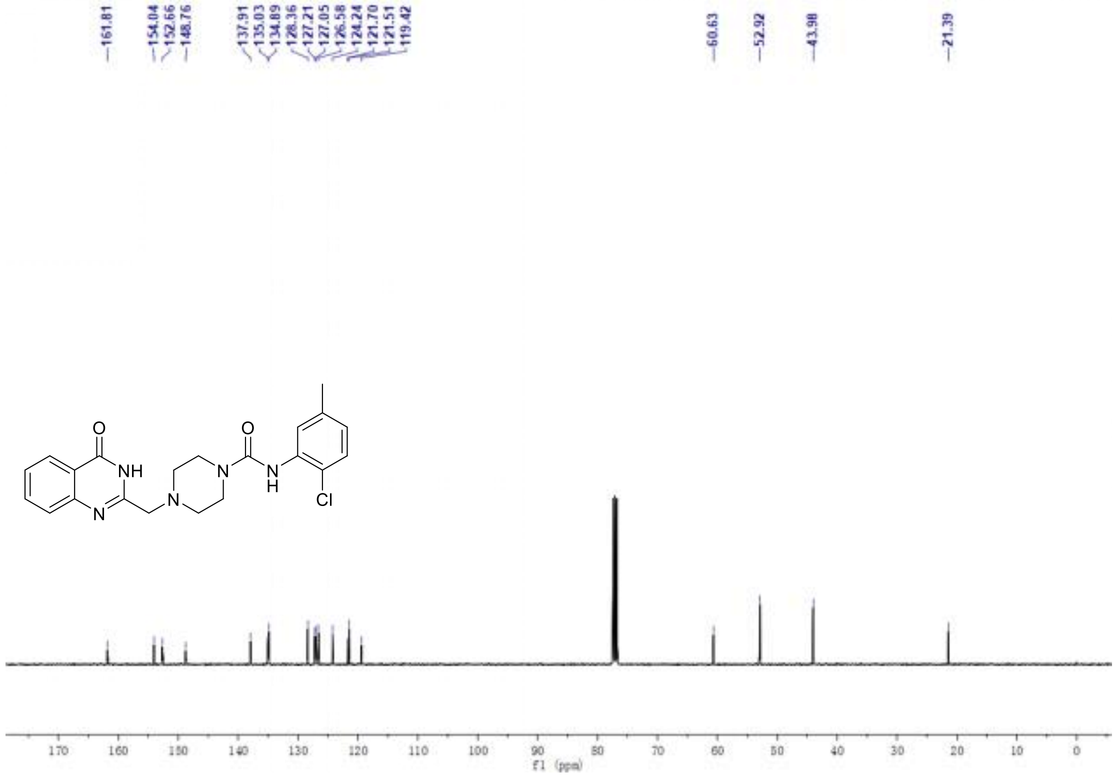

1.28 ^{1}H-NMR and ^{13}C-NMR (DMSO-d$_6$) spectrum of A28

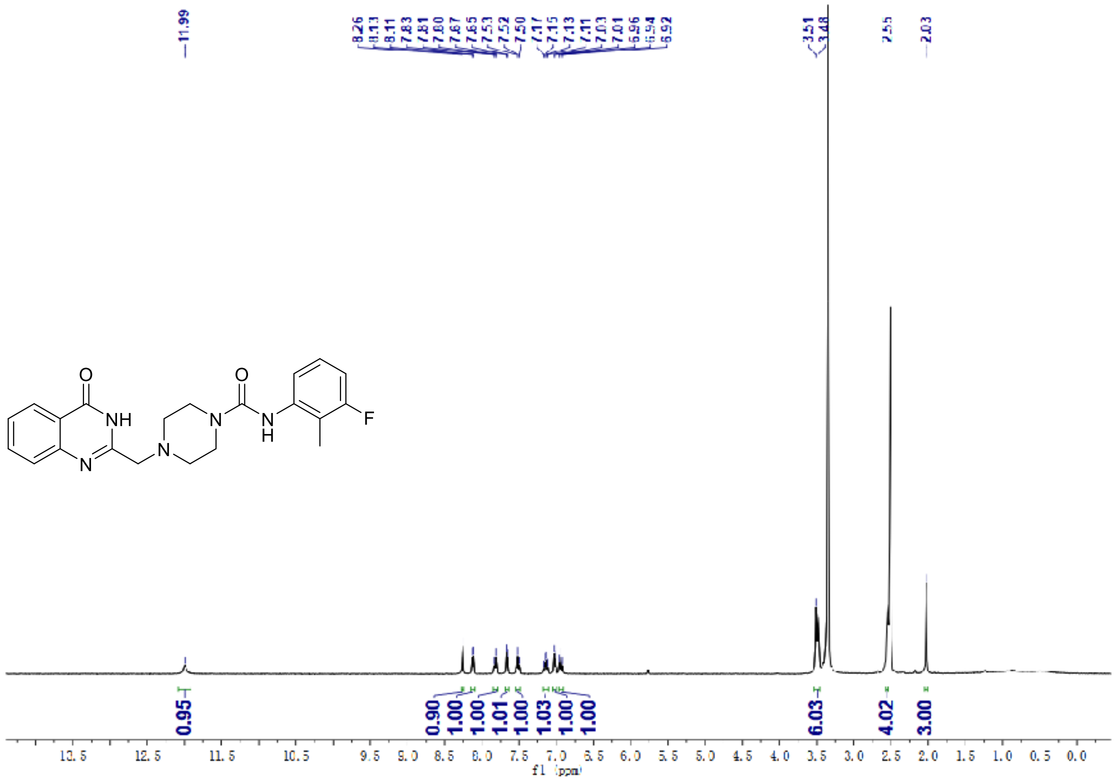

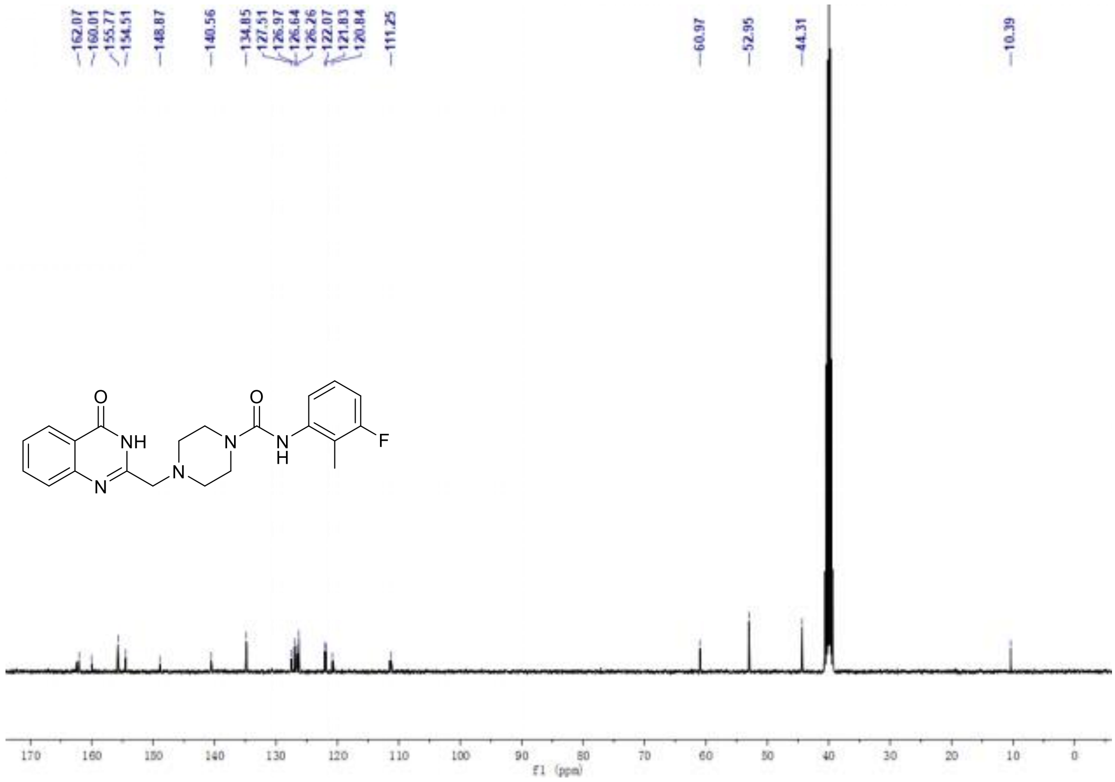

1.29 ^{1}H-NMR and ^{13}C-NMR (DMSO-d$_6$) spectrum of A29

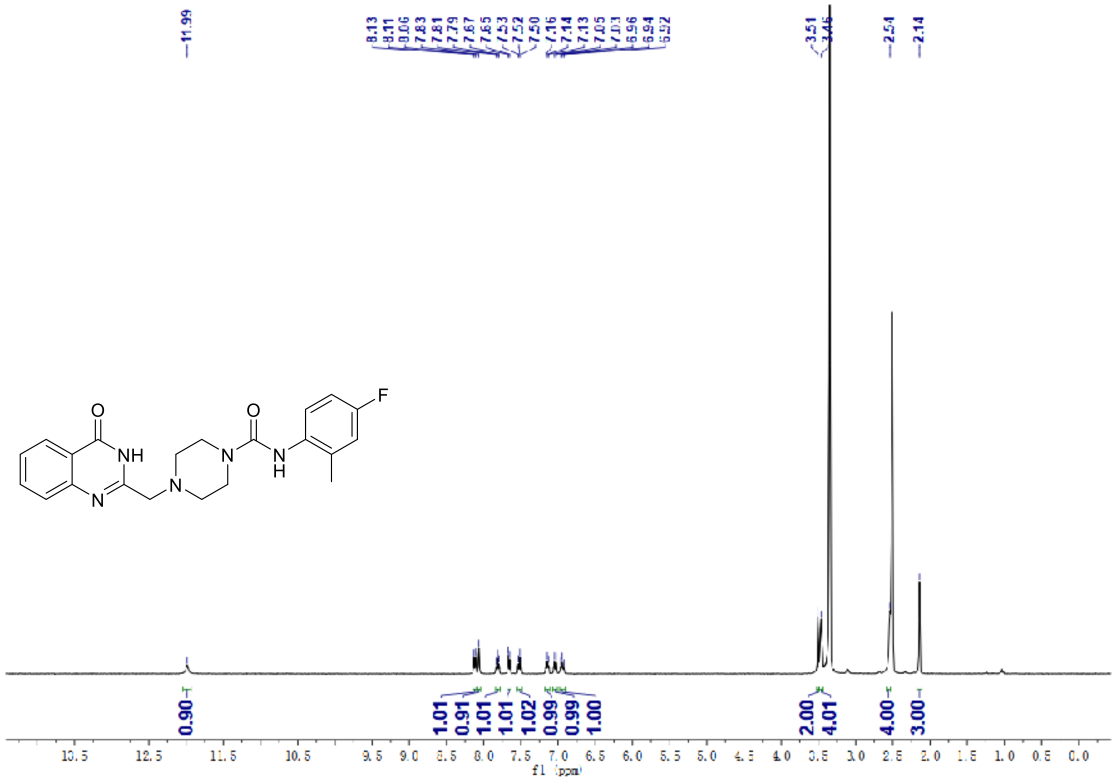

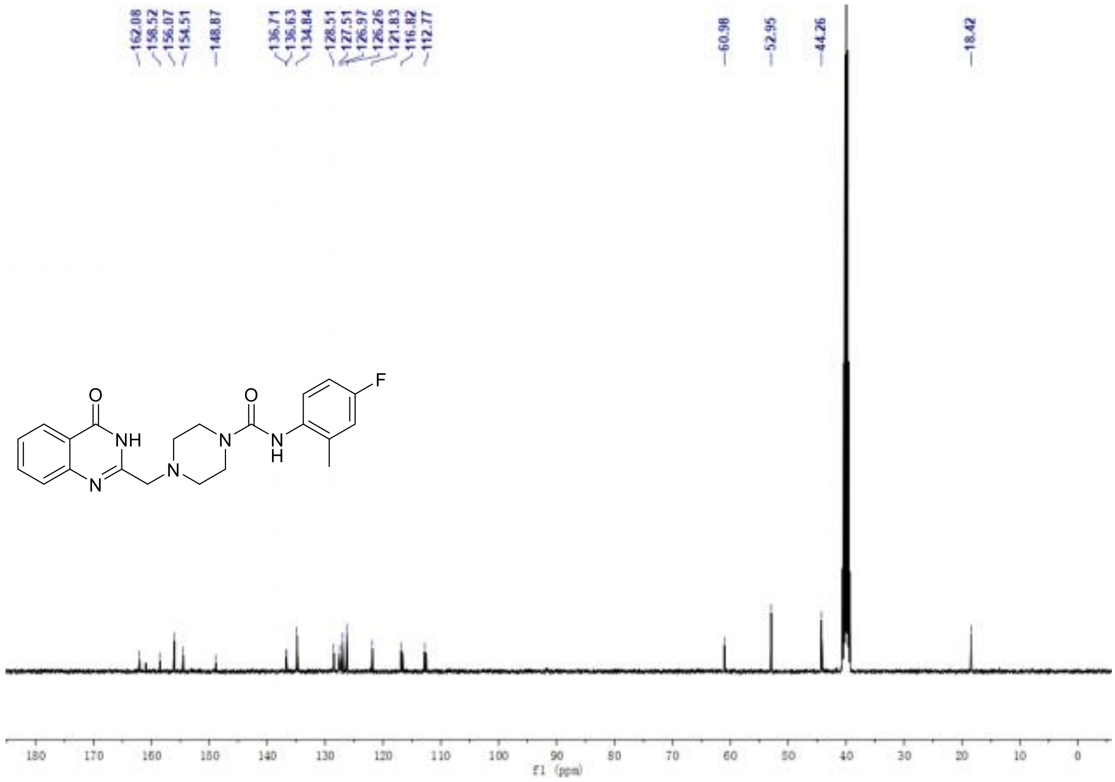

1.30 ^{1}H-NMR and ^{13}C-NMR (CDCl$_3$) spectrum of A30

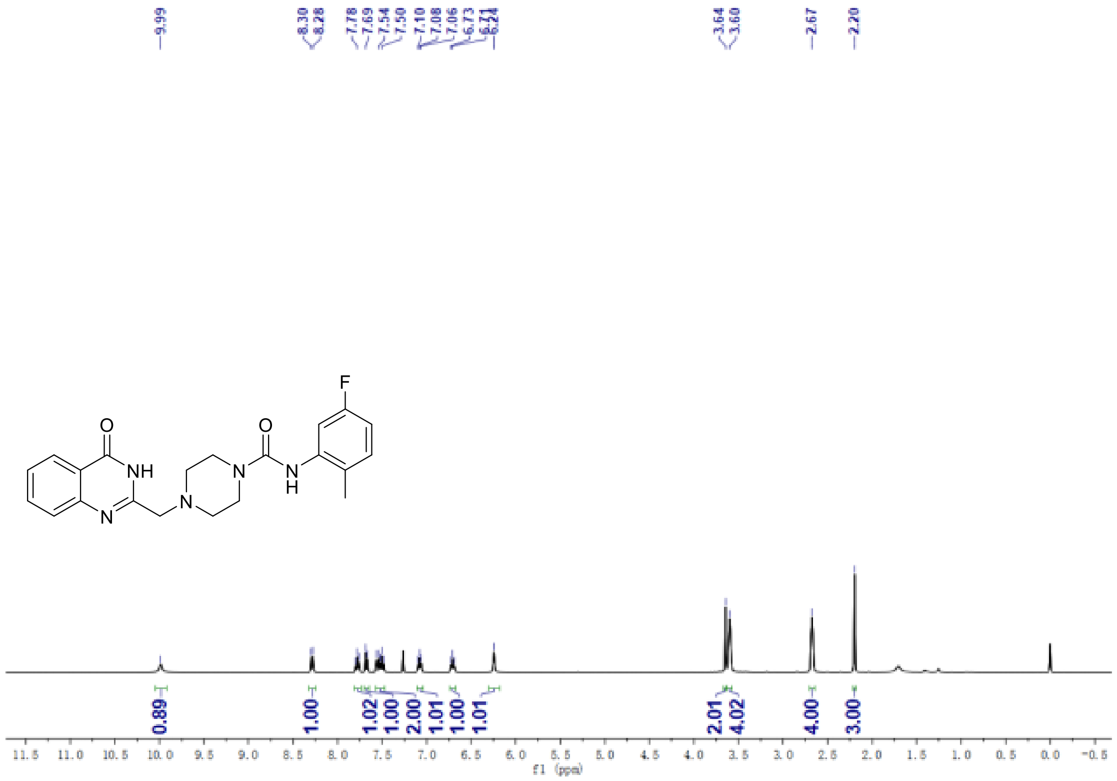

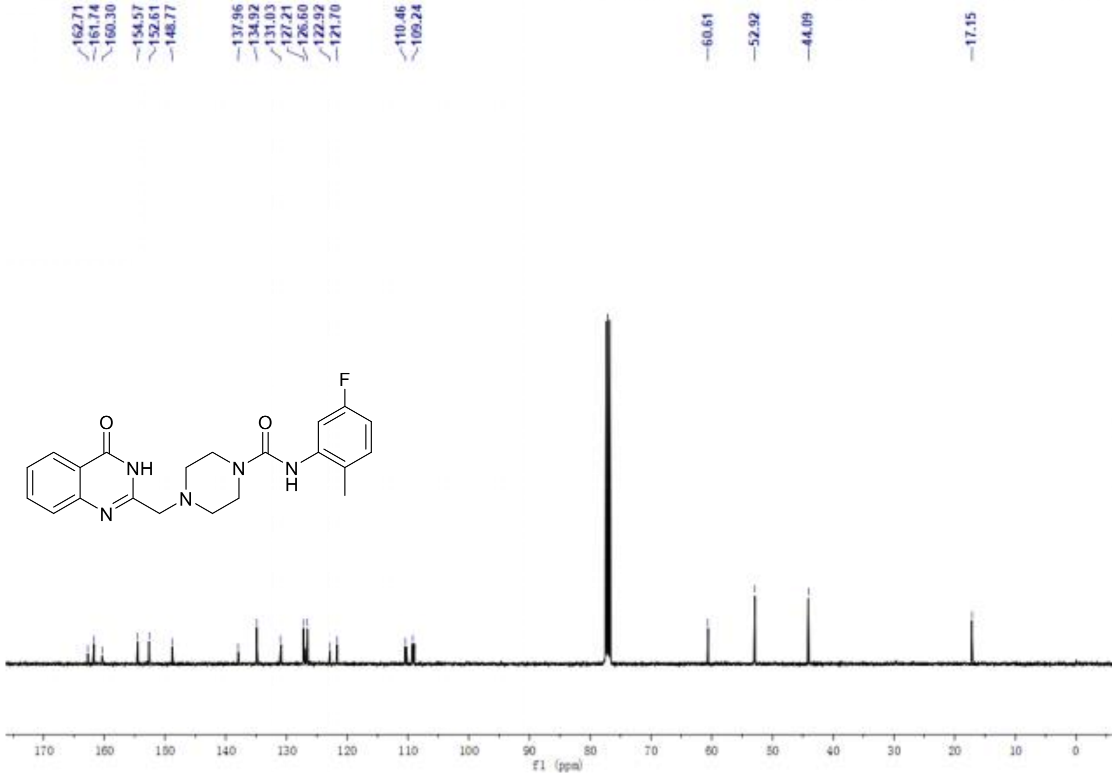

1.31 ^{1}H-NMR and ^{13}C-NMR (DMSO-d_6) spectrum of A31

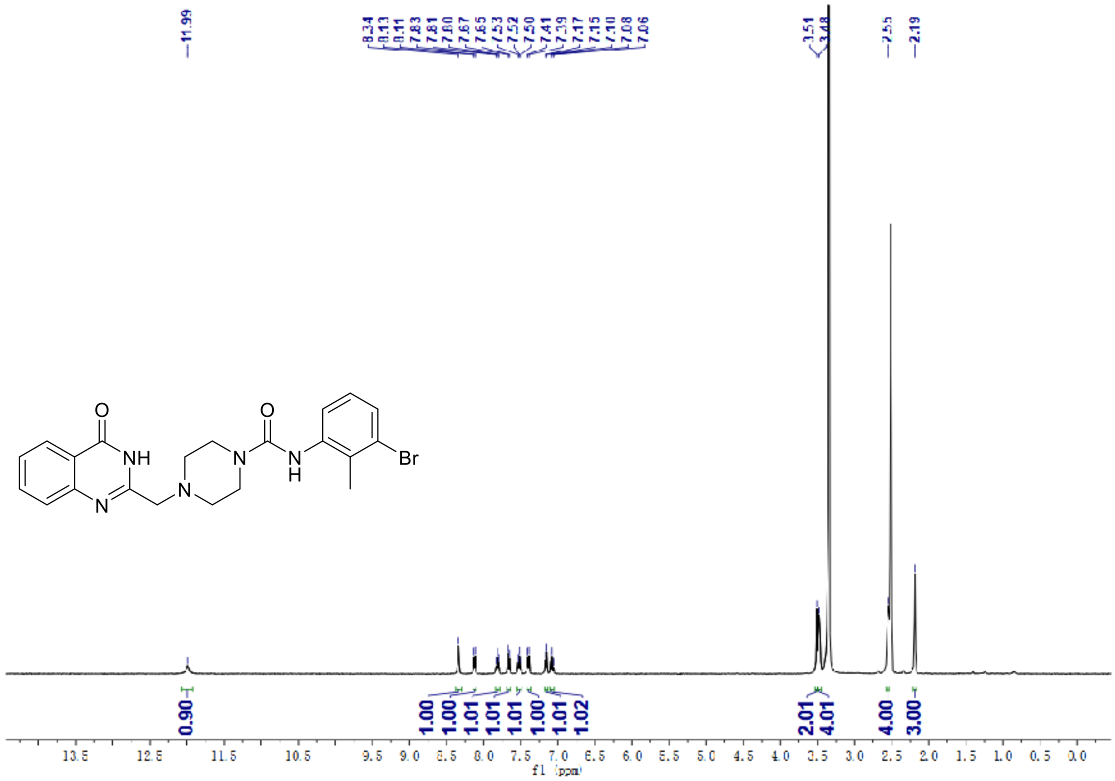

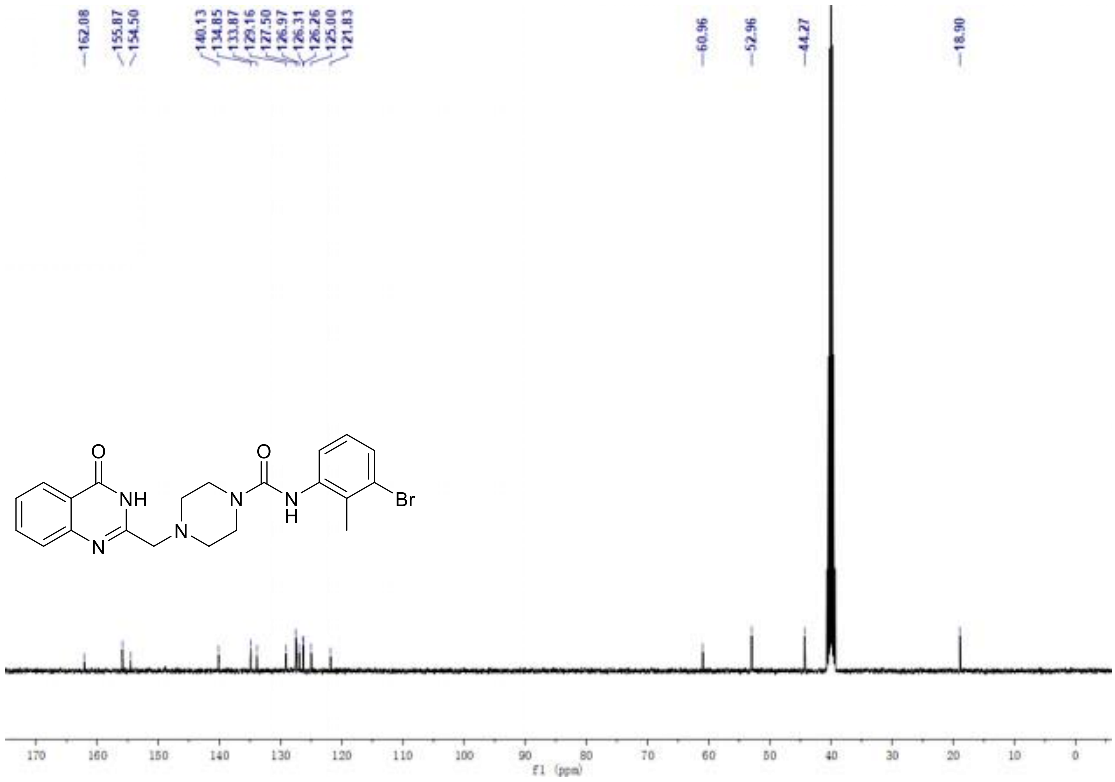

1.32 ^{1}H-NMR and ^{13}C-NMR (DMSO-d_6) spectrum of A32

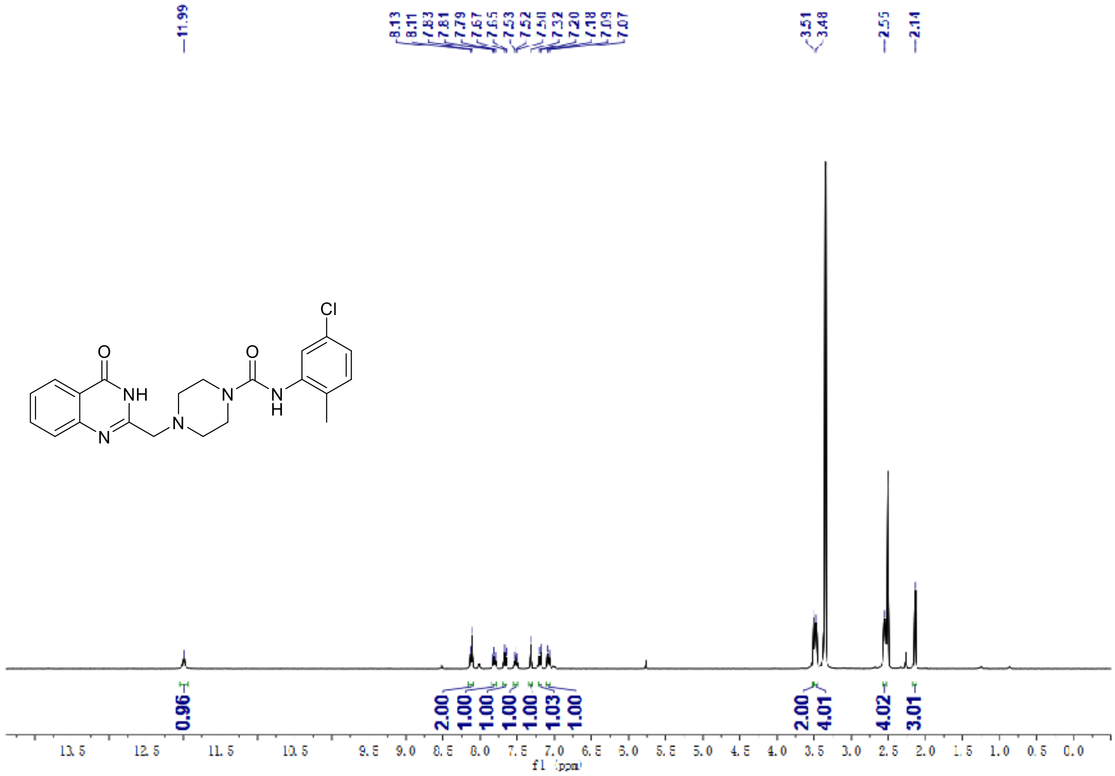

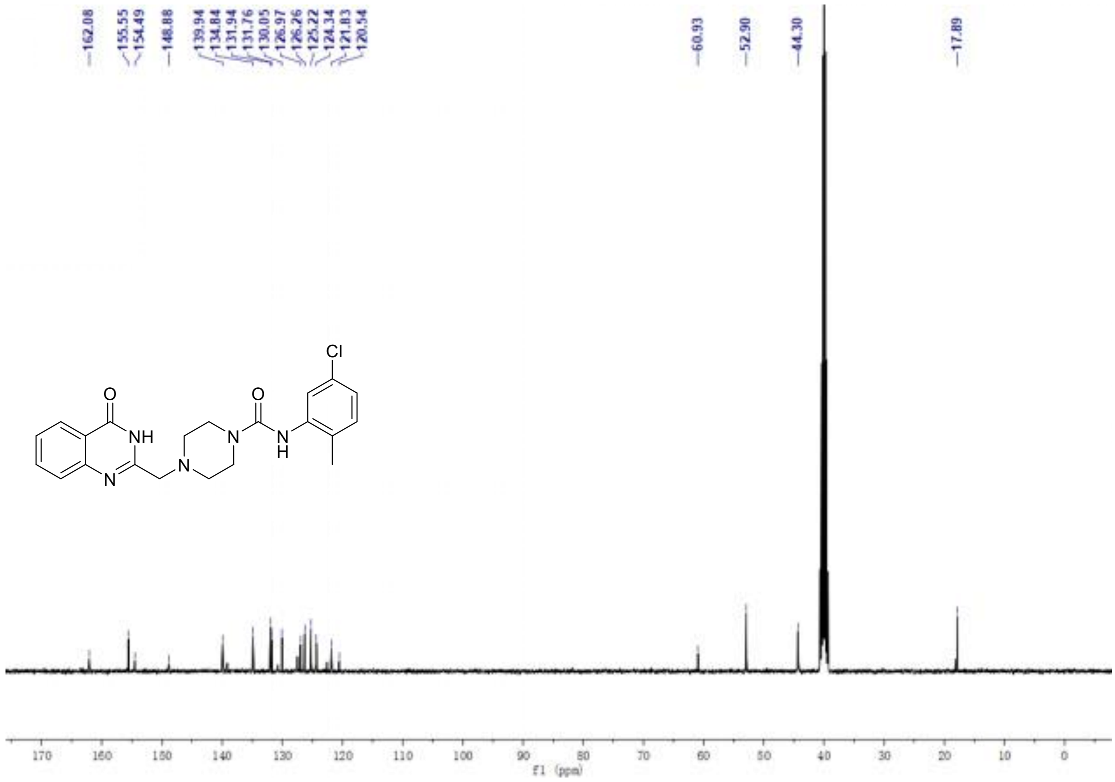

1.33 ^{1}H-NMR and ^{13}C-NMR (DMSO-d_6) spectrum of A33

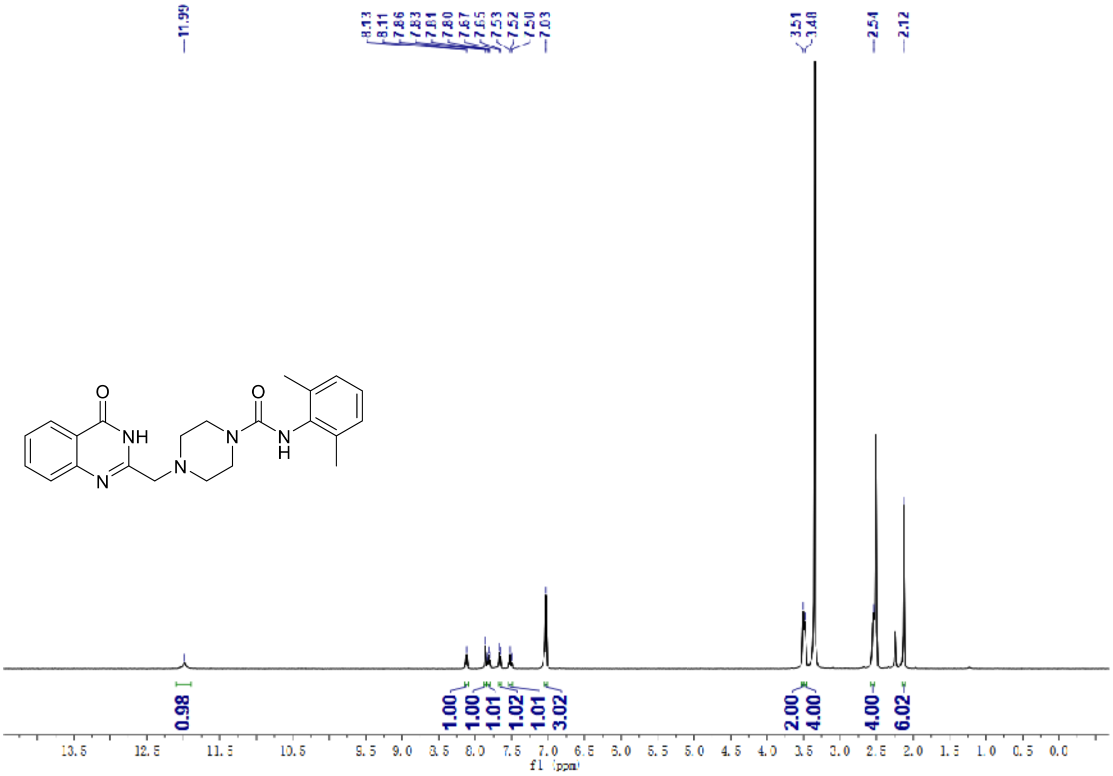

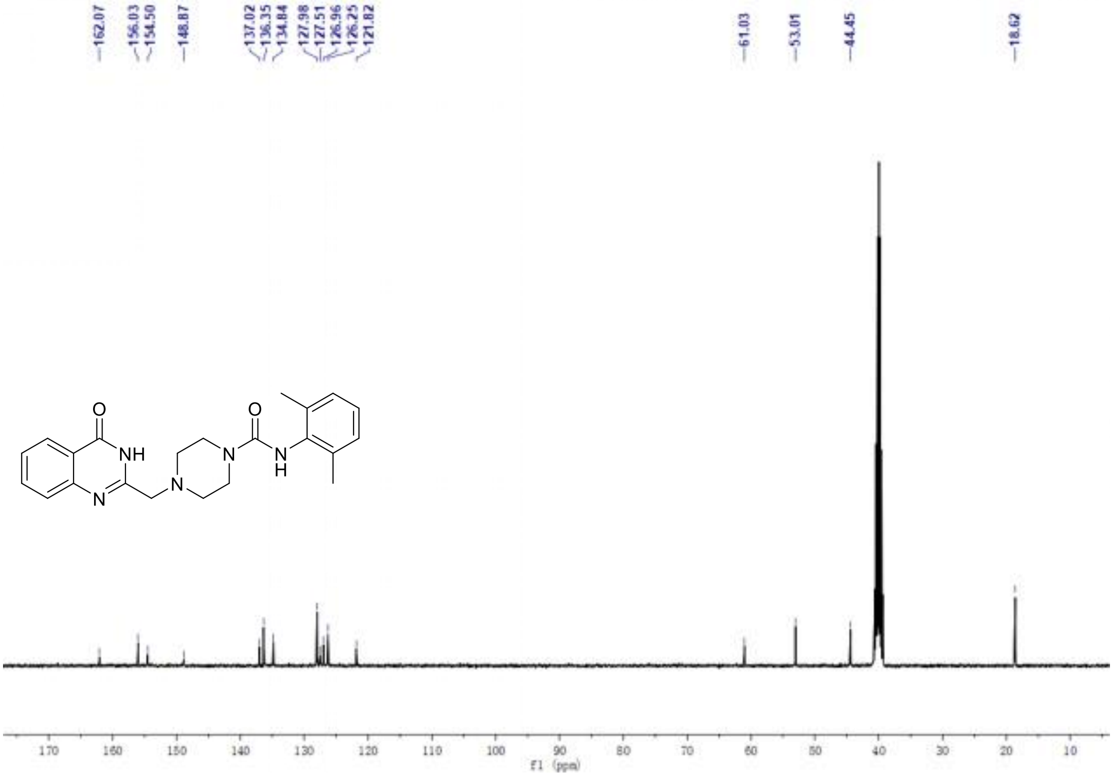

1.34 ^{1}H-NMR and ^{13}C-NMR (DMSO-d$_6$) spectrum of A34

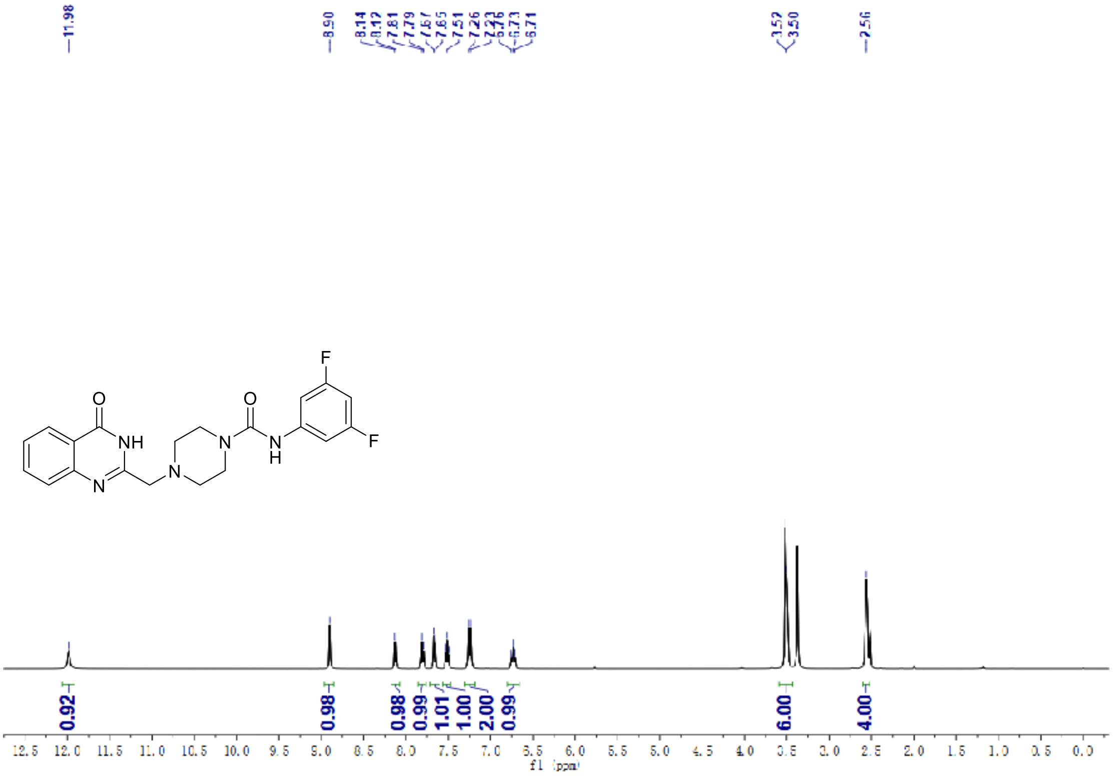

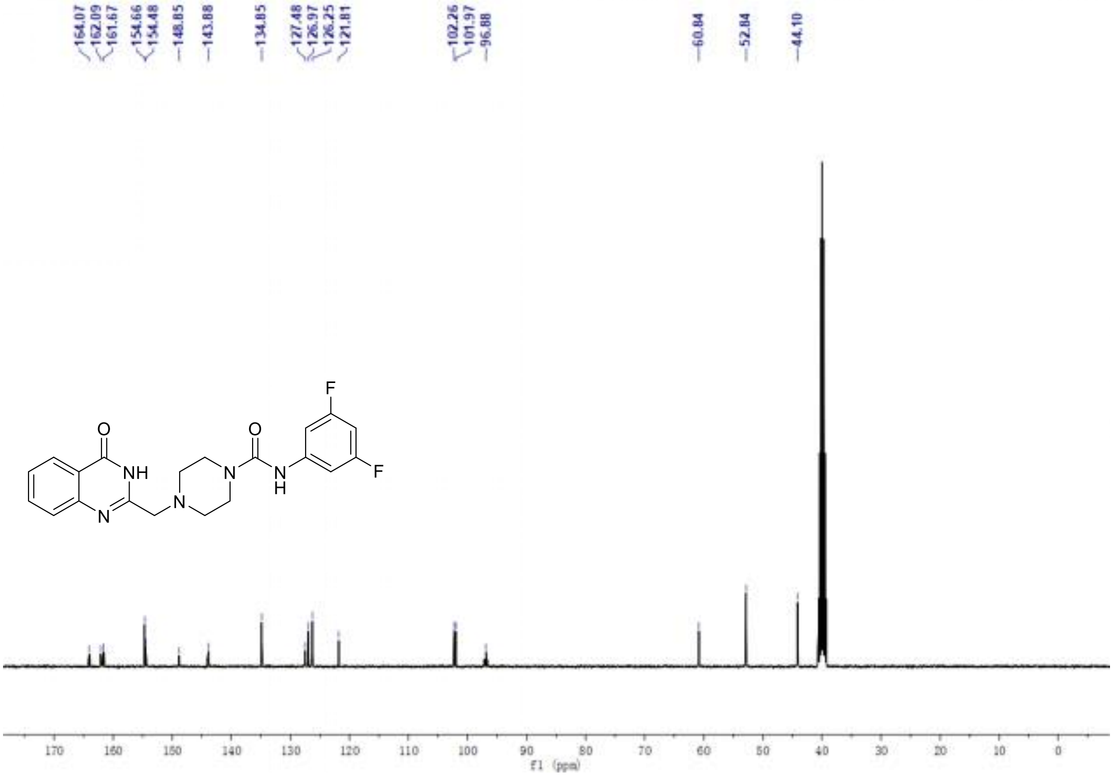

1.35 ^{1}H-NMR and ^{13}C-NMR (DMSO-d$_6$) spectrum of A35

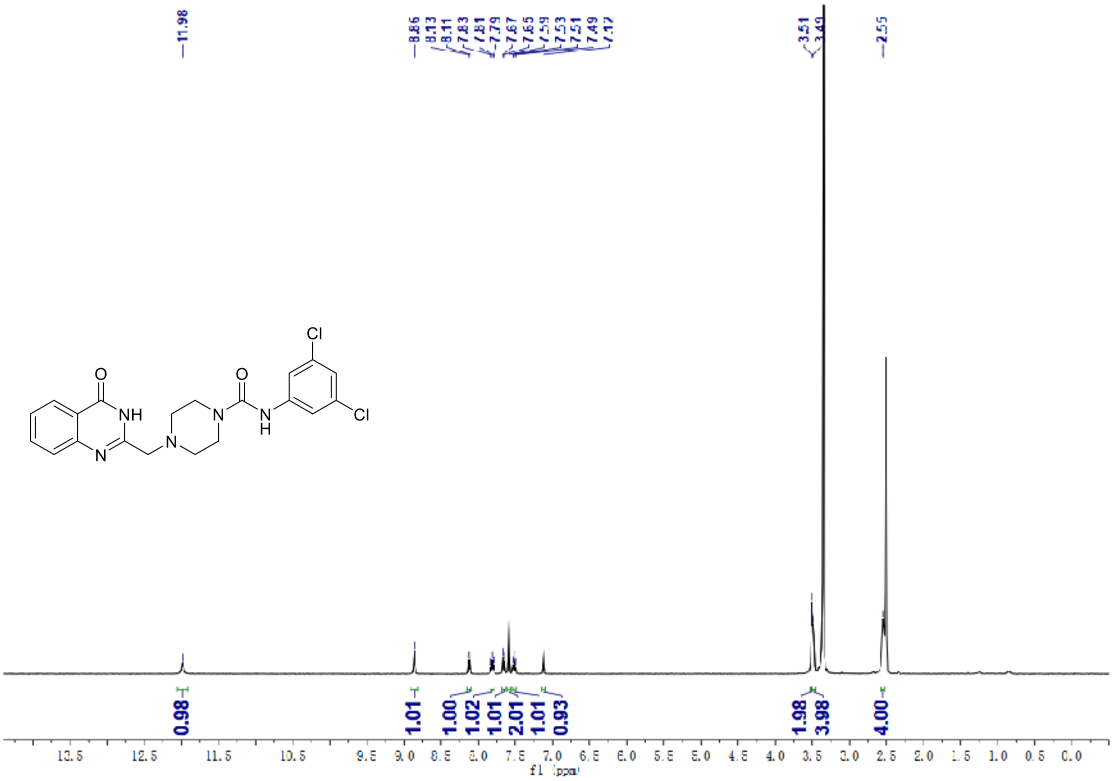

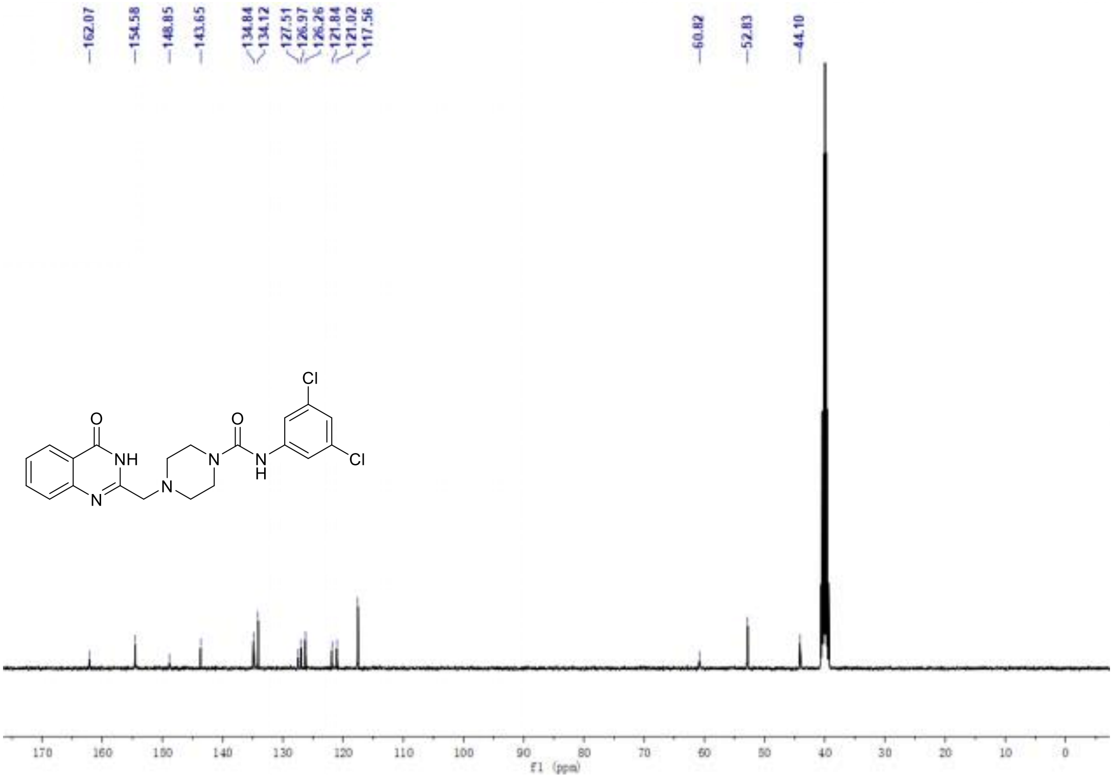

1.36 ^{1}H-NMR and ^{13}C-NMR (DMSO-d$_6$) spectrum of A36

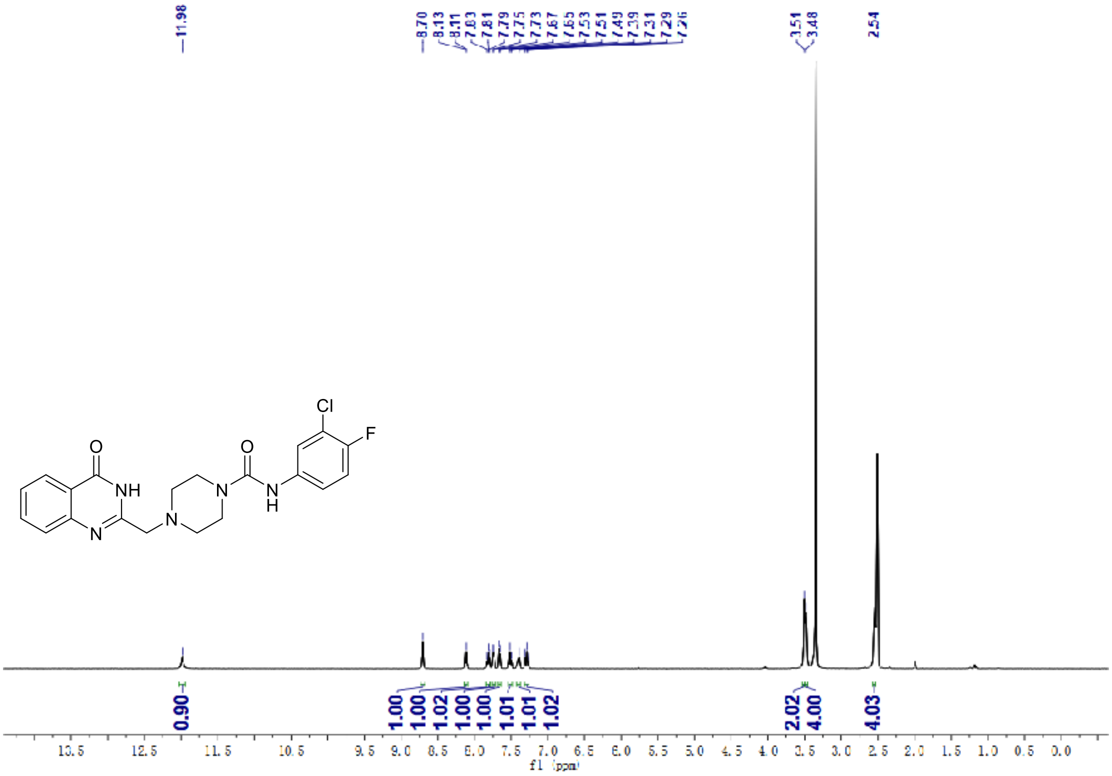

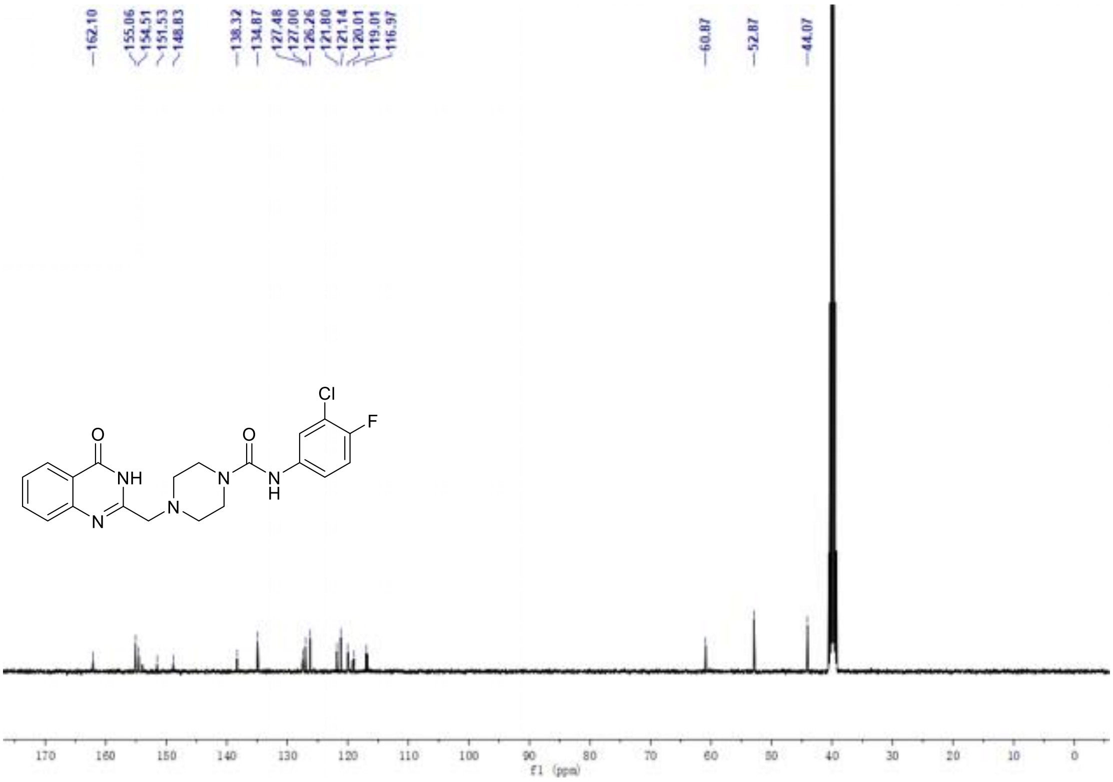

1.37 ^{1}H-NMR and ^{13}C-NMR (DMSO-d$_6$) spectrum of A37

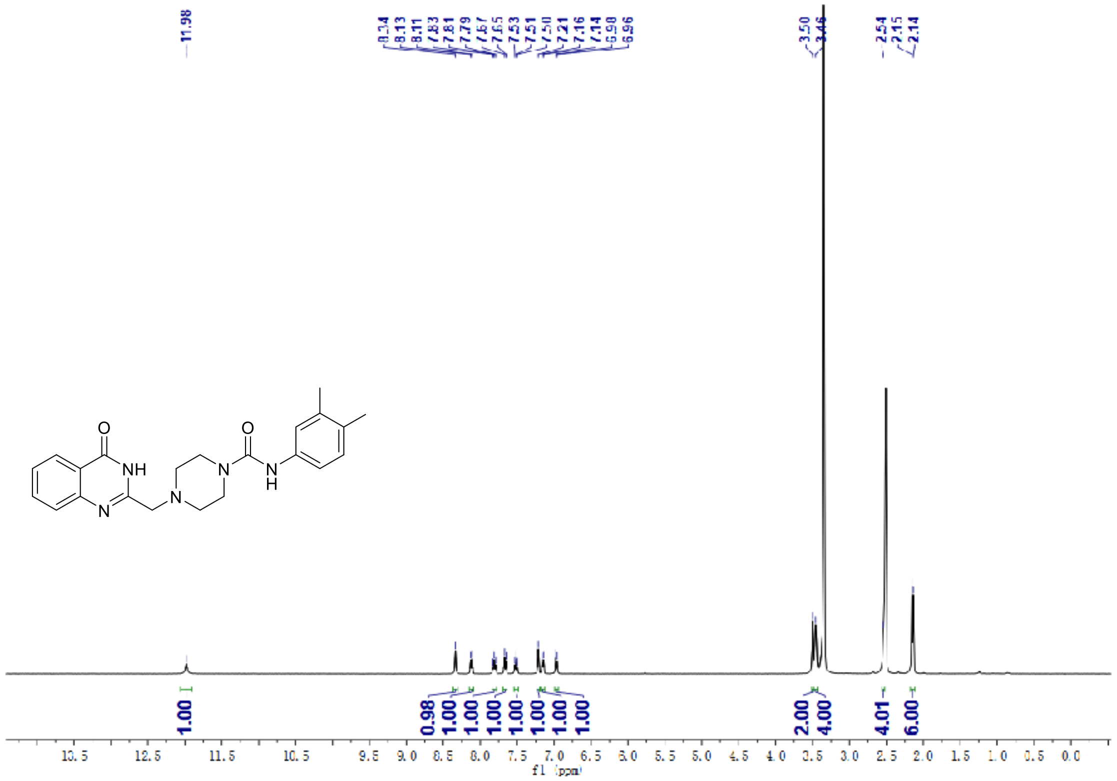

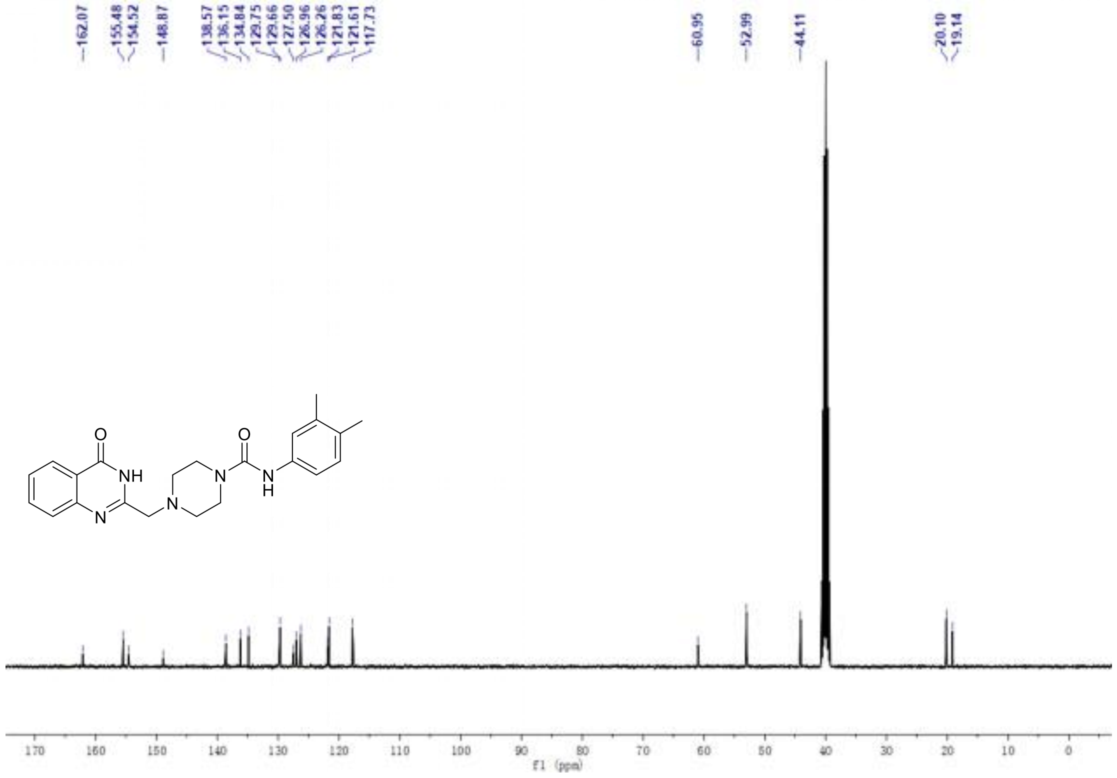

1.38 ^{1}H-NMR and ^{13}C-NMR (DMSO-d_6) spectrum of A38

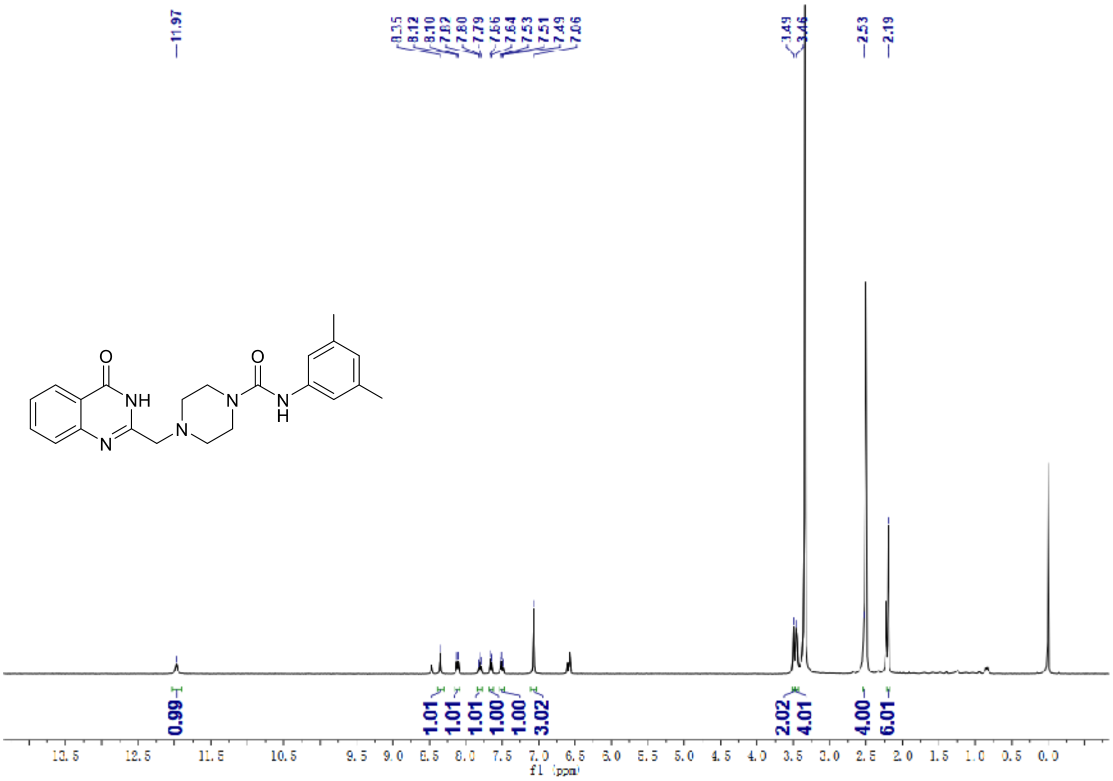

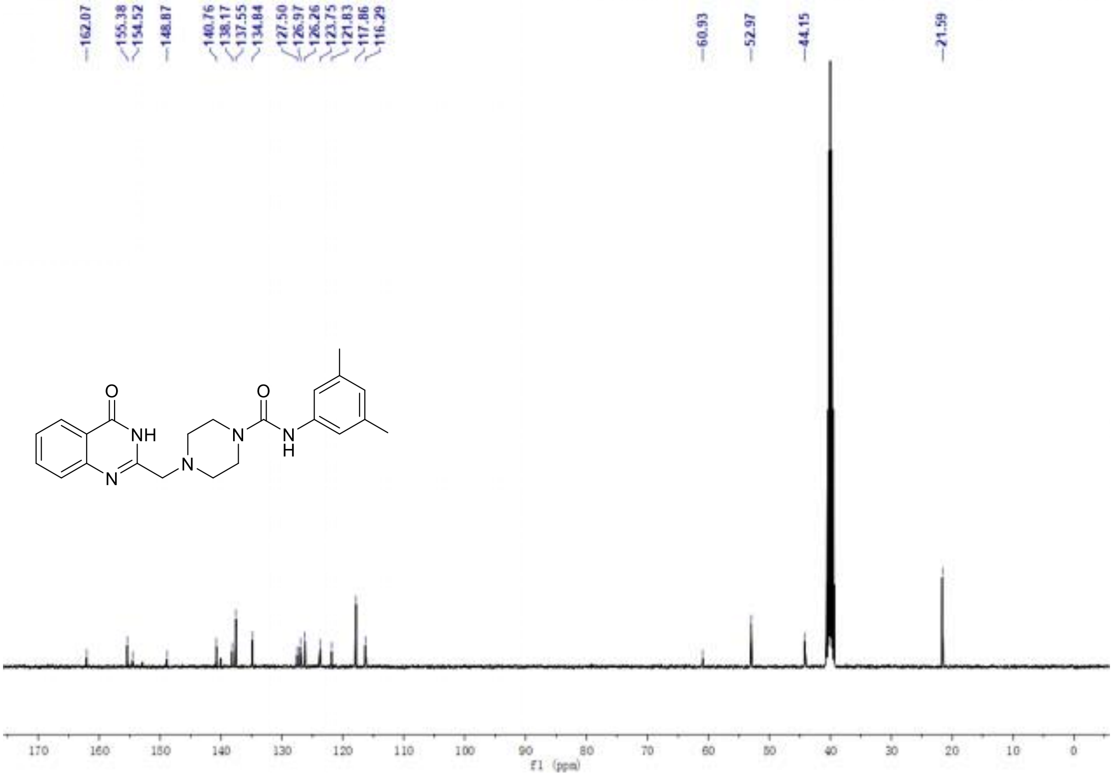

1.39 ^{1}H-NMR and ^{13}C-NMR (DMSO-d$_6$) spectrum of A39

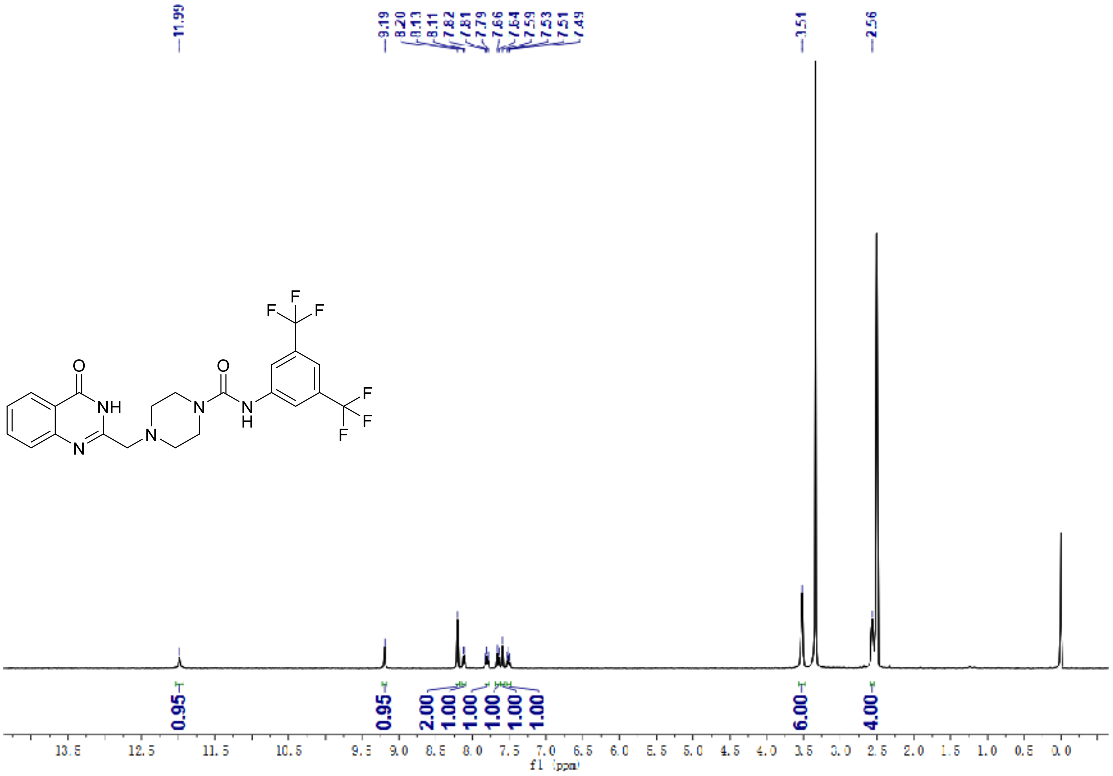

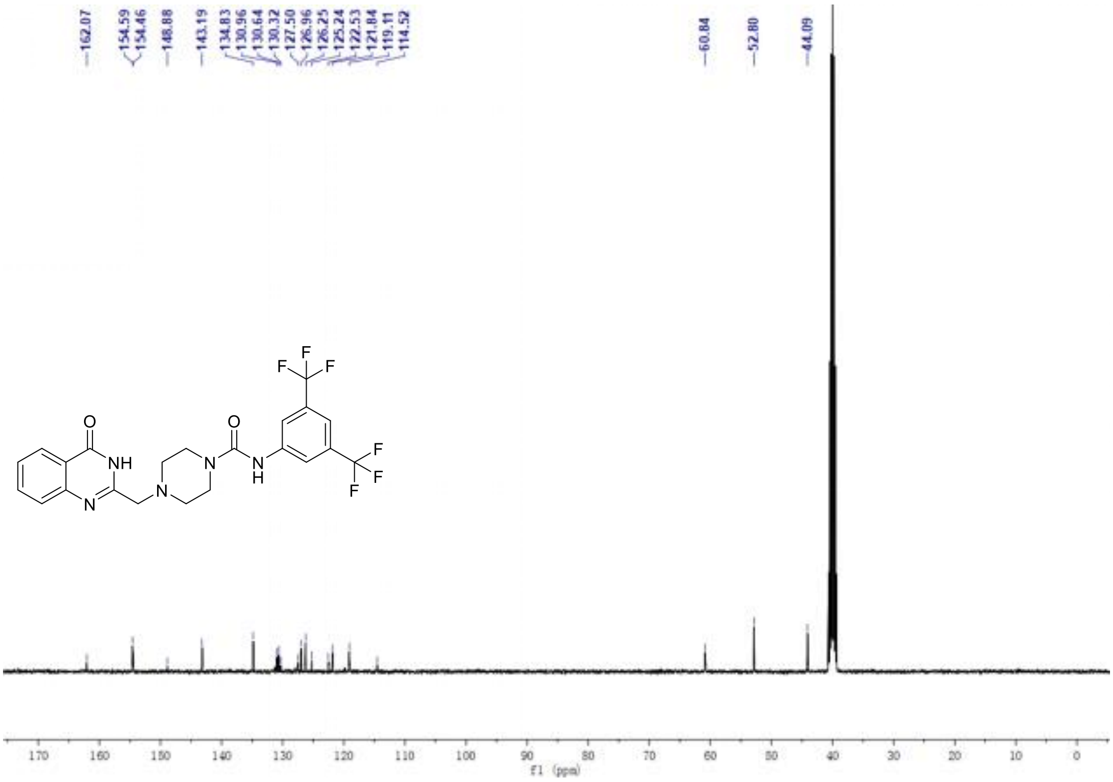

2.1 ^{1}H-NMR and ^{13}C-NMR (DMSO-d_6) spectrum of B1

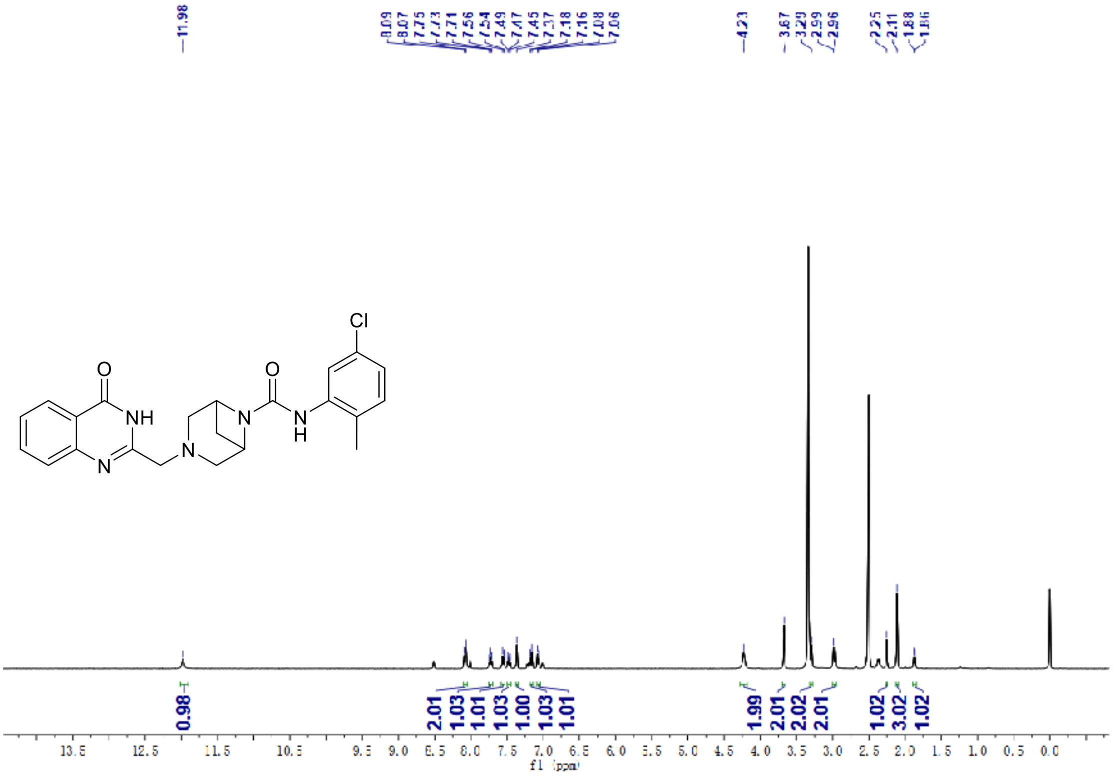

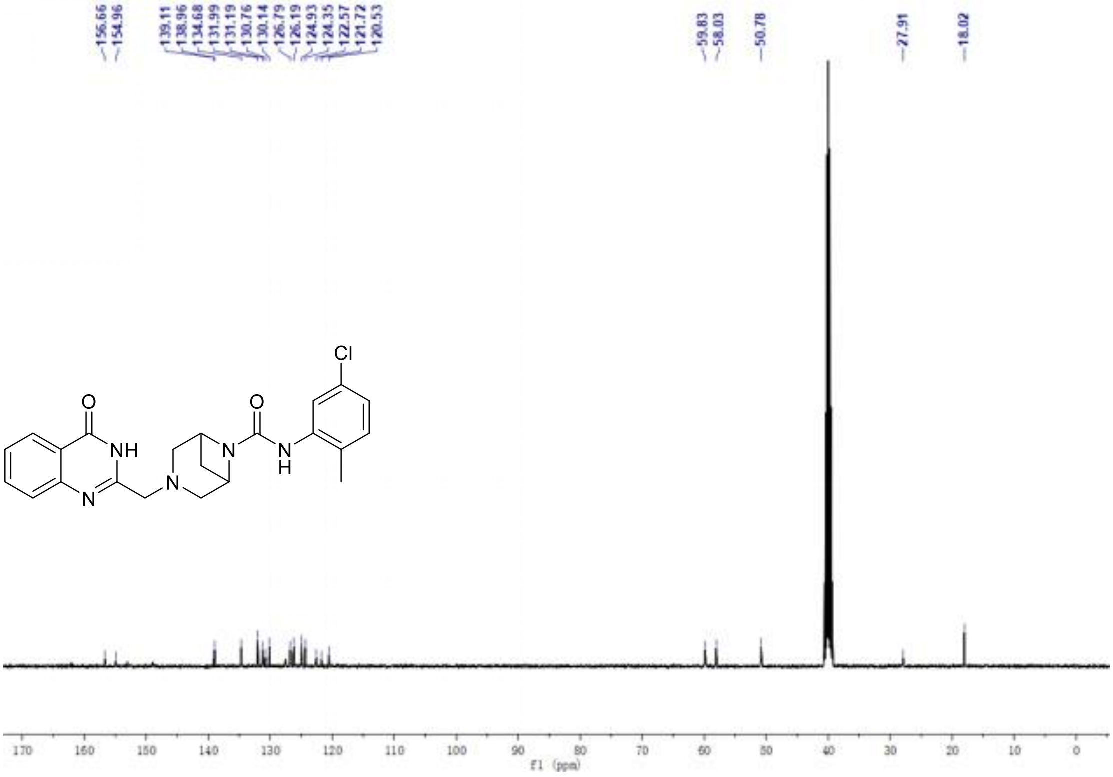

2.2 ^{1}H-NMR and ^{13}C-NMR (DMSO-d$_6$) spectrum of B2

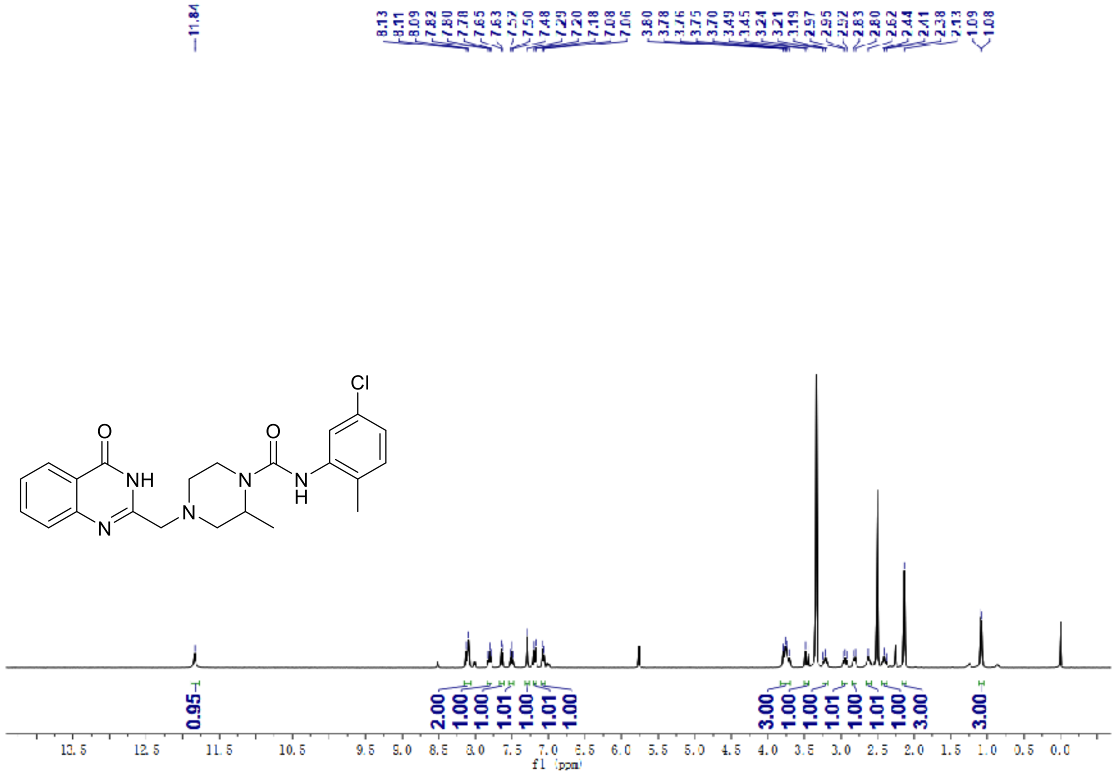

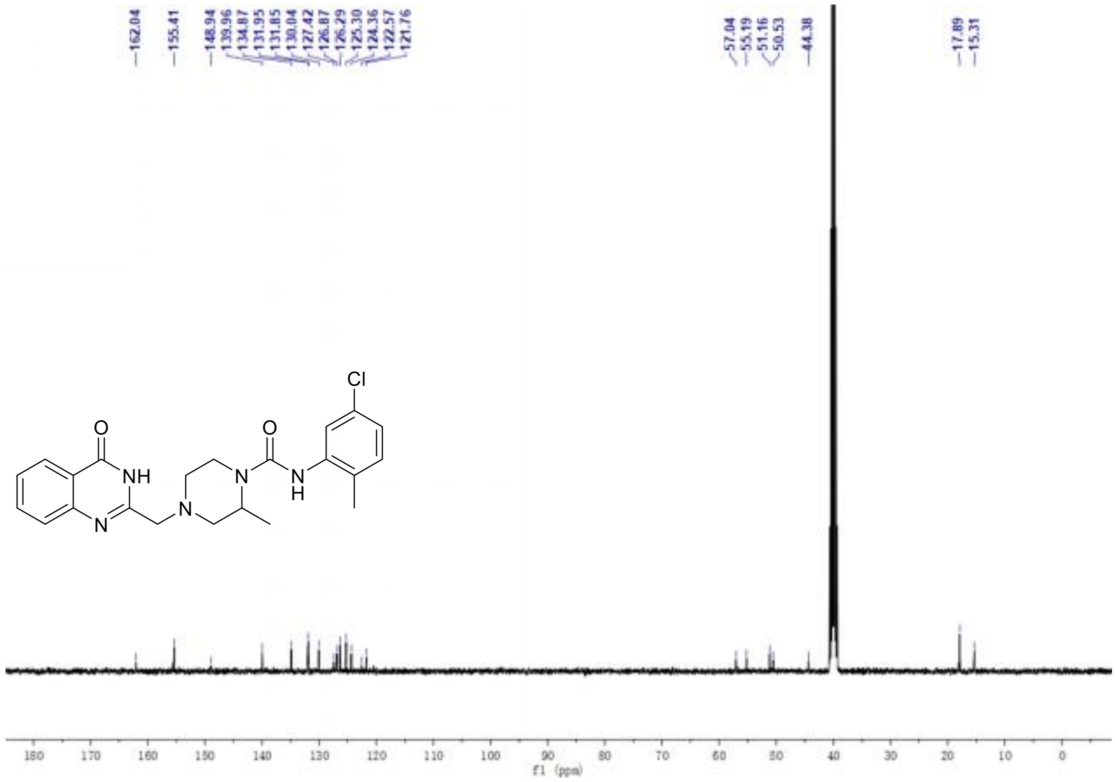

2.3 ^{1}H-NMR and ^{13}C-NMR (DMSO-d_6) spectrum of B3

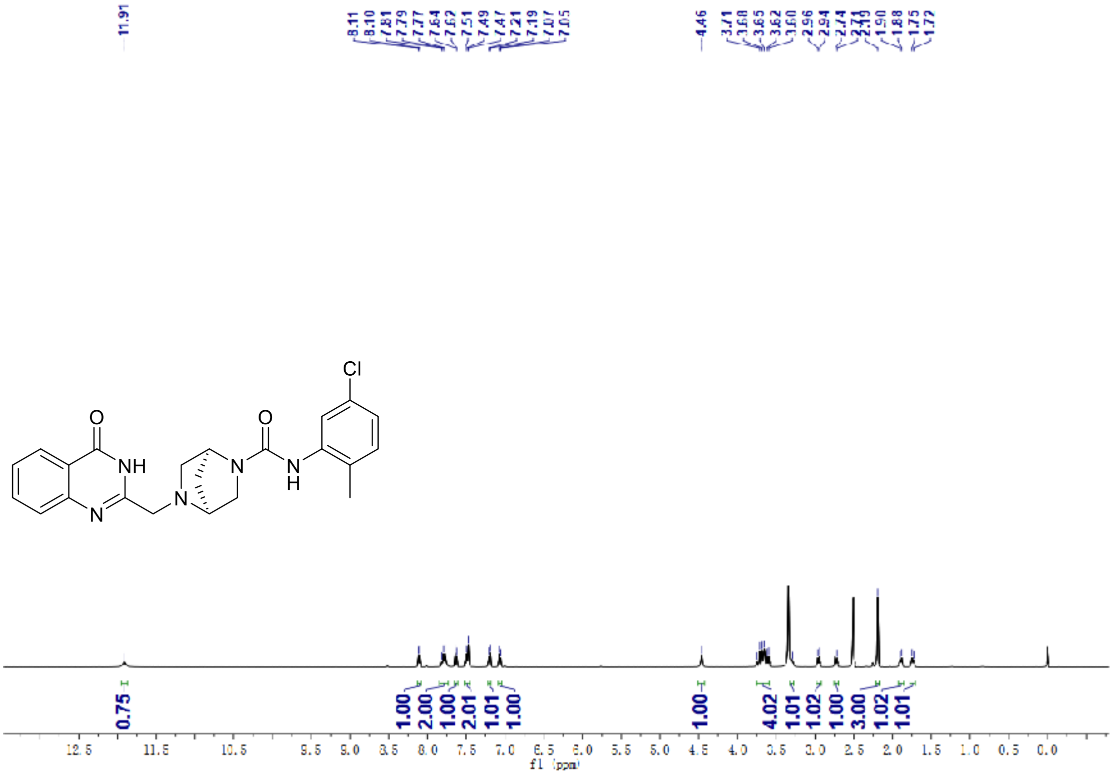

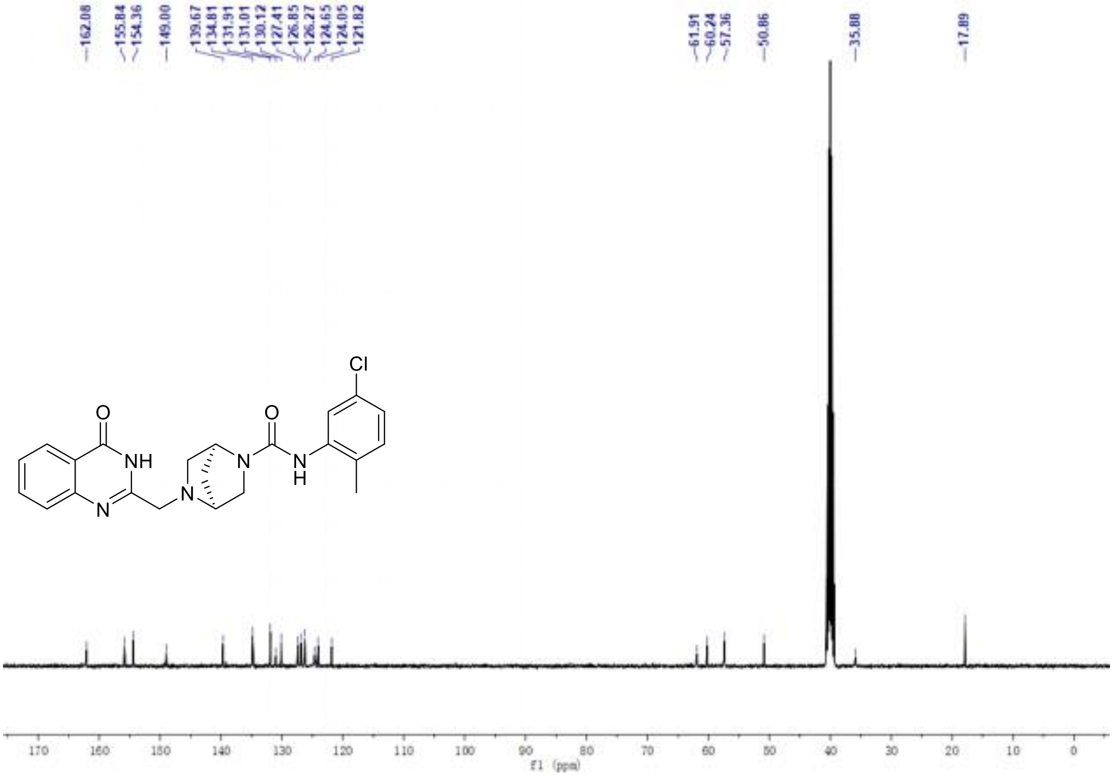

2.4 ^{1}H-NMR and ^{13}C-NMR (DMSO-d$_6$) spectrum of B4

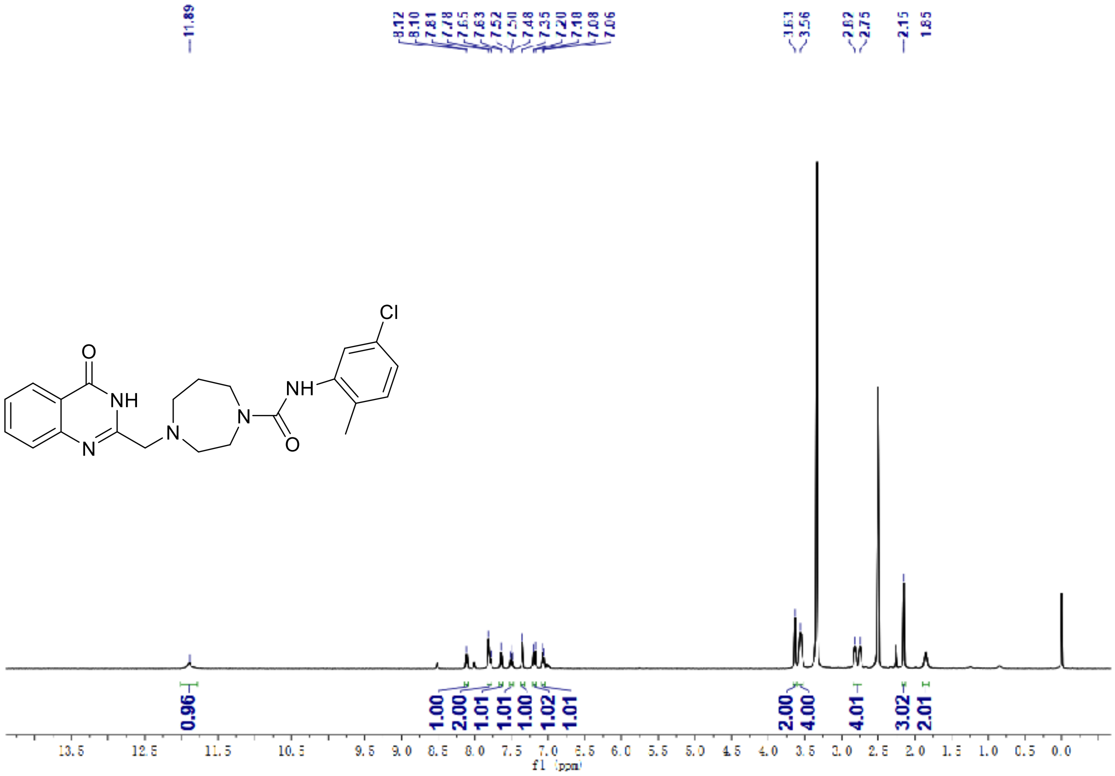

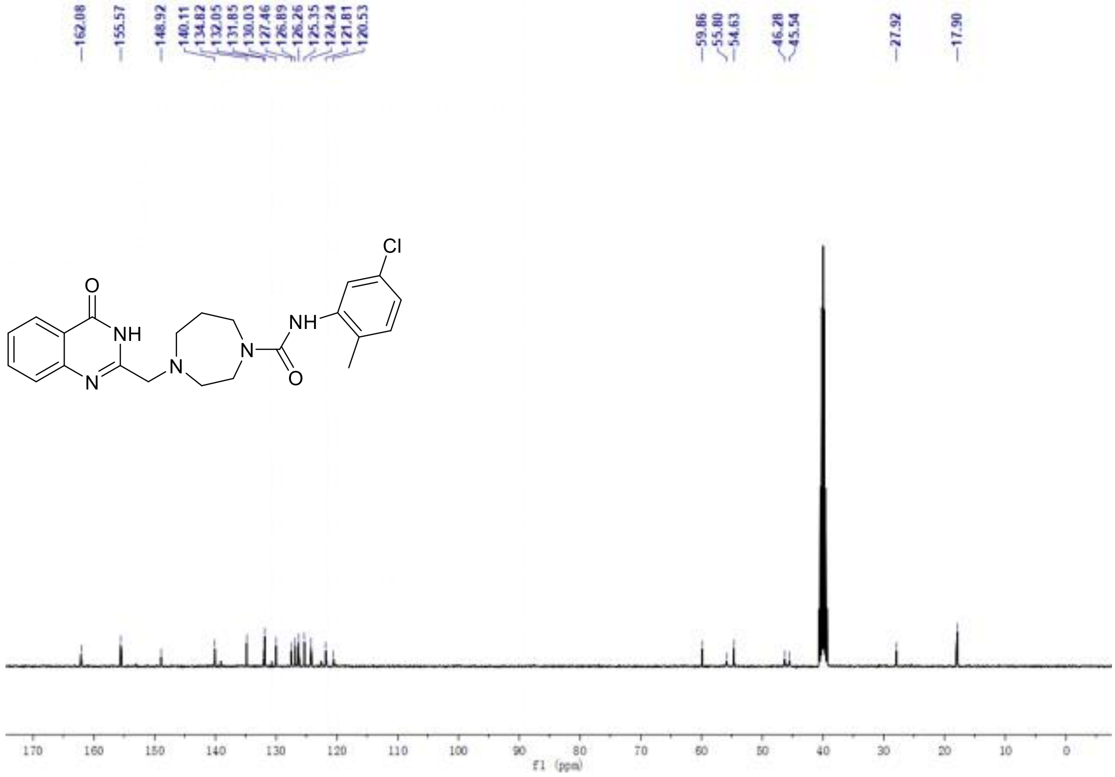

2.5 ^{1}H-NMR and ^{13}C-NMR (DMSO-d$_6$) spectrum of B5

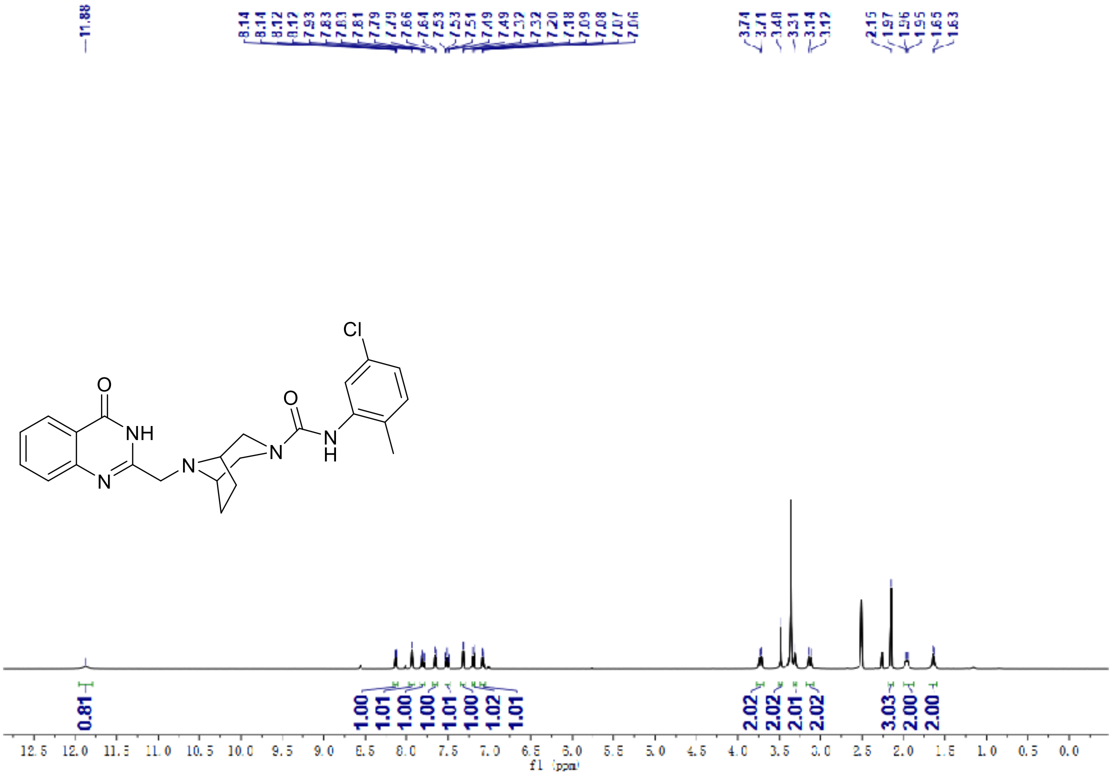

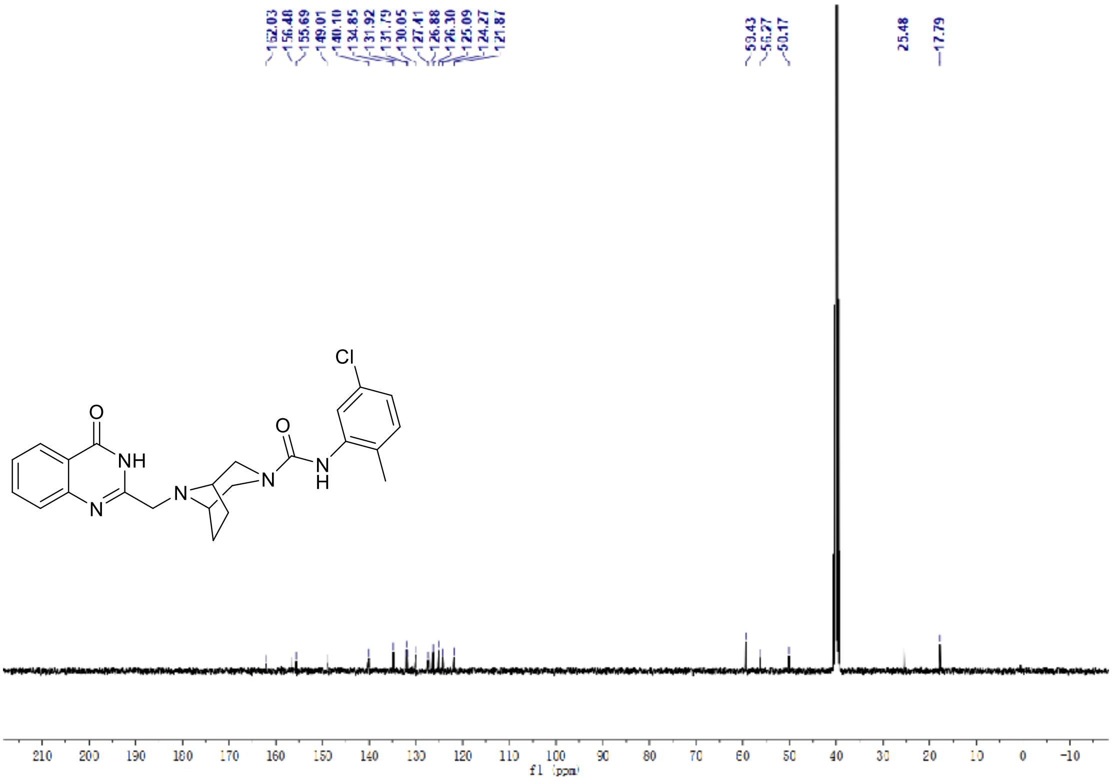

2.6 ^{1}H-NMR and ^{13}C-NMR (CDCl$_3$) spectrum of B6

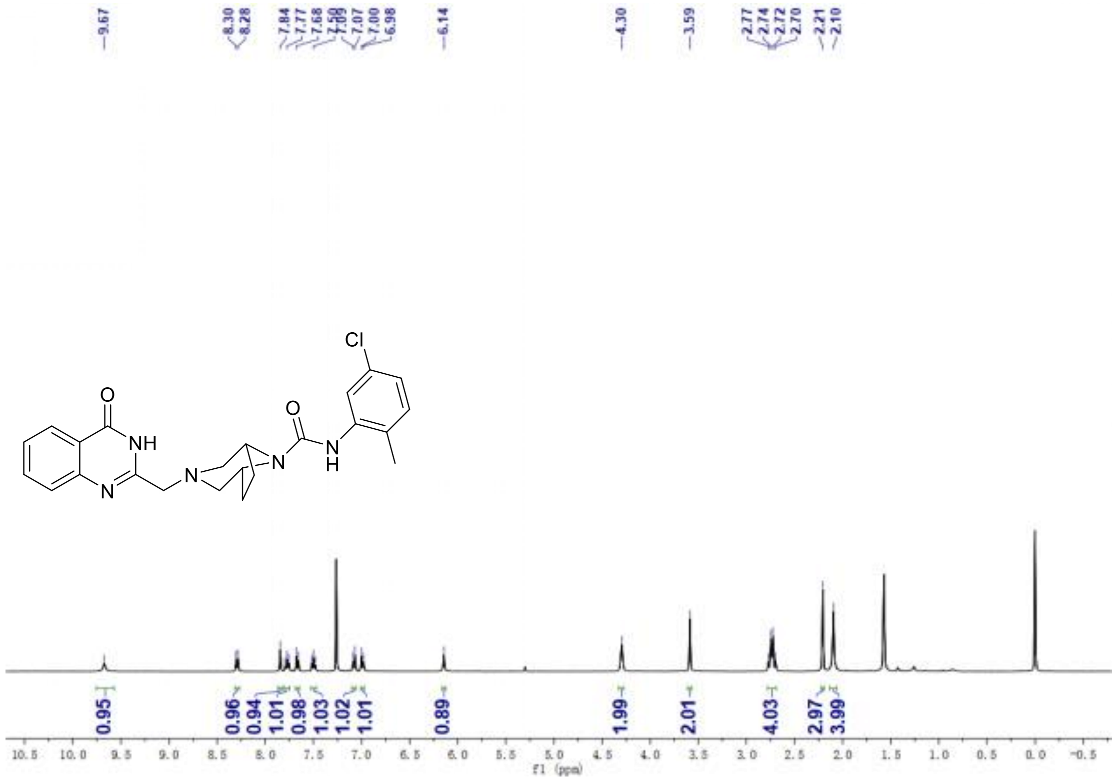

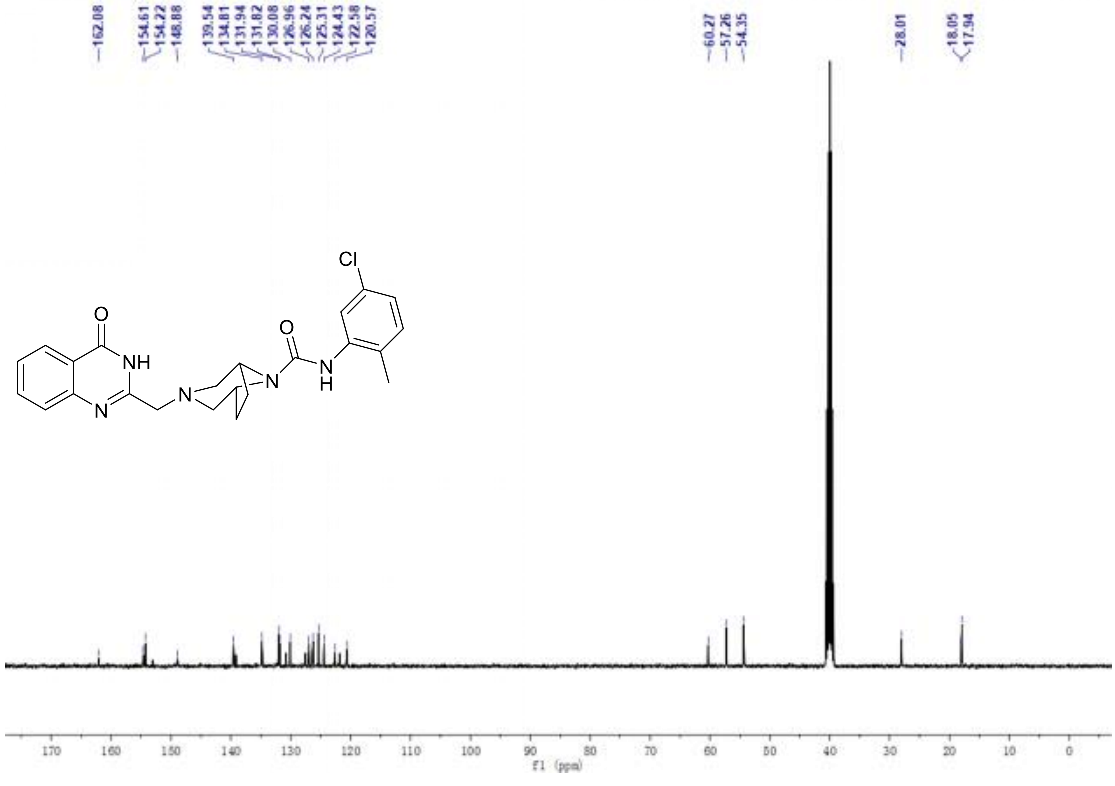

2.7 ^{1}H-NMR and ^{13}C-NMR (DMSO-d_6) spectrum of B7

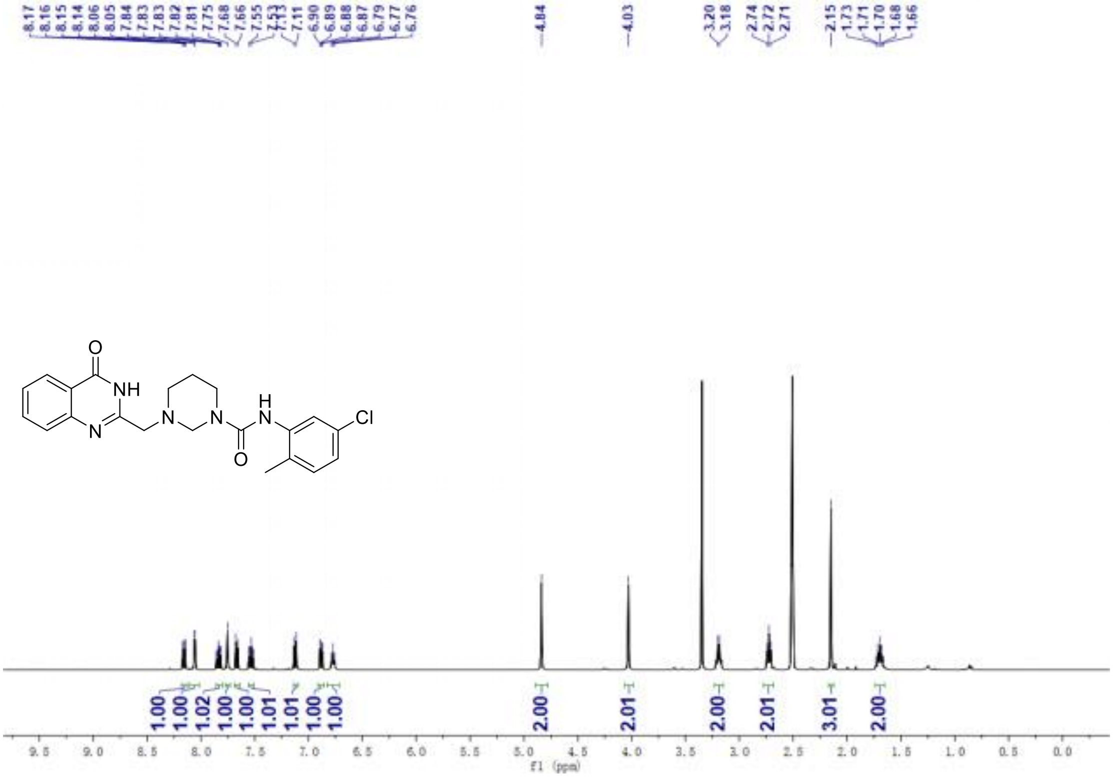

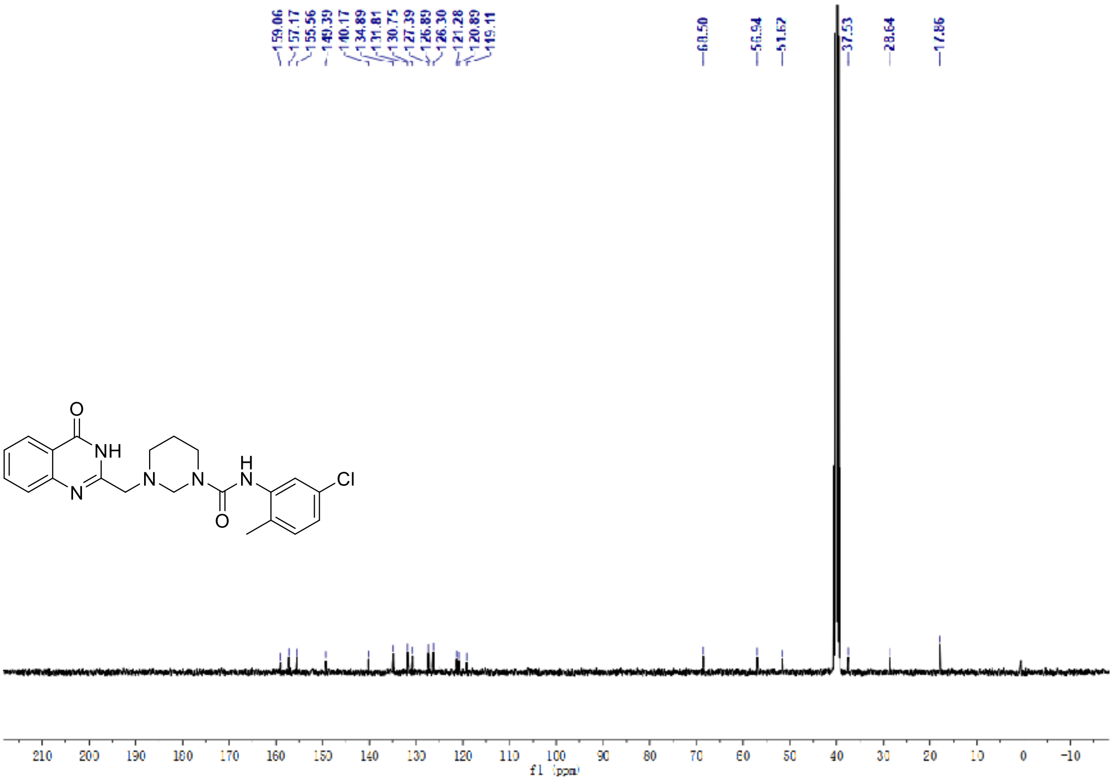

3.1 ^{1}H-NMR and ^{13}C-NMR (DMSO-d_6) spectrum of C1

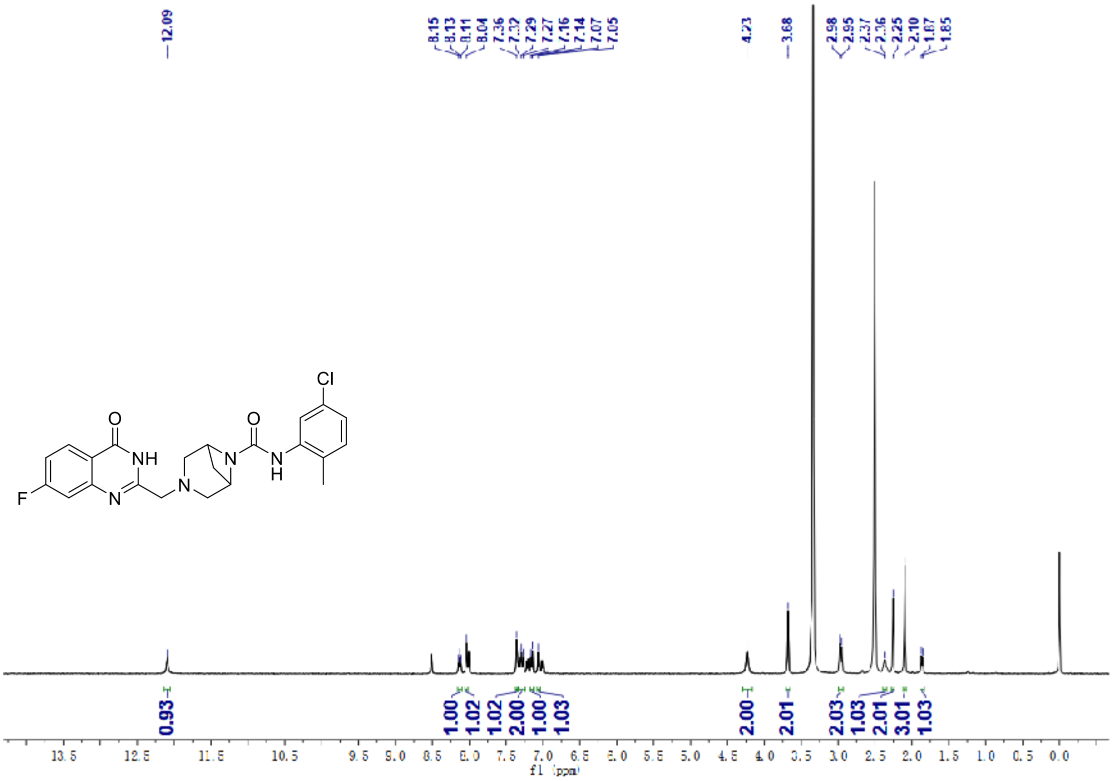

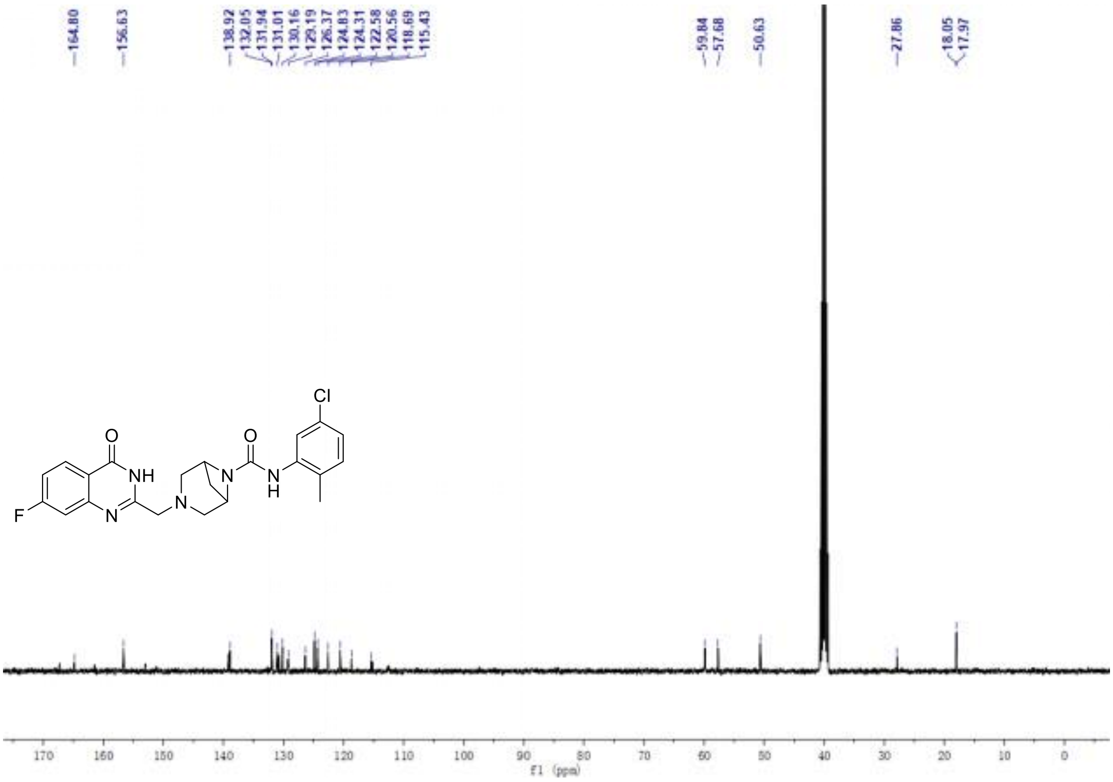

3.2 ^{1}H-NMR and ^{13}C-NMR (DMSO-d_6) spectrum of C2

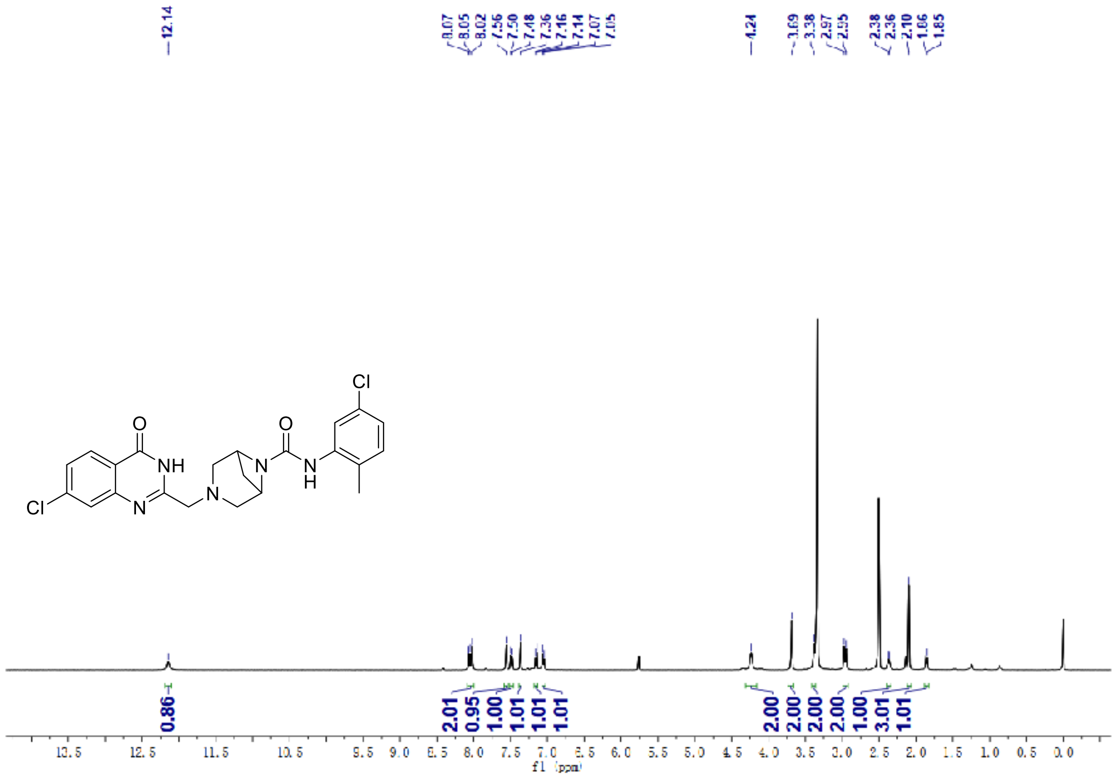

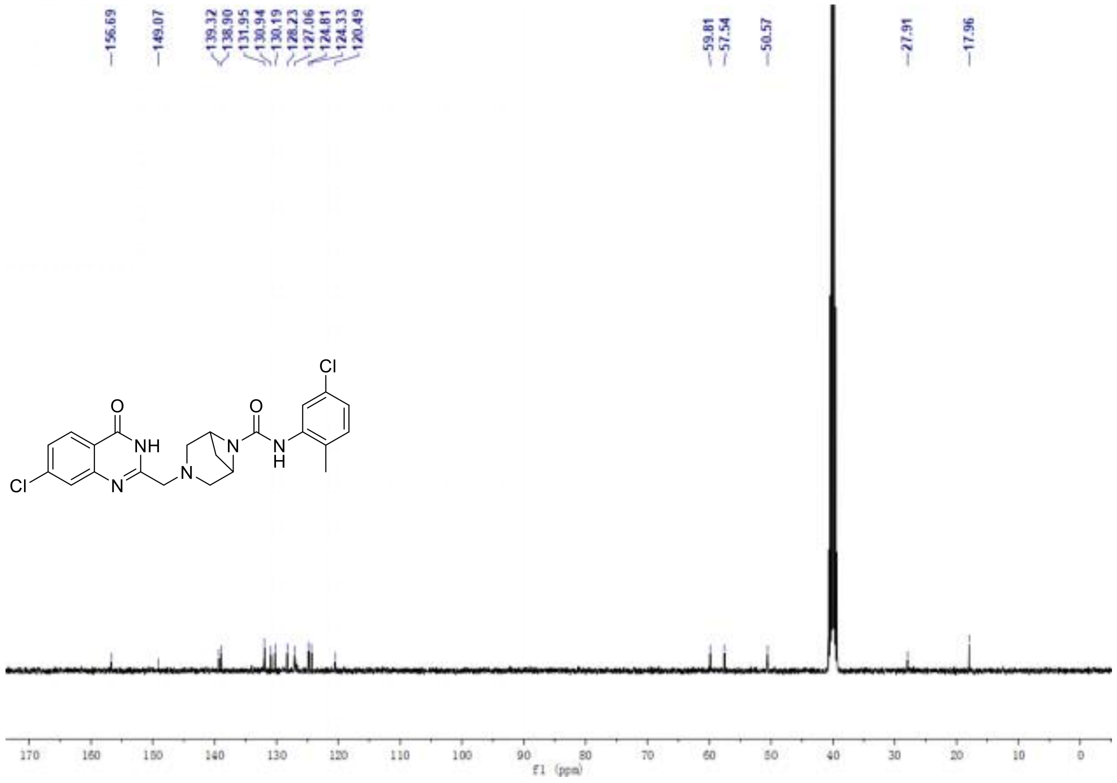

3.3 ¹H-NMR and ¹³C-NMR (DMSO-d_6) spectrum of C3

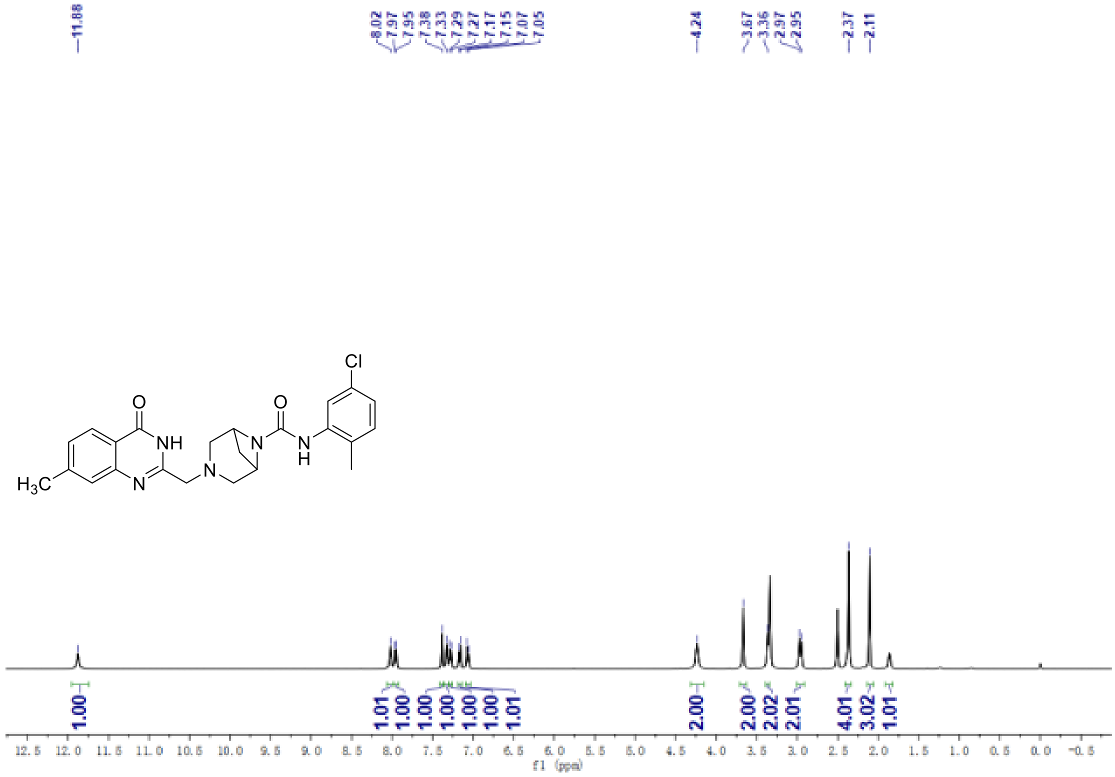

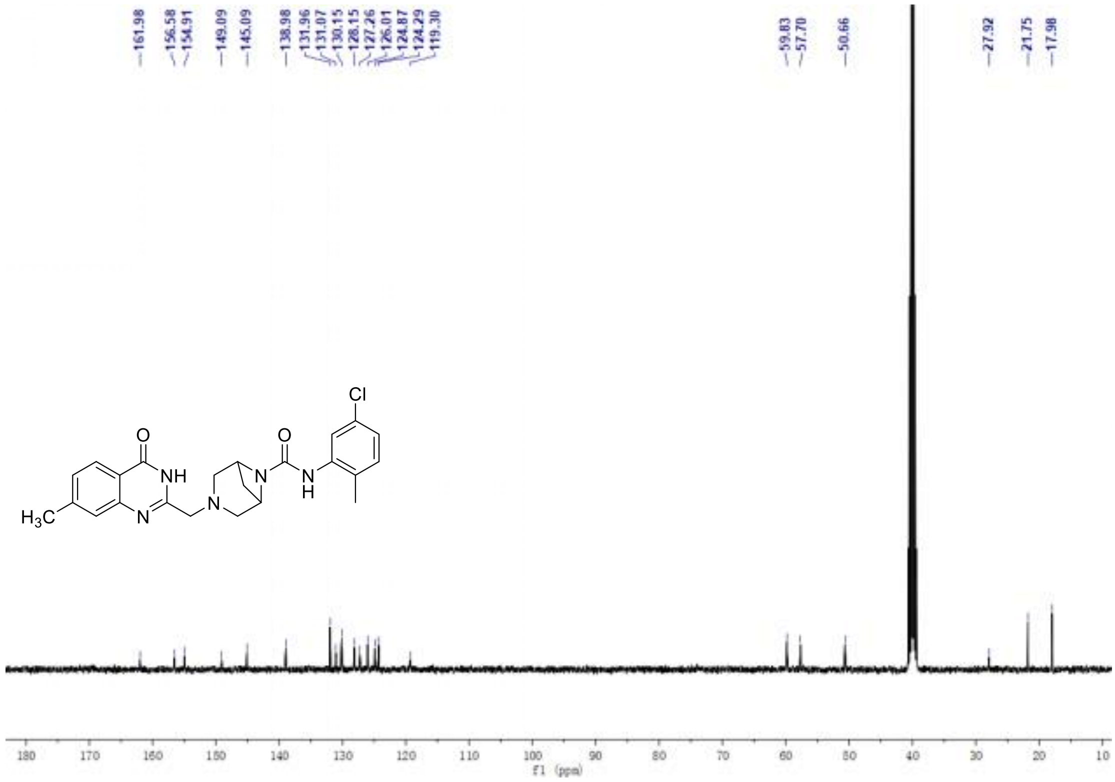

3.4 ^{1}H-NMR and ^{13}C-NMR (DMSO-d$_6$) spectrum of C4

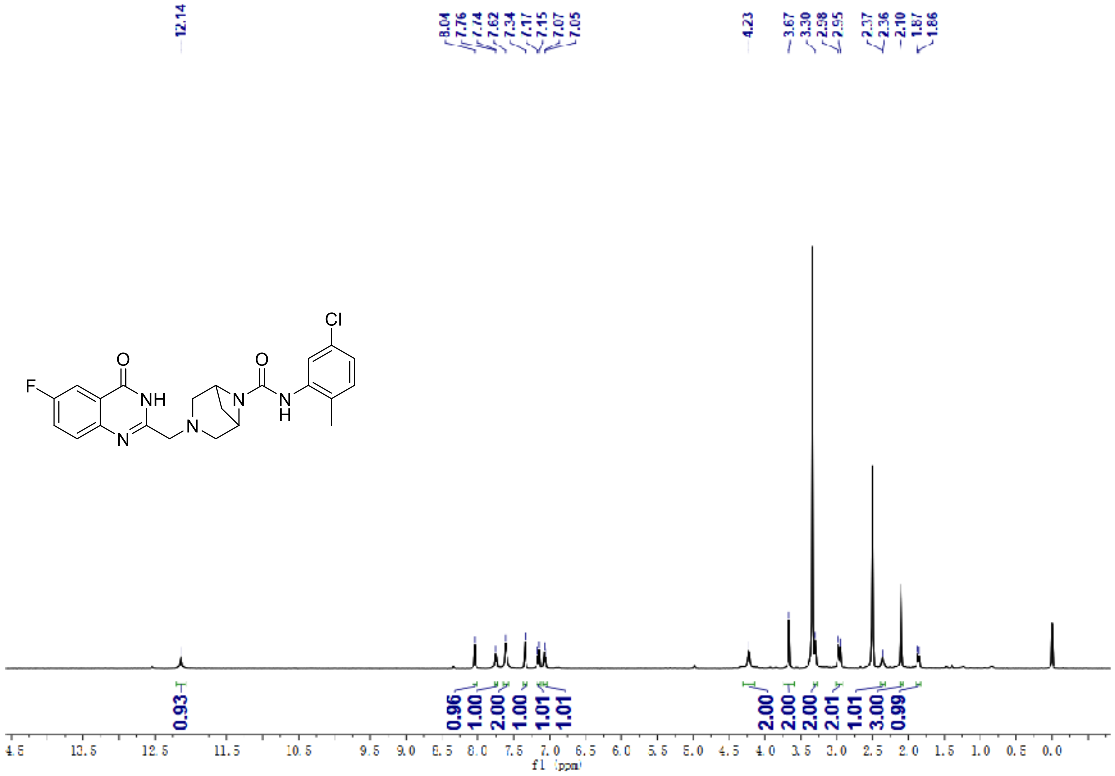

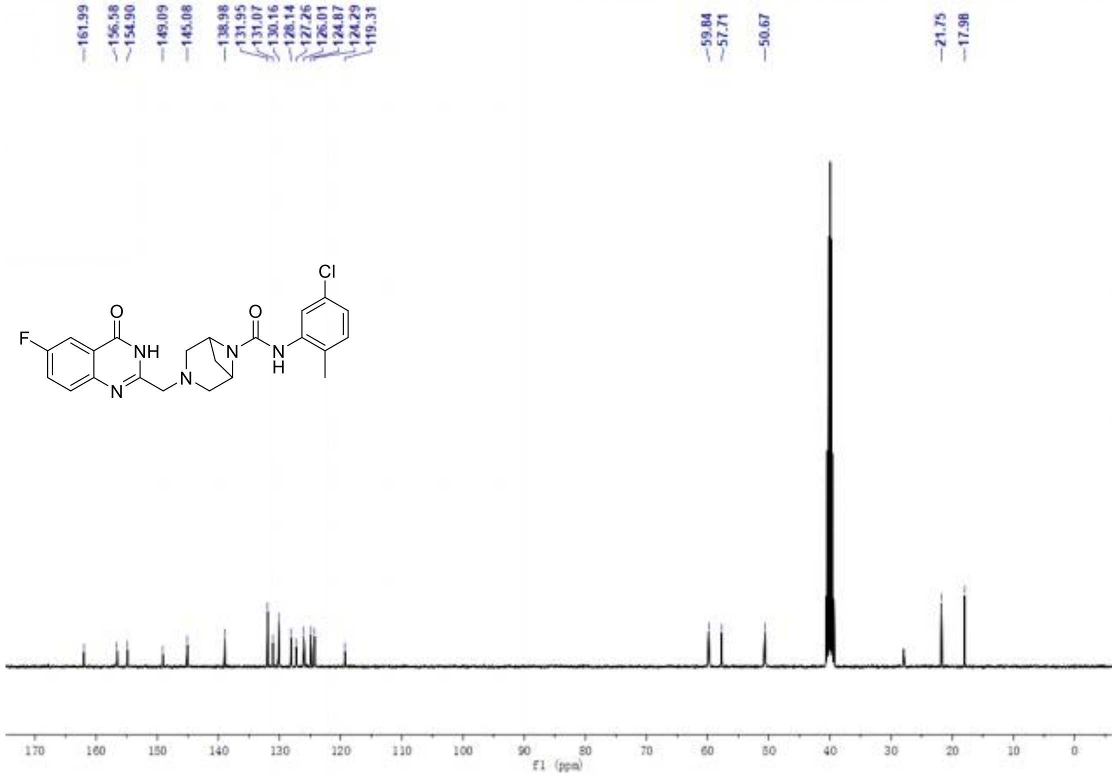

3.5 ^{1}H-NMR and ^{13}C-NMR (DMSO-d_6) spectrum of C5

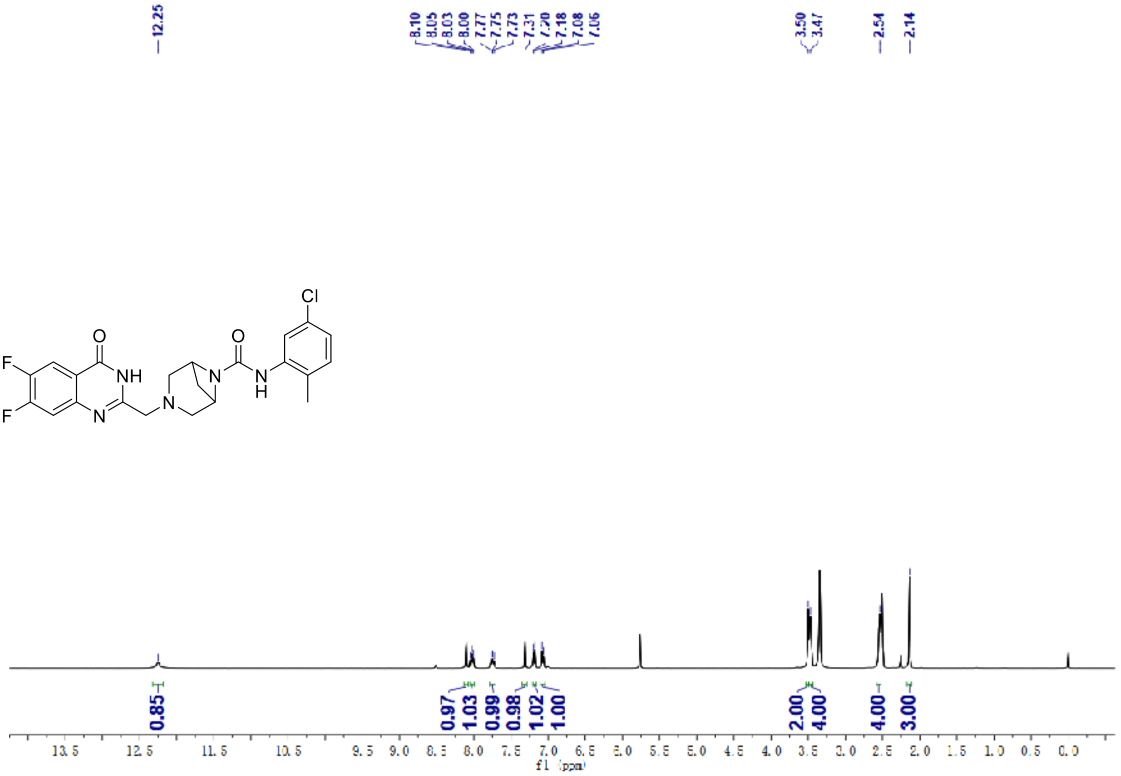

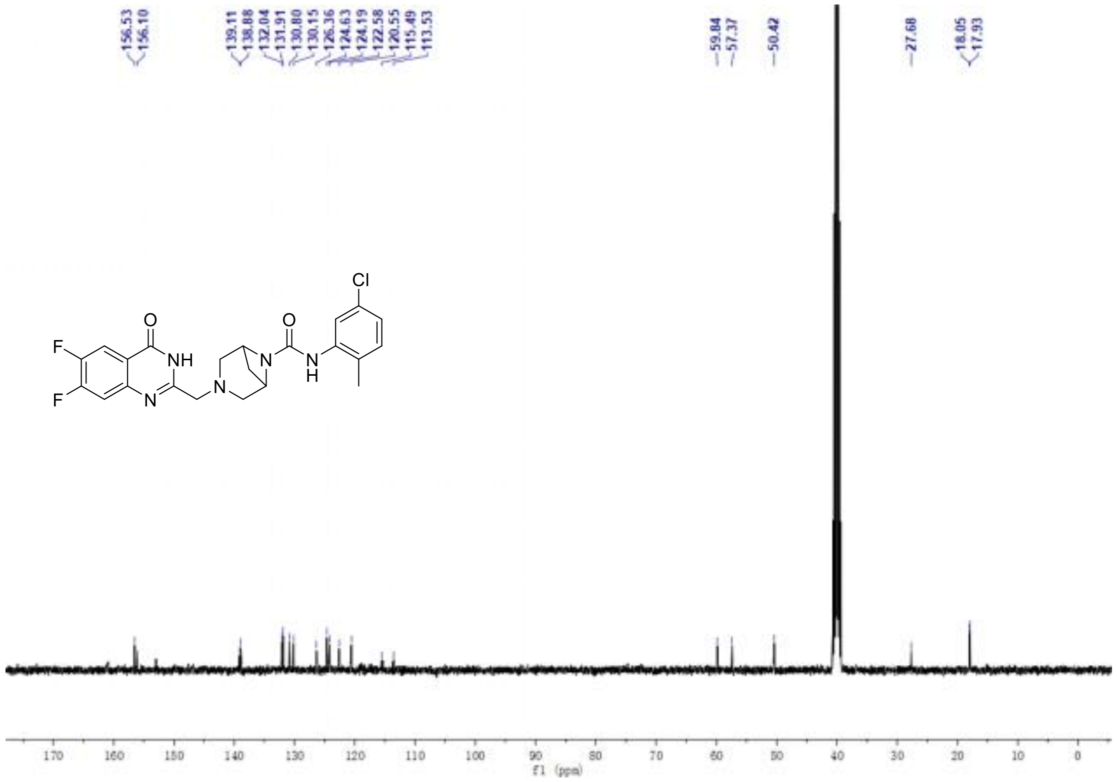

S5: Mass Spectral of synthetic compounds

S5: Mass Spectral of synthetic compounds

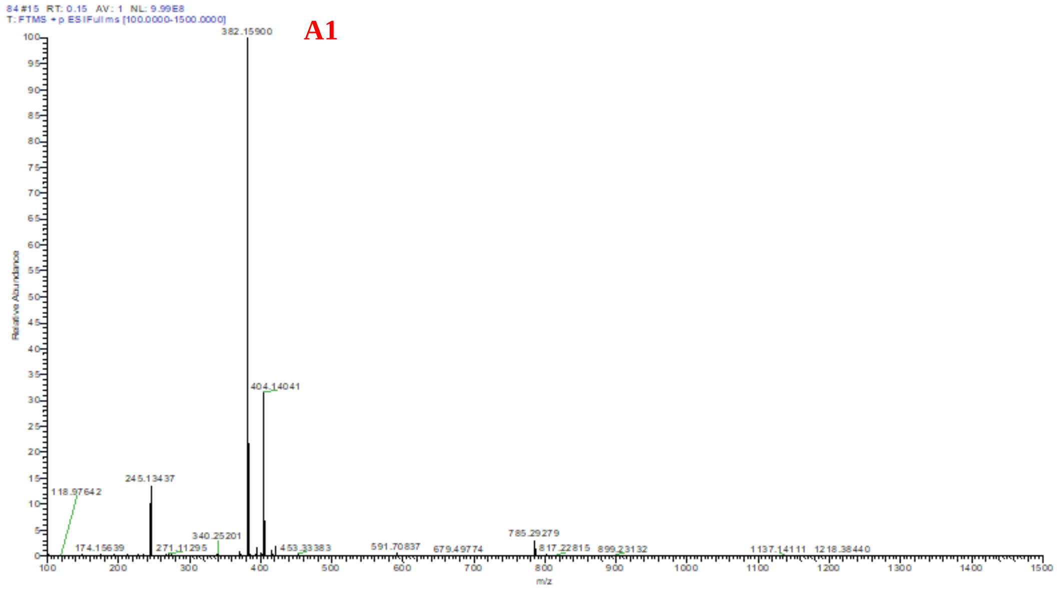

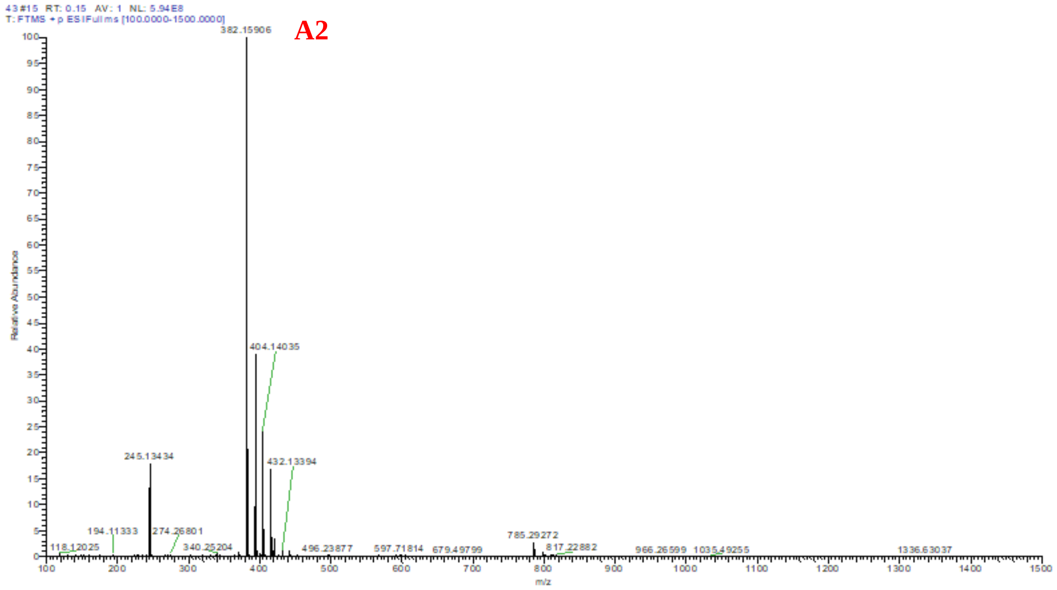

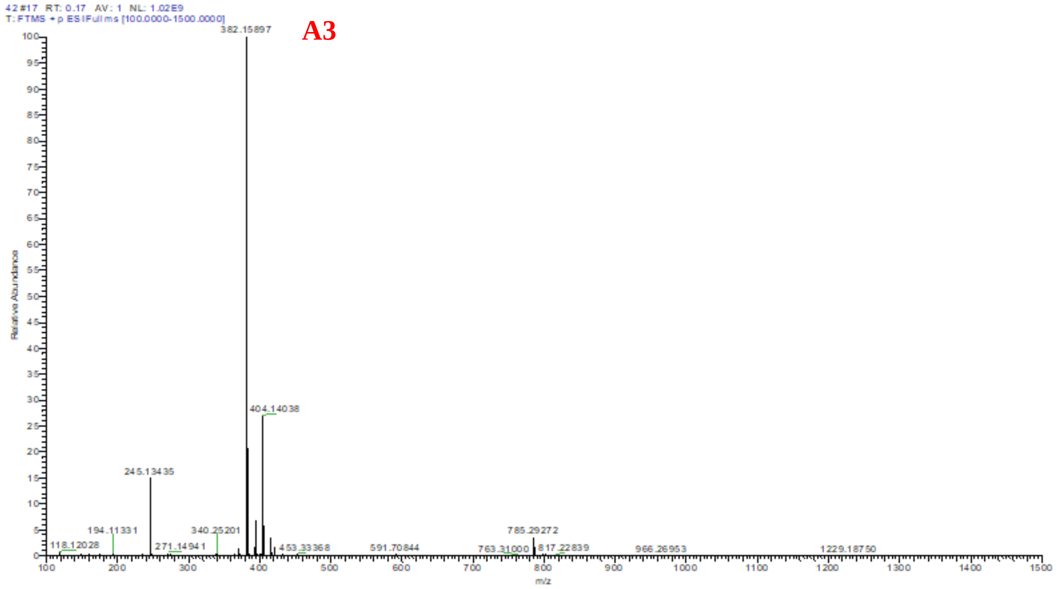
42 #17 RT: 0.17 AV: 1 NL: 1.02E9
T: FTMS + p ESI Full ms [100.0000-1500.0000]
A3
382.15897
404.14038
245.13435
194.11331
340.25201
785.29272
118.12028
271.14941
453.33368
591.70844
763.31000
817.22839
966.26953
1229.18750
Relative Abundance
m/z

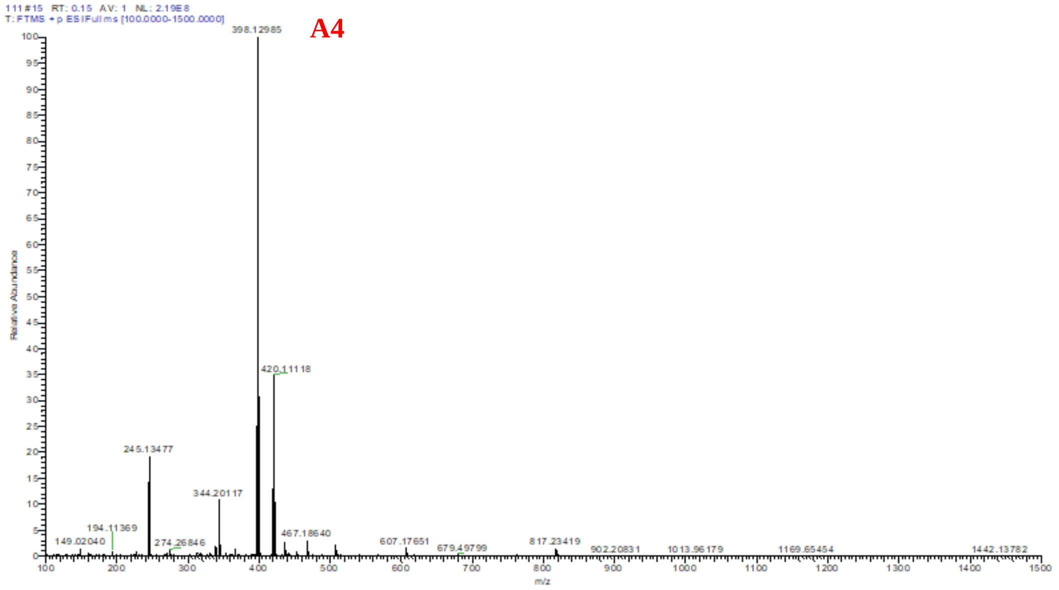
111 #15 RT: 0.15 AV: 1 NL: 2.19E8
T: FTMS + p ESI Full ms [100.0000-1500.0000]
A4
398.12985
420.11118
245.13477
344.20117
194.11369
149.02040
274.26846
467.18640
607.17651
679.49799
817.23419
902.20831
1013.96179
1169.65454
1442.13782
Relative Abundance
m/z

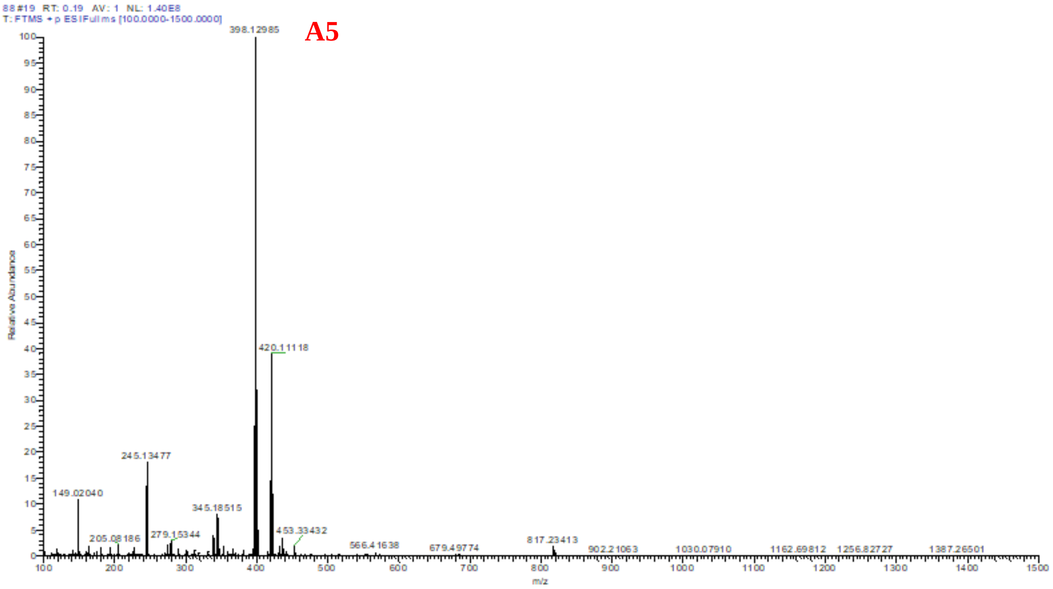
88 #19 RT: 0.19 AV: 1 NL: 1.40E8
T: FTMS + p ESI Full ms [100.0000-1500.0000]
A5
398.12985
420.11118
245.13477
149.02040
345.18515
453.33432
205.08186
279.15344
566.41638
679.49774
817.23413
902.21063
1030.07910
1162.69812
1256.82727
1387.26501
Relative Abundance
m/z

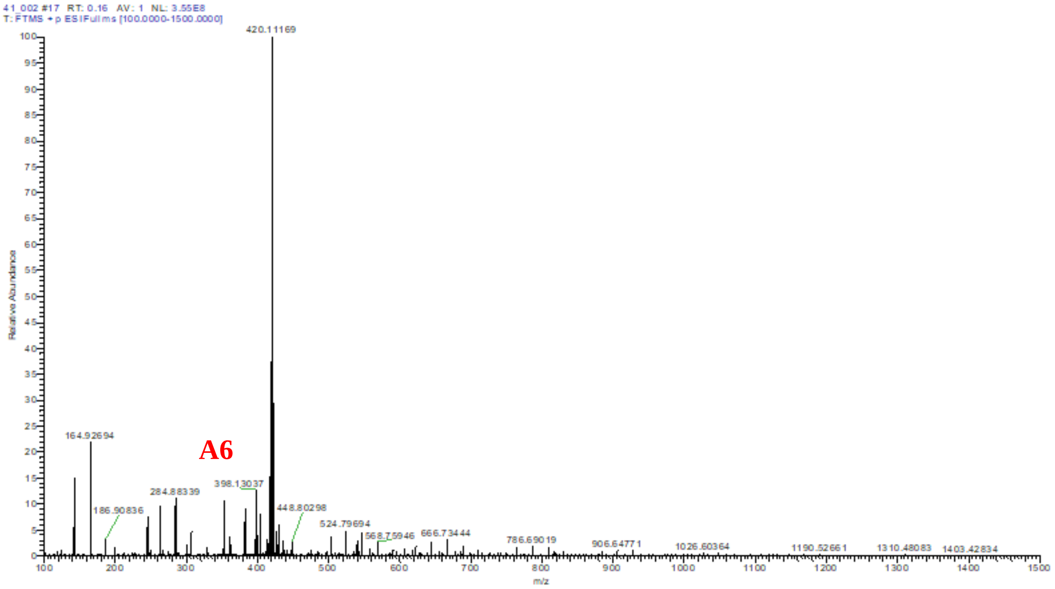
41_002 #17 RT: 0.16 AV: 1 NL: 3.55E8
T: FTMS + p ESI Full ms [100.0000-1500.0000]
420.11169
A6
164.92694
284.88339
398.13037
186.90836
448.80298
524.79694
568.75946
666.73444
786.69019
906.64771
1026.60364
1190.52661
1310.48083
1403.42834
Relative Abundance
m/z

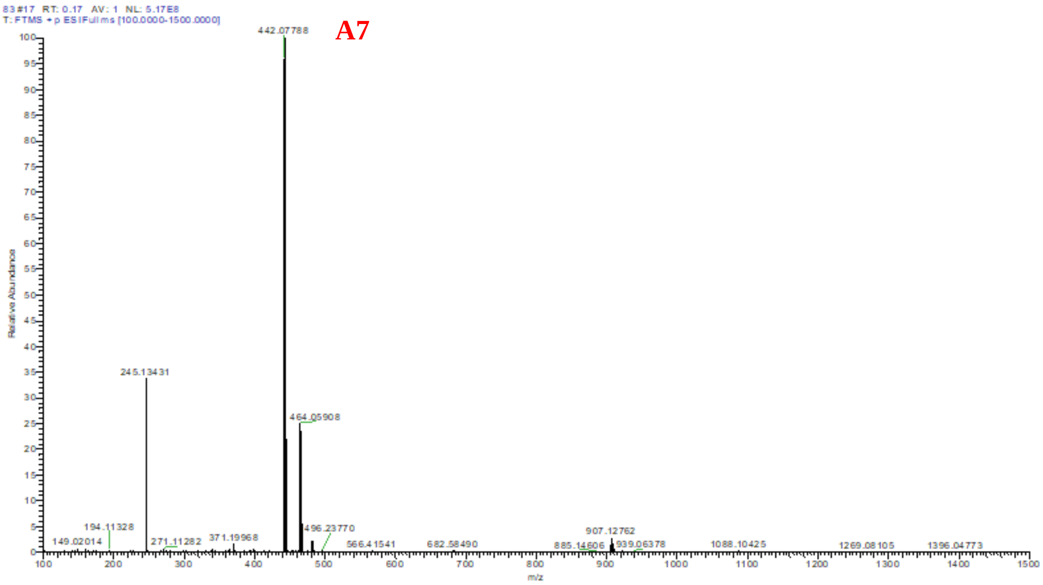
83 #17 RT: 0.17 AV: 1 NL: 5.17E8
T: FTMS + p ESI Full ms [100.0000-1500.0000]
442.07788
A7
245.13431
464.05908
194.11328
496.23770
149.02014
271.11282
371.19968
566.41541
682.58490
907.12762
885.14606
939.06378
1088.10425
1269.08105
1396.04773
Relative Abundance
m/z

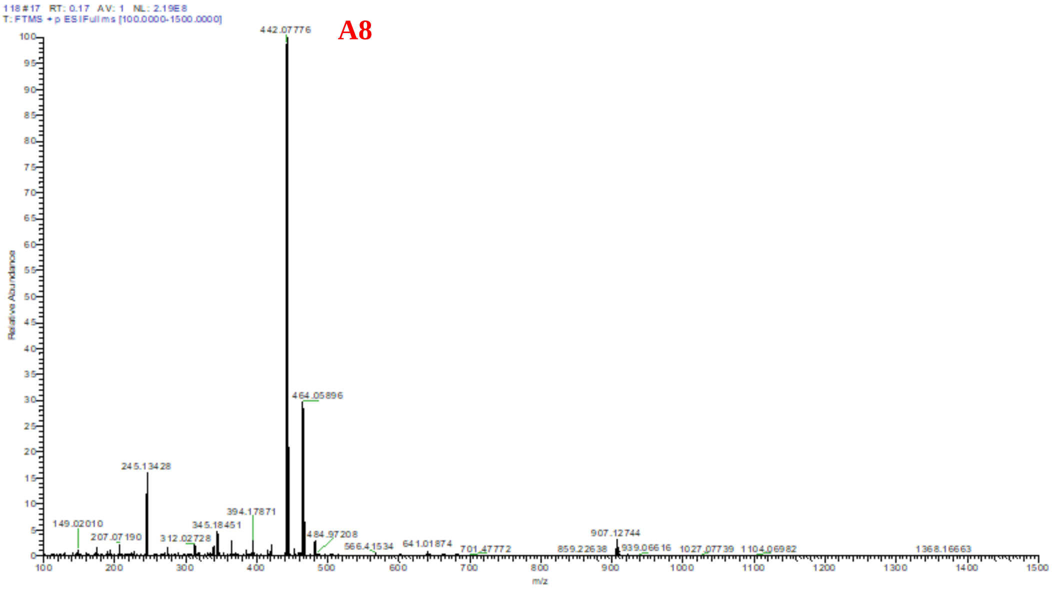
118 #17 RT: 0.17 AV: 1 NL: 2.19E8
T: FTMS + p ESI Full ms [100.0000-1500.0000]
442.07776
A8
464.05896
245.13428
394.17871
149.02010
345.18451
207.07190
312.02728
484.97208
566.41534
641.01874
701.47772
907.12744
859.22638
939.06616
1027.07739
1104.06982
1368.16663
Relative Abundance
m/z

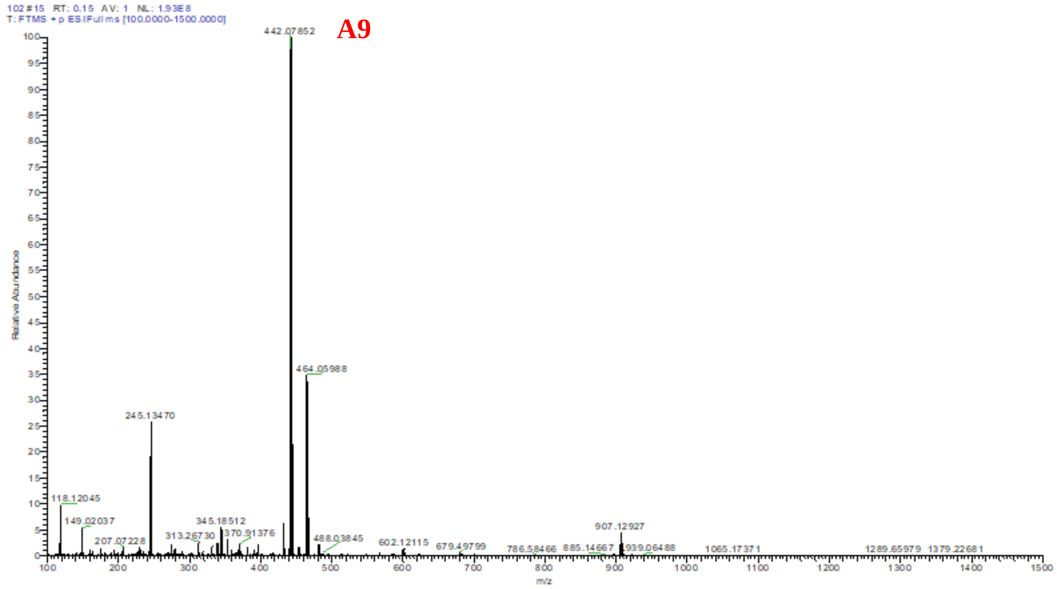
102 #15 RT: 0.15 AV: 1 NL: 1.93E8
T: FTMS + p ESI Full ms [100.0000-1500.0000]
A9
442.07852
464.05988
245.13470
118.12045
149.02037
207.07228
313.26730
345.18512
370.91376
488.03845
602.12115
679.49799
786.58466
885.14667
907.12927
939.06488
1065.17371
1289.65979
1379.22681
Relative Abundance
m/z

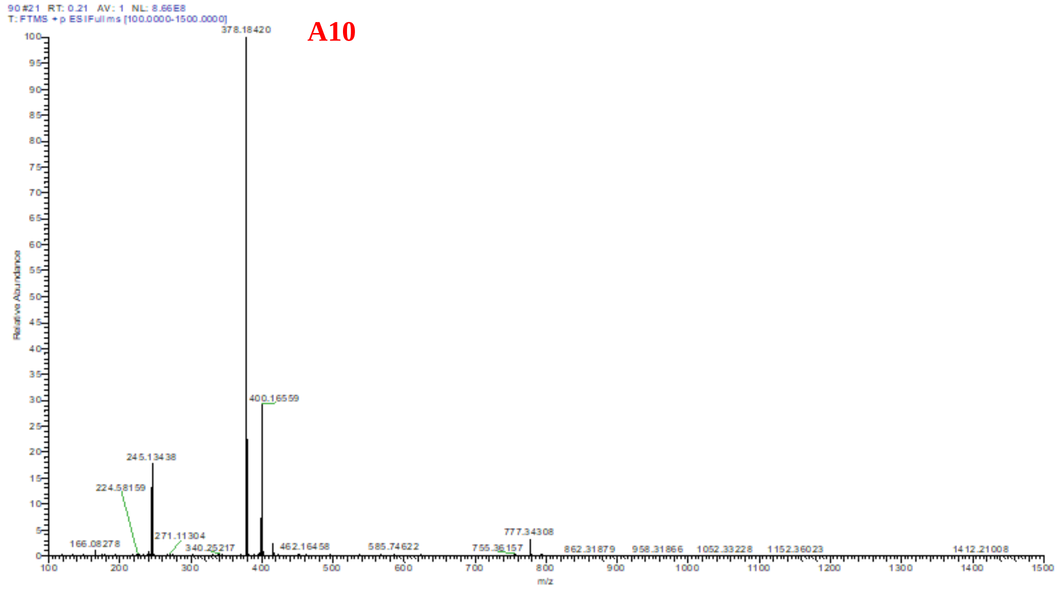
90 #21 RT: 0.21 AV: 1 NL: 8.66E8
T: FTMS + p ESI Full ms [100.0000-1500.0000]
A10
378.18420
400.16559
245.13438
224.58159
166.08278
271.11304
340.25217
462.16458
585.74622
755.36157
777.34308
862.31879
958.31866
1052.33228
1152.36023
1412.21008
Relative Abundance
m/z

A11

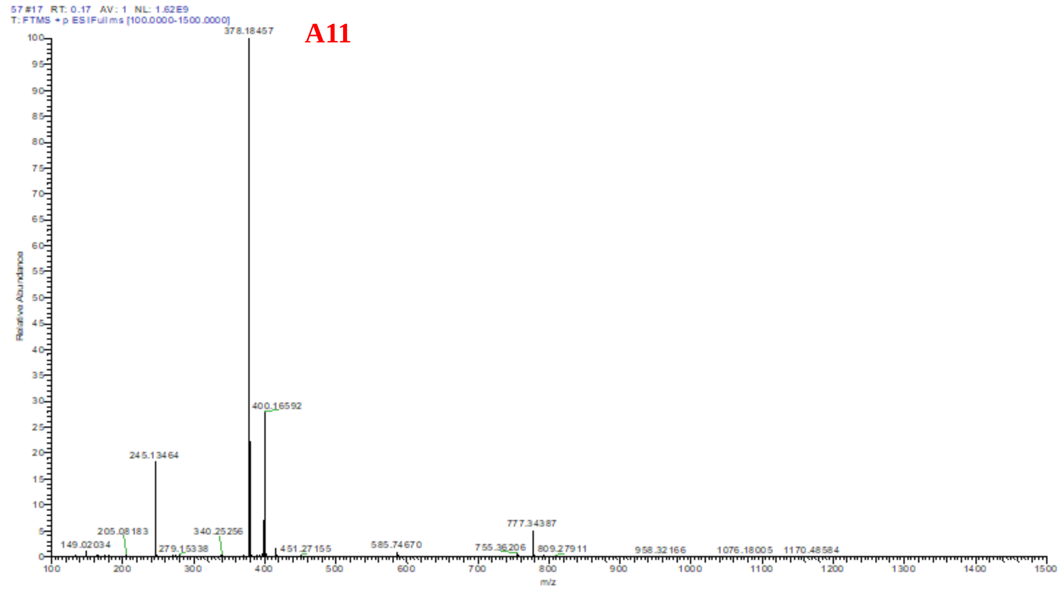
57 #17 RT: 0.17 AV: 1 NL: 1.62E9
T: FTMS + p ESI Full ms [100.0000-1500.0000]
378.18457
400.16592
245.13464
777.34387
205.08183
340.25256
149.02034
279.15338
451.27155
585.74670
755.36206
809.27911
958.32166
1076.18005
1170.48584
Relative Abundance
m/z

A12

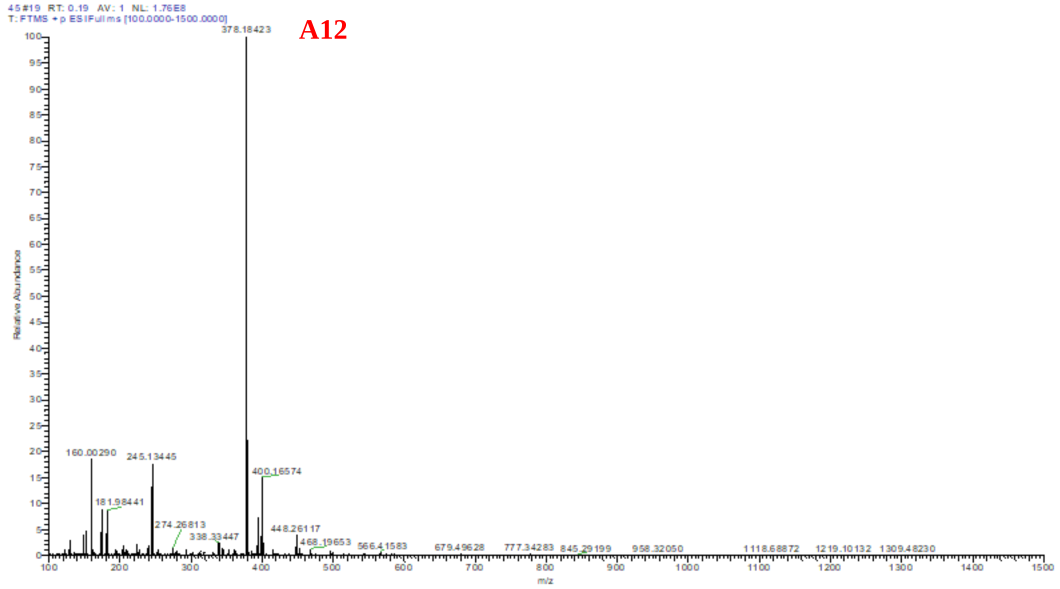
45 #19 RT: 0.19 AV: 1 NL: 1.76E8
T: FTMS + p ESI Full ms [100.0000-1500.0000]
378.18423
160.00290
245.13445
400.16574
181.98441
274.26813
448.26117
338.33447
468.19653
566.41583
679.49628
777.34283
845.29199
958.32050
1118.68872
1219.10132
1309.48230
Relative Abundance
m/z

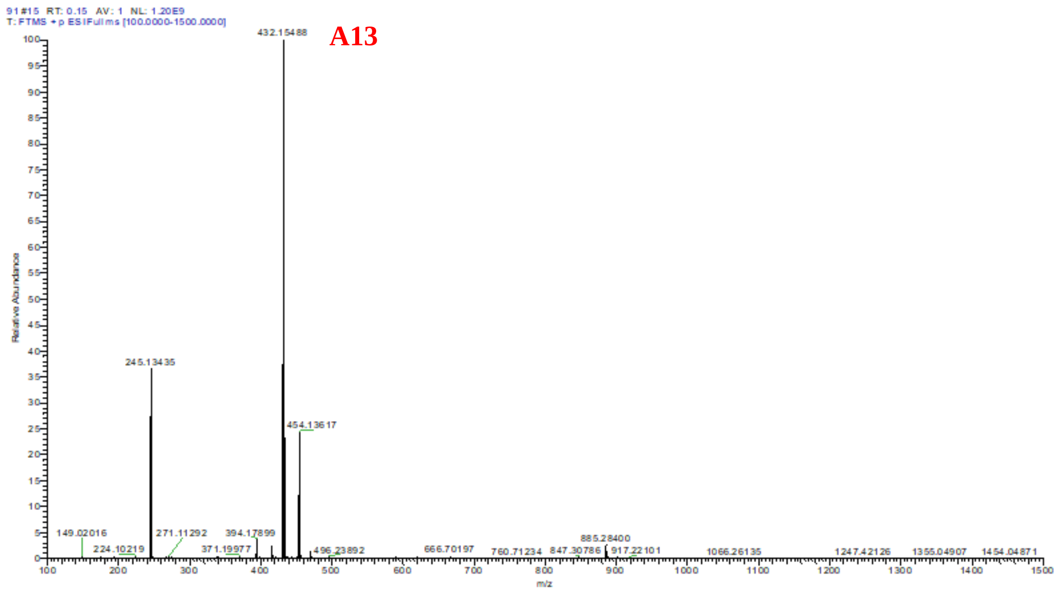
91 #15 RT: 0.15 AV: 1 NL: 1.20E9
T: FTMS + p ESI Full ms [100.0000-1500.0000]
A13
432.15488
245.13435
454.13617
149.02016
224.10219
271.11292
394.17899
371.19977
496.23892
666.70197
760.71234
847.30786
885.28400
917.22101
1066.26135
1247.42126
1355.04907
1454.04871
Relative Abundance
m/z

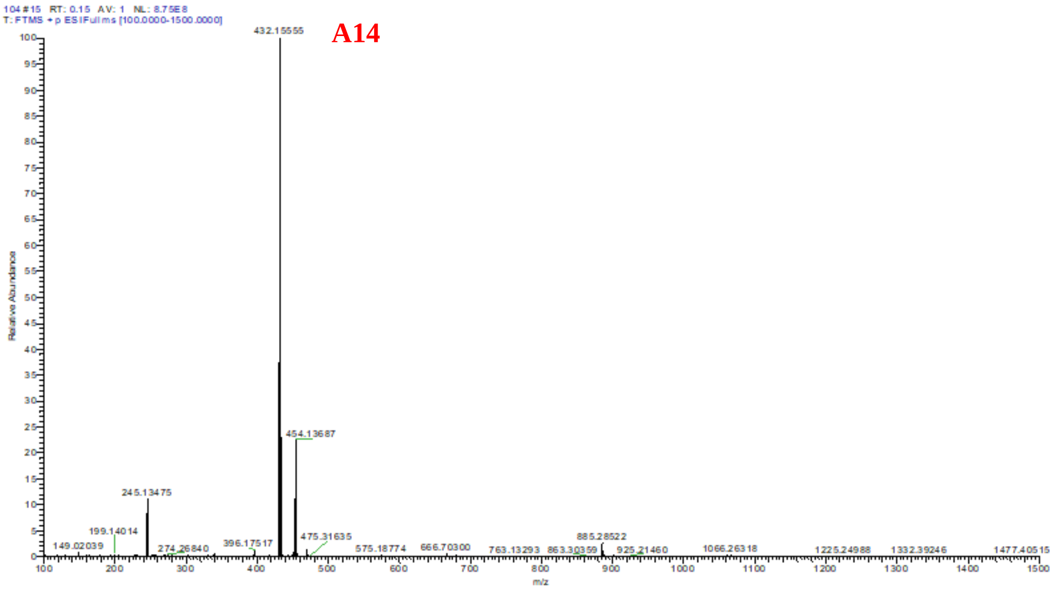
104 #15 RT: 0.15 AV: 1 NL: 8.75E8
T: FTMS + p ESI Full ms [100.0000-1500.0000]
A14
432.15555
454.13687
245.13475
199.14014
149.02039
274.26840
396.17517
475.31635
575.18774
666.70300
763.13293
863.30359
885.28522
925.21460
1066.26318
1225.24988
1332.39246
1477.40515
Relative Abundance
m/z

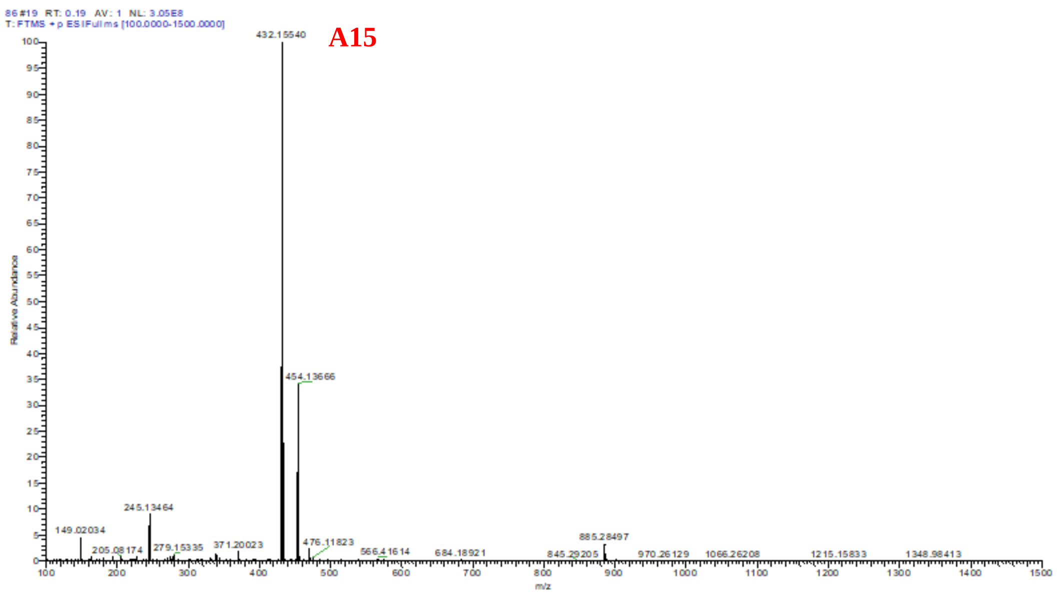

86 #19 RT: 0.19 AV: 1 NL: 3.05E8
T: FTMS + p ESI Full ms [100.0000-1500.0000]
432.15540
A15
454.13666
245.13464
149.02034
205.08174
279.15335
371.20023
476.11823
566.41614
684.18921
845.29205
885.28497
970.26129
1066.26208
1215.15833
1348.98413
Relative Abundance
m/z

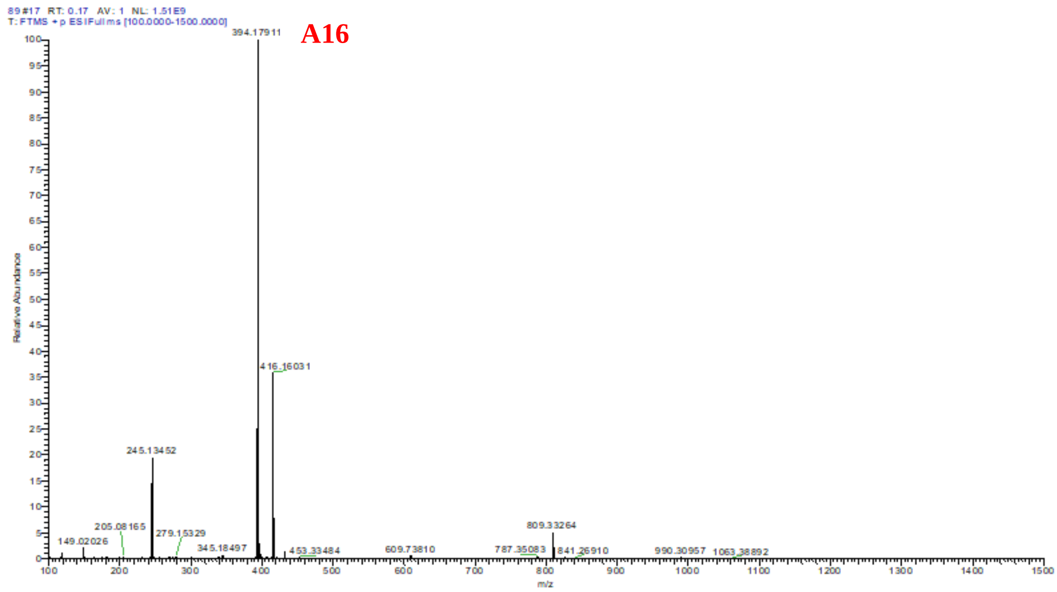

89 #17 RT: 0.17 AV: 1 NL: 1.51E9
T: FTMS + p ESI Full ms [100.0000-1500.0000]
394.17911
A16
416.16031
245.13452
205.08165
149.02026
279.15329
345.18497
453.33484
609.73810
787.35083
809.33264
841.26910
990.30957
1063.38892
Relative Abundance
m/z

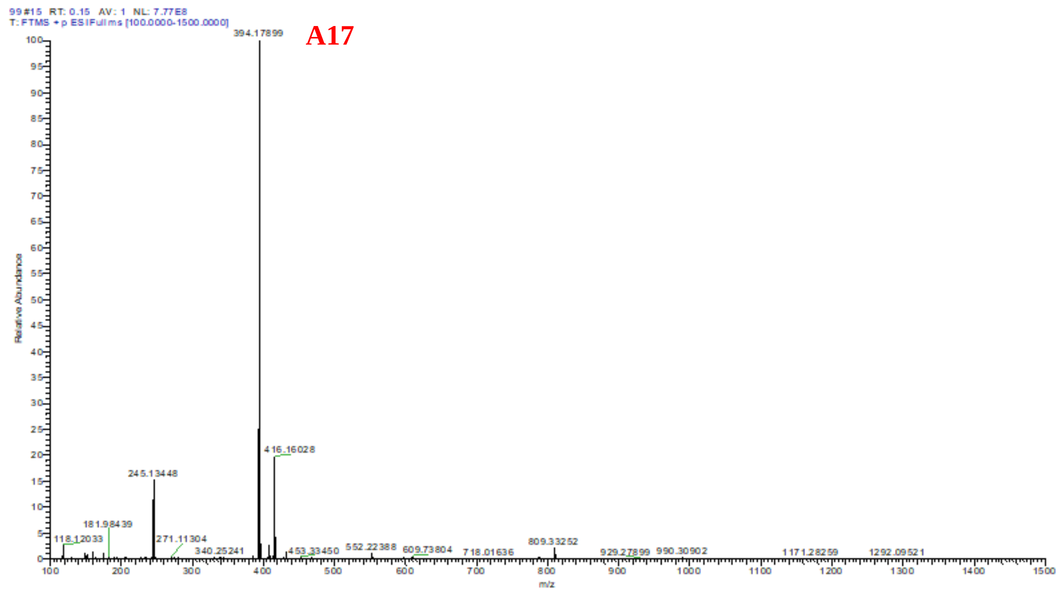
99 #15 RT: 0.15 AV: 1 NL: 7.77E8
T: FTMS + p ESI Full ms [100.0000-1500.0000]
A17
394.17899
416.16028
245.13448
181.98439
118.12033
271.11304
340.25241
453.33450
552.22388
609.73804
718.01636
809.33252
929.27899
990.30902
1171.28259
1292.09521
Relative Abundance
m/z

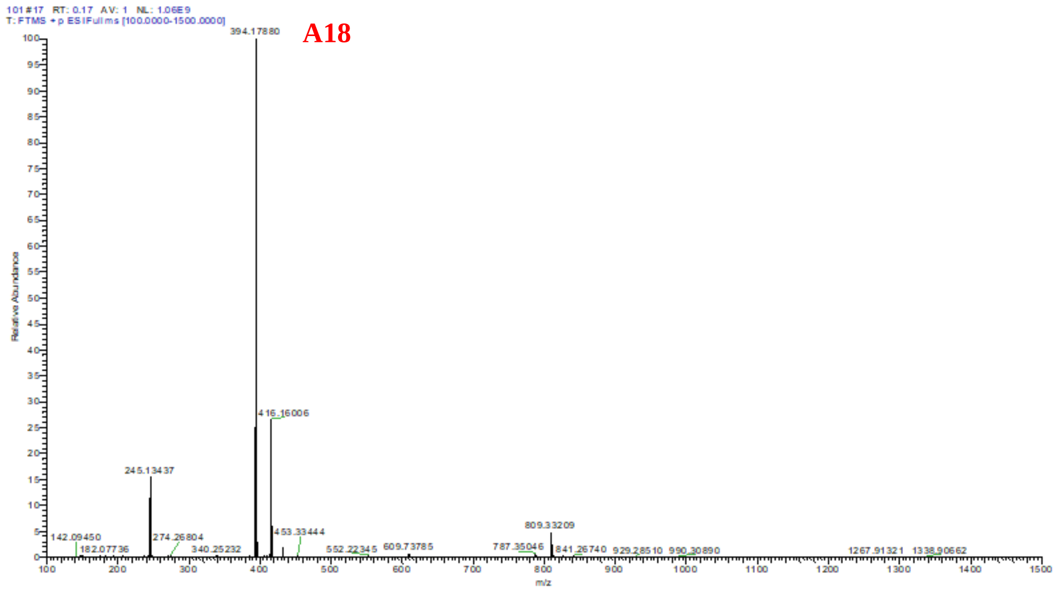
101 #17 RT: 0.17 AV: 1 NL: 1.06E9
T: FTMS + p ESI Full ms [100.0000-1500.0000]
A18
394.17880
416.16006
245.13437
142.09450
182.07736
274.26804
340.25232
453.33444
552.22345
609.73785
787.35046
809.33209
841.26740
929.28510
990.30890
1267.91321
1338.90662
Relative Abundance
m/z

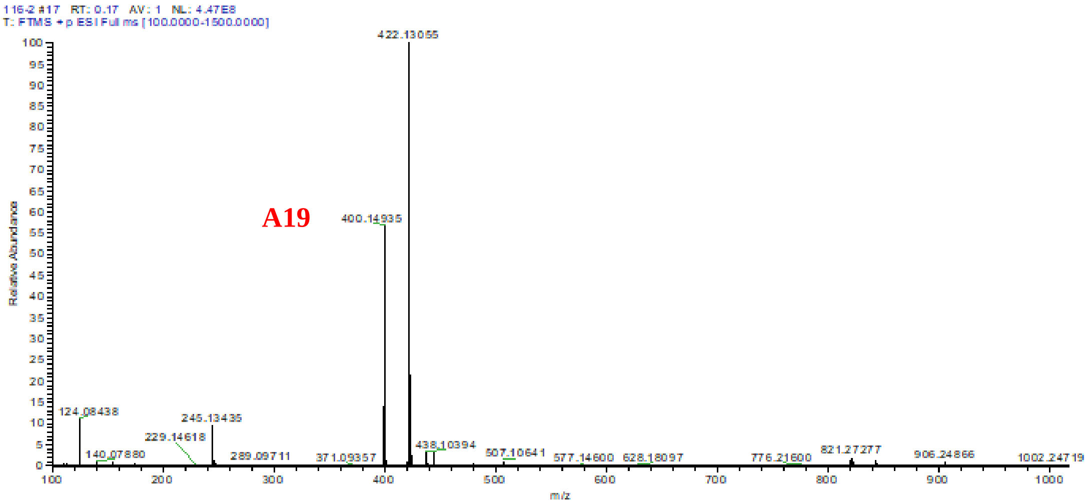
116-2 #17 RT: 0.17 AV: 1 NL: 4.47E8
T: FTMS + p ESI Full ms [100.0000-1500.0000]
A19
422.13055
400.14935
124.08438
245.13435
229.14618
140.07880
289.09711
371.09357
438.10394
507.10641
577.14600
628.18097
776.21600
821.27277
906.24866
1002.24719
Relative Abundance
m/z

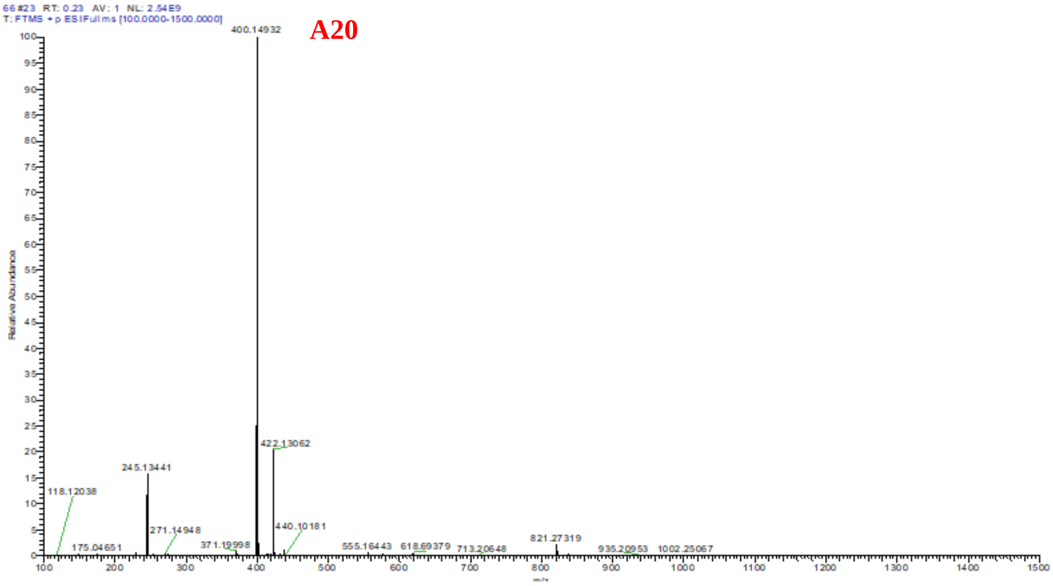
66 #23 RT: 0.23 AV: 1 NL: 2.54E9
T: FTMS + p ESI Full ms [100.0000-1500.0000]
A20
400.14932
422.13062
245.13441
118.12038
271.14948
175.04651
371.19998
440.10181
555.16443
618.69379
713.20648
821.27319
935.20953
1002.25067
Relative Abundance

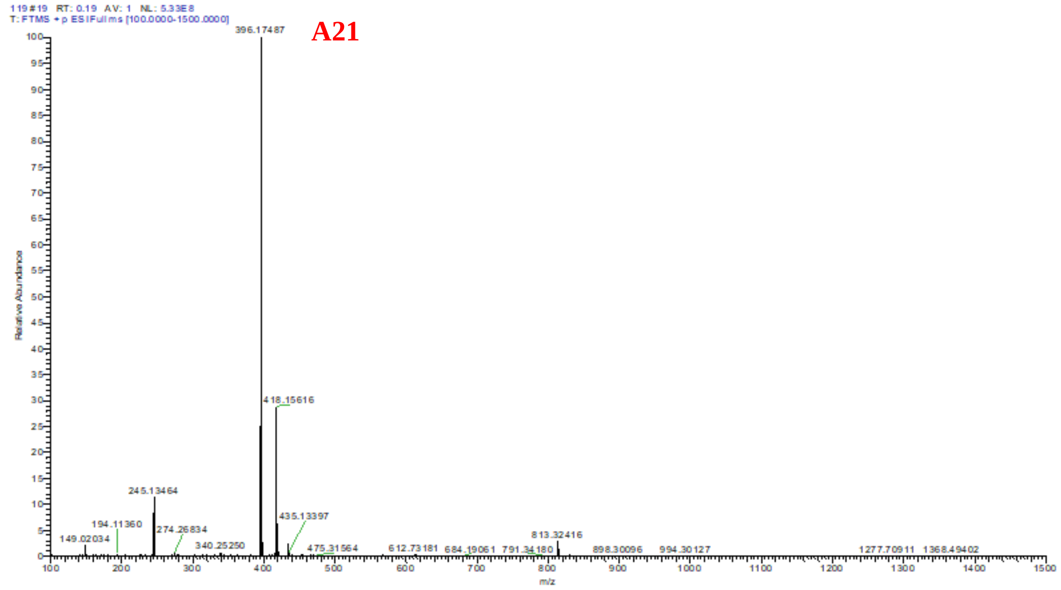
119 #19 RT: 0.19 AV: 1 NL: 5.33E8
T: FTMS + p ESI Full ms [100.0000-1500.0000]
A21
396.17487
418.15616
245.13464
435.13397
194.11360
274.26834
149.02034
340.25250
475.31564
612.73181
684.19061
791.34180
813.32416
898.30096
994.30127
1277.70911
1368.49402
Relative Abundance
m/z

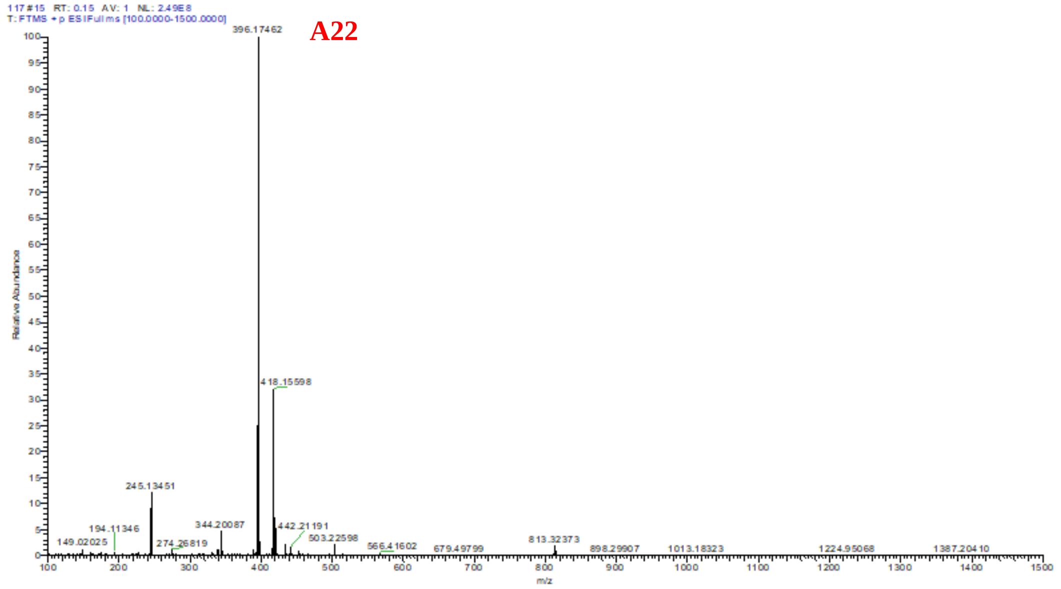
117 #15 RT: 0.15 AV: 1 NL: 2.49E8
T: FTMS + p ESI Full ms [100.0000-1500.0000]
A22
396.17462
418.15598
245.13451
194.11346
344.20087
442.21191
149.02025
274.26819
503.22598
566.41602
679.49799
813.32373
898.29907
1013.18323
1224.95068
1387.20410
Relative Abundance
m/z

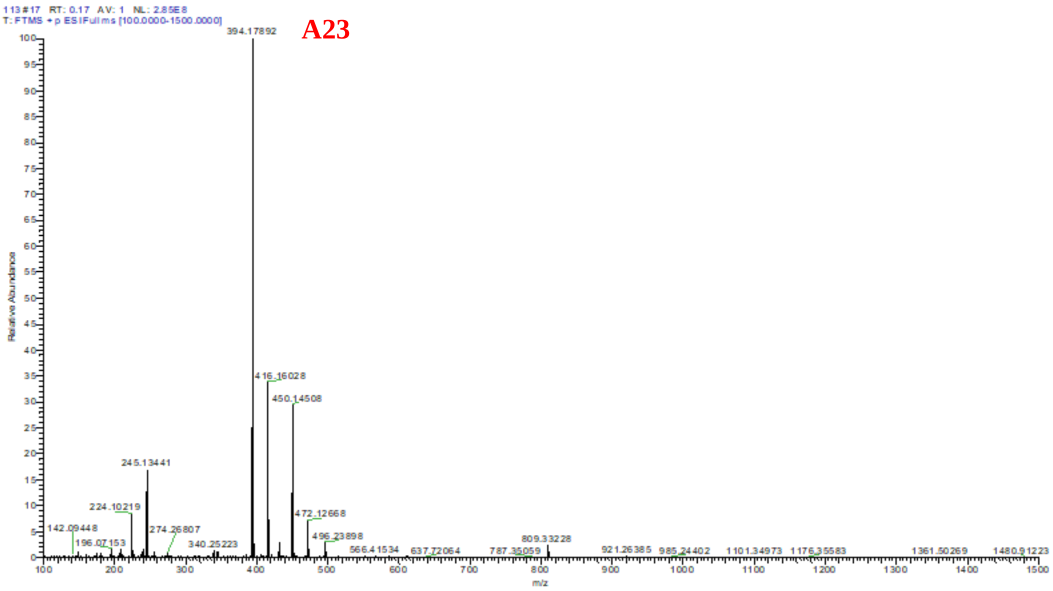

113 #17 RT: 0.17 AV: 1 NL: 2.85E8
T: FTMS + p ESI Full ms [100.0000-1500.0000]
A23
394.17892
416.16028
450.14508
245.13441
224.10219
142.09448
196.07153
274.26807
340.25223
472.12668
496.23898
566.41534
637.72064
787.35059
809.33228
921.26385
985.24402
1101.34973
1176.35583
1361.50269
1480.91223
Relative Abundance
m/z

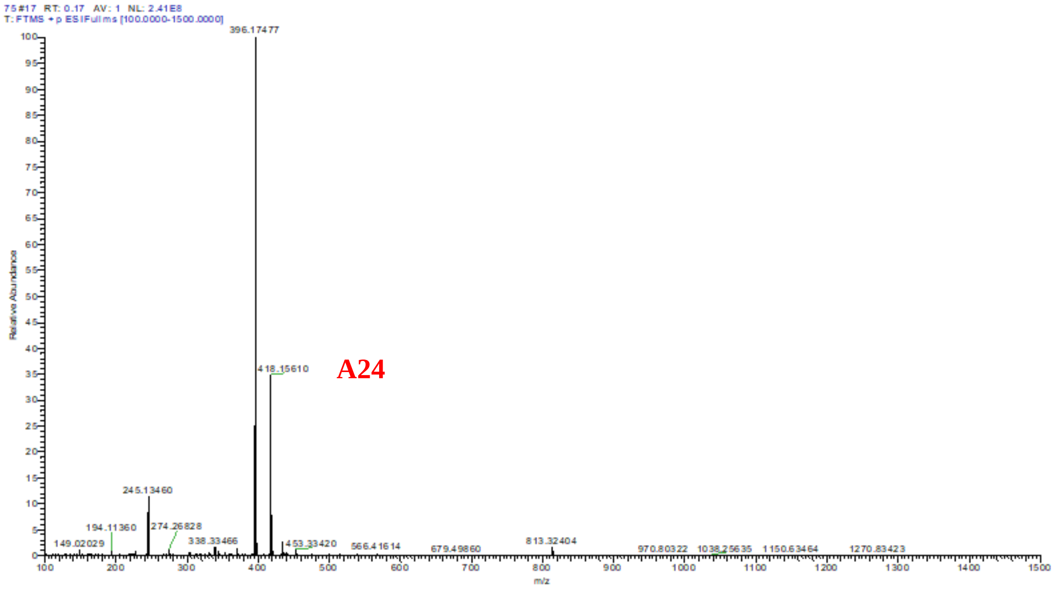

75 #17 RT: 0.17 AV: 1 NL: 2.41E8
T: FTMS + p ESI Full ms [100.0000-1500.0000]
396.17477
418.15610
A24
245.13460
194.11360
149.02029
274.26828
338.33466
453.33420
566.41614
679.49860
813.32404
970.80322
1038.25635
1150.63464
1270.83423
Relative Abundance
m/z

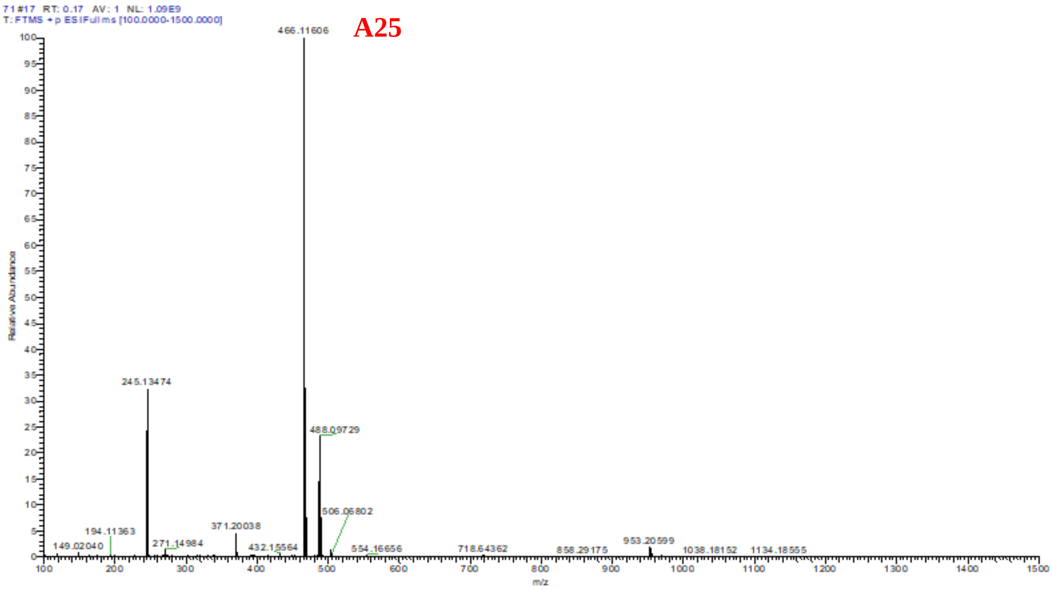
71 #17 RT: 0.17 AV: 1 NL: 1.09E9
T: FTMS + p ESI Full ms [100.0000-1500.0000]
A25
466.11606
245.13474
488.09729
506.06802
194.11363
371.20038
149.02040
271.14984
432.15564
554.16656
718.64362
858.29175
953.20599
1038.18152
1134.18555
Relative Abundance
m/z

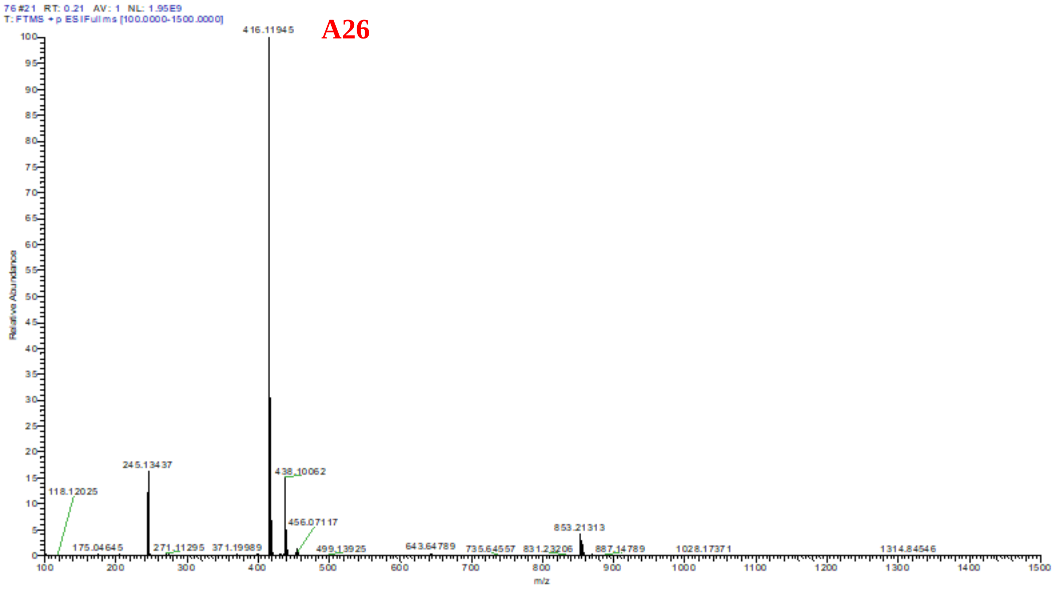
76 #21 RT: 0.21 AV: 1 NL: 1.95E9
T: FTMS + p ESI Full ms [100.0000-1500.0000]
A26
416.11945
245.13437
438.10062
118.12025
456.07117
853.21313
175.04645
271.11295
371.19989
499.13925
643.64789
735.64557
831.23206
887.14789
1028.17371
1314.84546
Relative Abundance
m/z

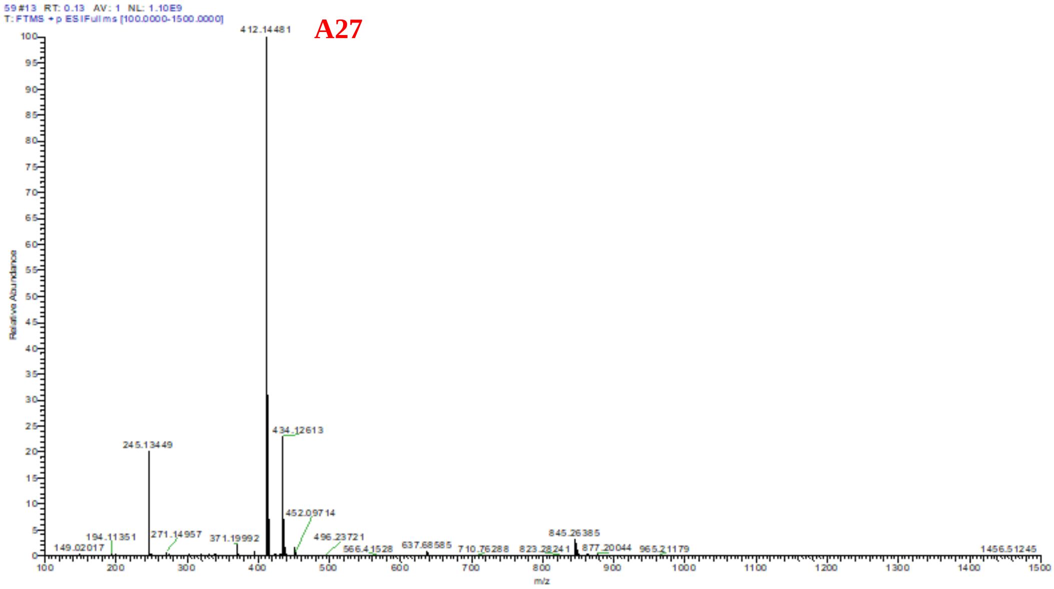
59 #13 RT: 0.13 AV: 1 NL: 1.10E9
T: FTMS + p ESI Full ms [100.0000-1500.0000]
A27
412.14481
434.12613
245.13449
452.09714
845.26385
Relative Abundance
m/z

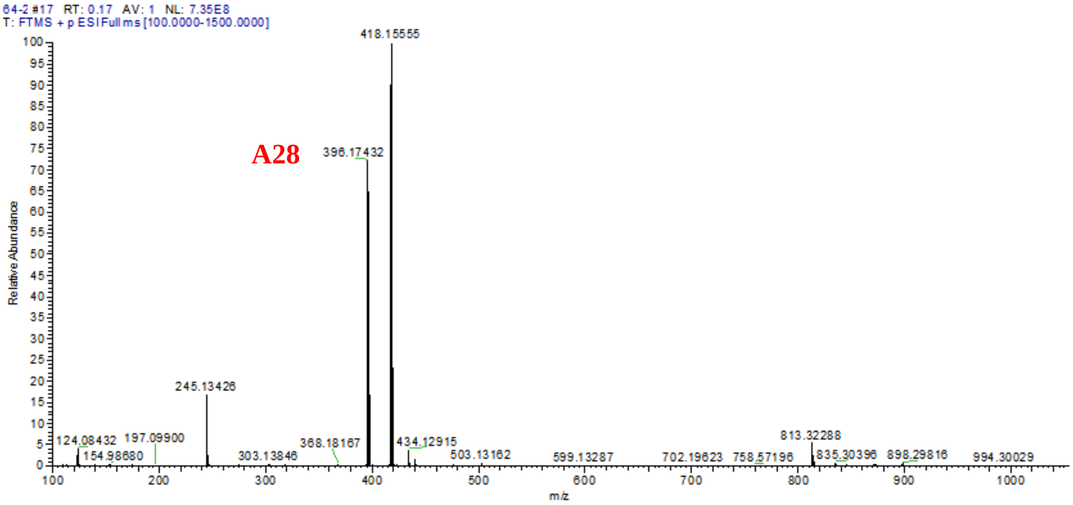
64-2 #17 RT: 0.17 AV: 1 NL: 7.35E8
T: FTMS + p ESI Full ms [100.0000-1500.0000]
A28
418.15555
396.17432
245.13426
813.32288
Relative Abundance
m/z

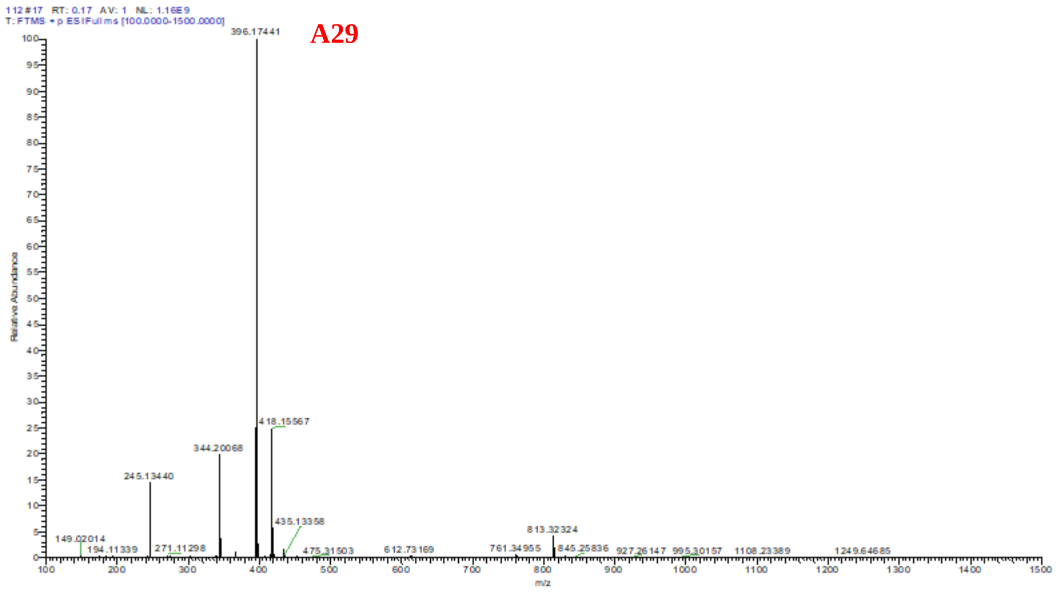
112 #17 RT: 0.17 AV: 1 NL: 1.16E9
T: FTMS + p ESI Full ms [100.0000-1500.0000]
A29
396.17441
418.15567
344.20068
245.13440
435.13358
813.32324
Relative Abundance
m/z

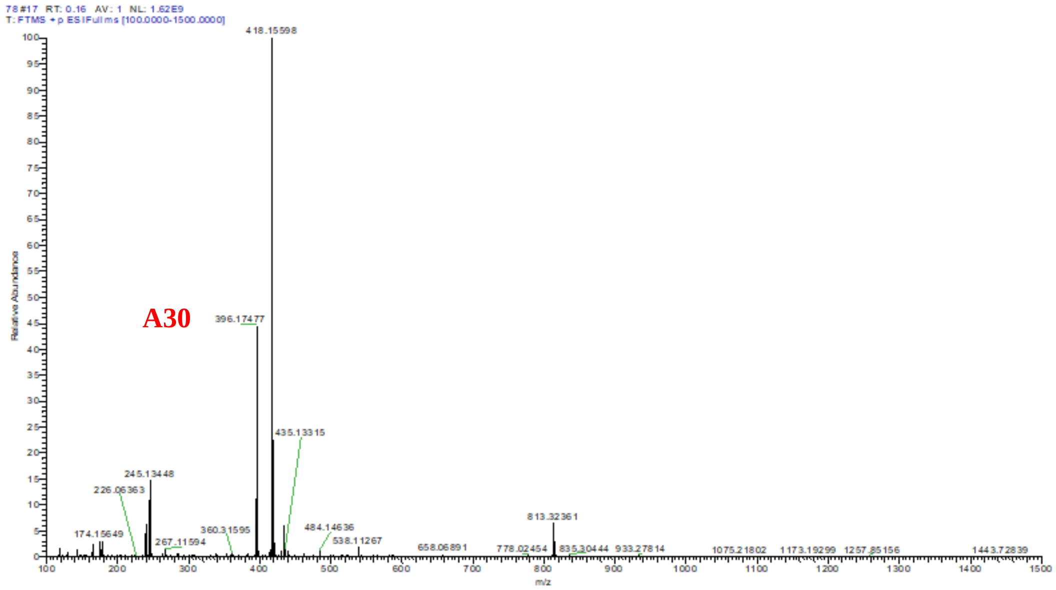
78 #17 RT: 0.16 AV: 1 NL: 1.62E9
T: FTMS + p ESI Full ms [100.0000-1500.0000]
418.15598
A30
396.17477
435.13315
245.13448
226.06363
813.32361
Relative Abundance
m/z

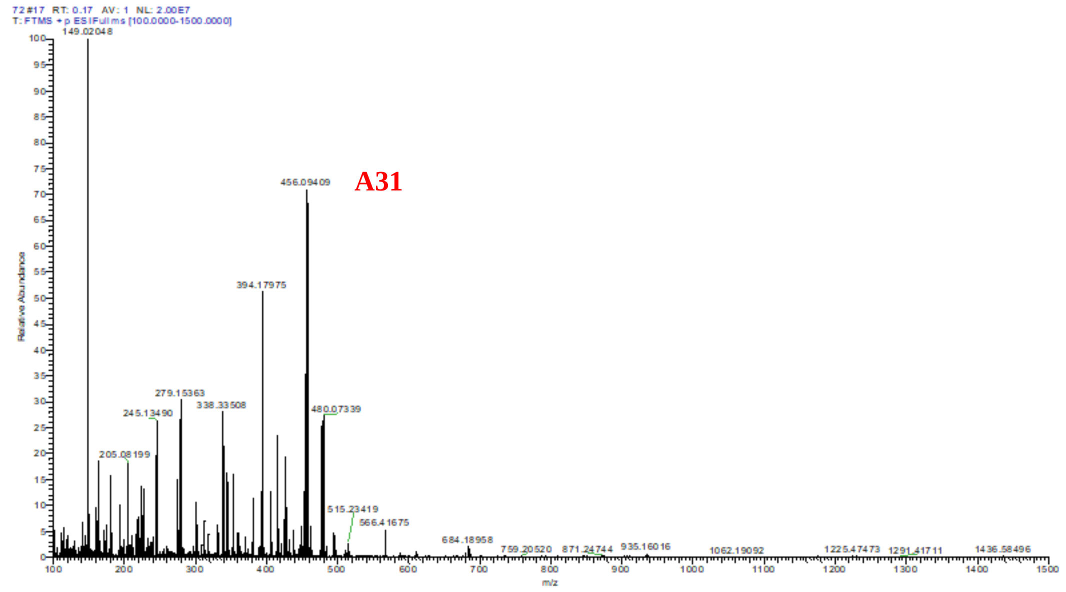
72 #17 RT: 0.17 AV: 1 NL: 2.00E7
T: FTMS + p ESI Full ms [100.0000-1500.0000]
A31
Relative Abundance
m/z

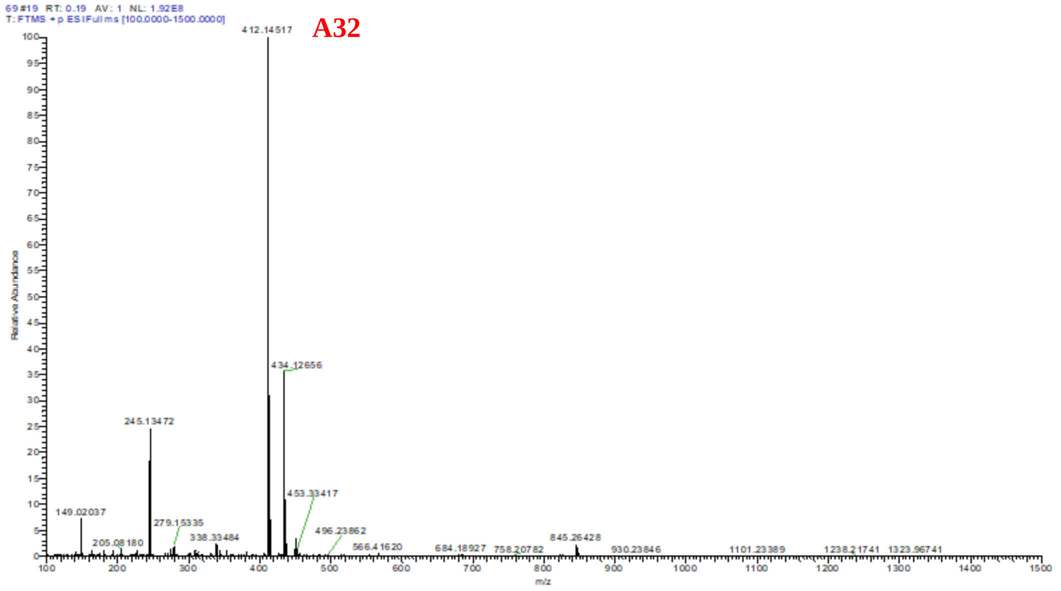
69 #19 RT: 0.19 AV: 1 NL: 1.92E8
T: FTMS + p ESI Full ms [100.0000-1500.0000]
A32
Relative Abundance
m/z

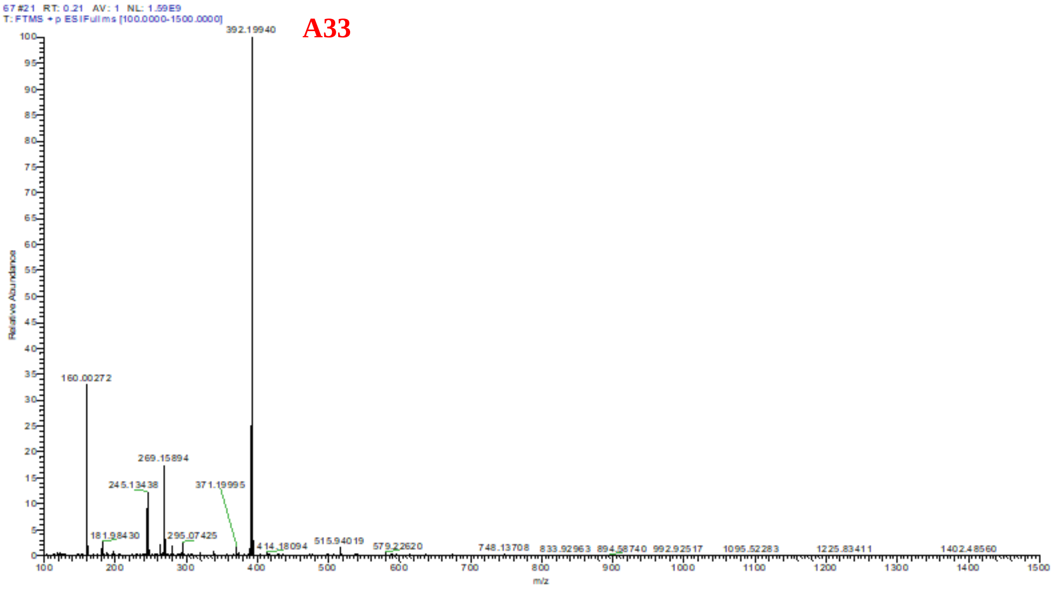
67 #21 RT: 0.21 AV: 1 NL: 1.59E9
T: FTMS + p ESI Full ms [100.0000-1500.0000]
392.19940
160.00272
269.15894
245.13438
371.19995
181.98430
295.07425
414.18094
515.94019
579.22620
748.13708
833.92963
894.58740
992.92517
1095.52283
1225.83411
1402.48560
Relative Abundance
m/z

A33

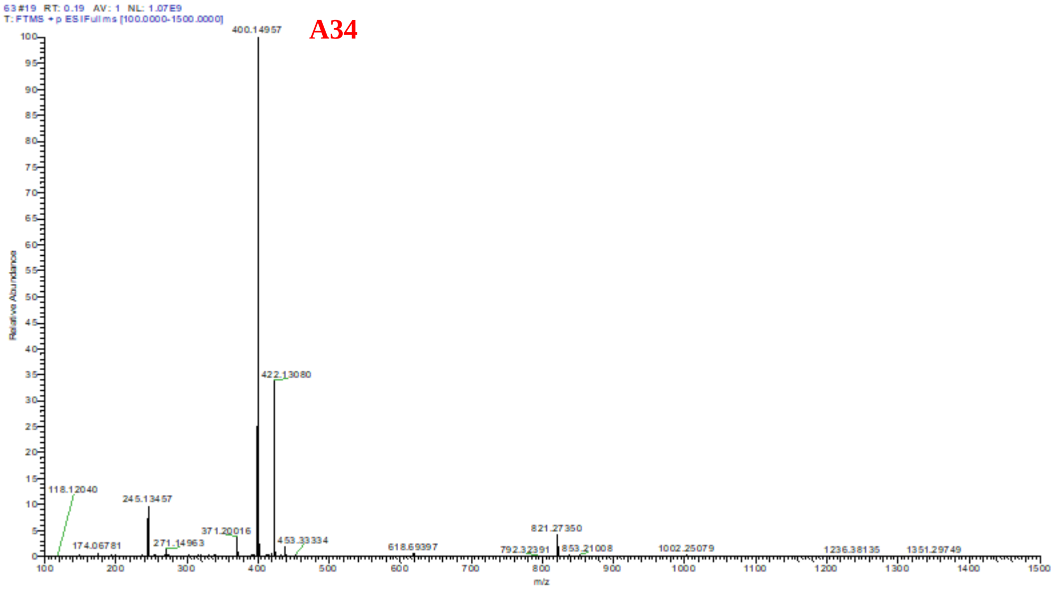
63 #19 RT: 0.19 AV: 1 NL: 1.07E9
T: FTMS + p ESI Full ms [100.0000-1500.0000]
400.14957
422.13080
118.12040
245.13457
371.20016
174.06781
271.14963
453.33334
618.69397
821.27350
792.32391
853.21008
1002.25079
1236.38135
1351.29749
Relative Abundance
m/z

A34

A35

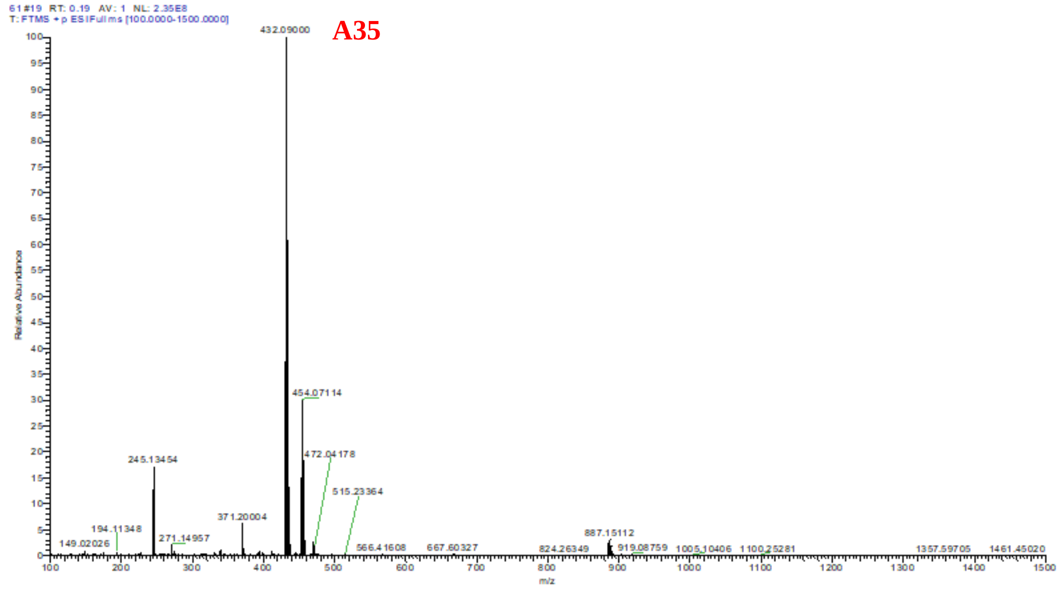
61 #19 RT: 0.19 AV: 1 NL: 2.35E8
T: FTMS + p ESI Full ms [100.0000-1500.0000]
432.09000
454.07114
472.04178
515.23364
245.13454
371.20004
194.11348
271.14957
149.02026
566.41608
667.60327
824.26349
887.15112
919.08759
1005.10406
1100.25281
1357.59705
1461.45020
Relative Abundance
m/z

A36

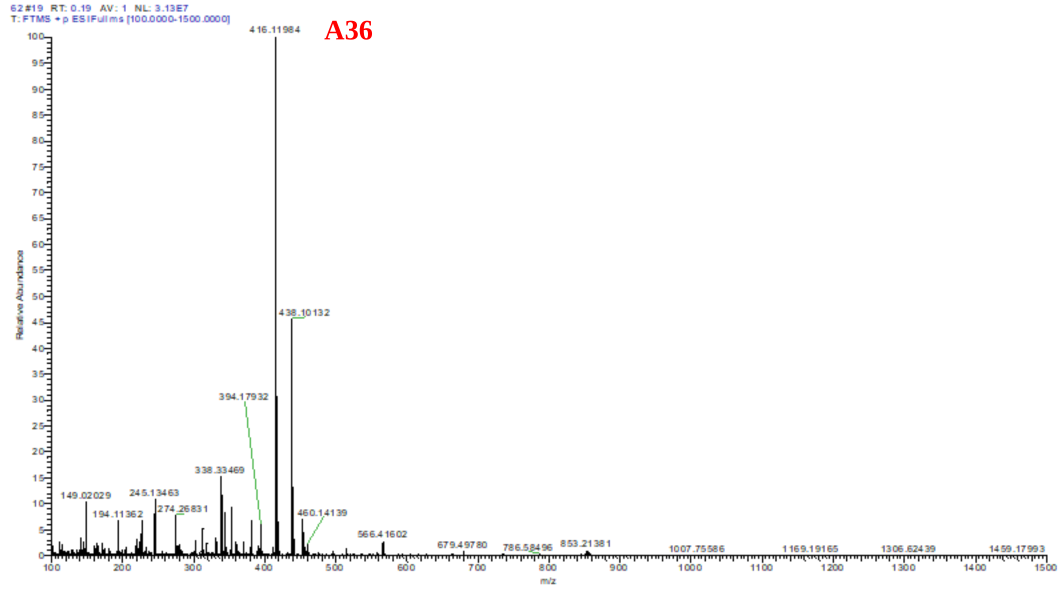
62 #19 RT: 0.19 AV: 1 NL: 3.13E7
T: FTMS + p ESI Full ms [100.0000-1500.0000]
416.11984
438.10132
394.17932
338.33469
149.02029
245.13463
194.11362
274.26831
460.14139
566.41602
679.49780
786.58496
853.21381
1007.75586
1169.19165
1306.62439
1459.17993
Relative Abundance
m/z

A37

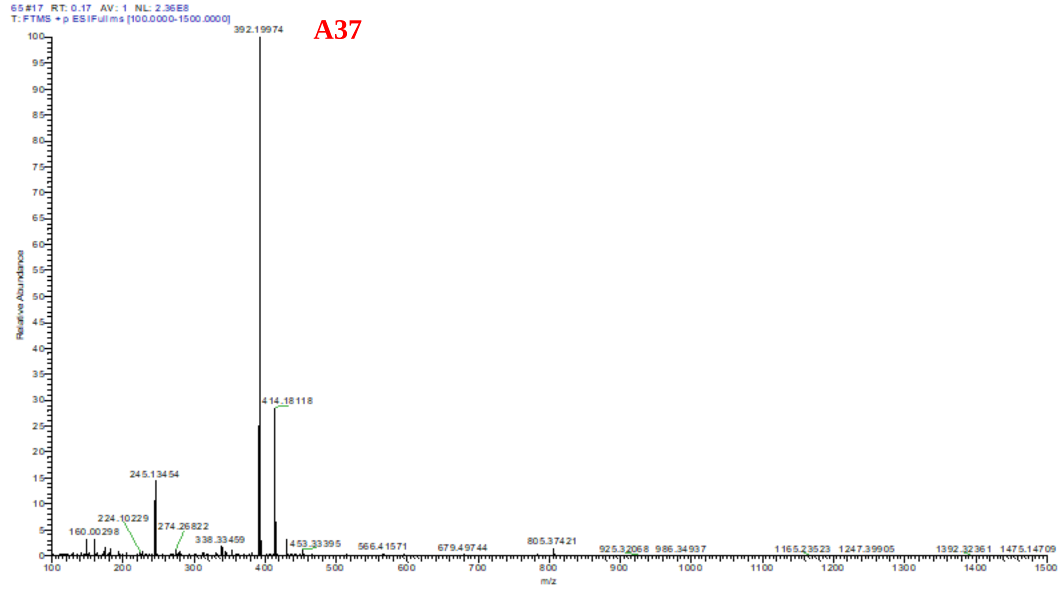
65 #17 RT: 0.17 AV: 1 NL: 2.36E8
T: FTMS + p ESI Full ms [100.0000-1500.0000]
392.19974
414.18118
245.13454
224.10229
160.00298
274.26822
338.33459
453.33395
566.41571
679.49744
805.37421
925.32068
986.34937
1165.23523
1247.39905
1392.32361
1475.14709
Relative Abundance
m/z

A38

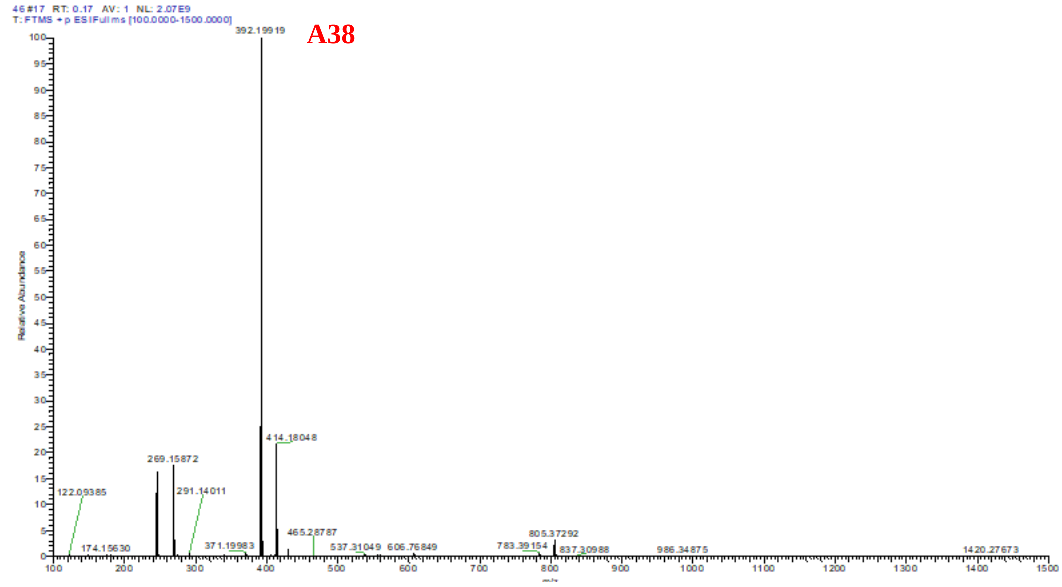
46 #17 RT: 0.17 AV: 1 NL: 2.07E9
T: FTMS + p ESI Full ms [100.0000-1500.0000]
392.19919
414.18048
269.15872
122.09385
291.14011
174.15630
371.19983
465.28787
537.31049
606.76849
783.39154
805.37292
837.30988
986.34875
1420.27673
Relative Abundance

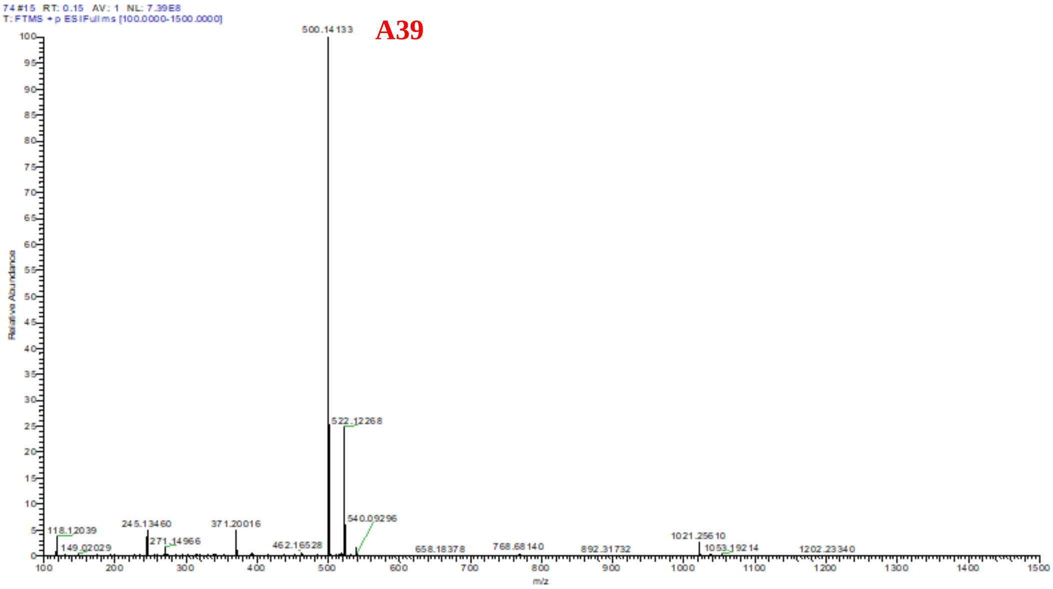
74 #15 RT: 0.15 AV: 1 NL: 7.39E8
T: FTMS + p ESI Full ms [100.0000-1500.0000]
A39
500.14133
522.12268
540.09296
118.12039
149.02029
245.13460
271.14966
371.20016
462.16528
658.18378
768.68140
892.31732
1021.25610
1053.19214
1202.23340
Relative Abundance
m/z

4.2 Mass spectrum of B1-B7

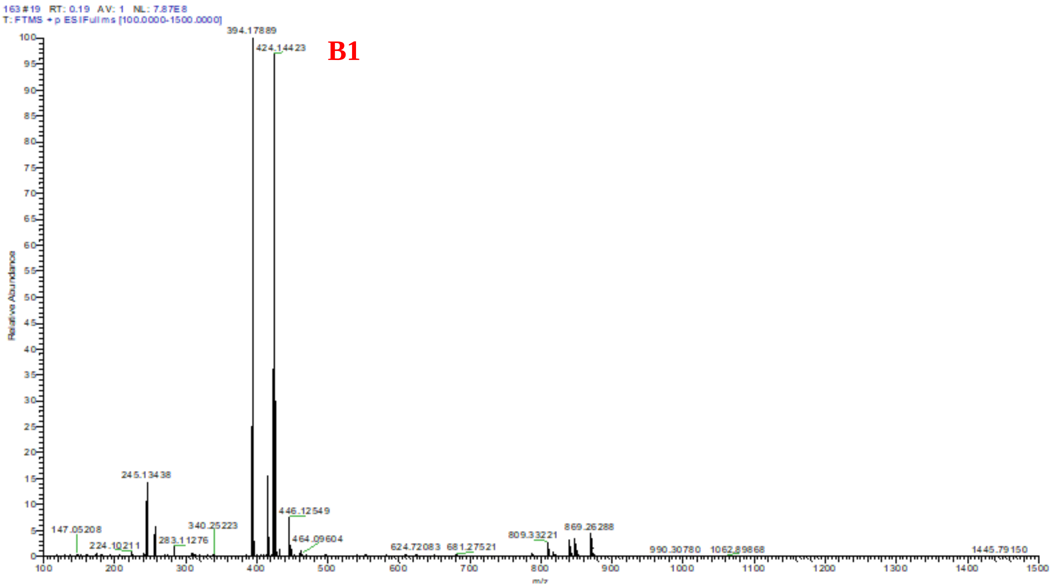

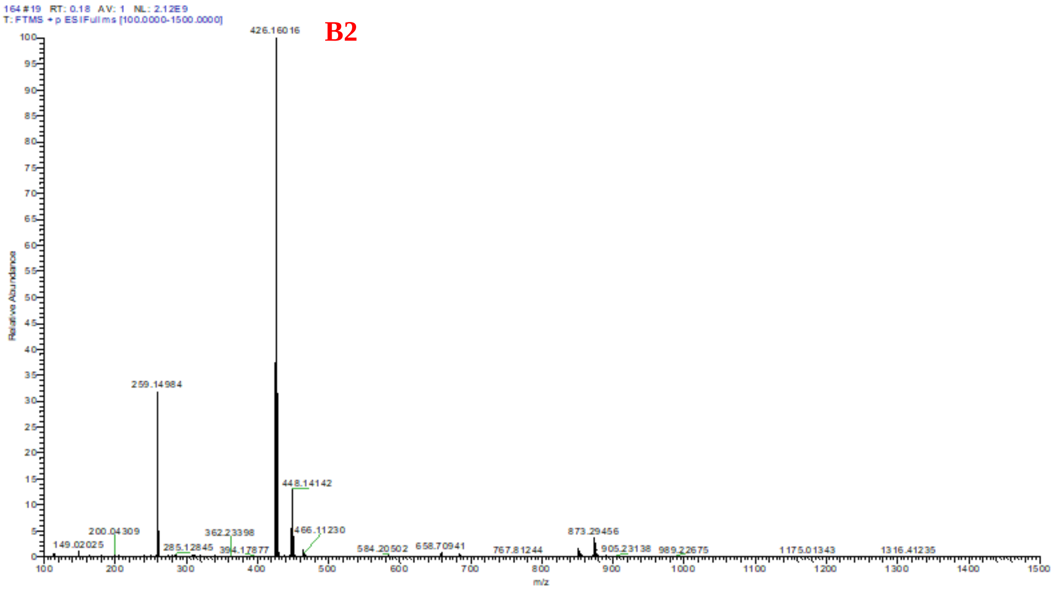

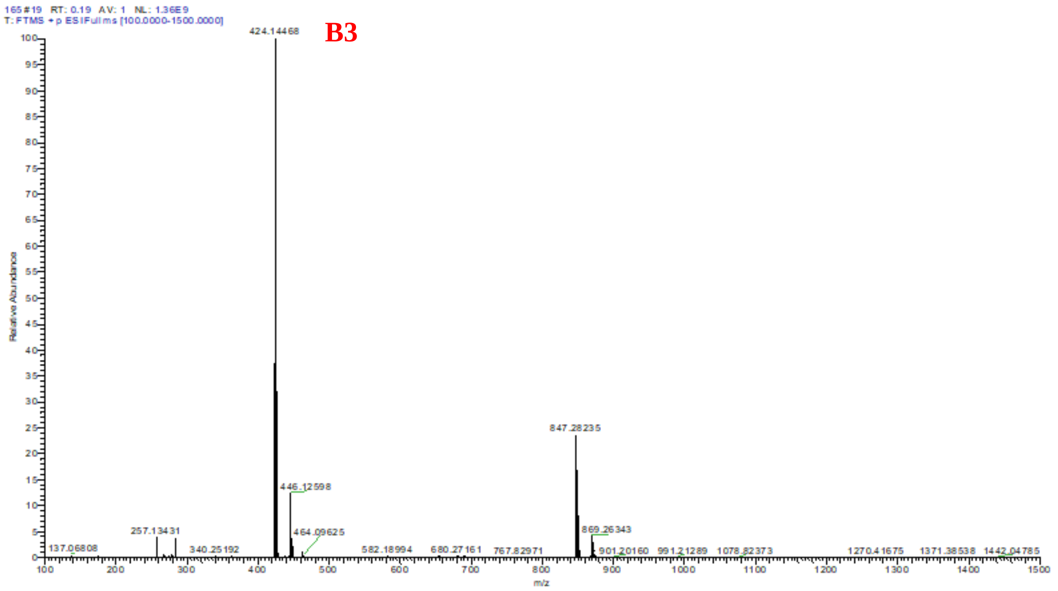
165 #19 RT: 0.19 AV: 1 NL: 1.36E9
T: FTMS + p ESI Full ms [100.0000-1500.0000]
B3
424.14468
847.28235
446.12598
464.09625
869.26343
257.13431
137.06808
340.25192
582.18994
680.27161
767.82971
901.20160
991.21289
1078.82373
1270.41675
1371.38538
1442.04785
Relative Abundance
m/z

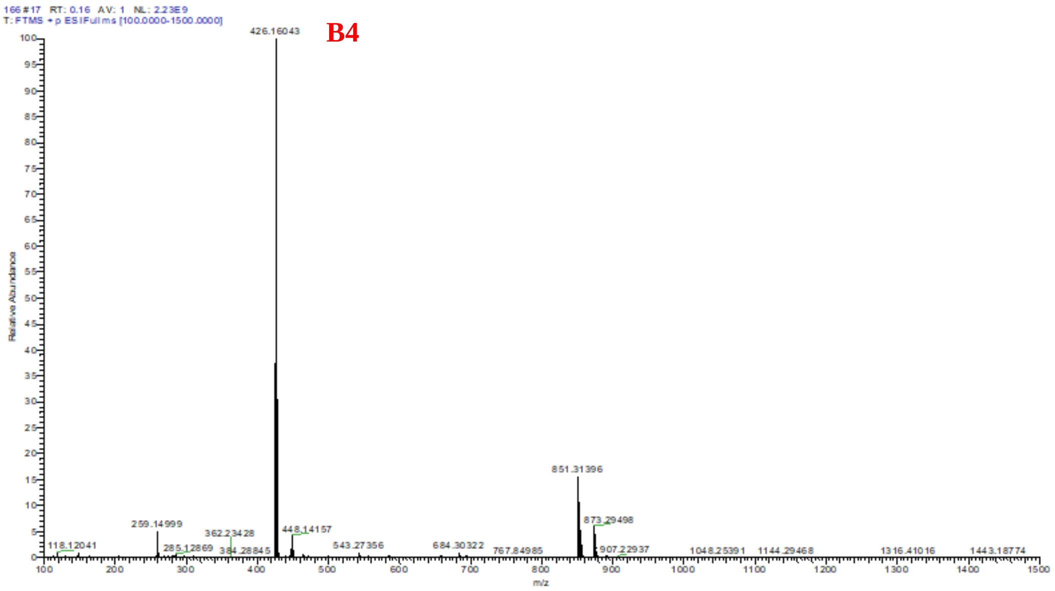
166 #17 RT: 0.16 AV: 1 NL: 2.23E9
T: FTMS + p ESI Full ms [100.0000-1500.0000]
B4
426.16043
851.31396
873.29498
259.14999
362.23428
448.14157
118.12041
285.12869
384.28845
543.27356
684.30322
767.84985
907.22937
1048.25391
1144.29468
1316.41016
1443.18774
Relative Abundance
m/z

B5

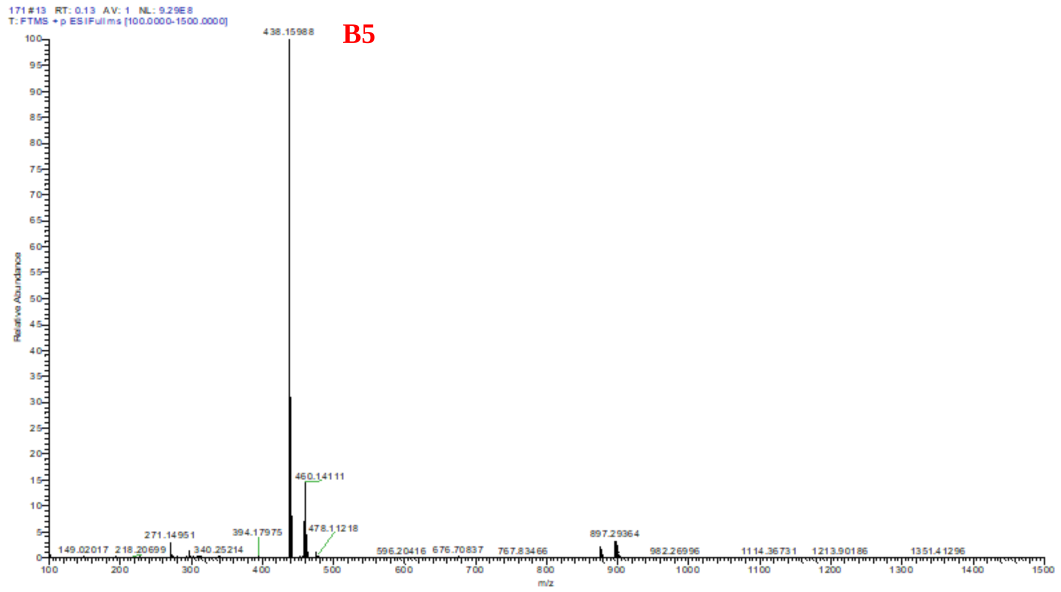

171 #13 RT: 0.13 AV: 1 NL: 9.29E8
T: FTMS + p ESI Full ms [100.0000-1500.0000]
Relative Abundance
m/z
438.15988
460.14111
478.11218
271.14951
394.17975
897.29364

B6

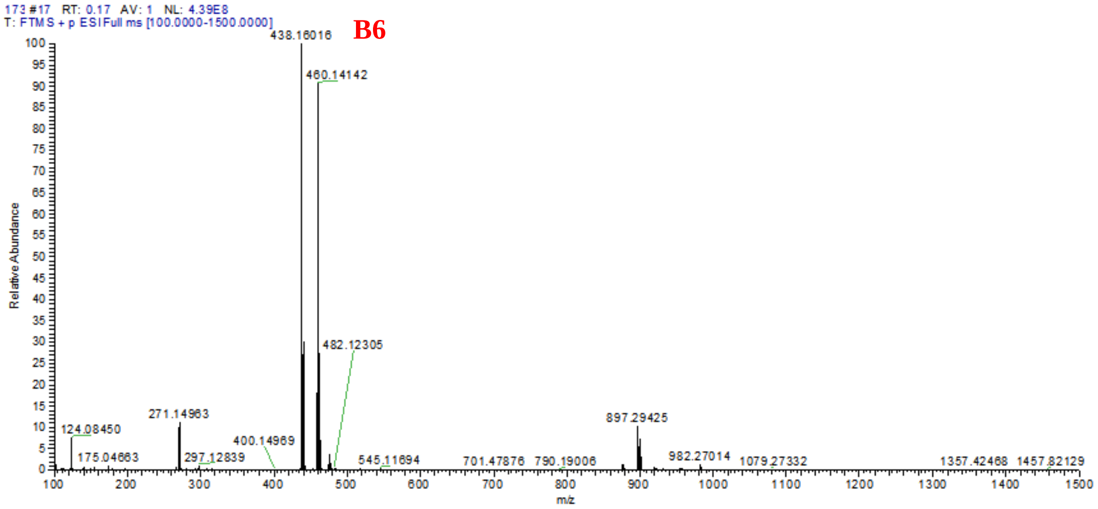

173 #17 RT: 0.17 AV: 1 NL: 4.39E8
T: FTMS + p ESI Full ms [100.0000-1500.0000]
Relative Abundance
m/z
438.16016
460.14142
482.12305
271.14963
124.08450
400.14969
897.29425
982.27014

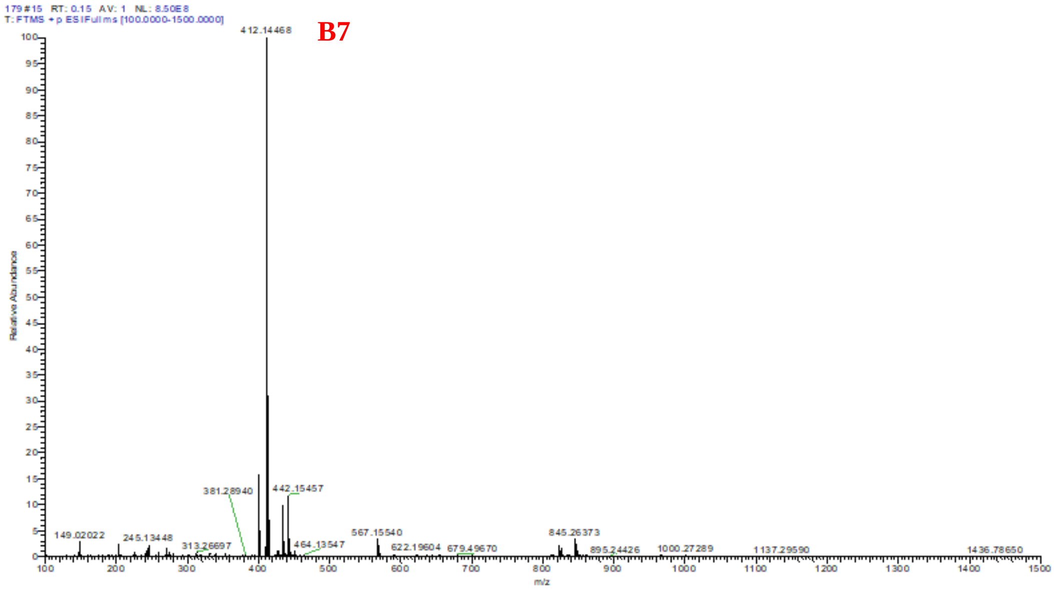
179 #15 RT: 0.15 AV: 1 NL: 8.50E8
T: FTMS + p ESI Full ms [100.0000-1500.0000]
B7
412.14468
381.28940
442.15457
149.02022
245.13448
313.26697
464.13547
567.15540
622.19604
679.49670
845.26373
895.24426
1000.27289
1137.29590
1436.78650
Relative Abundance
m/z

4.3 Mass spectrum of C1-C5

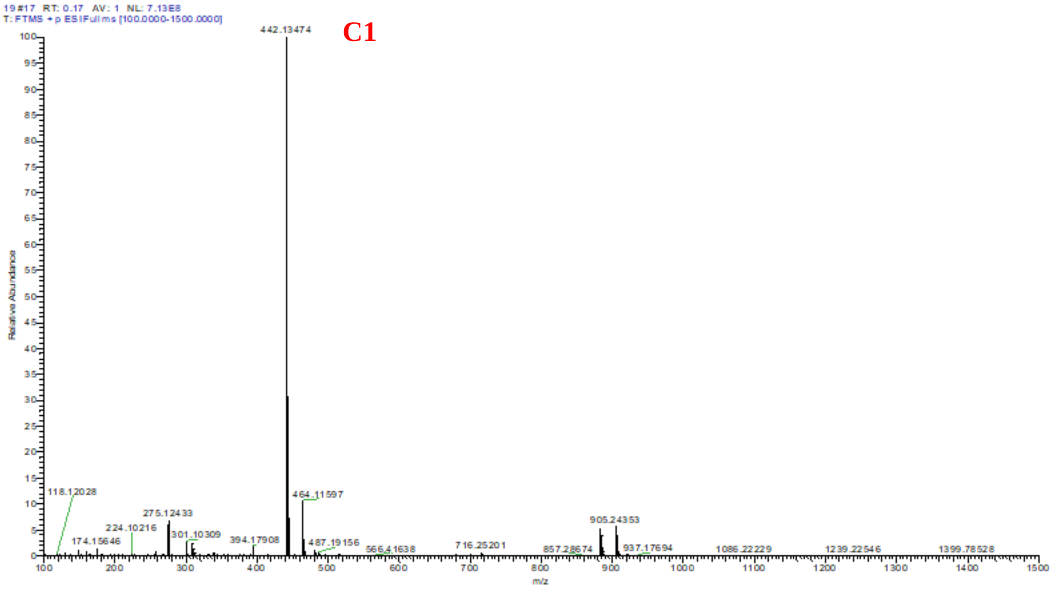

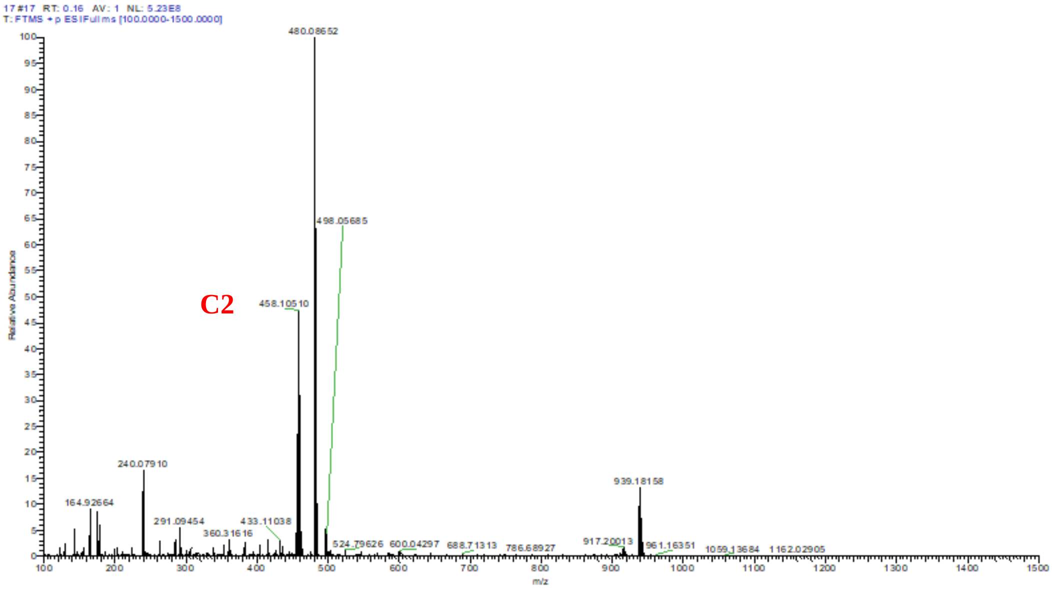

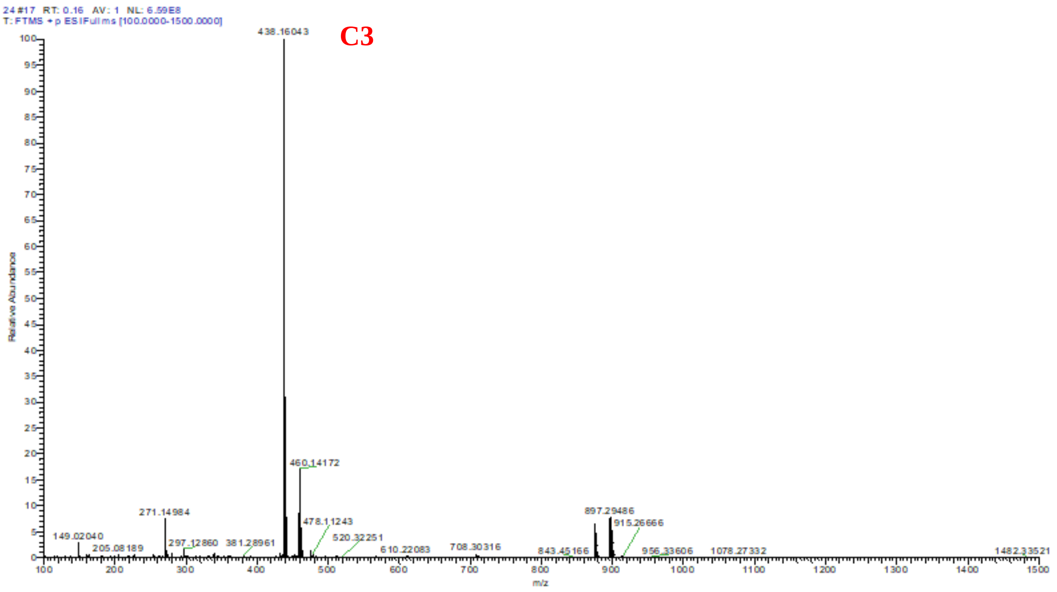
24 #17 RT: 0.16 AV: 1 NL: 6.59E8
T: FTMS + p ESI Full ms [100.0000-1500.0000]
C3
438.16043
460.14172
271.14984
897.29486
915.26666
478.11243
520.32251
149.02040
205.08189
297.12860
381.28961
610.22083
708.30316
843.45166
956.33606
1078.27332
1482.33521
Relative Abundance
m/z

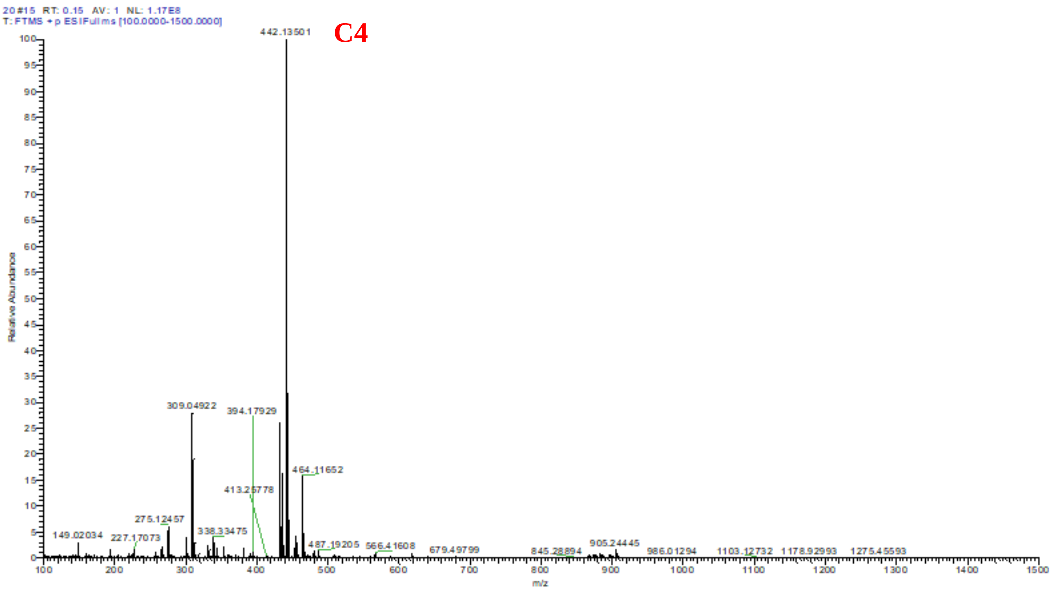
20 #15 RT: 0.15 AV: 1 NL: 1.17E8
T: FTMS + p ESI Full ms [100.0000-1500.0000]
C4
442.13501
309.04922
394.17929
464.11652
413.25778
275.12457
338.33475
149.02034
227.17073
487.19205
566.41608
679.49799
845.28894
905.24445
986.01294
1103.12732
1178.92993
1275.45593
Relative Abundance
m/z

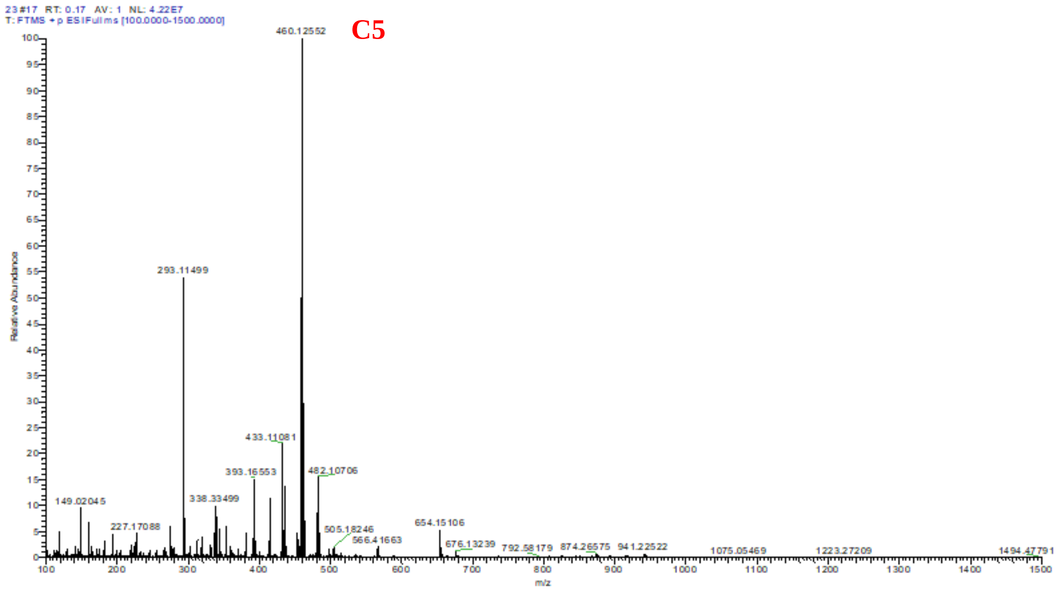
23 #17 RT: 0.17 AV: 1 NL: 4.22E7
T: FTMS + p ESI Full ms [100.0000-1500.0000]
C5
460.12552
293.11499
433.11081
393.16553
482.10706
338.33499
149.02045
227.17088
505.18246
566.41663
654.15106
676.13239
792.58179
874.26575
941.22522
1075.05469
1223.27209
1494.47791
Relative Abundance
m/z

S6: IR Spectral of compound B1

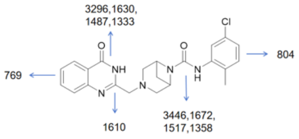

IR (KBr, cm^{-1}): 3446, 3296 (2NH), 3244-3045 (CH, aromatic), 2981-2821 (CH-alicyclic), 1672, 1630 (2C=O), 1610 (C=N)

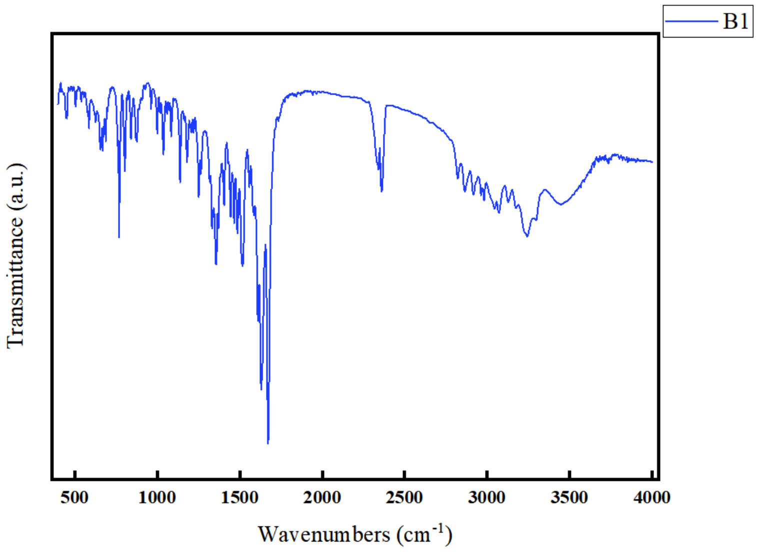

Supplement: Supplementary file 1 [file molecules-29-01407-s001.zip › molecules-2902860-supplementary.pdf]
